# Dynamic changes in chromatin accessibility reveal the role of NF-Y targeting AURKB in mediating cell cycle during asynchronous oogenesis in the Chinese Alligator (*Alligator sinensis*)

**DOI:** 10.1186/s12983-026-00611-8

**Published:** 2026-04-29

**Authors:** Pengfei Li, Yuting Ni, Yue Wen, Xiaojing Cao, Jin Li, Yongkang Zhou, Pingsi Yi, Xiaobing Wu, Haitao Nie

**Affiliations:** 1 College of Life Science, Anhui Normal University, Wuhu, 241000 Anhui Province People’s Republic of China; 2 The Anhui Provincial Key Laboratory of Biodiversity Conservation and Ecological Security in the Yangtze River Basin, Collaborative Innovation Center of Recovery and Reconstruction of Degraded Ecosystem in Wanjiang Basin Co-Founded By Anhui Province and Ministry of Education, Anhui Normal University, Wuhu, 241000 People’s Republic of China; 3 Alligator Research Center of Anhui Province, Xuanzhou, 242000 People’s Republic of China

**Keywords:** Chinese alligator (*Alligator sinensis*), Oogenesis, Chromatin accessibility, ATAC-seq, Transcription factor, NF-Y, *AURKB*

## Abstract

The dynamics of chromatin accessibility that regulate oogenesis for the establishment of the female ovarian reserve in mammals have not been explored in reptiles with lifelong continuous oogenesis. Assays for transposase-accessible chromatin with high-throughput sequencing (ATAC-seq) were conducted to generate a chromatin accessibility landscape from gonadal samples from three critical stages of oogenesis in female Chinese alligators, which revealed a pronounced global decrease in chromatin accessibility across these developmental stages. Functional enrichment analysis demonstrated that differentially accessible regions were associated with genes involved in cell cycle regulation, meiosis, RNA binding, and cellular metabolism. Transcription factor footprinting identified krüppel-like factor** (**KLF) and specificity protein (SP) family members, POZ/BTB and AT-hook containing zinc finger protein 1 (PATZ1), and the nuclear transcription factor Y (NF-Y) complex (including subunits NFYA, NFYB, and NFYC) as core regulatory factors, with NF-Y binding sites exhibiting marked dynamic changes during development. Further analysis showed that NF-Y target genes are significantly enriched in critical processes such as cell cycle, mitosis, and meiosis, including key regulators *Aurora Kinase A* (*AURKA)* and *AURKB*. Experimental validation confirmed widespread ovarian expression of NFYA, NFYB, and NFYC, with *AURKB* expression strongly correlated with NF-Y protein levels. Dual-luciferase reporter assays demonstrated that NFYA significantly enhances *AURKB* promoter activity through two CCAAT-box elements near the transcription start site. This research depicts the chromatin accessibility dynamics during oogenesis of the Chinese alligator and pinpoints NF-Y as a crucial regulator of *AURKB*, thereby providing a mechanistic foundation for understanding reproduction and advancing conservation in this endangered species.

**Graphical abstract:**

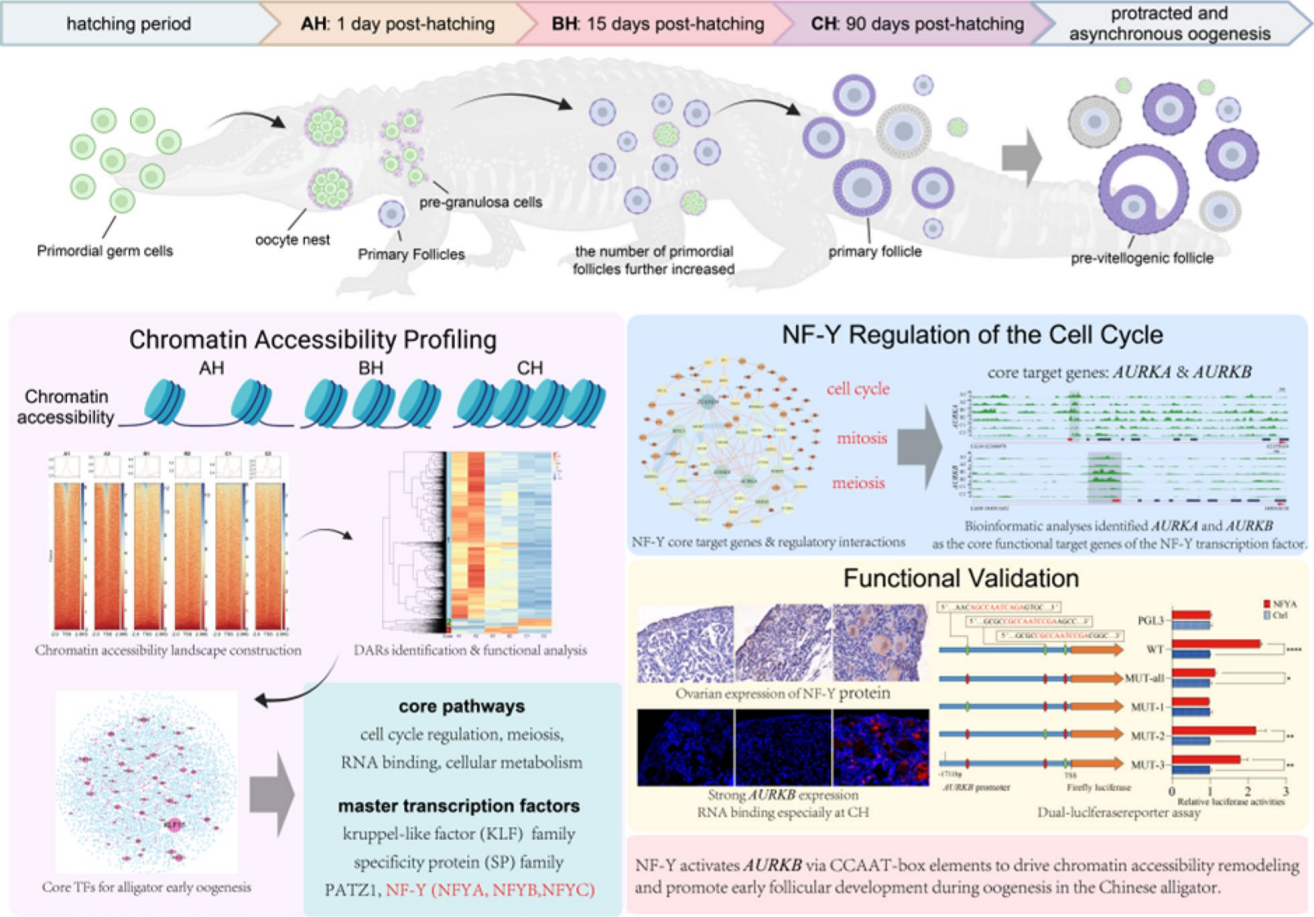

**Supplementary Information:**

The online version contains supplementary material available at 10.1186/s12983-026-00611-8.

## Background

Oogenesis is a multistage and highly coordinated biological process that encompasses oocyte origin and proliferation, meiotic initiation and arrest, and oocyte nest breakdown and primordial follicle (PF) formation [1]. These events are finely regulated by multi-layered mechanisms, including cell cycle control, intercellular communication, and transcriptional and translational regulation [2]. The formation of PF, which occurs within a specific developmental window, results from interactions between oocytes and surrounding granulosa cells. It is widely accepted that in most mammals, the ovary loses the capacity for germ cell renewal after embryogenesis, at which point the number of oocytes within follicles becomes fixed [3]. Pathological developmental abnormalities or depletion of this finite reserve often lead to loss of normal fertility [4]. This transient developmental window and the non-renewable nature of oocytes directly determine ovarian functional capacity in mammals. In contrast, some species of amphibian and reptile such as *Alligator mississippiensis* [5], *Xenopus laevis* [6], *Oryzias latipes* [7], and *Podarcis sicula* [8], exhibit continuous mitotic activity of oogonia and a special pattern of asynchronous oogenesis [9, 10]. The intrinsic mechanisms of limited ovarian reserve pattern in mammals has not been able to fully explain the reproductive biology of the lifelong continuous oogenesis. Understanding these key events and their molecular basis is crucial for providing theoretical foundations for understanding interspecies divergence in reproductive strategies.

Chromatin accessibility, denoting the extent to which particular genomic regions are accessible for binding by transcription factors and other regulatory proteins, represents a crucial epigenetic mechanism that directly modulates gene expression [11]. Dynamic changes in chromatin accessibility during oocyte development are critical for regulating gene expression and determining cell fate [12]. Accumulating evidence indicates significant heterogeneity in chromatin openness across oocyte developmental stages, which is closely associated with developmental potential [13]. For instance, porcine oocytes from large and small follicles exhibit distinct chromatin accessibility profiles, with involved genes enriched in vital processes such as chromatin remodeling, histone modification, and transcriptional regulation [14]. Oogenesis is precisely regulated through multi-layered mechanisms encompassing cell cycle control, intercellular communication, transcriptional and translational regulation, all of which are intricately associated with chromatin architecture. In mice, the transcription factor CCAAT enhancer binding protein beta (C/EBPβ) recruits the histone acetyltransferase p300 to enhance chromatin accessibility at the promoter regions of key genes (e.g., *Furin*) in the neurotrophic receptor tyrosine kinase** (**NTRK) signaling pathway, thereby regulating PF formation and establishing the ovarian reserve [15]. Recent studies have identified significant chromatin accessibility changes during early stage of oogenesis, driven by DNA methylation, histone modifications, and other mechanisms [16]. These changes play a key role in genome-wide reprogramming in female germ cells and the activation or repression of specific genes [17]. Moreover, regulatory sequences located within accessible chromatin regions, including promoters, enhancers, and insulators, engage in interactions with specific transcription factors to initiate transcriptional programs that regulate cell fate determination and development [18]. Consequently, profiling the dynamic alterations in chromatin accessibility through assay for transposase-accessible chromatin with high-throughput sequencing (ATAC-seq) would be helpful to elucidate the core transcriptional programs that govern the prioritized development of oocytes during the asynchronous oogenesis in species typified by crocodilians.

During oocyte development, dynamic changes in chromatin accessibility constitute the core epigenetic basis for the temporal regulation of gene expression [19]. This process is orchestrated by key transcription factors such as splicing factor 1 (SF1) [20], nuclear receptor subfamily 5 group A member 2 (NR5A2) [21], and GATA family members [22], forming a precise regulatory network. On one hand, some factors acting as pioneer factors recruit remodeling complexes to facilitate local chromatin opening and initiate the expression of gene associated with development [23]. On the other hand, established open chromatin states can feedback to enhance transcription factor binding efficiency, forming a bidirectional regulatory circuit [11]. This functional network, facilitated by dynamic chromatin accessibility, guarantees the spatiotemporal accuracy of gene expression throughout oocyte development, thereby laying a robust molecular groundwork for comprehending the mechanisms of germ cell development. The nuclear transcription factor Y (NF-Y), a conserved heterotrimeric transcription factor, plays a central role in oogenesis [24]. It consists of NF-YA, NF-YB, and NF-YC subunits [25]. The NF-YA subunit specifically recognizes the CCAAT box, while NF-YB/NF-YC form a dimeric scaffold via their histone fold domains [26]. Studies show strong chromatin accessibility signals at NF-Y binding sites during early oocyte development, suggesting its potential role as a pioneer factor that promotes target gene activation by recruiting chromatin remodelers and cooperating with other transcription factors [27]. NF-Y directly regulates key cell cycle genes (e.g., *Cyclin B1*, *CDK1*) [28] and apoptosis-related genes (e.g., *Bcl-*2, *Bax*) [29], thereby coordinating cell cycle progression and genomic stability, which provides the molecular basis for folliculogenesis. Therefore, due to its pivotal role in linking sequence-specific recognition with epigenetic regulation, NF-Y is a key factor for understanding the mechanisms of germ cell development.

The Chinese alligator is widely recognized as a “living fossil,” representing the only extant crocodilian species endemic to China. Crocodylians originated in the Late Triassic (~ 240 million years ago), and the ancestors of *A. sinensis* coexisted with dinosaurs, retaining many primitive features of early reptiles. Therefore, this species represents an invaluable model for understanding reptilian evolution and reproductive biology in several respects: (1) its unique evolutionary position as the sister group to birds and one of the closest reptilian relatives to mammals; (2) the distinctive regulatory mechanisms underlying its continuous oogenesis, which may differ from those in other reptiles; and (3) the conservation imperative—as a Critically Endangered (CR) species on the IUCN Red List and a first-class protected animal in China, elucidating its reproductive biology is essential for guiding captive breeding and population recovery.

The investigation of early oogenesis mechanisms in the Chinese alligator holds profound implications for understanding the differences in reproductive physiology between reptiles and mammals, as well as for the conservation of this endangered species. Meanwhile, although chromatin accessibility plays a critical role in early oogenesis, the underlying regulatory mechanisms remain poorly understood. This research aims to: (1) Construct the chromatin accessibility landscape during early oogenesis in the Chinese alligator using the assay for ATAC-seq, revealing its dynamic patterns; (2) Investigate differentially accessible regions (DARs) and perform functional enrichment analysis of associated genes; (3) Map the overall regulatory network of transcription factors and target genes during early oogenesis via transcription factor footprinting analysis; (4) Screen for interactions between key transcription factors and their target genes and explore critical regulatory relationships; (5) Preliminarily validate key regulatory interactions using immunohistochemistry, fluorescence in situ hybridization, and dual-luciferase reporter assays. This study provides important insights into the regulatory roles of key transcription factors during early oogenesis in the Chinese alligator, offering valuable references for elucidating its reproductive mechanisms and a theoretical basis for population restoration and captive breeding.

## Methods

### Sample collection

Experimental samples were provided by Wuhu Dajiang Farming (Wuhu, China), which operates in accordance with the *Wildlife Protection Law of the People’s Republic of China*. Under this legal framework, the artificial breeding of first‑class state‑protected species, such as the Chinese alligator, is explicitly sanctioned for scientific research and conservation purposes. Importantly, all samples used in this study were obtained from captive, artificially propagated individuals, thereby minimizing any impact on wild populations. Sample collection was performed with the assistance of professional staff from the breeding farm, ensuring adherence to animal welfare standards and causing no disturbance to wild populations. All experimental procedures were performed in accordance with animal ethical requirements. The animal protocol was conducted following the Regulation for the Administration of Experimental Animal Licenses (No. 593, Version 2, 2001) issued by the Ministry of Science and Technology of the People’s Republic of China, and all experimental procedures were approved by the Academic Ethics Committee of Anhui Normal University (Project identification number: AHNU-ET2023023; Approval date: March 8, 2023). This study adhered to the Guidelines for the Care and Use of Laboratory Animals to minimize animal stress throughout all experimental phases.

Based on preliminary laboratory results [9], three crucial stages of female Chinese alligator germ cell development were chosen: 1 day post-hatching (AH), 15 days post-hatching (BH), and 90 days post-hatching (CH). The AH stage is distinguished by the existence of oocyte nests that have not yet undergone disassembly. At the BH stage, certain oocytes within the nests develop preferentially and establish associations with pre-granulosa cells (Pre-GCs) outside the nests to form PF. The CH stage is characterized by the recruitment of a portion of PF, which commence further development into primary follicles. The samples at each developmental time point consisted of tissues from two female Chinese alligators.

Gonadal tissues located near the dorsal mesentery were bluntly dissected using ophthalmic forceps and transferred to a sterile petri dish containing 1 × phosphate-buffered Saline (PBS, Beyotime, China) solution. After rinsing with PBS to remove surface blood, the tissues were evenly divided into two portions using sterile ophthalmic scissors: the left gonadal tissue was fixed in a 15 mL centrifuge tube containing 4% paraformaldehyde for subsequent histochemical and in situ hybridization analyses; the right gonadal tissue was flash-frozen in liquid nitrogen for 1 h and then stored at -80 °C for ATAC-seq library construction.

### ATAC-seq sequencing and raw data processing

The ATAC-seq library construction involved four main steps: nuclei isolation via cell lysis, tagmentation reaction and product purification, PCR amplification and size selection of target DNA fragments, and final library quality control. The isolated nuclei were resuspended in a reaction system containing Tn5 transposase and incubated at 37 °C for 30 min to perform tagmentation. The reaction was then terminated, and the tagmented DNA was immediately purified using the QIAGEN MinElute kit (QIAGEN, Germany) to remove enzymatic proteins and reaction buffer components, providing a high-purity DNA template for subsequent PCR amplification. The purified product was then amplified [30] and sequenced on the Illumina NovaSeq™ 6000 platform (Illumina, USA).

Raw sequencing data obtained from the sequencer were filtered based on three criteria: removal of adapter sequences, elimination of reads containing more than 10% unknown nucleotides (N), and discarding of reads where over 50% of the bases were of low quality (*Q*-value ≤ 20). The fastp software [31] was used for quality control analysis of the raw sequencing data, which automatically identified and removed low-quality reads, adapter sequences, and contaminated fragments, resulting in high-quality clean reads for subsequent analysis. The clean reads from each sample were then aligned to the reference genome using Bowtie2 v2 [32] with parameters -X2000 and -m1. Reads aligning to mitochondrial DNA were filtered out.

### Analysis of differentially accessible regions

Due to the asymmetric cutting of DNA double strands by Tn5 transposase (+ 4 bp on the positive strand and -5 bp on the negative strand), the original alignment results were adjusted as follows: the start positions of reads aligned to the positive strand were shifted 4 bp to the right, while those aligned to the negative strand were shifted 5 bp to the left. MACS v2.2.7.1 [33] was used for peak calling. The Dynamic Poisson Distribution was applied based on uniquely mapped reads to calculate the *P*-value of specific regions. Additionally, DiffBind [34] was employed to identify differences in chromatin accessibility between groups. Regions with a log2FoldChange ≥ 1 and an FDR ≤ 0.05 were defined as significantly differentially accessible regions.

Based on the genomic coordinates of the DARs, the ChIPseeker software [35] was used to annotate the genes associated with these DARs and to systematically analyze their distribution across different genomic functional elements, including promoters, 5′ untranslated region (5′UTR), 3′ untranslated region (3′UTR), exons, introns, downstream regions, and intergenic regions. All genes associated with DARs were then mapped to the Gene Ontology (GO) database [36] (http://www.geneontology.org/, accessed May 2024) and the Kyoto Encyclopedia of Genes and Genomes (KEGG) database [37] (https://www.kegg.jp/, accessed May 2024) for functional enrichment and pathway analysis, using a significance threshold of FDR ≤ 0.05.

### Transcription factor footprinting analysis

In this study, the MEME suite [38] (http://meme-suite.org/, accessed June 2024) was used to detect transcription factor binding site motifs, with enrichment criteria set at an *E*-value ≤ 10 and a *P*-value < 0.05. MEME-ChIP [39] was subsequently employed to scan for motifs, and MEME-AME was used for the identification of known motifs. Transcription factors corresponding to the identified known motifs were screened using the NCBI database (https://www.ncbi.nlm.nih.gov/, accessed November 2024). Prediction of transcription factor target genes and analysis of their interactions were performed based on the GTRD v.20.06 database [40].

### Protein–protein interaction analysis and transcription factor prediction

Protein–protein interactions were predicted using the STRING online database [41] (https://cn.string-db.org/, accessed January 2025). The regulatory network of selected transcription factor target genes and the protein–protein interaction network were visualized using Cytoscape v.3.10.2 [42]. Concurrently, ATAC-seq signals were visualized with Integrative Genomics Viewer (IGV.16.2) software [43] to display signal enrichment near transcription start sites (TSS). Putative transcription factor binding sites were subsequently predicted using the JASPAR online tool [44] (https://jaspar.elixir.no/cart/, accessed December 2024), with the relative profile score threshold set to 95%. Visualization of gene structure was performed using GSDS [45] (https://gsds.gao-lab.org/index.php, accessed December 2024).

### Integration of published transcriptomic data

To integrate chromatin accessibility with transcriptional output, published transcriptomic data from female Chinese alligator gonads at the AH, BH, and CH stages were incorporated from Liu et al. [46] (SRA: PRJNA1398788 and PRJNA1398664). In that study, RNA-seq libraries were generated from gonadal tissues collected at the same three developmental stages, and gene expression abundance was quantified as FPKM. Differentially expressed genes were identified using the criteria |log2(fold change)|≥ 2 and *P* < 0.05. Predicted NF-Y target genes from our transcription factor footprinting analysis were matched to the published transcriptomic dataset to examine their stage-specific expression patterns, which were visualized as heatmaps (Supplementary Fig. S4).

### Immunohistochemistry

Tissue sections (6 μm thick) were deparaffinized in xylene and rehydrated through a graded ethanol series. Endogenous peroxidase activity was quenched by incubation with 0.3% hydrogen peroxide in methanol for 30 min at room temperature, followed by three 5-min washes in PBST (containing 0.05% Tween-20, Sangon Biotech, China) buffer. Antigen retrieval was performed using a microwave-heating method by immersing the sections in antigen retrieval solution (E673000, BBI Biotech, China). After natural cooling, the sections were washed again with PBST buffer and blocked in a humid chamber with deionized water for 1 h. Subsequently, the sections were incubated overnight at 4°C with the following primary antibodies: rabbit anti-NFYA (DF3125, Affinity Biosciences, China, 1:200 dilution), rabbit anti-NFYB (bs-12245R, Bioss, China, 1:250 dilution), and rabbit anti-NFYC (bs-19231R, Bioss, China, 1:250 dilution). After washing, the sections were incubated with a horseradish peroxidase (HRP)-conjugated goat anti-rabbit secondary antibody (bs-19231R, Bioss, China, 1:500 dilution) for 1 h at room temperature. Signal visualization was carried out using a DAB substrate kit (AR1027, Sangon Biotech, China) according to the manufacturer's instructions. Cell nuclei were counterstained with hematoxylin. Finally, the sections were dehydrated through an ethanol series, cleared in xylene, and mounted with neutral balsam. Images were acquired using a Nikon upright fluorescence microscope equipped with a digital camera.

### Fluorescence in situ hybridization

Paraffin-embedded tissue sections were subjected to deparaffinization, rehydration, and antigen retrieval. After pretreatment with 20 μg/mL proteinase K (General Biosystems, China) at 40 °C for 30 min, the sections were hybridized with Cy3-labeled oligonucleotide probes (*AURKA*-targeting probe: 5'-GGGCGGTTCTCTTTCCGGGTTCTG-3'; *AURKB*-targeting probe:5'-GGGTTCACGTTCTCCTTGTGGGCC-3') at 40°C overnight. Post-hybridization washes were performed using 2 × saline-sodium citrate buffer (SSC, General Biosystems, China) and 1 × SSC buffers at 40 °C. Cell nuclei were counterstained with 1 μg/mL 4',6-diamidino-2-phenylindole, dihydrochloride **(**DAPI, General Biosystems, China), and the sections were mounted with an anti-fade mounting medium (General Biosystems, China). All images were acquired using a Nikon upright fluorescence microscope.

### Cell culture and plasmid vector construction

HEK293T cells (ATCC® CRL-3216™, USA) were cultured in Dulbecco's Modified Eagle Medium (DMEM, Life Technologies, USA) supplemented with 10% fetal bovine serum (FBS, Thermo Fisher Scientific, USA) and 1% penicillin–streptomycin (Thermo Fisher Scientific, USA) at 37 °C with 5% CO₂. The complete coding sequence (CDS) of the NFYA gene was amplified by PCR and cloned into the NheI/HindIII restriction sites of the pcDNA3.1( +) vector using restriction enzyme-based cloning to construct the NFYA expression plasmid. All constructs were verified by restriction enzyme digestion and full-length sequencing. The *AURKB* promoter region was chemically synthesized and cloned into the MluI/XhoI sites of the pGL3-Basic luciferase reporter vector (Promega, USA) to generate the pGL3-*AURKB*-pro plasmid. Site-directed mutagenesis of the CCAAT boxes was performed using the QuikChange site-directed mutagenesis kit (Agilent, USA) with specific mutation primers. Primer sequences are listed in Supplementary Table S1.

### Dual-luciferase reporter assay

HEK293T cells were seeded in 24-well plates at a density of 2.5 × 10^5^ cells per well and cultured for 24 h under standard conditions (37 °C, 5% CO₂) until reaching 70%–80% confluence. Transfection was performed using Lipofectamine 2000 reagent (Thermo Fisher, USA) with a mixture containing 0.3 μg of the dual-luciferase reporter plasmid, 0.3 μg of either pcDNA/PCDNA-NFYA/*AURKB* plasmid, and 0.05 μg of the pRL-TK internal control plasmid. After 5 h of incubation with the transfection mixture, the medium was replaced with fresh complete medium. At 24 h post-transfection, the cells were harvested and analyzed using the Dual-Luciferase reporter assay system kit (TransGen Biotech, China). Assay reagents included 1 × cell lysis buffer (prepared by 1:4 dilution of 5 × stock with ddH₂O), firefly luciferase reagent (aliquoted and stored at − 80 °C), and Renilla luciferase working solution (freshly prepared at a 50:1 ratio of Stop & Glo Buffer to substrate). After washing with PBS, cells were lysed in 1 × lysis buffer for 5–10 min at room temperature. Cleared lysates were transferred in triplicate to a black 96-well plate (Beyotime, China), and dual luciferase activities were sequentially measured using a Gen5 microplate reader (PerkinElmer, USA). Relative reporter activity was calculated as the firefly to Renilla luminescence ratio, and statistical comparisons were made between wild-type (WT) and mutant (MUT) constructs normalized to the negative control.

## Results

### Construction of the chromatin accessibility landscape during early oogenesis in the Chinese alligator

To verify the morphological staging of samples used for ATAC-seq analysis (AH, BH, CH), we referenced the histological features of the ovaries described in our previous study [10]. Hematoxylin and eosin (HE) staining revealed that at AH (1 day post-hatching), the ovaries contained intact oocyte nests; at BH (15 days post-hatching), some oocytes began associating with pre-granulosa cells to form primordial follicles; and at CH (90 days post-hatching), a subset of primordial follicles had progressed to primary follicles. These characteristics confirm the accurate staging of the samples analyzed by ATAC-seq. The ATAC-seq sequencing was performed using the Illumina NovaSeq™ 6000 platform to construct a chromatin accessibility dataset. All six libraries (AH: A1, A2; BH: B1, B2; CH: C1, C2) were sequenced, generating approximately 141.51 Gb of high-quality data, with an average of 157.23 million raw reads per library. The raw data were then quality-controlled using fastp [31], with the raw data and quality control information detailed in Supplementary Table S2. The base composition after filtering is shown in Supplementary Figure S1, indicating good library quality without significant GC separation. Following ENCODE recommendations (https://www.encodeproject.org/atac-seq/), reads were aligned to the latest Chinese alligator genome [47] using Bowtie 2 [32], with an average of approximately 82.02 million reads per library successfully mapped to the genome (Supplementary Table S3).

**Fig. 1:**
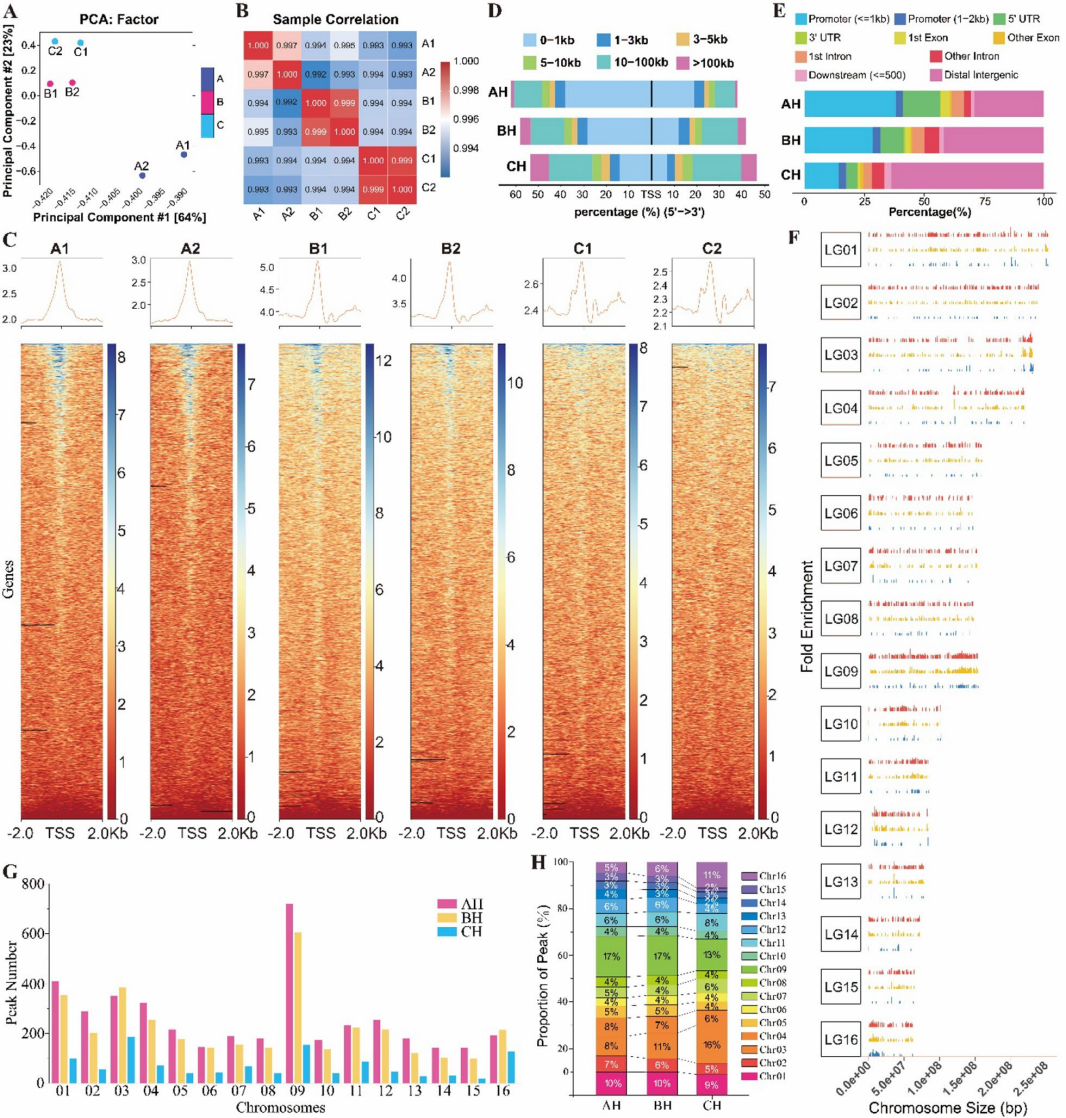
Overview of chromatin accessibility during the early oogenesis of the Chinese alligator. **A**: Principal component analysis. **B**: Sample correlation heat map. **C**: Signal distribution around TSS in each sample. **D**: Distribution of common peak relative to TSS distances. **E**: Distribution of common peak on functional elements of genes. **F**: Distribution of group common peak on chromosomes. **G**: Statistics of the number of common peak on different chromosomes. **H**: Percentage of intragroup common peak on different chromosomes

To further validate the quality of the ATAC-seq libraries, the insert fragment size distribution for each sample was plotted using ATACseqQC [48]. The results (Supplementary Figure S2) showed a clear periodicity of approximately 200 bp in fragment lengths, exhibiting characteristic ATAC-seq fragment distribution patterns. Principal component analysis (PCA) was performed on normalized read abundance values across samples (Fig. 1A). The results demonstrated clear clustering among groups, with significant differences between different developmental stages. Pearson correlation coefficient analysis (Fig. 1B) indicated that the correlations within the same treatment group were all above 0.997, suggesting robust intra-group clustering. Moreover, all correlations between samples were higher than 0.992, which demonstrated favorable reproducibility and high reliability among the experimental samples. Library quality was further assessed by examining read distribution patterns around TSS [49]. DeepTools [50] was used to analyze read distribution characteristics within ± 2 kb regions flanking TSSs. As shown in Fig. 1C, reads were significantly enriched at TSSs with relatively low background signals. These results indicate that the ATAC-seq libraries reliably and reproducibly measure chromatin accessibility in these samples.

Genome-wide identification of open chromatin regions was performed using MACS2 software [33]. Peak information for each sample is summarized in Supplementary Table S4. A total of 49 536 peaks were identified, with an average of 8 256 peaks per sample (range: 3 498–13 672 peaks). Peaks consistently present within each group were selected for subsequent analysis, and the merged results are shown in Supplementary Table S5. This analysis identified 4 209 common peaks in stage A (average length: 455 bp), 3 635 common peaks in stage B (average length: 459 bp), and 1 236 common peaks in stage C (average length: 450 bp).

To investigate the distribution characteristics of open chromatin regions across developmental stages in the Chinese alligator, the R package ChIPseeker [35] was used to analyze the distribution of common peaks across various functional genomic regions. The distribution of common peaks relative to TSS is shown in Fig. 1D. Common peaks were primarily distributed within 0–1 kb of TSS regions, followed by 10–100 kb regions, with most peaks located upstream of TSSs. As development progressed, the number of common peaks in the 0–1 kb region significantly decreased, while the number of common peaks in the 10–100 kb and > 100 kb regions significantly increased.

The distribution of common peaks across genomic functional elements is shown in Fig. 1E (Supplementary Table S6). Peaks were systematically annotated to the following functional regions: promoter regions (< 2 kb upstream of TSS), 5′UTR, exons, introns, 3′UTR, downstream regions (< 500 bp downstream of TSS), and distal intergenic regions (> 2 kb upstream or > 500 bp downstream of TSS). The majority of peaks were located in promoter regions, with percentages gradually decreasing across developmental stages: 41.15% (AH), 31.61% (BH), and 17.31% (CH). The second most abundant category was distal intergenic regions, with percentages progressively increasing: 29.06% (AH), 41.87% (BH), and 63.92% (CH).

ATAC-seq peaks were unevenly distributed throughout the genome, both between and within chromosomes (Fig. 1F). Chromosome 9 contained the highest number of ATAC-seq peaks (Fig. 1G), though its percentage contribution decreased rapidly (17–17–13%) across stages (Fig. 1H). Chromosome 3 showed the second highest peak count, with its percentage gradually increasing (8–11–16%). Chromosome 16 also showed a rapid increase in percentage contribution (5–6–11%). The fewest peaks were distributed on chromosomes 14 and 15. These patterns indicate substantial changes in chromatin openness across these chromosomes during different developmental stages.

### Identification and functional enrichment analysis of differentially accessible regions across early oogenesis stages in the Chinese alligator

**Fig. 2:**
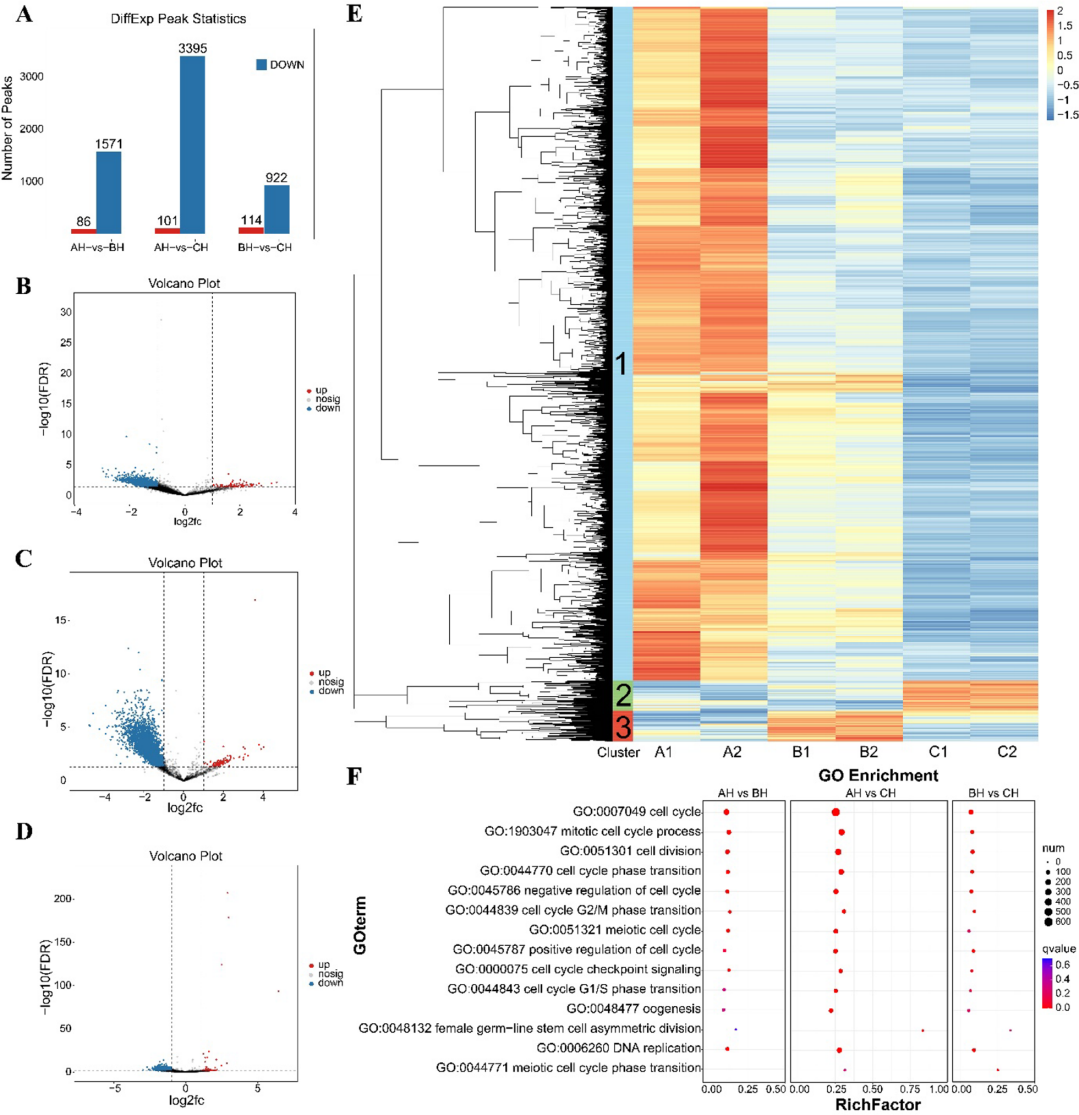
Analysis of differential chromatin accessible regions (DARs). **A**: Summary of DARs. **B**: Volcano plot of DARs in the AH versus BH comparison. **C**: Volcano plot of DARs in the AH versus CH comparison. **D**: Volcano plot of DARs in the BH versus CH comparison. **E**: Clustering heatmap of DAR signals. **F**: Enrichment of cell cycle- and division-related GO terms among DAR-associated genes

To investigate the differentially accessible chromatin regions during early oogenesis in the Chinese alligator, we performed a comparative analysis using DiffBind [34]. The statistical summary of the DARs is shown in Fig. 2A. Volcano plots were constructed to visualize DARs for the AH versus BH (Fig. 2B), AH versus CH (Fig. 2C), and BH versus CH (Fig. 2D) comparison groups. Regions were identified as significant DARs using thresholds of |log2(FC)|≥ 1 and FDR ≤ 0.05 and were annotated to their associated genes. The AH versus BH comparison yielded 1,657 DARs (86 up-regulated, 1,571 down-regulated); the BH versus CH comparison yielded 1,036 DARs (114 up-regulated, 922 down-regulated); and the AH vs CH comparison yielded 3,496 DARs (101 up-regulated, 3,395 down-regulated). Furthermore, a clustering heatmap of the DAR signals was generated (Fig. 2E). The DARs across the three stages segregated into three distinct clusters. Cluster 1, which comprised the largest proportion of DARs, exhibited a progressive decrease in chromatin accessibility across development. In contrast, Clusters 2 and 3 contained relatively few DARs and represented regions with the highest chromatin accessibility during the CH and BH stages, respectively.

Subsequently, the nearest gene to each peak was extracted as the putative target gene, while peaks in distal intergenic regions (located > 2 kb upstream or > 500 bp downstream of genes) were excluded to ensure reliable inference of regulatory relationships. This resulted in 1 177, 2 616, and 720 annotated genes for the AH versus BH, AH versus CH, and BH versus CH comparisons, respectively. The differential peak-associated genes were then mapped to the GO and KEGG databases, with significantly enriched GO terms (Supplementary Table S7) and KEGG metabolic and signal transduction pathways (Supplementary Table S8) identified using the hypergeometric test, considering terms with *P*-value ≤ 0.05 as significantly enriched. The TOP20 enrichment results for GO and KEGG are shown in Supplementary Figure S3, revealing significant enrichment primarily in cellular metabolic process, intracellular membrane-bounded organelle, RNA binding, and related processes.

Subsequently, we visualized the GO terms related to oogenesis and the cell cycle. As shown in Fig. 2F, during early oogenesis in the Chinese alligator, terms such as GO:0051321 meiotic cell cycle and GO:0000075 cell cycle checkpoint signaling were significantly down-regulated. In contrast, terms including GO:0048132 female germ-line stem cell asymmetric division and GO:0044771 meiotic cell cycle phase transition were significantly up-regulated. Notably, despite a lack of significant enrichment in the AH vs BH comparison, the term GO:0048132 (female germ-line stem cell asymmetric division) was significantly enriched in both the AH versus CH and BH versus CH groups.

### Analysis of transcription factors in the chromatin accessibility landscape during early oogenesis in the Chinese alligator

Transcription factor binding sites were identified using transcription factor footprinting [51] and annotated against known transcription factors. First, conserved motifs within peak regions were predicted using the MEME Suite [52]. Subsequently, screening with MEME-CHIP [39] identified 26 significant characteristic motifs (Supplementary Table S9). Among these, 3 were upregulated DARs motifs (1 in AH vs. BH, 2 in AH vs. CH) and 23 were downregulated DARs motifs (8 in AH vs. BH, 8 in AH vs. CH, 7 in BH vs. CH). These unannotated characteristic motif regions may function as regulatory elements involved in oogenesis and developmental regulation in the Chinese alligator [53].

The AME website [54] was then used to detect vertebrate transcription factor (TF) motifs in the DARs from various comparison groups during early oogenesis, with a significance threshold of *P*-value ≤ 0.05 (Supplementary Table S10). Following manual rectification and screening, 171 transcription factor (TF)-motifs were discerned in the comparison group of AH versus BH (1 upregulated and 170 downregulated), 211 TF-motifs in the comparison group of AH versus CH (5 upregulated and 206 downregulated), and 167 TF-motifs in the comparison group of BH versus CH (4 upregulated and 163 downregulated).

**Fig. 3:**
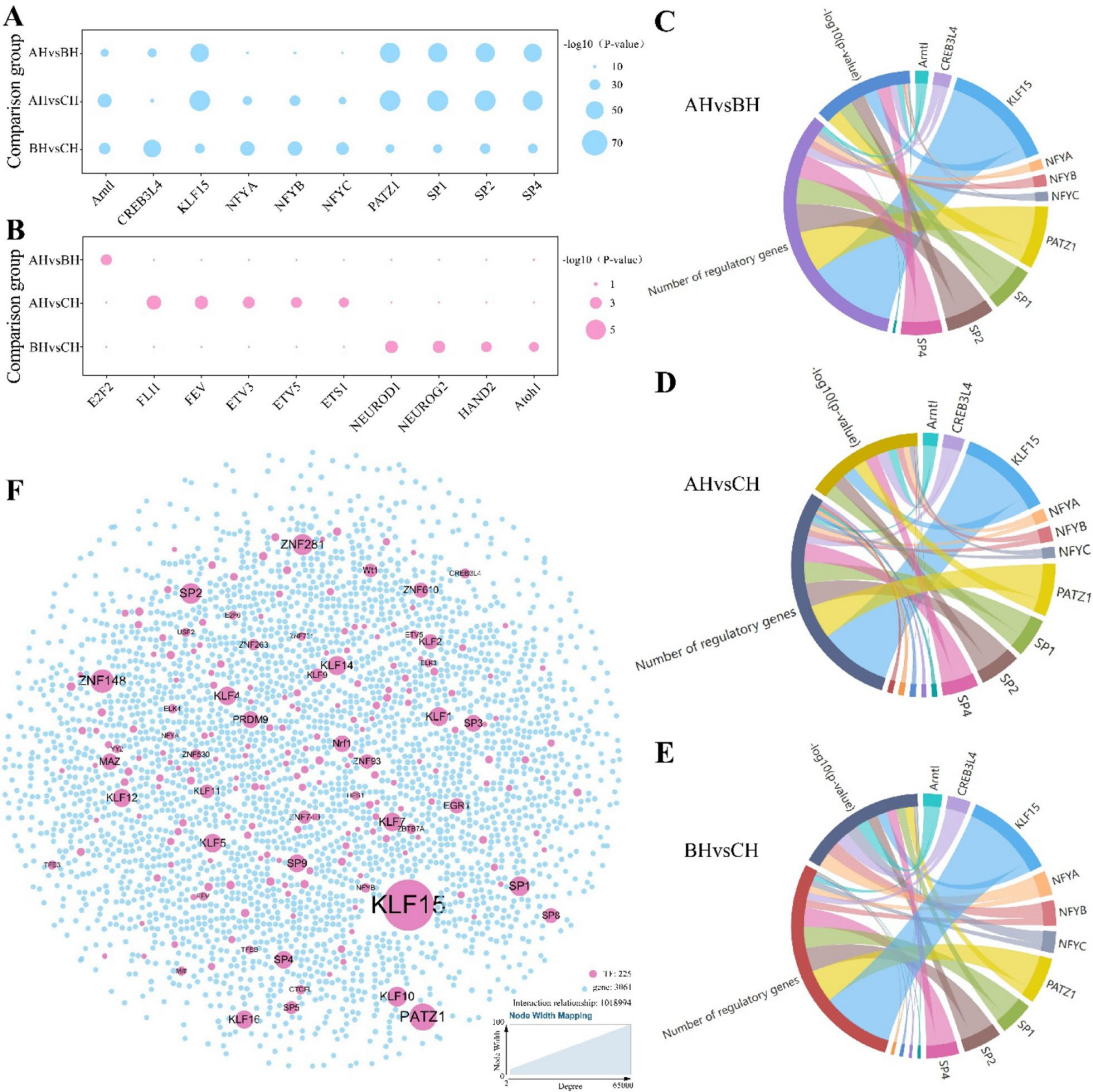
Transcription factor analysis of chromatin accessible regions during early oogenesis in the Chinese alligator. **A**, **B**: Bubble plots showing the enrichment of the top 10 transcription factors in **A** down-regulated and **B** up-regulated differentially accessible regions. **C**–**E**: Chord diagrams displaying the number of target genes regulated by major transcription factors in the **C** AH vs BH, **D** AH vs CH, and **E** BH vs CH comparison groups. **F**: Global regulatory network of transcription factors and target genes. Lines are hidden due to over 1 million regulatory relationships; only key transcription factors are labeled. Node size corresponds to the number of interactions

Based on the known TF-motif enrichment analysis, the enrichment levels of the primary transcription factor binding sites after screening are displayed, with a threshold of *P*-value ≤ 0.05. In the enrichment analysis of transcription factor binding sites within downregulated differentially accessible regions (DARs) (Fig. 3A), the enrichment of binding sites for transcription factors, including krüppel-like factor 15 (KLF15), specificity protein 1 (SP1), SP2, and SP4, gradually declined as development advanced. Conversely, the enrichment of binding sites for the POZ/BTB and AT-hook containing zinc finger protein 1 (PATZ1), NFYA, NFYB, and NFYC increased notably. In the analysis of upregulated DARs (Fig. 3B), the number of significantly enriched transcription factors was relatively fewer, and the enrichment levels were lower.

Based on the predicted target genes of transcription factors from DARs across stages, the number of primary transcription factor target genes was counted for the AH vs BH (Fig. 3C), AH vs CH (Fig. 3D), and BH vs CH (Fig. 3E) comparison groups (Supplementary Table S11). From AH vs BH to BH vs CH, the proportion of genes regulated by KLF15, PATZ1, SP1, SP2, and SP4 decreased, while the proportion regulated by cyclic AMP-responsive element-binding protein 3-like 4 (CREB3L4), NFYA, NFYB, and NFYC increased, although the total number of transcription factor target genes showed a declining trend over time.

To present a comprehensive view of transcription factor target gene regulation, a global regulatory network of transcription factors and target genes was constructed based on prediction results, after removing interactions with unannotated genes (Supplementary Table S12). As shown in Fig. 3F, a total of 225 transcription factors and 3 061 target genes were identified, resulting in 1 018 994 interactions. Key regulatory transcription factors included KLF15, PATZ1, KLF10, zinc finger protein 148 (ZNF148), ZNF281, KLF12, and NFYA. KLF and SP transcription factors, both belonging to the zinc finger gene family, appear to play important roles during early oogenesis in the Chinese alligator.

In conclusion, transcription factors including KLF, SP, PATZ1, NFYA, NFYB, and NFYC demonstrated significant alterations in binding site enrichment during the early stage of oogenesis in the Chinese alligator. These factors displayed high enrichment levels, which were predominantly down-regulated, indicating their potential significance. Notably, the NFYA, NFYB, and NFYC transcription factors collectively form the NF-Y transcription factor complex [55]. From the comparison group of AH vs BH to that of BH vs CH, the enrichment in the analysis of downregulated DARs showed a substantial increase. Specifically, the *P*-value shifted from 3.02e^−9^ (NFYA), 1.10e^−9^ (NFYB), and 3.13e^−8^ (NFYC) to 3.74e^−45^ (NFYA), 1.87e^−44^ (NFYB), and 2.05e^−39^ (NFYC) respectively. Moreover, the proportion of their potential target genes exhibited a significant rise. This rapid change suggests that they may play important roles as oogenesis progresses in the Chinese alligator.

### Screening of NF-Y transcription factor target relationships

**Fig. 4:**
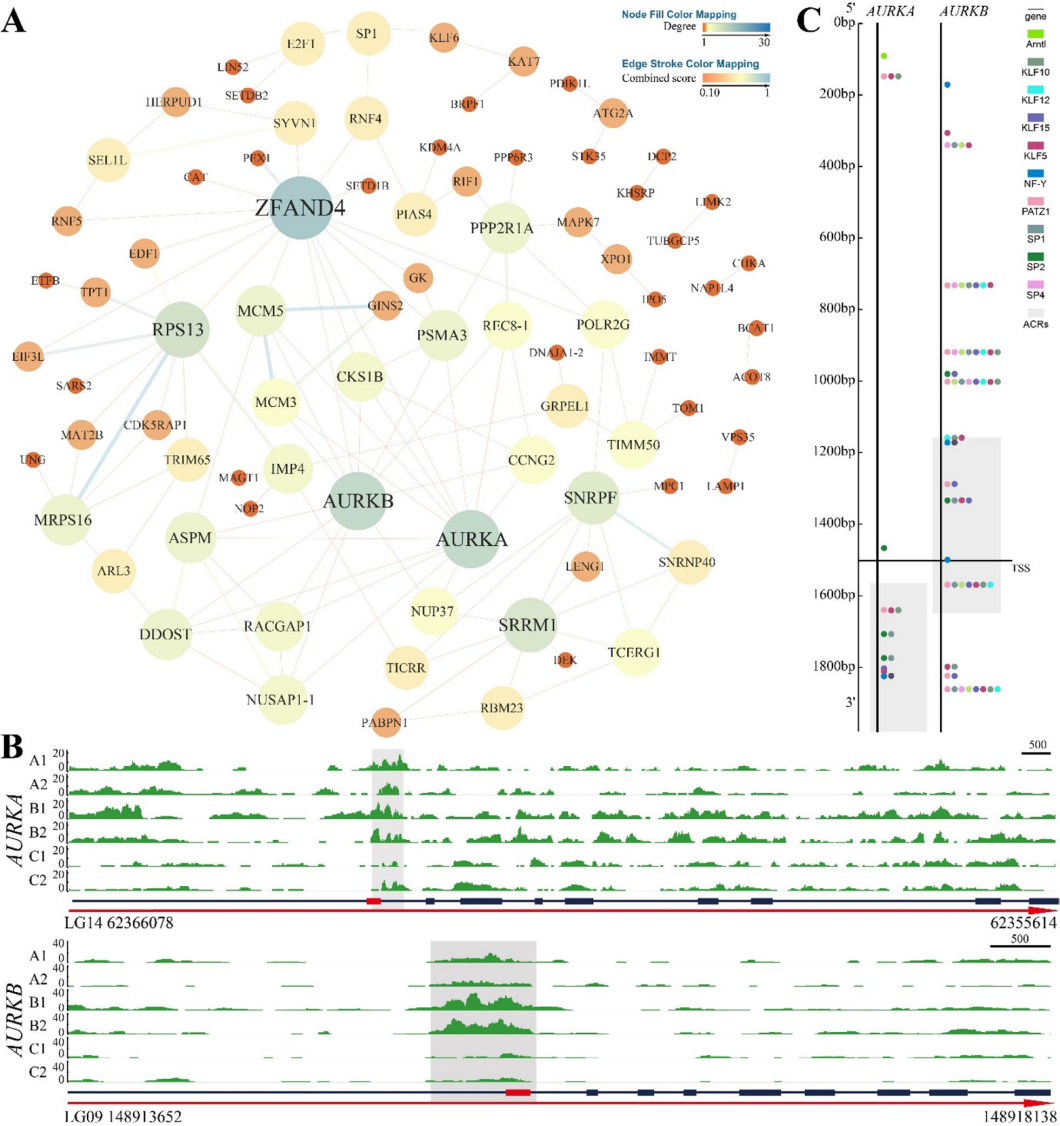
Screening of NF-Y transcription factor targeting relationships. **A**: Protein interaction diagram of target genes regulated by the NF-Y transcription factor in Alligator sinensis. **B**: ATAC-seq signal IGV plot of *AURKA* and *AURKB* genes. **C**: Prediction of transcription factor binding sites in the promoters and differential peak regions of *AURKA* and *AURKB* genes

The NF-Y transcription factor is composed of three protein subunits: NFYA, NFYB, and NFYC [56]. Based on transcription factor footprinting analysis [57], a total of 1 346 regulatory sites were identified for the three protein subunits (Supplementary Table S13). These regulatory sites correspond to 724 genes predicted as direct targets of at least one NF-Y subunit, accounting for 23.6% of all DAR-associated genes (3,063 unique genes across the three comparisons). This proportion indicates that NF-Y binding is broadly distributed among differentially accessible regions during early oogenesis. To investigate the potential regulatory role of the NF-Y transcription factor during early oogenesis in the Chinese alligator, a protein–protein interaction network of NF-Y target genes was constructed using the STRING online database [41] (Supplementary Table S14). As shown in Fig. 4A, four target genes (*ZFAND4*, *AURKB*, *AURKA*, and *RPS13*) encode proteins that exhibited ≥ 10 interactions in the protein–protein interaction network. Another twelve targets, including serine and arginine repetitive matrix 1 (SRRM1), small nuclear ribonucleoprotein polypeptide F (SNRPF), mitochondrial ribosomal protein S16 (MRPS16), dolichyl-diphosphooligosaccharide-protein glycosyltransferase non-catalytic subunit (DDOST), abnormal spindle microtubule assembly (ASPM), minichromosome maintenance complex component 5 (MCM5), proteasome 20S subunit alpha 3 (PSMA3), protein phosphatase 2 scaffold subunit Aalpha (PPP2R1A), Rac GTPase activating protein 1 (RACGAP1), nucleolar and spindle associated protein 1 (NUSAP1-1), CDC28 protein kinase regulatory subunit 1B (CKS1B), and IMP U3 small nucleolar ribonucleoprotein 4 (IMP4), were involved in ≥ 5 interactions. The remaining 67 target proteins had fewer interactions.Functional analysis of proteins with ≥ 5 interactions revealed associations with cell proliferation, cell cycle, mitosis, and meiosis for *AURKB* [58], *AURKA* [59], *DDOST* [60], *ASPM* [61], *MCM5* [62], *PPP2R1* [63], *RACGAP1* [64], *NUSAP1-1* [65], and *CKS1B* [66]. Genes related to ribosomal function included *RPS13* [67], *MRPS16* [68], and *IMP4* [69]. *SRRM1* [70] and *SNRPF* [71] were potentially associated with the regulation of RNA-binding activity. Overall, the downstream genes regulated by the NF-Y transcription factor are involved in cell cycle, mitosis, meiosis, ribosomal function, and RNA activity regulation. Notably, *AURKA* and *AURKB* genes play crucial roles in cell cycle progression and cell division [72].

To further relate chromatin accessibility to transcriptional output, we integrated the published transcriptomic dataset with our predicted NF-Y target genes. Overall, the 724 NF-Y target genes displayed clear stage-specific expression patterns across AH, BH, and CH (Supplementary Figure S4A). Among the differentially expressed NF-Y targets, genes associated with DNA replication and cell-cycle progression, including *CLSPN* [73], *MCM3*, *MCM5*, *MCM7* [74], *CDC25A* [75], *TICRR*, and *E2F1* [76], were elevated from AH to BH. In contrast, regulators associated with mitosis and meiosis, including *AURKA* [77], *AURKB* [78], *CDC20* [79], *MKI67*, *MELK*, *CENPF*, *KIF11*, *NEK2* [80], *ASPM*, *PRC1* [81], *RACGAP1*, and *CCNB2*, showed sustained or increased expression toward CH (Supplementary Figure S4B). In contrast, several stress- and protein-folding-related genes, such as *HSP90B1*, *HSPA5*, *CALR*, and *HERPUD1*, were reduced from BH to CH. These transcriptional patterns occurred alongside the maturation-phase upregulation of meiosis-related genes, such as *STAG3* [82] and *SPO11* [83], and germ cell factors, including *DAZL* [84] and *NOBOX* [85], reported in the published transcriptomic study [46]. Together, these findings suggest that predicted NF-Y target genes undergo stage-dependent transcriptional remodeling during early oogenesis in the Chinese alligator, rather than a uniform linear transition shared by all targets.

The ATAC-seq signals of potential NF-Y transcription factor target genes, *AURKA* and *AURKB*, were visualized using the IGV [43]. As depicted in Fig. 4B, both *AURKA* and *AURKB* displayed significant chromatin accessible regions, featuring high levels of chromatin accessibility during the AH and BH stages. However, the signals declined considerably during the CH stage. Notably, the DARs in *AURKA* was located in the first exon and first intron, downstream of the TSS. In contrast, the majority of DARs in *AURKB* were situated in the promoter region upstream of the TSS, with a small number in the first exon and intron. These DARs may represent transcription factor binding sites [86] involved in regulating *AURKA* and *AURKB* during early oogenesis in the Chinese alligator.

Subsequently, sequences spanning 1 500 bp upstream to 480 bp downstream of the transcription start sites of the Chinese alligator *AURKA* and *AURKB* genes were extracted, covering both the promoter regions and DARs (Supplementary Table S15). Prediction of major transcription factor binding sites during oogenesis was performed using the JASPAR online tool [44] (Supplementary Table S16). As depicted in Fig. 4C, a total of 18 transcription factor binding sites were detected in *AURKA*. Specifically, there was one binding site each for NFYA and NFYC, two binding sites each for KLF10 and KLF15, three binding sites for KLF5, two and three binding sites for SP1 and SP2 respectively, one binding site each for aryl hydrocarbon receptor nuclear translocator-like (Arntl) and Hap5p (HAP5), and two binding sites for PATZ1. Interestingly, the majority of these differential binding sites were located within the 13 DARs downstream of the TSS. In *AURKB*, 83 transcription factor binding sites were identified, including four NFYA and three NFYC binding sites, nine KLF10, eight KLF12, eleven KLF15, and twelve KLF5 binding sites, along with nine SP1, ten SP2, and eight SP4 binding sites, two HAP5 binding sites, and eight PATZ1 binding sites. Among these, 16 binding sites were located within the DARs. In the prediction of transcription factor binding sites in the promoter and DARs of the Chinese alligator *AURKA* and *AURKB* genes, both genes contained NFYA and NFYC binding sites that highly overlapped with binding sites for KLF, SP, and PATZ1 transcription factors, potentially providing favorable conditions for cooperative interactions among these transcription factors.

### Experimental validation of NF-Y transcription factor-mediated regulation of AURKA and AURKB gene expression

**Fig. 5:**
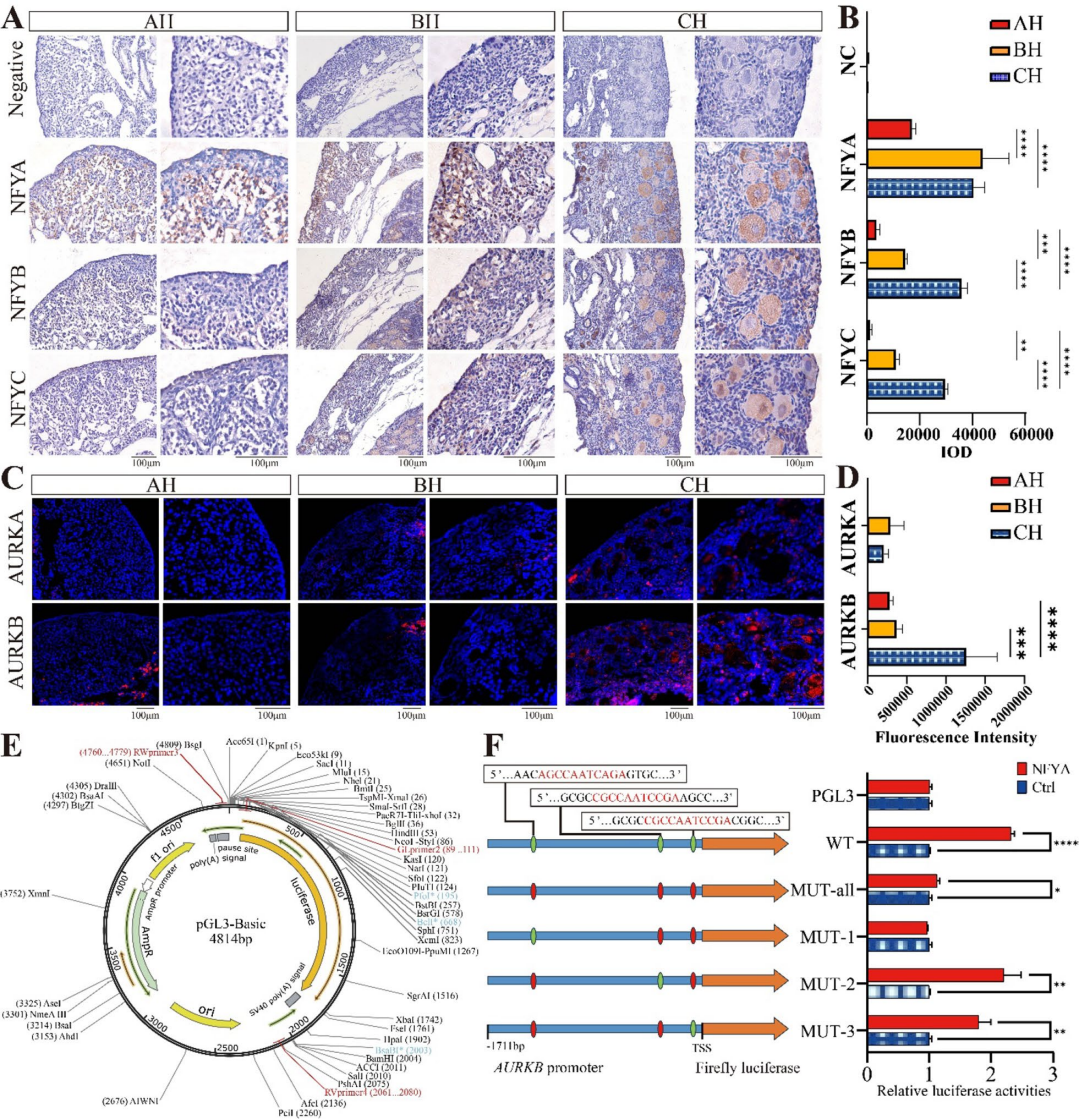
Experimental evidence for NF-Y transcription factor targeting and regulating the expression of *AURKA* and *AURKB* genes. **A**: Immunohistochemical localization of NFYA, NFYB, and NFYC proteins in gonadal tissues across developmental stages (AH, BH, CH). **B**: Semi-quantitative analysis of NF-Y subunit protein expression levels. **C**: Spatial distribution of *AURKA* and *AURKB* transcripts detected by fluorescence in situ hybridization (FISH). **D**: Semi-quantitative analysis of *AURKA* and *AURKB* expression levels based on fluorescence intensity. **E**: Schematic representation of plasmid constructs used for the *AURKB* promoter luciferase assays. **F**: Dual-luciferase reporter assay results validating NFYA binding to the *AURKB* promoter

To validate the regulatory role of the NF-Y transcription factor on *AURKA* and *AURKB* genes, paraffin sections of gonadal tissues from three developmental stages (AH, BH, and CH) were first selected for immunohistochemical (IHC) staining targeting the NF-Y transcription factor protein subunits (NFYA, NFYB, and NFYC). The expression patterns and localization characteristics were analyzed by observing the distribution and intensity of positive signals in cells. The staining results are shown in Fig. 5A, and the IOD semi-quantification results are shown in Fig. 5B (Supplementary Table S17). In the AH female gonads of the Chinese alligator, all three subunits of the NF-Y transcription factor protein were located in gonadal stromal cells, oogonia, epithelial cells, and granulosa cells. In the BH female gonads, the three subunits were expressed in oogonia, PF, granulosa cells, gonadal stromal cells, and epithelial cells. In the CH female gonads, all three subunits were expressed in follicles and their margins, oogonia, granulosa cells, gonadal stromal cells, and epithelial cells.

The three subunits of the NF-Y transcription factor protein were expressed in essentially the same locations; however, there were substantial differences in expression levels among different subunits within the same stage. The NFYA protein subunit showed the highest expression levels across all three stages, followed by NFYB, while NFYC had the lowest expression. Additionally, the expression patterns of different protein subunits varied slightly across stages. The expression level of the NFYA subunit increased significantly from AH to BH but did not change significantly during the CH stage. In contrast, the expression levels of NFYB and NFYC subunits increased significantly from AH to BH and again from BH to CH. The identical localization of the three NF-Y subunits during early oogenesis in the Chinese alligator provides the necessary conditions for their complete biological function, while the differences in expression levels and patterns among the subunits suggest precise regulatory mechanisms tailored to different developmental stages.

Subsequently, fluorescence in situ hybridization (FISH) was performed to detect the expression and localization of *AURKA* and *AURKB* RNA in Chinese alligator gonadal tissues. The fluorescence staining results are shown in Fig. 5C, and the semi-quantitative fluorescence intensity results are shown in Fig. 5D (Supplementary Table S18). During the AH and BH stages, both *AURKA* and *AURKB* RNA expression was localized in the cytoplasm at the junction between the mesonephros and gonadal interstitium, with minimal expression in the cortical region of the female gonads. However, during the CH stage, *AURKA* and *AURKB* RNA showed extensive and strong expression in the interstitial cells, intrafollicular and thecal cells, and granulosa cells of the female Chinese alligator gonads, with high expression levels. Semi-quantitative fluorescence intensity analysis revealed no significant differences in *AURKA* RNA expression across the three stages, potentially due to strong expression in cells at the mesonephros-gonadal junction. In contrast, *AURKB* RNA expression was low during AH and BH stages but increased significantly during the CH stage. In general, the RNA expression levels of *AURKB* were higher than those of *AURKA*. Specifically, weak or undetectable expression of *AURKB* was detected in female gonads during the AH and BH stages, while significant expression was observed during the CH stage. Moreover, there was a high degree of overlap between the expression of *AURKB* and the protein expression of the NF—Y transcription factor at this stage.

Since only the NFYA subunit of the NF-Y transcription factor directly binds to DNA [87], and three NF-Y transcription factor binding sites (i.e., three CCAAT homologous boxes) were predicted in the promoter of its target gene *AURKB*, the NFYA protein was selected for dual-luciferase reporter assays to validate its targeting binding capability to the *AURKB* promoter. Six experimental groups were established: blank control; WT (wild-type); MUT-all (all NFYA binding sites knocked out); MUT-1 (retaining the NFYA binding site farthest from the transcription start site in the promoter region); MUT-2 (retaining the second farthest NFYA binding site); and MUT-3 (retaining the NFYA binding site closest to the transcription start site). The vector maps are shown in Fig. 5E, and the synthesized sequence information together with all plasmid sequencing reports are provided in the Supplementary Information; all matching the expected sequences.

The experimental results are shown in Fig. 5F (Supplementary Table S19). The pGL3 empty vector control performed well, indicating no issues with the reaction system. Firefly luciferase activity increased significantly (*P* ≤ 0.0001) for the wild-type *AURKB* promoter upon NFYA overexpression, demonstrating that NFYA can bind the *AURKB* promoter and enhance its transcription. In the case of MUT-all, which is devoid of all CCAAT box NFYA transcription factor binding sites, the activity of firefly luciferase exhibited a marginal yet non-substantial elevation (statistically significant at *P* ≤ 0.05). This finding implies that factors beyond the NF-Y transcription factor might exert an influence on the transcription of the *AURKB* promoter. In Mut-1, retaining the CCAAT box farthest from the TSS (-1 455 to -1 444 bp), transcriptional activity changes were not significant. Regarding MUT-2 and MUT-3, which respectively retained the middle CCAAT boxes (ranging from − 237 to − 227 bp) and the CCAAT boxes closest to the TSS (ranging from − 143 to − 132 bp), the changes in transcriptional activity were statistically significant (*P* ≤ 0.01). This finding suggests that NFYA exerts a significant positive regulatory influence on *AURKB* in vitro. These in vitro validation experiments demonstrate that the CCAAT box farthest from the TSS does not contribute to NFYA-mediated regulation, while the regulatory effect of NFYA is primarily achieved through the two CCAAT boxes closest to the TSS.

## Discussion

This research integrated ATAC-seq and molecular functional validation to systematically depict the dynamic landscape of chromatin accessibility during the early stage of oogenesis in the endangered reptile, the Chinese alligator, and to clarify the core role of the transcription factor NF-Y in regulating the key cell cycle gene *AURKB* during follicular development. Although the conventional perspective links open chromatin regions with active transcription, an increasing body of evidence emphasizes the highly context-dependent relationship between chromatin characteristics and gene expression regulation [88, 89]. Evaluating the regulatory implications of changes in chromatin accessibility is especially significant during oocyte growth, which coincides with genome-wide epigenetic reprogramming, such as de novo DNA methylation [90–92]. Through the examination of three critical postnatal time points, 1 day (AH), 15 days (BH), and 90 days (CH), we uncovered a substantial global decline in chromatin accessibility from AH to CH. This is in consistent alignment with findings in humans [91], mice [90], rats [92], cattle [93], and geese [94], strongly indicating that the gradual chromatin compaction and "closure" during early oogenesis is a conserved and cross-species epigenetic remodeling characteristic. This global decline is presumably inextricably associated with two synergistic biological processes: firstly, the global heterochromatin reorganization that accompanies cell differentiation [95]; secondly, its close coupling with concurrent DNA methylation reprogramming. In mammals, female primordial germ cells experience DNA methylation erasure [96], followed by the re-establishment of oocyte-specific methylation patterns during folliculogenesis [97], accompanied by a decrease in accessibility near TSSs [90]. The similar trend observed in the Chinese alligator implies that this combined regulatory strategy of chromatin compaction and enhanced DNA methylation may be a shared mechanism among amniotes for genome stabilization and restriction of non-essential transcription. and guiding cell fate during the early stages of oogenesis.

Notably, amidst this generally conserved decreasing trend, significant local heterogeneity was observed. The dynamics of chromatin accessibility varied among chromosomes. For instance, the proportion of accessible regions on Chr03 and Chr16 increased throughout the developmental process. These regions may contain regulatory elements that are crucial for oogenesis in the Chinese alligator, and their distinctively maintained open chromatin state could serve as the basis for key lineage-specific epigenomic disparities [90, 91]. Functional enrichment analysis of differentially accessible regions further disclosed subtle specializations in functional networks between species. Genes that are active during the early oogenesis of the Chinese alligator were predominantly enriched in fundamental processes such as cellular metabolism, RNA processing, protein synthesis, and oxidative phosphorylation. This pattern both concurs with and deviates from the enrichment in cell cycle and meiosis reported in avian studies [94], indicating that within the conserved chromatin dynamic framework, distinct species may precisely adjust their supporting gene networks to coordinately propel oocyte growth, meiotic initiation, and follicle formation.

At the regulatory level, an analysis of cis-regulatory elements within accessible chromatin regions has identified a set of core transcription factors, such as SP1, SP2, KLF10, and KLF15. The expression of these factors significantly declined during development, which is consistent with findings in species like cattle [93]. Nevertheless, NF-Y demonstrated a notably remarkable pattern: the enrichment of its binding sites and the quantity of its target genes displayed an inverse-trend increase from the BH to CH stages, suggesting a distinctive regulatory mode. NF-Y is a highly conserved heterotrimeric transcription factor present in all eukaryotes [98], composed of NFYA, NFYB, and NFYC subunits [99], and it typically binds to the CCAAT box in promoter regions. Although it was initially regarded as a component of the basal transcriptional machinery, in-depth investigations have confirmed it exerts a core regulatory function in specific biological processes, such as cell cycle progression [26], differentiation [100], and stress response [101].

Via the construction of gene regulatory networks, we identified NF-Y target genes, including *AURKB*, *AURKA*, and *ZFAND4*, which are closely linked to the cell cycle and proliferation. Our quantitative analysis revealed that NF‑Y binding sites are widely distributed among DAR‑associated genes across multiple functional categories. Specifically, among the 3,063 DAR‑associated genes, 724 (23.6%) were predicted as direct targets of at least one NF‑Y subunit. These target genes are not limited to cell cycle regulators but also encompass RNA processing, ribosome biogenesis, protein modification, and metabolic processes (Supplementary Table S7, S14). This broad distribution suggests that NF‑Y acts as a pleiotropic regulator during Chinese alligator oogenesis, coordinating multiple cellular processes to collectively support oocyte growth and follicle development. Notably, the strong regulation of *AURKB* (a key cell cycle gene) likely represents one important effector arm of this network, but it is by no means the only one.

Integration with the published transcriptomic landscape further refines our interpretation of NF-Y function during early oogenesis. Liu et al. [46] showed that the AH-to-BH transition is dominated by cell-cycle activation, whereas the BH-to-CH transition is characterized by the induction of meiosis-related genes such as *STAG3* [82] and *SPO11* [83] and germ cell-specific factors such as *DAZL* [84] and *NOBOX* [85]. Consistent with this stage-dependent framework, our integrated analysis of NF-Y target genes revealed early enrichment of replication- and cell-cycle-associated genes, including *CLSPN* [73], *MCM3*, *MCM5*, *MCM7* [74], *CDC25A* [75], *TICRR*, and *E2F1* [76], followed by sustained or increased expression of mitotic regulators such as *AURKA*, *AURKB*, *CDC20* [79], *MKI67*, *KIF11*, *NEK2* [80], *ASPM*, *PRC1* [81], *RACGAP1*, and *CCNB2* toward CH. These patterns suggest that NF-Y regulates a broader cell-cycle/mitotic module rather than a single downstream gene, thereby helping to coordinate proliferative expansion, follicular remodeling, and the transition toward meiotic competence during primary follicle formation. Because the published transcriptomic dataset was generated from whole gonadal tissues, these expression patterns likely reflect coordinated changes in both germ cells and somatic follicular cells.

*AURKB* is the catalytic subunit of the chromosomal passenger complex (CPC), whose core functions include regulating cell-cycle progression, promoting the correction of chromosome-spindle attachment errors, and maintaining spindle assembly checkpoint activity [102]. In mouse and human oocytes, *AURKB* protein is present at very low levels, and these meiotic functions are thought to be performed predominantly by its highly expressed homolog *AURKC* [103, 104]. However, during Chinese alligator oogenesis, our transcriptomic analyses did not detect *AURKC* expression. Instead, we found that *AURKB* mRNA was significantly upregulated at the CH stage, and its expression pattern showed a strong spatial correspondence with the localization of NF-Y subunits in follicles and granulosa cells. In addition, in situ hybridization clearly revealed prominent *AURKB* expression in oocytes of primary follicles. NF-Y regulatory motifs were significantly enriched near chromatin-accessible differential genes during Chinese alligator oogenesis, particularly among genes involved in cell-cycle and meiotic pathways. Together, these findings suggest that NF-Y may influence the timing of oocyte division by activating *AURKB* and other cell-cycle-associated genes. In this context, the early activation of *AURKB* and downstream cell-cycle genes driven by NF-Y may represent a spatiotemporally specific regulatory feature of Chinese alligator oogenesis. Overall, these correlative data support the hypothesis that the NF-Y–AURKB axis contributes to follicular development and oocyte maturation during primary follicle formation, while also indicating that additional functional studies will be required to establish the underlying causal mechanism. 

To directly validate this regulatory relationship, dual-luciferase reporter assays verified that the NFYA subunit of the Chinese alligator significantly augments the transcriptional activity of the *AURKB* promoter through binding to its CCAAT boxes [105]. Nevertheless, in vivo ATAC-seq data uncovered an interesting paradox: despite the evident activating potential of NFYA in vitro and the elevated *AURKB* mRNA expression in vivo, the chromatin accessibility of the *AURKB* promoter region containing NF-Y binding sites declined during development. This contradiction underscores the complexity of NF-Y's in vivo function, which mainly stems from its cooperative and dualistic characteristics [106]. Firstly, NF-Y frequently serves as a core scaffold within transcriptional complexes, collaborating with adjacent transcription factors such as SP1 [26], KLF [107], and E2F [108]. Our discovery of SP and KLF binding motifs that highly coincide with NF-Y sites on the *AURKB* promoter offers a structural foundation for this. Secondly, NF-Y can function as either an activator or a repressor, highly dependent on NF-Y's activity is contingent upon its interacting co-factors (e.g., the histone acetyltransferase p300 or the tumor suppressor p53) [109–111]. Thus, in the in-vivo context, although NF-Y harbors intrinsic activating potential, its integration into specific complexes with particular co-factors or combinations of transcription factors probably gives rise to the observed phenomenon where regional chromatin accessibility declines, yet transcriptional efficiency is augmented. This intricate regulatory mechanism guarantees the precise and high-fidelity expression of crucial cell cycle genes within specific developmental time frames.

An important question raised in the Introduction is whether the chromatin-regulatory logic underlying oogenesis is conserved between reptiles and mammals or has diverged with their contrasting reproductive strategies. First, the progressive decline in chromatin accessibility from AH to CH is consistent with single-cell multi-omics studies in mouse and human oocytes [90, 91], supporting the view that large-scale chromatin remodeling is a conserved feature of female germ-cell development rather than a reptile-specific phenomenon. Second, the NF-Y-centered network identified here also fits a conserved promoter-centered regulatory logic. In mammals, NF-Y helps maintain promoter accessibility and nucleosome-depleted regions and regulates many cell-cycle-associated genes [27, 108, 112, 113]; accordingly, the enrichment of NF-Y motifs in promoter-proximal DARs and the transactivation of *AURKB* by NFYA in the Chinese alligator are most parsimoniously interpreted as deployment of a conserved regulatory module. Third, the *AURKB* result suggests species-specific redeployment of this conserved circuitry. In mammalian oocytes, CPC function is shared between *AURKB* and the germ-cell-enriched homolog *AURKC*, with *AURKC* making especially prominent contributions in human oocytes and in specific meiotic processes in mice, whereas in the Chinese alligator we did not detect *AURKC* expression and instead observed CH-stage upregulation and primary-follicle localization of *AURKB*. We therefore speculate that the Chinese alligator may rely more heavily on an NF-Y–AURKB-linked cell-cycle module during follicular progression, representing a spatiotemporally specific deployment of a conserved Aurora/CPC regulatory framework rather than a reptile-specific class of regulators.

We acknowledge several limitations in this study. The small sample size (n = 2 per stage) limits statistical power and may increase the risk of false positives or false negatives. This constraint arose from our initial design of three biological replicates per stage, the exclusion of one sample identified as male to ensure data reliability and consistency, and the necessity of maintaining genetic homogeneity by using individuals from the same clutch. Given that a typical alligator clutch contains approximately 20 eggs, with at least half required for population maintenance, the number of individuals available for research is inherently limited. Nonetheless, technical reproducibility was high (Pearson R > 0.997, Fig. 1B), and the core NF‑Y‑AURKB axis was independently validated by orthogonal methods (immunohistochemistry, fluorescence in situ hybridization, and dual‑luciferase assays). Furthermore, although we provide RNA‑seq data from independent samples [46], the lack of sample‑matched multi‑omics integration precludes direct individual‑level correlation between chromatin accessibility and transcriptional output. Future studies integrating ATAC‑seq and RNA‑seq from the same individuals will be essential to more precisely quantify these regulatory relationships.

The identification of the NF-Y-AURKB axis as a critical regulator of follicular development offers a foundation for monitoring reproductive success in captive alligator populations. The most immediate translational value of this finding lies not in direct pathway intervention, but rather in its potential as a conservation-oriented monitoring tool. In our study, *AURKB* expression was low at AH and BH but markedly elevated at CH, with substantial spatial overlap with NF Y subunits in developing follicular compartments, suggesting that this axis may serve as a candidate molecular indicator of ovarian developmental readiness. Moreover, given the documented sensitivity of crocodilians to endocrine disruption [114, 115], this developmentally regulated module may also serve as a sentinel of ovarian health in captive or environmentally exposed populations. More broadly, integrating such molecular staging information with genome informed breeding strategies [47] could enhance the precision of conservation breeding programs by adding a female specific physiological dimension to reproductive management.

In conclusion, this study systematically delineates the chromatin dynamic landscape during the early stage of oogenesis in the Chinese alligator and offers an in-depth examination of the potential central role of the NF-Y transcription factor in follicular development through the regulation of target genes such as *AURKB* [116]. These findings not only concur with the conclusions from human and mouse studies regarding NF-Y as a key oocyte transcription factor but also imply findings present potential theoretical targets for enhancing the efficiency of artificial breeding in this endangered species. Future research ought to further validate the cooperative regulatory mechanisms of NF-Y in vivo and elucidate the contribution of epigenetic modifications such as DNA methylation to comprehensively uncover the distinctive operation of this conserved regulatory network in reptilian reproductive biology.

## Conclusion

This study revealed the dynamic changes in chromatin accessibility during early oogenesis in the Chinese alligator using ATAC-seq technology, and found that the NF-Y transcription factor plays a key role in regulating the expression of the *AURKB* gene. NF-Y significantly enhances the promoter activity of *AURKB* through the CCAAT box binding site, participating in the dynamic changes of the cell cycle during the asynchronous oogenesis. The above mechanism may provide an epigenetic regulatory explanation for the asynchronous oogenesis pattern of Chinese alligator. In summary, this research elucidates the dynamic features of chromatin accessibility during oogenesis in the Chinese alligator and defines the critical regulatory role of NF-Y in targeting *AURKB*, providing a theoretical foundation for understanding the reproductive mechanisms and informing conservation breeding strategies for this endangered species.

## Supplementary Information

Additional file1 (PDF 94 KB): AURKB MUT-1 promoter in pGL3-Basic.

Additional file2 (PDF 150 KB): AURKB MUT-1 promoter Sequencing Report.

Additional file3 (PDF 182 KB): AURKB MUT-1 promoter Target Gene Sequence.

Additional file4 (PDF 95 KB): AURKB MUT-2 promoter in pGL3-Basic.

Additional file5 (PDF 147 KB): AURKB MUT-2 promoter Sequencing Report.

Additional file6 (PDF 182 KB): AURKB MUT-2 promoter Target Gene Sequence.

Additional file7 (PDF 94 KB): AURKB MUT-3 promoter in pGL3-Basic.

Additional file8 (PDF 149 KB): AURKB MUT-3 promoter Sequencing Report.

Additional file9 (PDF 182 KB): AURKB MUT-3 promoter Target Gene Sequence.

Additional file10 (PDF 1043 KB): AURKB MUT-all promoter in pGL3-Basic.

Additional file11 (PDF 137 KB): AURKB MUT-all promoter Sequencing Report.

Additional file12 (PDF 182 KB): AURKB MUT-all promoter Target Gene Sequence.

Additional file13 (PDF 94 KB): AURKB WT promoter in pGL3-Basic.

Additional file14 (PDF 145 KB): AURKB WT promoter Sequencing Report.

Additional file15 (PDF 182 KB): AURKB WT promoter Target Gene Sequence.

Additional file16 (PDF 97 KB): NFYA in pcDNA3.1(+).

Additional file17 (PDF 101 KB): NFYA Sequencing Report.

Additional file18 (PDF 175 KB): NFYA Target Gene Sequence.

Additional file19 (XLSX 2760 KB): Table S1. Dual-luciferase reporter assay and primers used in its preparation.

Additional file20 (XLSX 54 KB): Table S10. Statistically Significant TF-motifs.

Additional file21 (XLSX 11 KB): Table S11. Statistics of Target Gene Numbers Regulated by Major Transcription Factors During Oogenesis in the Chinese Alligator.

Additional file22 (XLSX 21265 KB): Table S12. Total Regulatory Network of Transcription Factor Target Genes during Early Oogenesis in the Chinese alligator.

Additional file23 (XLSX 185 KB): Table S13. NF-Y Transcription Factor Regulatory Targets During Oogenesis in the Chinese Alligator, Based on Transcription.

Additional file24 (XLSX 18 KB): Table S14. Protein Interaction Network of NF-Y Transcription Factor Target Genes.

Additional file25 (XLSX 12 KB): Table S15. Promoter-TSS Sequence Features of AURKA/AURKB in the Chinese Alligator.

Additional file26 (XLSX 14 KB): Table S16. Prediction of Transcription Factor Binding Sites in Promoter-TSS Sequence of AURKA and AURKB Genes in the Chinese Alligator.

Additional file27 (XLSX 13 KB): Table S17. Semiquantitative Profiling of NF-Y Complex Expression During Early Oogenesis in the Chinese Alligator.

Additional file28 (XLSX 12 KB): Table S18. Semiquantitative Profiling of AURKA and AURKB Expression Dynamics in Early Oogenesis in the Chinese Alligator.

Additional file29 (XLSX 2761 KB): Table S19. Dual-Luciferase Reporter Assay Results.

Additional file30 (XLSX 11 KB): Table S2. Statistics of Base Information Before and After ATAC-seq Filtering.

Additional file31 (XLSX 10 KB): Table S3. Statistics of Gene Reference Genomes for Sample Comparisons.

Additional file32 (XLSX 10 KB): Table S4. Statistical Table of Peak Numbers for Each Sample.

Additional file33 (XLSX 10 KB): Table S5. Statistical Table of Peak Numbers in Each Group.

Additional file34 (XLSX 10 KB): Table S6. Distribution of Common Peaks Across Functional Genomic Elements.

Additional file35 (XLSX 328 KB): Table S7. GO Enrichment Analysis of Genes Associated with DARs.

Additional file36 (XLSX 19 KB): Table S8. Pathway Enrichment Analysis of Genes Associated with DARs.

Additional file37 (XLSX 200 KB): Table S9. MEME-predicted Motif Enrichment Analysis.

Additional file38 (TXT 9 KB): Datasets Sequence of the Inserted Fragment for the Dual-Luciferase Reporter Assay.

Additional file39 (DOCX 187 KB): Figure S1. The base curves are similar to each other, with GC content ranging from 46.12% to 53.34%. The differences are not significant, and there is no obvious GC separation phenomenon.

Additional file40 (DOCX 765 KB): Figure S2. The signal is strongest for short fragments (less than 100bp in length), which correspond to DNA fragments bound by histones in open chromatin regions. As the fragment length increases, the signal gradually weakens, and these longer fragments originate from DNA regions with histone binding.

Additional file41 (DOCX 339 KB): Figure S3. On the left are the top 20 GO term circle plots of the DARs enriched in the comparisons of AH vs. BH (A), AH vs. CH (C), and BH vs. CH (E). The outermost circle shows the top 20 GO terms, with a gene count scale outside the circle. Different colors indicate distinct Ontologies. The second circle displays the background gene count and Q-value for each GO term. The third circle shows the number of genes enriched in each GO term. The fourth circle presents the RichFactor values of the GO terms. On the right are the top 20 KEGG pathway enrichment plots of the DARs in AH vs. BH (B), AH vs. CH (D), and BH vs. CH (F).

Additional file42 (DOCX 403 KB): Figure S4. (A) Expression levels of all 724 NF-Y target genes identified by transcription factor footprinting analysis. (B) Expression levels of differentially expressed NF-Y target genes.

## Data Availability

Supplementary information includes additional analytical and experimental details, with the relevant sequencing data currently being submitted to a public repository.
