# Supplementary figures and images for: Dynamic changes in chromatin accessibility reveal the role of NF-Y targeting AURKB in mediating cell cycle during asynchronous oogenesis in the Chinese Alligator (Alligator sinensis)
Source: Front Zool. 2026 Apr 29;23:24. doi: 10.1186/s12983-026-00611-8 (PMC13274144; doi:10.1186/s12983-026-00611-8)

Project: Untitled.sqd Contig 1

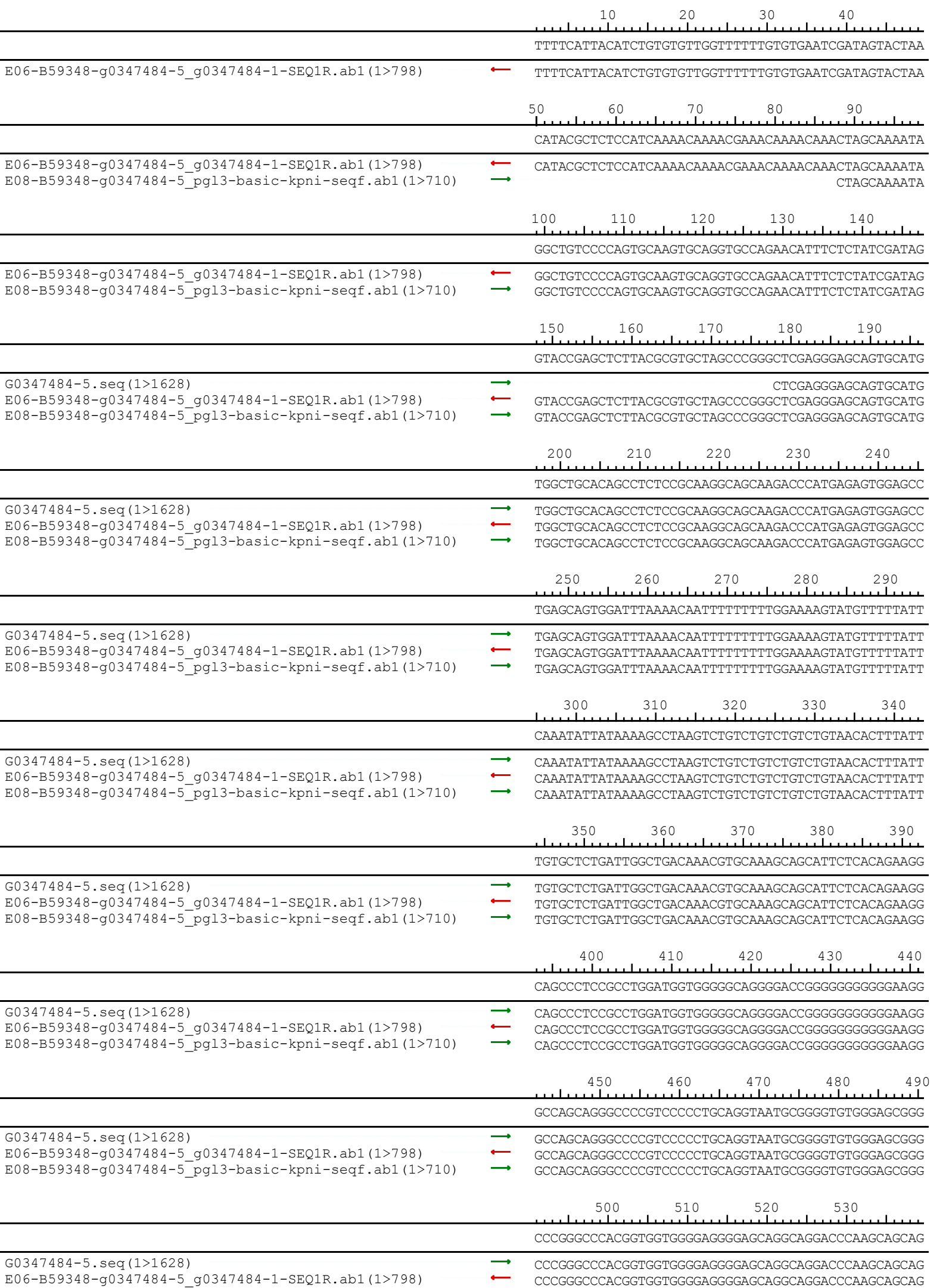

Project: Untitled.sqd Contig 1

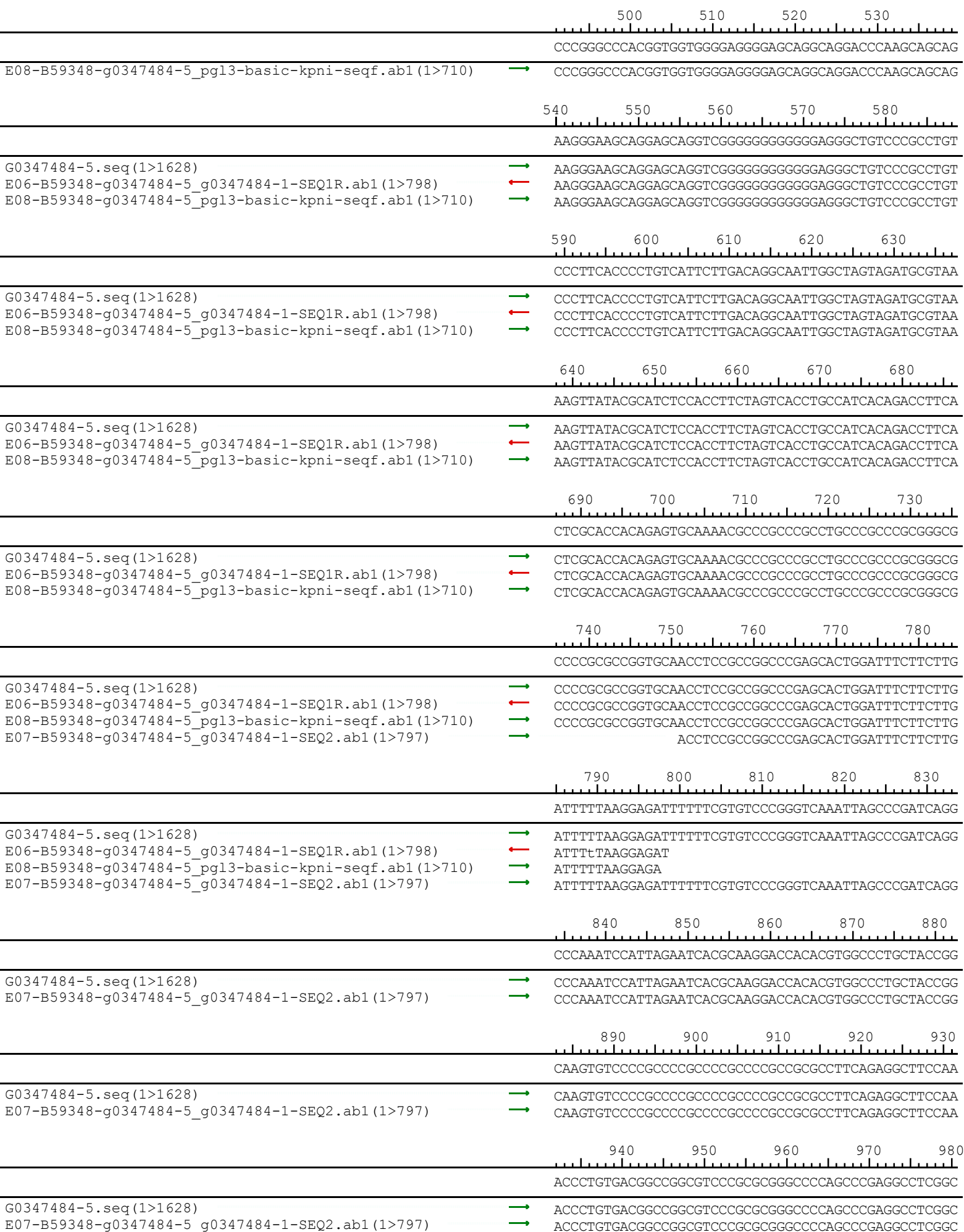

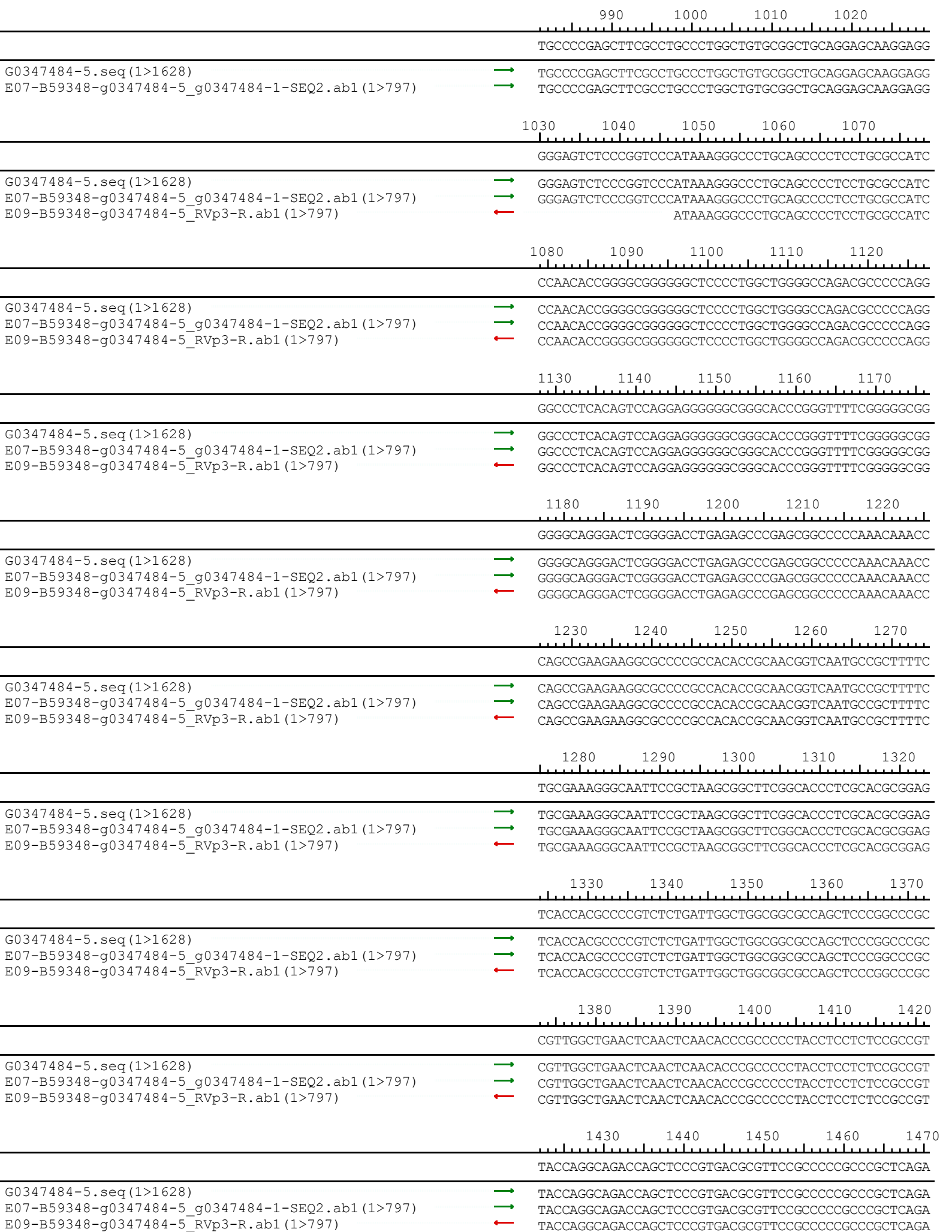

Project: Untitled.sqd Contig 1

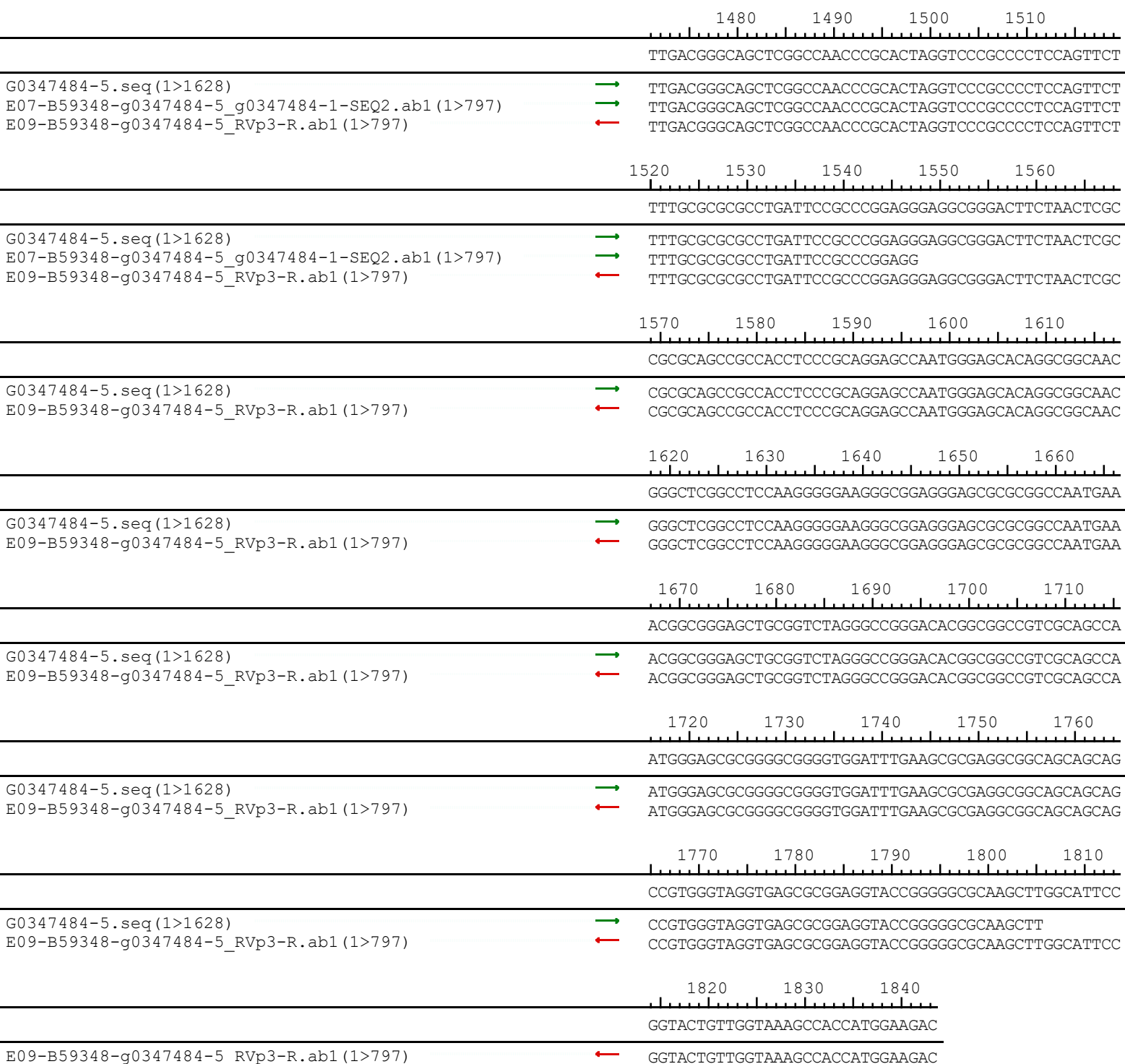

Supplement: Supplementary file 2 — Additional file2 (PDF 150 KB): AURKB MUT-1 promoter Sequencing Report. [file 12983_2026_611_MOESM2_ESM.pdf]

Project: Untitled.sqd Contig 1

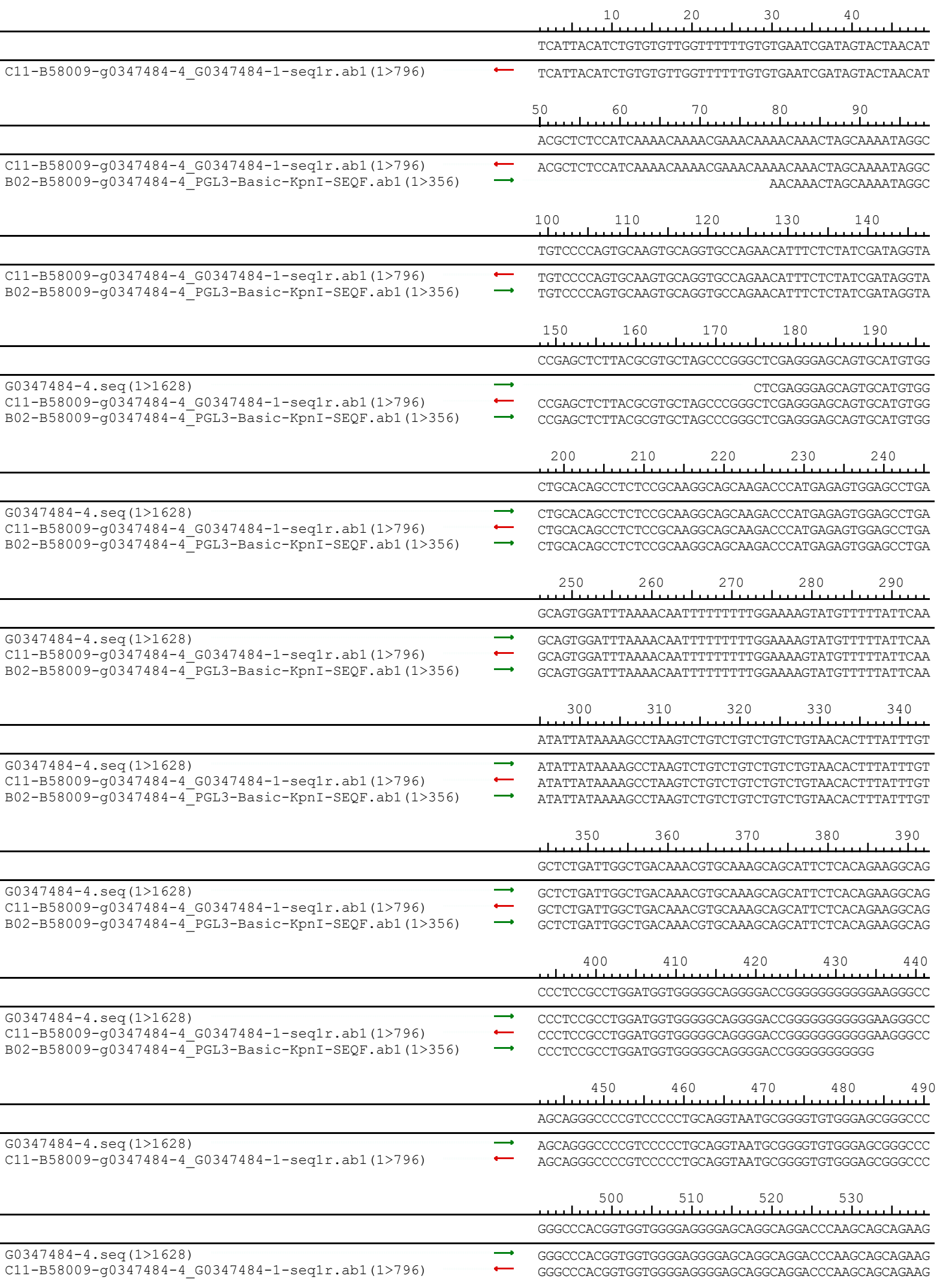

Project: Untitled.sqd Contig 1

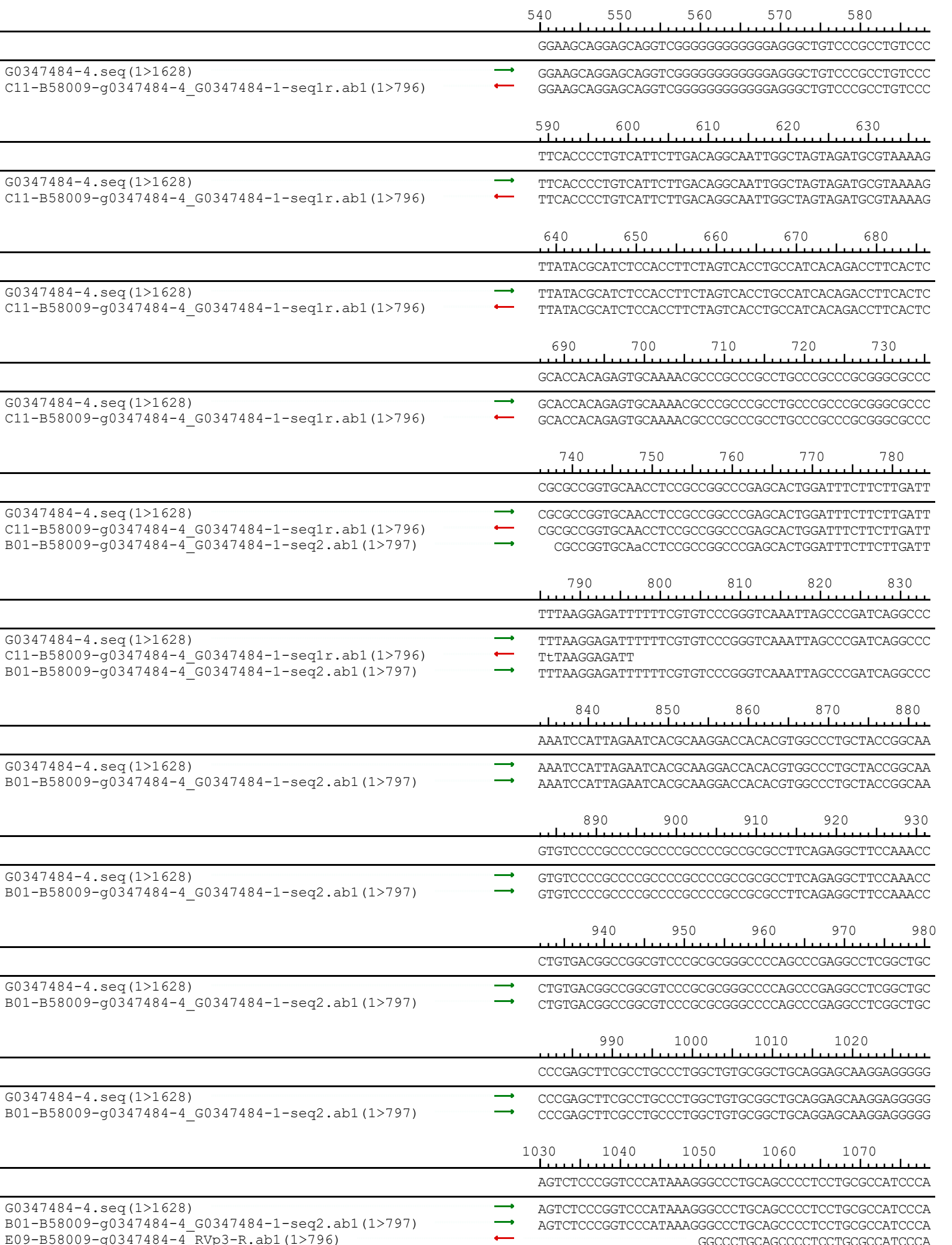

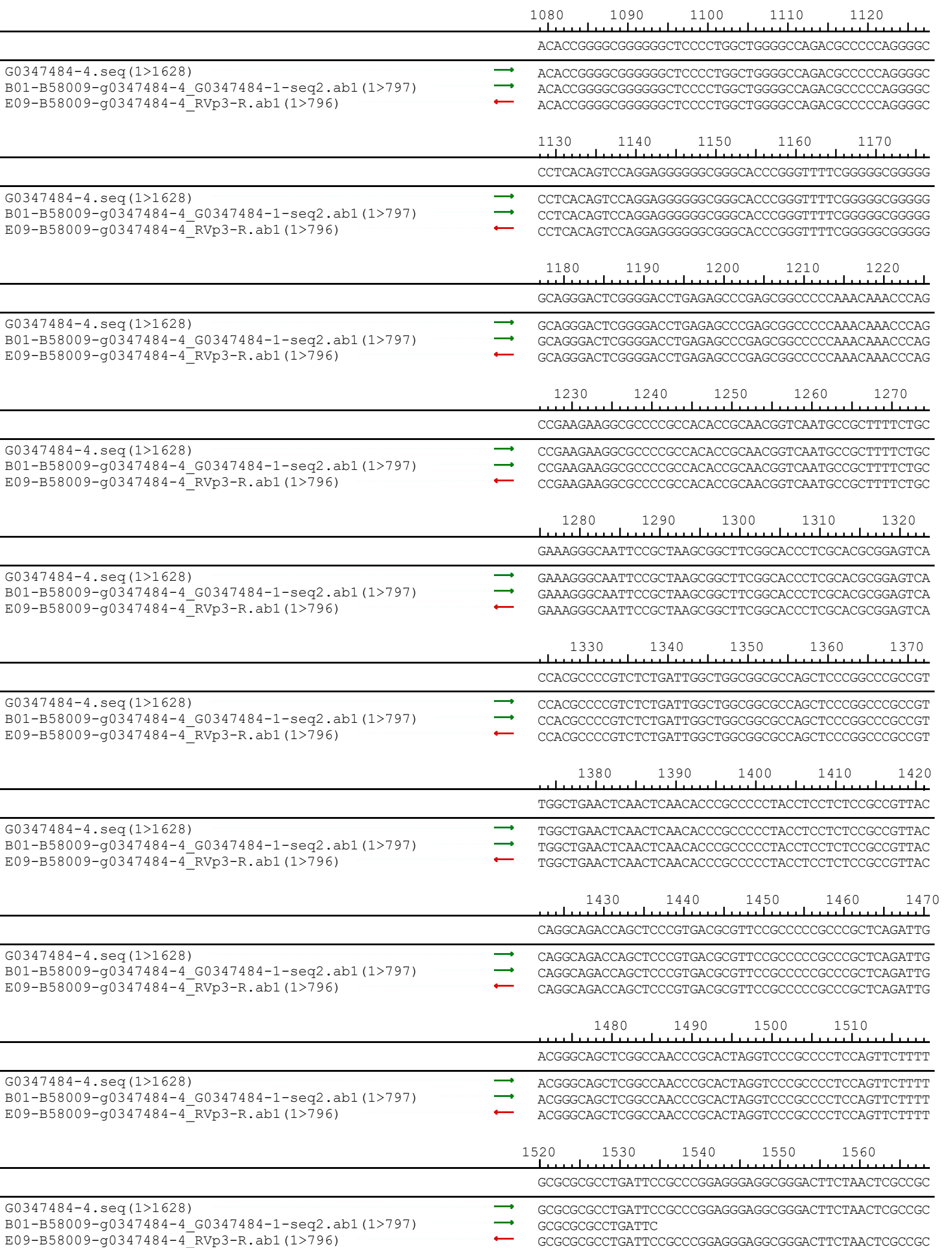

Project: Untitled.sqd Contig 1

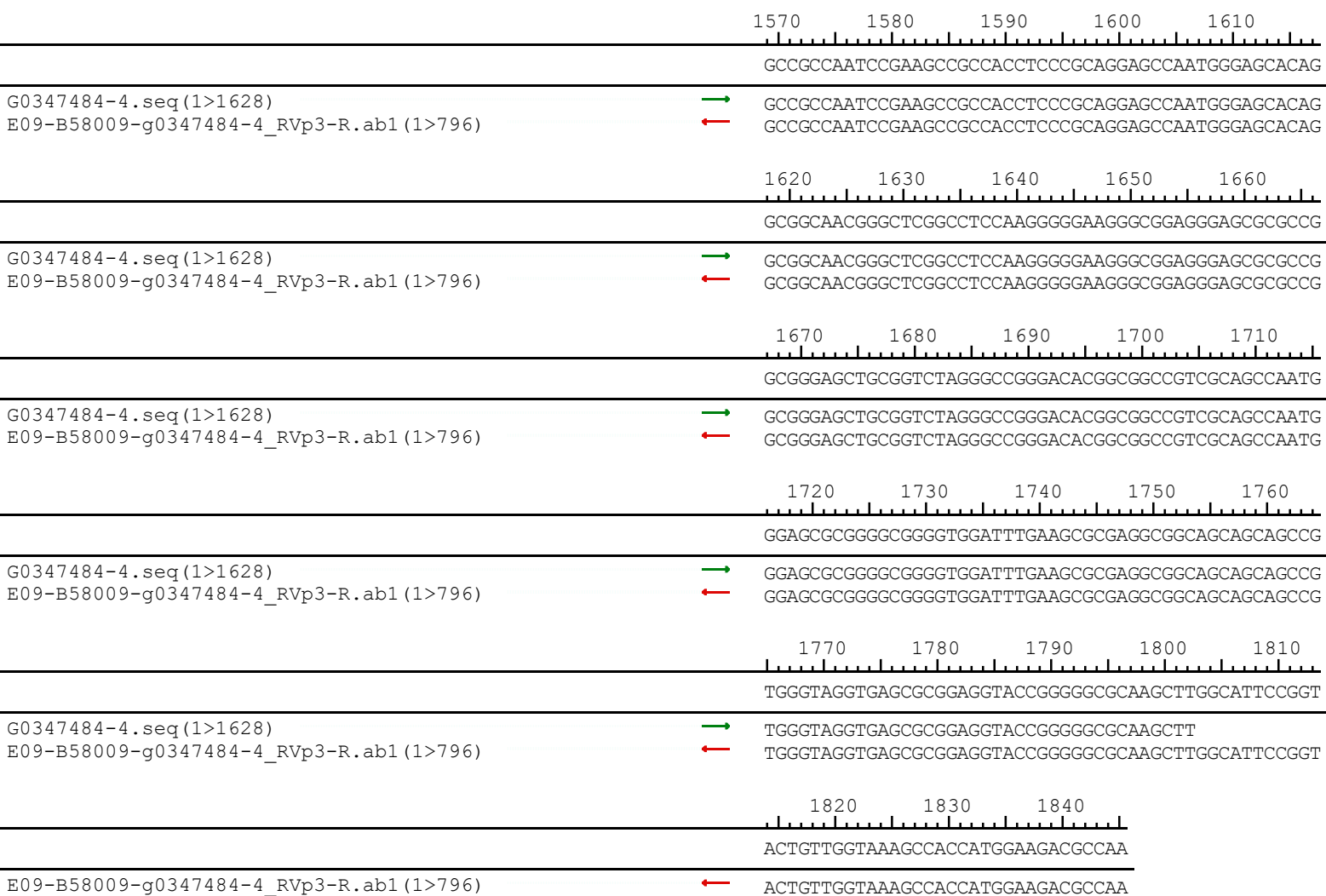

Supplement: Supplementary file 5 — Additional file5 (PDF 147 KB): AURKB MUT-2 promoter Sequencing Report. [file 12983_2026_611_MOESM5_ESM.pdf]

Project: Untitled.sqd Contig 1

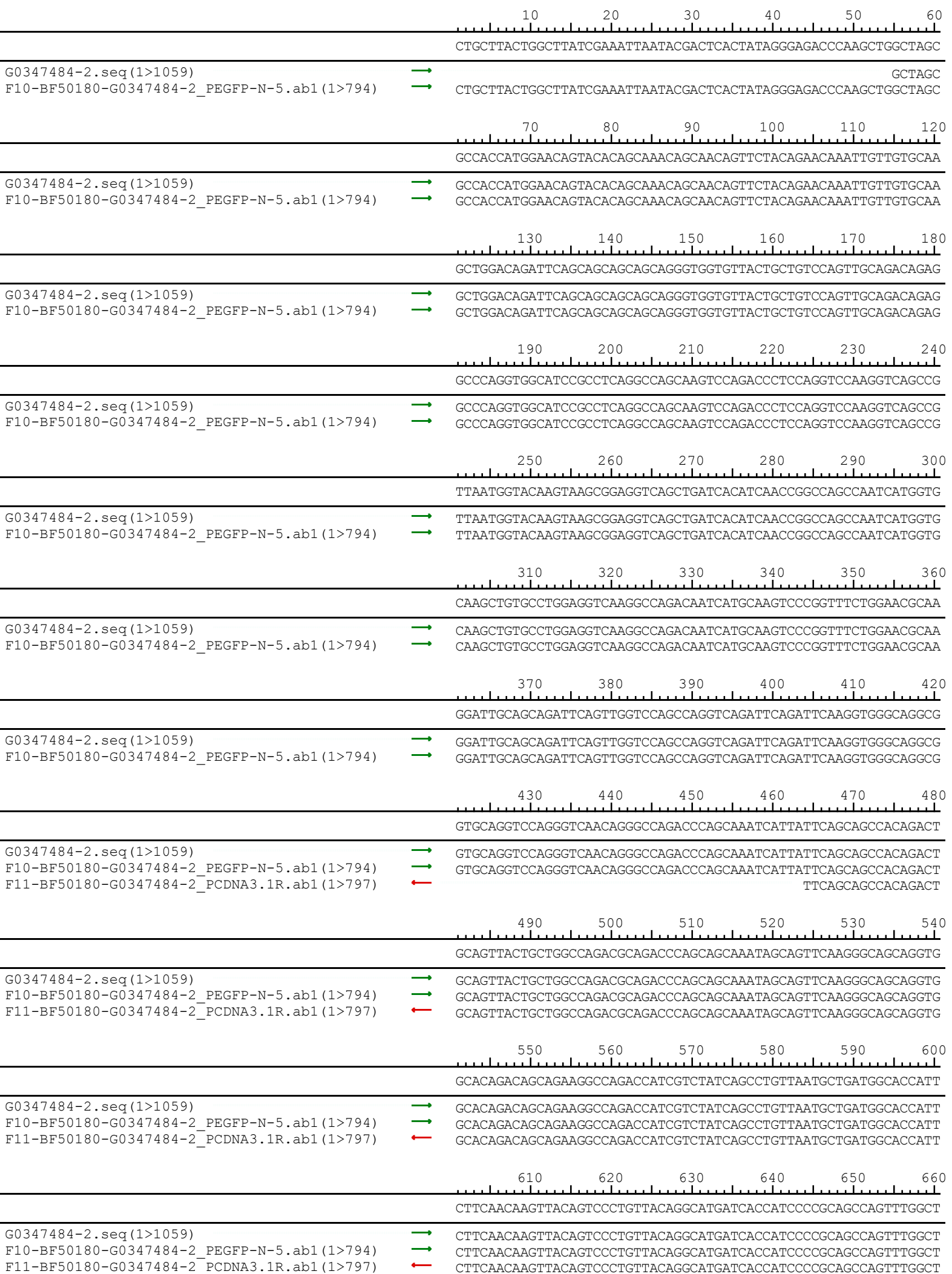

Project: Untitled.sqd Contig 1

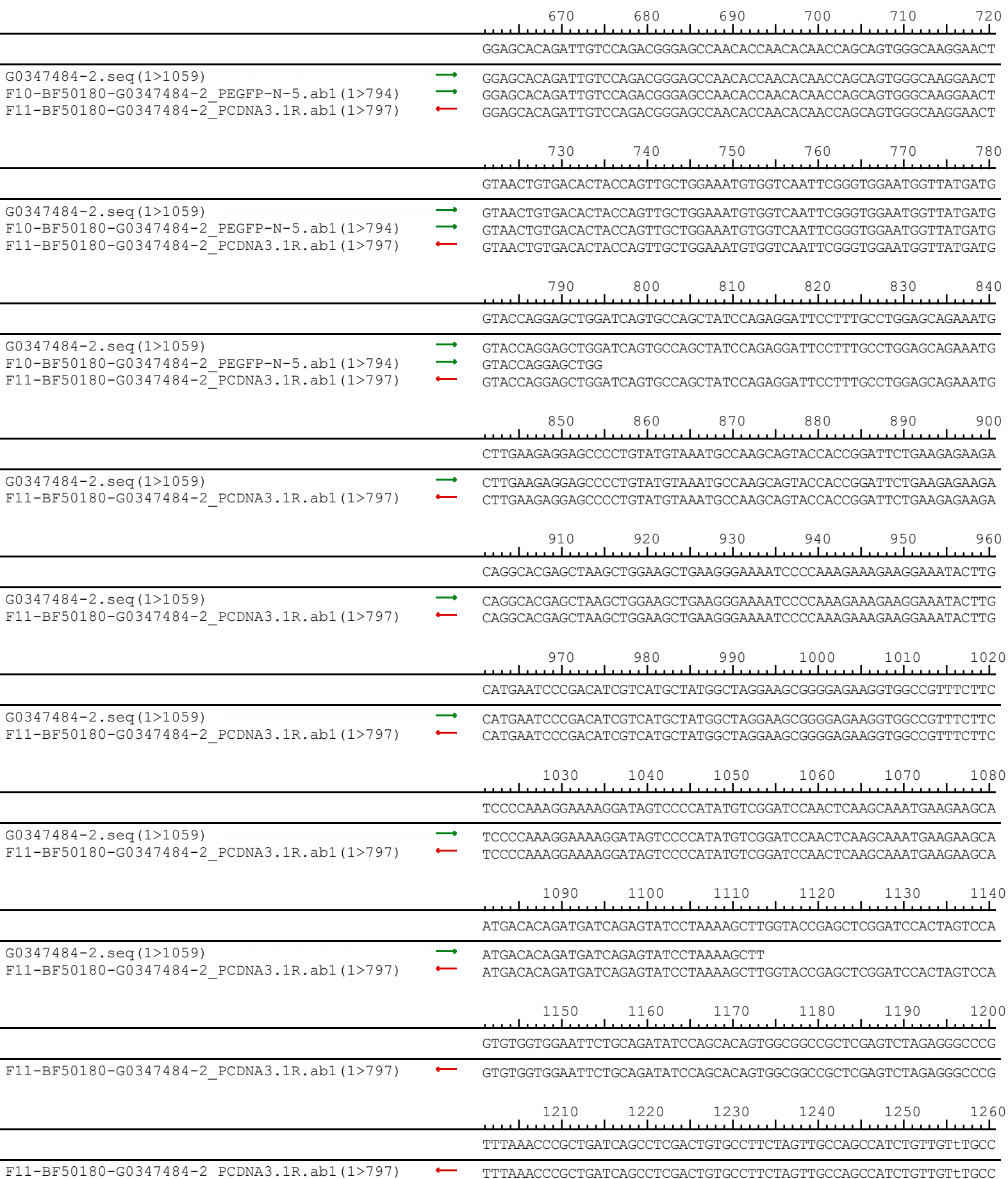

Supplement: Supplementary file 17 — Additional file17 (PDF 101 KB): NFYA Sequencing Report. [file 12983_2026_611_MOESM17_ESM.pdf]
