# Supplementary material for: Dynamic changes in chromatin accessibility reveal the role of NF-Y targeting AURKB in mediating cell cycle during asynchronous oogenesis in the Chinese Alligator (Alligator sinensis)
Source: Front Zool. 2026 Apr 29;23:24. doi: 10.1186/s12983-026-00611-8 (PMC13274144; doi:10.1186/s12983-026-00611-8)
Supplement: Supplementary file 4 — Additional file4 (PDF 95 KB): AURKB MUT-2 promoter in pGL3-Basic. [file 12983_2026_611_MOESM4_ESM.pdf]

```
LOCUS       Exported                6419 bp ds-DNA     circular SYN 15-一月-2025
DEFINITION  .
ACCESSION   .
VERSION     .
KEYWORDS    Untitled 21
SOURCE      synthetic DNA construct
  ORGANISM  synthetic DNA construct
REFERENCE   1  (bases 1 to 6419)
  AUTHORS   vdf3d
  TITLE     Direct Submission
  JOURNAL   Exported 2025年1月15日 星期三 from SnapGene 2.3.2
            http://www.snapgene.com
FEATURES             Location/Qualifiers
     source          1..6419
                     /organism="synthetic DNA construct"
                     /mol_type="other DNA"
     misc_feature    32..1659
                     /note="AURKB MUT3 promoter"
     CDS             1689..3341
                     /codon_start=1
                     /gene="luc+"
                     /product="firefly luciferase"
                     /EC_number="
                     "
                     /note="luciferase"
                     /note="enhanced luc+ version of the luciferase gene"
                     /protein_id="
                     "
                     /translation="MEDAKNIKKGPAPFYPLEDGTAGEQLHKAMKRYALVPGTIAFTDA
                     HIEVDITYAEYFEMSVRLAEAMKRYGLNTNHRIVVCSENSLQFFMPVLGALFIGVAVAP
                     ANDIYNERELLNSMGISQPTVVFVSKKGLQKILNVQKKLPIIQKIIIMDSKTDYQGFQS
                     MYTFVTSHLPPGFNEYDFVPESFDRDKTIALIMNSSGSTGLPKGVALPHRTACVRFSHA
                     RDPIFGNQIIPDTAILSVVPFHHGFGMFTTLGYLICGFRVVLMYRFEEELFLRSLQDYK
                     IQSALLVPTLFSFFAKSTLIDKYDLSNLHEIASGGAPLSKEVGEAVAKRFHLPGIRQGY
                     GLTETTSAILITPEGDDKPGAVGKVVPFFEAKVVDLDTGKTLGVNQRGELCVRGPMIMS
                     GYVNNPEATNALIDKDGWLHSGDIAYWDEDEHFFIVDRLKSLIKYKGYQVAPAELESIL
                     LQHPNIFDAGVAGLPDDDAGELPAAVVVLEHGKTMTEKEIVDYVASQVTTAKKLRGGVV
                     FVDEVPKGLTGKLDARKIREILIKAKKGGKIAV"
     polyA_signal    3382..3503
                     /note="SV40 poly(A) signal"
                     /note="SV40 polyadenylation signal"
     rep_origin      complement(3922..4510)
                     /direction=LEFT
                     /note="ori"
                     /note="high-copy-number ColE1/pMB1/pBR322/pUC origin of
                     replication"
     CDS             complement(4681..5541)
                     /codon_start=1
                     /gene="bla"
                     /product="beta-lactamase"
                     /note="AmpR"
                     /note="confers resistance to ampicillin, carbenicillin, and
                     related antibiotics"
                     /translation="MSIQHFRVALIPFFAAFCLPVFAHPETLVKVKDAEDQLGARVGYI
                     ELDLNSGKILESFRPEERFPMMSTFKVLLCGAVLSRIDAGQEQLGRRIHYSQNDLVEYS
                     PVTEKHLTDGMTVRELCSAAITMSDNTAANLLLTTIGGPKELTAFLHNMGDHVTRLDRW
                     EPELNEAIPNDERDTTMPVAMATTLRKLLTGELLTLASRQQLIDWMEADKVAGPLLRSA
                     LPAGWFIADKSGAGERGSRGIIAALGPDGKPSRIVVIYTTGSQATMDERNRQIAEIGAS
                     LIKHW"
     promoter        complement(5542..5646)
                     /gene="bla"
                     /note="AmpR promoter"
     rep_origin      5673..6128
                     /direction=RIGHT
                     /note="f1 ori"
                     /note="f1 bacteriophage origin of replication; arrow
                     indicates direction of (+) strand synthesis"
     polyA_signal    6259..6307
                     /note="synthetic polyadenylation signal"
     misc_feature    6321..6412
                     /note="pause site"
                     /note="RNA polymerase II transcriptional pause signal from
                     the human alpha-2 globin gene"
```

```
ORIGIN
        1 ggtaccgagc tcttacgcgt gctagcccgg gctcgaggga gcagtgcatg tggctgcaca
       61 gcctctccgc aaggcagcaa gacccatgag agtggagcct gagcagtgga tttaaaacaa
      121 tttttttttg gaaaagtatg tttttattca aatattataa aagcctaagt ctgtctgtct
      181 gtctgtaaca ctttatttgt gctctgattg gctgacaaac gtgcaaagca gcattctcac
      241 agaaggcagc cctccgcctg gatggtgggg gcaggggacc gggggggggg gaagggccag
      301 cagggccccg tccccctgca ggtaatgcgg ggtgtgggag cgggcccggg cccacggtgg
      361 tggggagggg agcaggcagg acccaagcag cagaagggaa gcaggagcag gtcggggggg
      421 gggggagggc tgtcccgcct gtcccttcac ccctgtcatt cttgacaggc aattggctag
      481 tagatgcgta aaagttatac gcatctccac cttctagtca cctgccatca cagaccttca
      541 ctcgcaccac agagtgcaaa acgcccgccc gcctgcccgc ccgcgggcgc cccgcgccgg
      601 tgcaacctcc gccggcccga gcactggatt tcttcttgat ttttaaggag atttttttcgt
      661 gtcccgggtc aaattagccc gatcaggccc aaatccatta gaatcacgca aggaccacac
      721 gtggccctgc taccggcaag tgtccccgcc ccgccccgcc ccgccgcgcc ttcagaggct
      781 tccaaaccct gtgacggccg gcgtcccgcg cgggccccag cccgaggcct cggctgcccc
      841 gagcttcgcc tgccctggct gtgcggctgc aggagcaagg agggggagtc tcccggtccc
      901 ataaagggcc ctgcagcccc tcctgcgcca tcccaacacc ggggcggggg gctcccctgg
      961 ctggggccag acgcccccag ggccctcac agtccaggag gggggcgggc acccgggttt
     1021 tcgggggcgg ggggcaggga ctcggggacc tgagagcccg agcggccccc aaacaaaccc
     1081 agccgaagaa ggcgccccgc cacaccgcaa cggtcaatgc cgcttttctg cgaaagggca
     1141 attccgctaa gcggcttcgg caccctcgca cgcggagtca ccacgccccg tctctgattg
     1201 gctggcggcg ccagctcccg gcccgccgtt ggctgaactc aactcaacac ccgcccccta
     1261 cctcctctcc gccgttacca ggcagaccag ctcccgtgac gcgttccgcc cccgcccgct
     1321 cagattgacg ggcagctcgg ccaacccgca ctaggtcccg cccctccagt tcttttgcgc
     1381 gcgcctgatt ccgcccggag ggaggcggga cttctaactc gccgcgccgc caatccgaag
     1441 ccgccacctc ccgcaggagc caatgggagc acaggcggca acgggctcgg cctccaaggg
     1501 ggaagggcgg agggagcgcg ccggcgggag ctgcggtcta gggccgggac acggcggccg
     1561 tcgcagccaa tgggagcgcg gggcggggtg gatttgaagc gcgaggcggc agcagcagcc
     1621 gtgggtaggt gagcgcggag gtaccggggg cgcaagcttg gcattccggt actgttggta
     1681 aagccaccat ggaagacgcc aaaaacataa agaaaggccc ggcgccattc tatccgctgg
     1741 aagatggaac cgctggagag caactgcata aggctatgaa gagatacgcc ctggttcctg
     1801 gaacaattgc ttttacagat gcacatatcg aggtggacat cacttacgct gagtacttcg
     1861 aaatgtccgt tcggttggca gaagctatga aacgatatgg gctgaataca aatcacagaa
     1921 tcgtcgtatg cagtgaaaac tctcttcaat tctttatgcc ggtgttgggc gcgttattta
     1981 tcggagttgc agttgcgccc gcgaacgaca tttataatga acgtgaattg ctcaacagta
     2041 tgggcatttc gcagcctacc gtggtgttcg tttccaaaaa ggggttgcaa aaaattttga
     2101 acgtgcaaaa aaagctccca atcatccaaa aaattattat catggattct aaaacggatt
     2161 accagggatt tcagtcgatg tacacgttcg tcacatctca tctacctccc ggttttaatg
     2221 aatacgattt tgtgccagag tccttcgata gggacaagac aattgcactg atcatgaact
     2281 cctctggatc tactggtctg cctaaaggtg tcgctctgcc tcatagaact gcctgcgtga
     2341 gattctcgca tgccagagat cctatttttg gcaatcaaat cattccggat actgcgattt
     2401 taagtgttgt tccattccat cacggttttg gaatgtttac tacactcgga tatttgatat
     2461 gtggatttcg agtcgtctta atgtatagat ttgaagaaga gctgtttctg aggagccttc
     2521 aggattacaa gattcaaagt gcgctgctgg tgccaaccct attctccttc ttcgccaaaa
     2581 gcactctgat tgacaaatac gatttatcta atttacacga aattgcttct ggtggcgctc
     2641 ccctctctaa ggaagtcggg gaagcggttg ccaagaggtt ccatctgcca ggtatcaggc
     2701 aaggatatgg gctcactgag actacatcag ctattctgat tacacccgag gggggatgata
     2761 aaccgggcgc ggtcggtaaa gttgttccat tttttgaagc gaaggttgtg gatctggata
     2821 ccgggaaaac gctgggcgtt aatcaaagag gcgaactgtg tgtgagaggt cctatgatta
     2881 tgtccggtta tgtaaacaat ccggaagcga ccaacgcctt gattgacaag gatggatggc
     2941 tacattctgg agacatagct tactgggacg aagacgaaca cttcttcatc gttgaccgcc
     3001 tgaagtctct gattaagtac aaaggctatc aggtggctcc cgctgaattg gaatccatct
     3061 tgctccaaca ccccaacatc ttcgacgcag gtgtcgcagg tcttcccgac gatgacgccg
     3121 gtgaacttcc cgccgccgtt gttgttttgg agcacggaaa gacgatgacg gaaaaagaga
     3181 tcgtggatta cgtcgccagt caagtaacaa ccgcgaaaaa gttgcgcgga ggagttgtgt
     3241 ttgtggacga agtaccgaaa ggtcttaccg gaaaactcga cgcaagaaaa atcagagaga
     3301 tcctcataaa ggccaagaag ggcggaaaga tcgccgtgta attctagagt cggggcggcc
     3361 ggccgcttcg agcagacatg ataagataca ttgatgagtt tggacaaacc acaactagaa
     3421 tgcagtgaaa aaaatgcttt atttgtgaaa tttgtgatgc tattgcttta tttgtaacca
     3481 ttataagctg caataaacaa gttaacaaca acaattgcat tcattttatg tttcaggttc
     3541 agggggaggt gtgggaggtt ttttaaagca agtaaaacct ctacaaatgt ggtaaaatcg
     3601 ataaggatcc gtcgaccgat gcccttgaga gccttcaacc cagtcagctc cttccggtgg
     3661 gcgcggggca tgactatcgt cgccgcactt atgactgtct tctttatcat gcaactcgta
     3721 ggacaggtgc cggcagcgct cttccgcttc ctcgctcact gactcgctgc gctcggtcgt
     3781 tcggctgcgg cgagcggtat cagctcactc aaaggcggta atacggttat ccacagaatc
     3841 aggggataac gcaggaaaga acatgtgagc aaaaggccag caaaaggcca ggaaccgtaa
     3901 aaaggccgcg ttgctggcgt ttttccatag gctccgcccc cctgacgagc atcacaaaaa
     3961 tcgacgctca agtcagaggt ggcgaaaccc gacaggacta taaagatacc aggcgtttcc
     4021 ccctggaagc tccctcgtgc gctctcctgt tccgaccctg ccgcttaccg gatacctgtc
     4081 cgcctttctc ccttcgggaa gcgtggcgct ttctcatagc tcacgctgta ggtatctcag
     4141 ttcggtgtag gtcgttcgct ccaagctggg ctgtgtgcac gaaccccccg ttcagcccga
     4201 ccgctgcgcc ttatccggta actatcgtct tgagtccaac ccggtaagac acgacttatc
     4261 gccactggca gcagccactg gtaacaggat tagcagagcg aggtatgtag gcggtgctac
```

```
     4321 agagttcttg aagtggtggc ctaactacgg ctacactaga agaacagtat ttggtatctg
     4381 cgctctgctg aagccagtta ccttcggaaa aagagttggt agctcttgat ccggcaaaca
     4441 aaccaccgct ggtagcggtg gtttttttgt ttgcaagcag cagattacgc gcagaaaaaa
     4501 aggatctcaa gaagatcctt tgatcttttc tacggggtct gacgctcagt ggaacgaaaa
     4561 ctcacgttaa gggattttgg tcatgagatt atcaaaaagg atcttcacct agatcctttt
     4621 aaattaaaaa tgaagtttta aatcaatcta aagtatatat gagtaaactt ggtctgacag
     4681 ttaccaatgc ttaatcagtg aggcacctat ctcagcgatc tgtctatttc gttcatccat
     4741 agttgcctga ctccccgtcg tgtagataac tacgatacgg gagggcttac catctggccc
     4801 cagtgctgca atgataccgc gagacccacg ctcaccggct ccagatttat cagcaataaa
     4861 ccagccagcc ggaagggccg agcgcagaag tggtcctgca actttatccg cctccatcca
     4921 gtctattaat tgttgccggg aagctagagt aagtagttcg ccagttaata gtttgcgcaa
     4981 cgttgttgcc attgctacag gcatcgtggt gtcacgctcg tcgtttggta tggcttcatt
     5041 cagctccggt tcccaacgat caaggcgagt tacatgatcc cccatgttgt gcaaaaaagc
     5101 ggttagctcc ttcggtcctc cgatcgttgt cagaagtaag ttggccgcag tgttatcact
     5161 catggttatg gcagcactgc ataattctct tactgtcatg ccatccgtaa gatgcttttc
     5221 tgtgactggt gagtactcaa ccaagtcatt ctgagaatag tgtatgcggc gaccgagttg
     5281 ctcttgcccg gcgtcaatac gggataatac cgcgccacat agcagaactt taaaagtgct
     5341 catcattgga aaacgttctt cggggcgaaa actctcaagg atcttaccgc tgttgagatc
     5401 cagttcgatg taacccactc gtgcacccaa ctgatcttca gcatctttta ctttcaccag
     5461 cgtttctggg tgagcaaaaa caggaaggca aaatgccgca aaaaagggaa taagggcgac
     5521 acggaaatgt tgaatactca tactcttcct ttttcaatat tattgaagca tttatcaggg
     5581 ttattgtctc atgagcggat acatatttga atgtatttag aaaaataaac aaataggggt
     5641 tccgcgcaca tttccccgaa aagtgccacc tgacgcgccc tgtagcggcg cattaagcgc
     5701 ggcgggtgtg gtggttacgc gcagcgtgac cgctacactt gccagcgccc tagcgcccgc
     5761 tcctttcgct ttcttccctt cctttctcgc cacgttcgcc ggctttcccc gtcaagctct
     5821 aaatcggggg ctccctttag ggttccgatt tagtgcttta cggcacctcg accccaaaaa
     5881 acttgattag ggtgatggtt cacgtagtgg gccatcgccc tgatagacgg tttttcgccc
     5941 tttgacgttg gagtccacgt tctttaatag tggactcttg ttccaaactg gaacaacact
     6001 caaccctatc tcggtctatt cttttgattt ataagggatt ttgccgattt cggcctattg
     6061 gttaaaaaat gagctgattt aacaaaaatt taacgcgaat tttaacaaaa tattaacgct
     6121 tacaatttgc cattcgccat tcaggctgcg caactgttgg gaagggcgat cggtgcgggc
     6181 ctcttcgcta ttacgccagc ccaagctacc atgataagta agtaatatta aggtacggga
     6241 ggtacttgga gcggccgcaa taaaatatct ttattttcat tacatctgtg tgttggtttt
     6301 ttgtgtgaat cgatagtact aacatacgct ctccatcaaa acaaaacgaa acaaaacaaa
     6361 ctagcaaaat aggctgtccc cagtgcaagt gcaggtgcca gaacatttct ctatcgata
//
```
