# Supplementary material for: Dynamic changes in chromatin accessibility reveal the role of NF-Y targeting AURKB in mediating cell cycle during asynchronous oogenesis in the Chinese Alligator (Alligator sinensis)
Source: Front Zool. 2026 Apr 29;23:24. doi: 10.1186/s12983-026-00611-8 (PMC13274144; doi:10.1186/s12983-026-00611-8)
Supplement: Supplementary file 8 — Additional file8 (PDF 149 KB): AURKB MUT-3 promoter Sequencing Report. [file 12983_2026_611_MOESM8_ESM.pdf]

```
                                                                     10        20        30        40
Consensus                                                TTTTCATTACATCTGTGTGTTGGTTTTTTGTGTGAATCGATAGTACTAA
E06-B59348-g0347484-5_g0347484-1-SEQ1R.ab1(1>798)     ←  TTTTCATTACATCTGTGTGTTGGTTTTTTGTGTGAATCGATAGTACTAA

                                                         50        60        70        80        90
Consensus                                                CATACGCTCTCCATCAAAACAAAACGAAACAAAACAAACTAGCAAAATA
E06-B59348-g0347484-5_g0347484-1-SEQ1R.ab1(1>798)     ←  CATACGCTCTCCATCAAAACAAAACGAAACAAAACAAACTAGCAAAATA
E08-B59348-g0347484-5_pgl3-basic-kpni-seqf.ab1(1>710) →                                         CTAGCAAAATA

                                                          100       110       120       130       140
Consensus                                                GGCTGTCCCCAGTGCAAGTGCAGGTGCCAGAACATTTCTCTATCGATAG
E06-B59348-g0347484-5_g0347484-1-SEQ1R.ab1(1>798)     ←  GGCTGTCCCCAGTGCAAGTGCAGGTGCCAGAACATTTCTCTATCGATAG
E08-B59348-g0347484-5_pgl3-basic-kpni-seqf.ab1(1>710) →  GGCTGTCCCCAGTGCAAGTGCAGGTGCCAGAACATTTCTCTATCGATAG

                                                           150       160       170       180       190
Consensus                                                GTACCGAGCTCTTACGCGTGCTAGCCCGGGCTCGAGGGAGCAGTGCATG
G0347484-5.seq(1>1628)                                →                                 CTCGAGGGAGCAGTGCATG
E06-B59348-g0347484-5_g0347484-1-SEQ1R.ab1(1>798)     ←  GTACCGAGCTCTTACGCGTGCTAGCCCGGGCTCGAGGGAGCAGTGCATG
E08-B59348-g0347484-5_pgl3-basic-kpni-seqf.ab1(1>710) →  GTACCGAGCTCTTACGCGTGCTAGCCCGGGCTCGAGGGAGCAGTGCATG

                                                            200       210       220       230       240
Consensus                                                TGGCTGCACAGCCTCTCCGCAAGGCAGCAAGACCCATGAGAGTGGAGCC
G0347484-5.seq(1>1628)                                →  TGGCTGCACAGCCTCTCCGCAAGGCAGCAAGACCCATGAGAGTGGAGCC
E06-B59348-g0347484-5_g0347484-1-SEQ1R.ab1(1>798)     ←  TGGCTGCACAGCCTCTCCGCAAGGCAGCAAGACCCATGAGAGTGGAGCC
E08-B59348-g0347484-5_pgl3-basic-kpni-seqf.ab1(1>710) →  TGGCTGCACAGCCTCTCCGCAAGGCAGCAAGACCCATGAGAGTGGAGCC

                                                             250       260       270       280       290
Consensus                                                TGAGCAGTGGATTTAAAACAATTTTTTTTTGGAAAAGTATGTTTTTATT
G0347484-5.seq(1>1628)                                →  TGAGCAGTGGATTTAAAACAATTTTTTTTTGGAAAAGTATGTTTTTATT
E06-B59348-g0347484-5_g0347484-1-SEQ1R.ab1(1>798)     ←  TGAGCAGTGGATTTAAAACAATTTTTTTTTGGAAAAGTATGTTTTTATT
E08-B59348-g0347484-5_pgl3-basic-kpni-seqf.ab1(1>710) →  TGAGCAGTGGATTTAAAACAATTTTTTTTTGGAAAAGTATGTTTTTATT

                                                              300       310       320       330       340
Consensus                                                CAAATATTATAAAAGCCTAAGTCTGTCTGTCTGTCTGTAACACTTTATT
G0347484-5.seq(1>1628)                                →  CAAATATTATAAAAGCCTAAGTCTGTCTGTCTGTCTGTAACACTTTATT
E06-B59348-g0347484-5_g0347484-1-SEQ1R.ab1(1>798)     ←  CAAATATTATAAAAGCCTAAGTCTGTCTGTCTGTCTGTAACACTTTATT
E08-B59348-g0347484-5_pgl3-basic-kpni-seqf.ab1(1>710) →  CAAATATTATAAAAGCCTAAGTCTGTCTGTCTGTCTGTAACACTTTATT

                                                               350       360       370       380       390
Consensus                                                TGTGCTCTGATTGGCTGACAAACGTGCAAAGCAGCATTCTCACAGAAGG
G0347484-5.seq(1>1628)                                →  TGTGCTCTGATTGGCTGACAAACGTGCAAAGCAGCATTCTCACAGAAGG
E06-B59348-g0347484-5_g0347484-1-SEQ1R.ab1(1>798)     ←  TGTGCTCTGATTGGCTGACAAACGTGCAAAGCAGCATTCTCACAGAAGG
E08-B59348-g0347484-5_pgl3-basic-kpni-seqf.ab1(1>710) →  TGTGCTCTGATTGGCTGACAAACGTGCAAAGCAGCATTCTCACAGAAGG

                                                                400       410       420       430       440
Consensus                                                CAGCCCTCCGCCTGGATGGTGGGGGCAGGGGACCGGGGGGGGGGGAAGG
G0347484-5.seq(1>1628)                                →  CAGCCCTCCGCCTGGATGGTGGGGGCAGGGGACCGGGGGGGGGGGAAGG
E06-B59348-g0347484-5_g0347484-1-SEQ1R.ab1(1>798)     ←  CAGCCCTCCGCCTGGATGGTGGGGGCAGGGGACCGGGGGGGGGGGAAGG
E08-B59348-g0347484-5_pgl3-basic-kpni-seqf.ab1(1>710) →  CAGCCCTCCGCCTGGATGGTGGGGGCAGGGGACCGGGGGGGGGGGAAGG

                                                                 450       460       470       480       490
Consensus                                                GCCAGCAGGGCCCCGTCCCCCTGCAGGTAATGCGGGGTGTGGGAGCGGG
G0347484-5.seq(1>1628)                                →  GCCAGCAGGGCCCCGTCCCCCTGCAGGTAATGCGGGGTGTGGGAGCGGG
E06-B59348-g0347484-5_g0347484-1-SEQ1R.ab1(1>798)     ←  GCCAGCAGGGCCCCGTCCCCCTGCAGGTAATGCGGGGTGTGGGAGCGGG
E08-B59348-g0347484-5_pgl3-basic-kpni-seqf.ab1(1>710) →  GCCAGCAGGGCCCCGTCCCCCTGCAGGTAATGCGGGGTGTGGGAGCGGG

                                                                  500       510       520       530
Consensus                                                CCCGGGCCCACGGTGGTGGGGAGGGGAGCAGGCAGGACCCAAGCAGCAG
G0347484-5.seq(1>1628)                                →  CCCGGGCCCACGGTGGTGGGGAGGGGAGCAGGCAGGACCCAAGCAGCAG
E06-B59348-g0347484-5_g0347484-1-SEQ1R.ab1(1>798)     ←  CCCGGGCCCACGGTGGTGGGGAGGGGAGCAGGCAGGACCCAAGCAGCAG
```

```
                                                                                     500       510       520       530
                                                                         CCCGGGCCCACGGTGGTGGGGAGGGGAGCAGGCAGGACCCAAGCAGCAG
E08-B59348-g0347484-5_pgl3-basic-kpni-seqf.ab1(1>710)          →        CCCGGGCCCACGGTGGTGGGGAGGGGAGCAGGCAGGACCCAAGCAGCAG

                                                                         540       550       560       570       580
                                                                         AAGGGAAGCAGGAGCAGGTCGGGGGGGGGGGAGGGCTGTCCCGCCTGT
G0347484-5.seq(1>1628)                                         →        AAGGGAAGCAGGAGCAGGTCGGGGGGGGGGGAGGGCTGTCCCGCCTGT
E06-B59348-g0347484-5_g0347484-1-SEQ1R.ab1(1>798)              ←        AAGGGAAGCAGGAGCAGGTCGGGGGGGGGGGAGGGCTGTCCCGCCTGT
E08-B59348-g0347484-5_pgl3-basic-kpni-seqf.ab1(1>710)          →        AAGGGAAGCAGGAGCAGGTCGGGGGGGGGGGAGGGCTGTCCCGCCTGT

                                                                          590       600       610       620       630
                                                                         CCCTTCACCCCTGTCATTCTTGACAGGCAATTGGCTAGTAGATGCGTAA
G0347484-5.seq(1>1628)                                         →        CCCTTCACCCCTGTCATTCTTGACAGGCAATTGGCTAGTAGATGCGTAA
E06-B59348-g0347484-5_g0347484-1-SEQ1R.ab1(1>798)              ←        CCCTTCACCCCTGTCATTCTTGACAGGCAATTGGCTAGTAGATGCGTAA
E08-B59348-g0347484-5_pgl3-basic-kpni-seqf.ab1(1>710)          →        CCCTTCACCCCTGTCATTCTTGACAGGCAATTGGCTAGTAGATGCGTAA

                                                                           640       650       660       670       680
                                                                         AAGTTATACGCATCTCCACCTTCTAGTCACCTGCCATCACAGACCTTCA
G0347484-5.seq(1>1628)                                         →        AAGTTATACGCATCTCCACCTTCTAGTCACCTGCCATCACAGACCTTCA
E06-B59348-g0347484-5_g0347484-1-SEQ1R.ab1(1>798)              ←        AAGTTATACGCATCTCCACCTTCTAGTCACCTGCCATCACAGACCTTCA
E08-B59348-g0347484-5_pgl3-basic-kpni-seqf.ab1(1>710)          →        AAGTTATACGCATCTCCACCTTCTAGTCACCTGCCATCACAGACCTTCA

                                                                            690       700       710       720       730
                                                                         CTCGCACCACAGAGTGCAAAACGCCCGCCCGCCTGCCCGCCCGCGGGCG
G0347484-5.seq(1>1628)                                         →        CTCGCACCACAGAGTGCAAAACGCCCGCCCGCCTGCCCGCCCGCGGGCG
E06-B59348-g0347484-5_g0347484-1-SEQ1R.ab1(1>798)              ←        CTCGCACCACAGAGTGCAAAACGCCCGCCCGCCTGCCCGCCCGCGGGCG
E08-B59348-g0347484-5_pgl3-basic-kpni-seqf.ab1(1>710)          →        CTCGCACCACAGAGTGCAAAACGCCCGCCCGCCTGCCCGCCCGCGGGCG

                                                                             740       750       760       770       780
                                                                         CCCCGCGCCGGTGCAACCTCCGCCGGCCCGAGCACTGGATTTCTTCTTG
G0347484-5.seq(1>1628)                                         →        CCCCGCGCCGGTGCAACCTCCGCCGGCCCGAGCACTGGATTTCTTCTTG
E06-B59348-g0347484-5_g0347484-1-SEQ1R.ab1(1>798)              ←        CCCCGCGCCGGTGCAACCTCCGCCGGCCCGAGCACTGGATTTCTTCTTG
E08-B59348-g0347484-5_pgl3-basic-kpni-seqf.ab1(1>710)          →        CCCCGCGCCGGTGCAACCTCCGCCGGCCCGAGCACTGGATTTCTTCTTG
E07-B59348-g0347484-5_g0347484-1-SEQ2.ab1(1>797)               →                       ACCTCCGCCGGCCCGAGCACTGGATTTCTTCTTG

                                                                              790       800       810       820       830
                                                                         ATTTTTAAGGAGATTTTTTCGTGTCCCGGGTCAAATTAGCCCGATCAGG
G0347484-5.seq(1>1628)                                         →        ATTTTTAAGGAGATTTTTTCGTGTCCCGGGTCAAATTAGCCCGATCAGG
E06-B59348-g0347484-5_g0347484-1-SEQ1R.ab1(1>798)              ←        ATTTtTAAGGAGAT
E08-B59348-g0347484-5_pgl3-basic-kpni-seqf.ab1(1>710)          →        ATTTTTAAGGAGA
E07-B59348-g0347484-5_g0347484-1-SEQ2.ab1(1>797)               →        ATTTTTAAGGAGATTTTTTCGTGTCCCGGGTCAAATTAGCCCGATCAGG

                                                                               840       850       860       870       880
                                                                         CCCAAATCCATTAGAATCACGCAAGGACCACACGTGGCCCTGCTACCGG
G0347484-5.seq(1>1628)                                         →        CCCAAATCCATTAGAATCACGCAAGGACCACACGTGGCCCTGCTACCGG
E07-B59348-g0347484-5_g0347484-1-SEQ2.ab1(1>797)               →        CCCAAATCCATTAGAATCACGCAAGGACCACACGTGGCCCTGCTACCGG

                                                                                890       900       910       920       930
                                                                         CAAGTGTCCCCGCCCCGCCCCGCCCCGCCGCGCCTTCAGAGGCTTCCAA
G0347484-5.seq(1>1628)                                         →        CAAGTGTCCCCGCCCCGCCCCGCCCCGCCGCGCCTTCAGAGGCTTCCAA
E07-B59348-g0347484-5_g0347484-1-SEQ2.ab1(1>797)               →        CAAGTGTCCCCGCCCCGCCCCGCCCCGCCGCGCCTTCAGAGGCTTCCAA

                                                                                 940       950       960       970       980
                                                                         ACCCTGTGACGGCCGGCGTCCCGCGCGGGCCCCAGCCCGAGGCCTCGGC
G0347484-5.seq(1>1628)                                         →        ACCCTGTGACGGCCGGCGTCCCGCGCGGGCCCCAGCCCGAGGCCTCGGC
E07-B59348-g0347484-5_g0347484-1-SEQ2.ab1(1>797)               →        ACCCTGTGACGGCCGGCGTCCCGCGCGGGCCCCAGCCCGAGGCCTCGGC
```

990 1000 1010 1020

TGCCCCGAGCTTCGCCTGCCCTGGCTGTGCGGCTGCAGGAGCAAGGAGG

G0347484-5.seq(1>1628) → TGCCCCGAGCTTCGCCTGCCCTGGCTGTGCGGCTGCAGGAGCAAGGAGG
E07-B59348-g0347484-5_g0347484-1-SEQ2.ab1(1>797) → TGCCCCGAGCTTCGCCTGCCCTGGCTGTGCGGCTGCAGGAGCAAGGAGG

1030 1040 1050 1060 1070

GGGAGTCTCCCGGTCCCATAAAGGGCCCTGCAGCCCCTCCTGCGCCATC

G0347484-5.seq(1>1628) → GGGAGTCTCCCGGTCCCATAAAGGGCCCTGCAGCCCCTCCTGCGCCATC
E07-B59348-g0347484-5_g0347484-1-SEQ2.ab1(1>797) → GGGAGTCTCCCGGTCCCATAAAGGGCCCTGCAGCCCCTCCTGCGCCATC
E09-B59348-g0347484-5_RVp3-R.ab1(1>797) ← ATAAAGGGCCCTGCAGCCCCTCCTGCGCCATC

1080 1090 1100 1110 1120

CCAACACCGGGGCGGGGGGCTCCCCTGGCTGGGGCCAGACGCCCCCAGG

G0347484-5.seq(1>1628) → CCAACACCGGGGCGGGGGGCTCCCCTGGCTGGGGCCAGACGCCCCCAGG
E07-B59348-g0347484-5_g0347484-1-SEQ2.ab1(1>797) → CCAACACCGGGGCGGGGGGCTCCCCTGGCTGGGGCCAGACGCCCCCAGG
E09-B59348-g0347484-5_RVp3-R.ab1(1>797) ← CCAACACCGGGGCGGGGGGCTCCCCTGGCTGGGGCCAGACGCCCCCAGG

1130 1140 1150 1160 1170

GGCCCTCACAGTCCAGGAGGGGGGCGGGCACCCGGGTTTTCGGGGGCGG

G0347484-5.seq(1>1628) → GGCCCTCACAGTCCAGGAGGGGGGCGGGCACCCGGGTTTTCGGGGGCGG
E07-B59348-g0347484-5_g0347484-1-SEQ2.ab1(1>797) → GGCCCTCACAGTCCAGGAGGGGGGCGGGCACCCGGGTTTTCGGGGGCGG
E09-B59348-g0347484-5_RVp3-R.ab1(1>797) ← GGCCCTCACAGTCCAGGAGGGGGGCGGGCACCCGGGTTTTCGGGGGCGG

1180 1190 1200 1210 1220

GGGGCAGGGACTCGGGGACCTGAGAGCCCGAGCGGCCCCCAAACAAACC

G0347484-5.seq(1>1628) → GGGGCAGGGACTCGGGGACCTGAGAGCCCGAGCGGCCCCCAAACAAACC
E07-B59348-g0347484-5_g0347484-1-SEQ2.ab1(1>797) → GGGGCAGGGACTCGGGGACCTGAGAGCCCGAGCGGCCCCCAAACAAACC
E09-B59348-g0347484-5_RVp3-R.ab1(1>797) ← GGGGCAGGGACTCGGGGACCTGAGAGCCCGAGCGGCCCCCAAACAAACC

1230 1240 1250 1260 1270

CAGCCGAAGAAGGCGCCCCGCCACACCGCAACGGTCAATGCCGCTTTTC

G0347484-5.seq(1>1628) → CAGCCGAAGAAGGCGCCCCGCCACACCGCAACGGTCAATGCCGCTTTTC
E07-B59348-g0347484-5_g0347484-1-SEQ2.ab1(1>797) → CAGCCGAAGAAGGCGCCCCGCCACACCGCAACGGTCAATGCCGCTTTTC
E09-B59348-g0347484-5_RVp3-R.ab1(1>797) ← CAGCCGAAGAAGGCGCCCCGCCACACCGCAACGGTCAATGCCGCTTTTC

1280 1290 1300 1310 1320

TGCGAAAGGGCAATTCCGCTAAGCGGCTTCGGCACCCTCGCACGCGGAG

G0347484-5.seq(1>1628) → TGCGAAAGGGCAATTCCGCTAAGCGGCTTCGGCACCCTCGCACGCGGAG
E07-B59348-g0347484-5_g0347484-1-SEQ2.ab1(1>797) → TGCGAAAGGGCAATTCCGCTAAGCGGCTTCGGCACCCTCGCACGCGGAG
E09-B59348-g0347484-5_RVp3-R.ab1(1>797) ← TGCGAAAGGGCAATTCCGCTAAGCGGCTTCGGCACCCTCGCACGCGGAG

1330 1340 1350 1360 1370

TCACCACGCCCCGTCTCTGATTGGCTGGCGGCGCCAGCTCCCGGCCCGC

G0347484-5.seq(1>1628) → TCACCACGCCCCGTCTCTGATTGGCTGGCGGCGCCAGCTCCCGGCCCGC
E07-B59348-g0347484-5_g0347484-1-SEQ2.ab1(1>797) → TCACCACGCCCCGTCTCTGATTGGCTGGCGGCGCCAGCTCCCGGCCCGC
E09-B59348-g0347484-5_RVp3-R.ab1(1>797) ← TCACCACGCCCCGTCTCTGATTGGCTGGCGGCGCCAGCTCCCGGCCCGC

1380 1390 1400 1410 1420

CGTTGGCTGAACTCAACTCAACACCCGCCCCCTACCTCCTCTCCGCCGT

G0347484-5.seq(1>1628) → CGTTGGCTGAACTCAACTCAACACCCGCCCCCTACCTCCTCTCCGCCGT
E07-B59348-g0347484-5_g0347484-1-SEQ2.ab1(1>797) → CGTTGGCTGAACTCAACTCAACACCCGCCCCCTACCTCCTCTCCGCCGT
E09-B59348-g0347484-5_RVp3-R.ab1(1>797) ← CGTTGGCTGAACTCAACTCAACACCCGCCCCCTACCTCCTCTCCGCCGT

1430 1440 1450 1460 1470

TACCAGGCAGACCAGCTCCCGTGACGCGTTCCGCCCCCGCCCGCTCAGA

G0347484-5.seq(1>1628) → TACCAGGCAGACCAGCTCCCGTGACGCGTTCCGCCCCCGCCCGCTCAGA
E07-B59348-g0347484-5_g0347484-1-SEQ2.ab1(1>797) → TACCAGGCAGACCAGCTCCCGTGACGCGTTCCGCCCCCGCCCGCTCAGA
E09-B59348-g0347484-5_RVp3-R.ab1(1>797) ← TACCAGGCAGACCAGCTCCCGTGACGCGTTCCGCCCCCGCCCGCTCAGA

```
                                                        1480      1490      1500      1510
                                                   TTGACGGGCAGCTCGGCCAACCCGCACTAGGTCCCGCCCCTCCAGTTCT
G0347484-5.seq(1>1628)                          →  TTGACGGGCAGCTCGGCCAACCCGCACTAGGTCCCGCCCCTCCAGTTCT
E07-B59348-g0347484-5_g0347484-1-SEQ2.ab1(1>797) →  TTGACGGGCAGCTCGGCCAACCCGCACTAGGTCCCGCCCCTCCAGTTCT
E09-B59348-g0347484-5_RVp3-R.ab1(1>797)         ←  TTGACGGGCAGCTCGGCCAACCCGCACTAGGTCCCGCCCCTCCAGTTCT
```

```
                                                   1520      1530      1540      1550      1560
                                                   TTTGCGCGCGCCTGATTCCGCCCGGAGGGAGGCGGGACTTCTAACTCGC
G0347484-5.seq(1>1628)                          →  TTTGCGCGCGCCTGATTCCGCCCGGAGGGAGGCGGGACTTCTAACTCGC
E07-B59348-g0347484-5_g0347484-1-SEQ2.ab1(1>797) →  TTTGCGCGCGCCTGATTCCGCCCGGAGG
E09-B59348-g0347484-5_RVp3-R.ab1(1>797)         ←  TTTGCGCGCGCCTGATTCCGCCCGGAGGGAGGCGGGACTTCTAACTCGC
```

```
                                                    1570      1580      1590      1600      1610
                                                   CGCGCAGCCGCCACCTCCCGCAGGAGCCAATGGGAGCACAGGCGGCAAC
G0347484-5.seq(1>1628)                          →  CGCGCAGCCGCCACCTCCCGCAGGAGCCAATGGGAGCACAGGCGGCAAC
E09-B59348-g0347484-5_RVp3-R.ab1(1>797)         ←  CGCGCAGCCGCCACCTCCCGCAGGAGCCAATGGGAGCACAGGCGGCAAC
```

```
                                                   1620      1630      1640      1650      1660
                                                   GGGCTCGGCCTCCAAGGGGGAAGGGCGGAGGGAGCGCGCGGCCAATGAA
G0347484-5.seq(1>1628)                          →  GGGCTCGGCCTCCAAGGGGGAAGGGCGGAGGGAGCGCGCGGCCAATGAA
E09-B59348-g0347484-5_RVp3-R.ab1(1>797)         ←  GGGCTCGGCCTCCAAGGGGGAAGGGCGGAGGGAGCGCGCGGCCAATGAA
```

```
                                                     1670      1680      1690      1700      1710
                                                   ACGGCGGGAGCTGCGGTCTAGGGCCGGGACACGGCGGCCGTCGCAGCCA
G0347484-5.seq(1>1628)                          →  ACGGCGGGAGCTGCGGTCTAGGGCCGGGACACGGCGGCCGTCGCAGCCA
E09-B59348-g0347484-5_RVp3-R.ab1(1>797)         ←  ACGGCGGGAGCTGCGGTCTAGGGCCGGGACACGGCGGCCGTCGCAGCCA
```

```
                                                      1720      1730      1740      1750      1760
                                                   ATGGGAGCGCGGGGCGGGGTGGATTTGAAGCGCGAGGCGGCAGCAGCAG
G0347484-5.seq(1>1628)                          →  ATGGGAGCGCGGGGCGGGGTGGATTTGAAGCGCGAGGCGGCAGCAGCAG
E09-B59348-g0347484-5_RVp3-R.ab1(1>797)         ←  ATGGGAGCGCGGGGCGGGGTGGATTTGAAGCGCGAGGCGGCAGCAGCAG
```

```
                                                       1770      1780      1790      1800      1810
                                                   CCGTGGGTAGGTGAGCGCGGAGGTACCGGGGGCGCAAGCTTGGCATTCC
G0347484-5.seq(1>1628)                          →  CCGTGGGTAGGTGAGCGCGGAGGTACCGGGGGCGCAAGCTT
E09-B59348-g0347484-5_RVp3-R.ab1(1>797)         ←  CCGTGGGTAGGTGAGCGCGGAGGTACCGGGGGCGCAAGCTTGGCATTCC
```

```
                                                        1820      1830      1840
                                                   GGTACTGTTGGTAAAGCCACCATGGAAGAC
E09-B59348-g0347484-5_RVp3-R.ab1(1>797)         ←  GGTACTGTTGGTAAAGCCACCATGGAAGAC
```
