# Supplementary material for: Dynamic changes in chromatin accessibility reveal the role of NF-Y targeting AURKB in mediating cell cycle during asynchronous oogenesis in the Chinese Alligator (Alligator sinensis)
Source: Front Zool. 2026 Apr 29;23:24. doi: 10.1186/s12983-026-00611-8 (PMC13274144; doi:10.1186/s12983-026-00611-8)
Supplement: Supplementary file 10 — Additional file10 (PDF 1043 KB): AURKB MUT-all promoter in pGL3-Basic. [file 12983_2026_611_MOESM10_ESM.pdf]

NULNULNULSOSnapGeneNULSOHNUL
NULBELNULNULNULEM
USggtaccgagctcttacgcgtgctagcccgggCTCGAGGGAGCAGTGCATGTGGCTGCACAGCCTCTCCGCAAGGCAGCAAGACC
CATGAGAGTGGAGCCTGAGCAGTGGATTTAAAACAATTTTTTTTTGGAAAAGTATGTTTTTATTCAAATATTATAAAAGCCTAAGT
CTGTCTGTCTGTCTGTAACACTTTATTTGTGCTCTGATTGGCTGACAAACGTGCAAAGCAGCATTCTCACAGAAGGCAGCCCTCCG
CCTGGATGGTGGGGGCAGGGGACCGGGGGGGGGGGAAGGGCCAGCAGGGCCCCGTCCCCCTGCAGGTAATGCGGGGTGTGGGAGCG
GGCCCGGGCCCACGGTGGTGGGGAGGGGAGCAGGCAGGACCCAAGCAGCAGAAGGGAAGCAGGAGCAGGTCGGGGGGGGGGGGAGG
GCTGTCCCGCCTGTCCCTTCACCCCTGTCATTCTTGACAGGCAATTGGCTAGTAGATGCGTAAAAGTTATACGCATCTCCACCTTC
TAGTCACCTGCCATCACAGACCTTCACTCGCACCACAGAGTGCAAAACGCCCGCCCGCCTGCCCGCCCGCGGGCGCCCCGCGCCGG
TGCAACCTCCGCCGGCCCGAGCACTGGATTTCTTCTTGATTTTTAAGGAGATTTTTTCGTGTCCCGGGTCAAATTAGCCCGATCAG
GCCCAAATCCATTAGAATCACGCAAGGACCACACGTGGCCCTGCTACCGGCAAGTGTCCCCGCCCCGCCCCGCCCCGCCGCGCCTT
CAGAGGCTTCCAAACCCTGTGACGGCCGGCGTCCCGCGCGGGCCCCAGCCCGAGGCCTCGGCTGCCCCGAGCTTCGCCTGCCCTGG
CTGTGCGGCTGCAGGAGCAAGGAGGGGGAGTCTCCCGGTCCCATAAAGGGCCCTGCAGCCCCTCCTGCGCCATCCCAACACCGGGG
CGGGGGGCTCCCCTGGCTGGGGCCAGACGCCCCCAGGGGCCCTCACAGTCCAGGAGGGGGGCGGGCACCCGGGTTTCGGGGGCGG
GGGGCAGGGACTCGGGGACCTGAGAGCCCGAGCGGCCCCCAAACAAACCCAGCCGAAGAAGGCGCCCCGCCACACCGCAACGGTCA
ATGCCGCTTTTCTGCGAAAGGGCAATTCCGCTAAGCGGCTTCGGCACCCTCGCACGCGGAGTCACCACGCCCCGTCTCTGATTGGC
TGGCGGCGCCAGCTCCCGGCCCGCCGTTGGCTGAACTCAACTCAACACCCGCCCCCTACCTCCTCTCCGCCGTTACCAGGCAGACC
AGCTCCCGTGACGCGTTCCGCCCCCGCCCGCTCAGATTGACGGGCAGCTCGGCCAACCCGCACTAGGTCCCGCCCCTCCAGTTCTT
TTGCGCGCGCCTGATTCCGCCCGGAGGGAGGCGGGACTTCTAACTCGCCGCGCAGCCGCCACCTCCCGCAGGAGCCAATGGGAGCA
CAGGCGGCAACGGGCTCGGCCTCCAAGGGGGAAGGGCGGAGGGAGCGCGCCGGCGGGAGCTGCGGTCTAGGGCCGGGACACGGCGG
CCGTCGCAGCCAATGGGAGCGCGGGGCGGGGTGGATTTGAAGCGCGAGGCGGCAGCAGCAGCCGTGGGTAGGTGAGCGCGGAGGTA
CCGGGGGCGCAAGCTTggcattccggtactgttggtaaagccaccatggaagacgccaaaaacataaagaaaggcccggcgccatt
ctatccgctggaagatggaaccgctggagagcaactgcataaggctatgaagagatacgccctggttcctggaacaattgctttta
cagatgcacatatcgaggtggacatcacttacgctgagtacttcgaaatgtccgttcggttggcagaagctatgaaacgatatggg
ctgaatacaaatcacagaatcgtcgtatgcagtgaaaactctcttcaattctttatgccggtgttgggcgcgttatttatcggagt
tgcagttgcgcccgcgaacgacatttataatgaacgtgaattgctcaacagtatgggcatttcgcagcctaccgtggtgttcgttt
ccaaaaaggggttgcaaaaaattttgaacgtgcaaaaaaagctcccaatcatccaaaaaattattatcatggattctaaaacggat
taccagggatttcagtcgatgtacacgttcgtcacatctcatctacctcccggttttaatgaatacgattttgtgccagagtcctt
cgatagggacaagacaattgcactgatcatgaactcctctggatctactggtctgcctaaaggtgtcgctctgcctcatagaactg
cctgcgtgagattctcgcatgccagagatcctatttttggcaatcaaatcattccggatactgcgattttaagtgttgttccattc
catcacggttttggaatgtttactacactcggatatttgatatgtggatttcgagtcgtcttaatgtatagatttgaagaagagct
gtttctgaggagccttcaggattacaagattcaaagtgcgctgctggtgccaaccctattctccttcttcgccaaaagcactctga
ttgacaaatacgatttatctaatttacacgaaattgcttctggtggcgctcccctctctaaggaagtcggggaagcggttgccaag
aggttccatctgccaggtatcaggcaaggatatgggctcactgagactacatcagctattctgattacacccgaggggggatgataa
accgggcgcggtcggtaaagttgttccattttttgaagcgaaggttgtggatctggataccgggaaaacgctgggcgttaatcaaa
gaggcgaactgtgtgtgagaggtcctatgattatgtccggttatgtaaacaatccggaagcgaccaacgccttgattgacaaggat
ggatggctacattctggagacatagcttactgggacgaagacgaacacttcttcatcgttgaccgcctgaagtctctgattaagta
caaaggctatcaggtggctcccgctgaattggaatccatcttgctccaacaccccaacatcttcgacgcaggtgtcgcaggtcttc
ccgacgatgacgccggtgaacttcccgccgccgttgttgttttggagcacggaaagacgatgacggaaaaagagatcgtggattac
gtcgccagtcaagtaacaaccgcgaaaaagttgcgcggaggagttgtgtttgtggacgaagtaccgaaaggtcttaccggaaaact
cgacgcaagaaaaatcagagagatcctcataaaggccaagaagggcggaaagatcgccgtgtaattctagagtcggggcggccggc
cgcttcgagcagacatgataagatacattgatgagtttggacaaaccacaactagaatgcagtgaaaaaaatgctttatttgtgaa
atttgtgatgctattgctttatttgtaaccattataagctgcaataaacaagttaacaacaacaattgcattcattttatgtttca
ggttcagggggaggtgtgggaggttttttaaagcaagtaaaacctctacaaatgtggtaaaatcgataaggatccgtcgaccgatg
cccttgagagccttcaacccagtcagctccttccggtgggcgcggggcatgactatcgtcgccgcacttatgactgtcttctttat
catgcaactcgtaggacaggtgccggcagcgctcttccgcttcctcgctcactgactcgctgcgctcggtcgttcggctgcggcga
gcggtatcagctcactcaaaggcggtaatacggttatccacagaatcaggggataacgcaggaaagaacatgtgagcaaaaggcca
gcaaaaggccaggaaccgtaaaaaggccgcgttgctggcgtttttccataggctccgcccccctgacgagcatcacaaaaatcgac
gctcaagtcagaggtggcgaaacccgacaggactataaagataccaggcgtttccccctggaagctccctcgtgcgctctcctgtt
ccgaccctgccgcttaccggatacctgtccgcctttctcccttcgggaagcgtggcgctttctcaTAgctcacgctgtaggtatct
cagttcggtgtaggtcgttcgctccaagctgggctgtgtgcacgaaccccccgttcagcccgaccgctgcgccttatccggtaact
atcgtcttgagtccaacccggtaagacacgacttatcgccactggcagcagccactggtaacaggattagcagagcgaggtatgta
ggcggtgctacagagttcttgaagtggtggcctaactacggctacactagaagaacagtatttggtatctgcgctctgctgaagcc
agttaccttcggaaaaagagttggtagctcttgatccggcaaacaaaccaccgctggtagcggtggtttttttgtttgcaagcagc
agattacgcgcagaaaaaaaggatctcaagaagatcctttgatcttttctacggggtctgacgctcagtggaacgaaaactcacgt
taagggattttggtcatgagattatcaaaaaggatcttcacctagatccttttaaattaaaaatgaagttttaaatcaatctaaag
tatatatgagtaaacttggtctgacagttaccaatgcttaatcagtgaggcacctatctcagcgatctgtctatttcgttcatcca
tagttgcctgactccccgtcgtgtagataactacgatacgggagggcttaccatctggccccagtgctgcaatgataccgcgagac
ccacgctcaccggctccagatttatcagcaataaaccagccagccggaagggccgagcgcagaagtggtcctgcaactttatccgc
ctccatccagtctattaattgttgccgggaagctagagtaagtagttcgccagttaatagtttgcgcaacgttgttgccattgcta
caggcatcgtggtgtcacgctcgtcgtttggtatggcttcattcagctccggttcccaacgatcaaggcgagttacatgatccccc
atgttgtgcaaaaaagcggttagctccttcggtcctccgatcgttgtcagaagtaagttggccgcagtgttatcactcatggttat
ggcagcactgcataattctcttactgtcatgccatccgtaagatgcttttctgtgactggtgagtactcaaccaagtcattctgag
aatagtgtatgcggcgaccgagttgctcttgcccggcgtcaatacgggataataccgcgccacatagcagaactttaaaagtgctc
atcattggaaaacgttcttcggggcgaaaactctcaaggatcttaccgctgttgagatccagttcgatgtaacccactcgtgcacc
caactgatcttcagcatcttttactttcaccagcgtttctgggtgagcaaaaacaggaaggcaaaatgccgcaaaaaagggaataa
gggcgacacggaaatgttgaatactcatactcttcctttttcaatattattgaagcatttatcagggttattgtctcatgagcgga
tacatatttgaatgtatttagaaaaataaacaaataggggttccgcgcacatttccccgaaaagtgccacctgacgcgccctgtag
cggcgcattaagcgcggcgggtgtggtggttacgcgcagcgtgaccgctacacttgccagcgccctagcgcccgctcctttcgctt
tcttcccttcctttctcgccacgttcgccggctttccccgtcaagctctaaatcgggggctccctttagggttccgatttagtgct
ttacggcacctcgaccccaaaaaacttgattagggtgatggttcacgtagtgggccatcgccctgatagacggtttttcgcccttt
gacgttggagtccacgttctttaatagtggactcttgttccaaactggaacaacactcaaccctatctcggtctattcttttgatt
tataagggattttgccgatttcggcctattggttaaaaaatgagctgatttaacaaaaatttaacgcgaattttaacaaaatatta

acgCTTACAATTTGccattcgccattcaggctgcgcaactgttgggaagggcgatcggtgcgggcctcttcgctattacgccagcc
caagctaccatgataagtaagtaatattaaggtacgggaggtacttggagcggccgcaataaaatatctttattttcattacatct
gtgtgttggtttttttgtgtgaatcgatagtactaacatacgctctccatcaaaacaaaacgaaacaaaacaaactagcaaaatagg
ctgtccccagtgcaagtgcaggtgccagaacatttctctatcgataSTXNULNUL8DC3SOHNULNULNUL<NULNULNUL%NUL
NULNUL
3 NULNULNUL,NULNULNUL#NULNULNUL NULNULNULRSNULNULNULSINULNULNUL
NULNULNULSYNNULNULNULEMNULNULNULDC3NULNULNUL(NULNULNULFSNULNULNUL
NULNULNUL"NULNULNULSUBNULNULNULDC2NULNULNULDC4NULNULNUL!NULNULNUL%NULNULNULFSNULNUL
NULDLENULNULNULDC3NULNULNULDLENULNULNULCANNULNULNUL%NULNULNULETBNULNULNULDC4NULNUL
NULDC1NULNULNULVTNULNULNULDLENULNULNUL!NULNULNULDC3NULNULNULETBNULNULNULEMNULNULNUL
FFNULNULNULETBNULNULNULSONULNULNULVTNULNULNULUSNULNULNULDC2NULNULNULSYNNULNULNULESC
NULNULNULDC1NULNULNULDLENULNULNULDC4NULNULNULDC3NULNULNUL)NULNULNULDC3NULNULNULSONUL
NULNUL+NULNULNUL/NULNULNUL&NULNULNULSINULNULNULDC3NULNULNUL"NULNULNULDC1NULNULNULSYN
NULNULNUL)NULNULNUL!NULNULNUL'NULNULNULSUBNULNULNUL1NULNULNUL$NULNULNULCANNULNULNUL
SUBNULNULNULUSNULNULNULSYNNULNULNULCANNULNULNULETBNULNULNULSONULNULNULETBNULNULNUL
DC2NULNULNULETBNULNULNULDC2NULNULNULSUBNULNULNUL#NULNULNULEMNULNULNULCANNULNULNUL)
NULNULNULESCNULNULNULGSNULNULNULESCNULNULNUL&NULNULNULRNULNULNULGSNULNULNUL"NULNUL
NULDC3NULNULNULSYNNULNULNULSYNNULNULNULDC3NULNULNULDC3NULNULNUL
NULNULNULEMNULNULNULNAKNULNULNULSINULNULNULSYNNULNULNULDC1NULNULNULDC1NULNULNULSINUL
NULNULSYNNULNULNUL
4 NULNULNULDC3NULNULNULDC4NULNULNULCANNULNULNUL+NULNULNULCANNULNULNULDC2NULNULNULGSNUL
NULNULCANNULNULNULDC4NULNULNULDC3NULNULNULDC1NULNULNUL
5 NULNULNULGSNULNULNULDC3NULNULNULRSNULNULNULDC3NULNULNULDC1NULNULNULDC4NULNULNULFFNUL
NULNULSINULNULNULETBNULNULNULEMNULNULNULDC2NULNULNULSINULNULNULSYNNULNULNULDC3NULNUL
NUL
NULNULNULESCNULNULNULCANNULNULNULSYNNULNULNULEMNULNULNULSUBNULNULNULNAKNULNULNULESC
NULNULNULSONULNULNULCANNULNULNULDC4NULNULNULNAKNULNULNULFSNULNULNULNAKNULNULNULFSNUL
NULNUL
6 NULNULNULFFNULNULNUL
7 NULNULNULFFNULNULNULNAKNULNULNULGSNULNULNULSINULNULNULDLENULNULNULDC3NULNULNUL
NULNULNULSUBNULNULNULEMNULNULNULDC3NULNULNULDC4NULNULNUL
8 NULNULNULNAKNULNULNULSUBNULNULNULEMNULNULNULDC4NULNULNULEMNULNULNULBSNULNULNULEMNUL
NULNUL
NULNULNULSUBNULNULNULRSNULNULNUL#NULNULNUL7NULNULNULGSNULNULNULRSNULNULNULCANNULNUL
NUL#NULNULNULGSNULNULNULCANNULNULNULDLENULNULNULNAKNULNULNULEMNULNULNULSUBNULNULNUL
EMNULNULNULETBNULNULNULCANNULNULNULFSNULNULNULDC2NULNULNUL#NULNULNULEMNULNULNULESC
NULNULNULSUBNULNULNULDC4NULNULNULUSNULNULNUL'NULNULNULDC3NULNULNULUSNULNULNUL#NULNUL
NULDC2NULNULNULDC3NULNULNULNAKNULNULNULSONULNULNULDC2NULNULNULACKNULNULNULSUBNULNUL
NULSINULNULNULRSNULNULNULFSNULNULNUL$NULNULNUL=NULNULNUL%NULNULNULSUBNULNULNULETBNUL
NULNUL&NULNULNULFSNULNULNUL,NULNULNULSYNNULNULNULFSNULNULNULDC1NULNULNULSINULNULNUL
SUBNULNULNULCANNULNULNULDC4NULNULNULDLENULNULNULNAKNULNULNULSYNNULNULNULFSNULNULNUL
FSNULNULNULEMNULNULNULUSNULNULNULFFNULNULNULVTNULNULNULDC4NULNULNULDC1NULNULNULSINUL
NULNULSONULNULNULGSNULNULNUL&NULNULNULNAKNULNULNULGSNULNULNULESCNULNULNUL$NULNULNUL(
NULNULNULDC4NULNULNULDC1NULNULNUL:NULNULNUL
NULNULNULGSNULNULNULEMNULNULNULDC3NULNULNULRSNULNULNUL
9 NULNULNULCANNULNULNULCANNULNULNUL2NULNULNULSYNNULNULNULRSNULNULNUL*NULNULNULNULNUL
NULNULNUL<NUL
10 SOHVTSOHDC1SOHCANSOHEMSOHSUBSOHESCSOHFSSOHGSSOHRSSOHUSSOHHSOHiSOHnSOHx9DSOHx9ESOH
x9FSOHxA0SOHxA1SOHxA2SOHxA3SOHxA4SOHxA5SOHqETXrETXxACETXxB1ETXxB2ETXxB3ETXxC2ETX
xD3ETXxE7ETXxE8ETXxE9ETXxFEETXxFFETXEOTEOTENQEOTACKEOTDC3EOTxCEENQxCFENQ
ACK%ACKbACKcACKNAKBSV
11 xB2
12 xB3
13 FF
14 xCA
15 xCB
16 ESOxF6SOxAADC1xE6DC4xF8NAKxB6SYNxB7SYN%NUL$NULDC2SOH
SOHOSOHjSOHoSOHx8BSOHxA6SOHsETXFFEOTDC4EOTwENQ~ENQxADENQxD0ENQxDCENQxEAENQxFEENQCAN
ACKiBSxBFBSW
17 xB4
18 xFB
19 x89VTxCC
20 xD5
21 xF7SOxF4SIxCFDC1x9FDC2>DC3xA9DC4vNAKx87ETBETXCANQCAN
22 NULISOHx99STXxF6ETX&ACKKACKSYNBSxABDC1PNAKxBENAKxF9NAK<SYNxC4SYNxF6SYN,NULFSNULFFSOH
%SOH.SOHUSOH[SOHxAASOHHSTX+ETXx89ETXxADETXxB4ETXxC3ETXxD4ETXxEAETXxEEETXNULEOTBELEOTo
EOT1ENQxBFENQxD5ENQxF9ENQ!ACKdACK_BELxA3BELxEEBELx89
23 xC1
24 ACKVTxECFF
25
26 ?SOFSO<DLExA3DC2xFFDC2xE7DC4~NAKxB8SYN
ETBBSCANNAKCAN#NUL%NUL]NULPSOHkSOHpSOHx93SOHxA7SOHx86STXgETXnETXtETXxE4ETXx95EOTtENQx
ENQxA4ENQxAEENQxD9ENQxDDENQxEBENQEMACKYACKxCFACKxB3BELxC4 yVTAFFx90FFx93FFxCD

xD6
xA0DC22ETBRCAN\CAN NULx81NUL!SOHx8CSOHxD1ENQx8FACKxBFACKxC6ACKxFCACKr O
X
xFC
KVTxDEVTHFFVFFxBAFFxF0FFSTXSI'SIxADSIxF5SI*DC1xBBDC1xFBDC2?DC3xD8DC4_NAKwNAKx87NAKZ
ETBEOTCANRSNULkNULETXSOHqSTX*ACKTBS`BSjBSxE2BSG x84 x93 xCE x83
xB5
xEE
xF4
fVTjVTeFF
SOxF8SOxDASIxB3DLEx8ADC1xD0DC1xEBDC1xAADC4xF7DC4xD1NAKx88ETBSINULDC3SOHzSOHxC7STX
EOTNAKEOT ENQxFFENQUSBELxC0BSx8AVTxA1FF>
}SOx8FSIHETB NULBELSOHJSOHcSOHfSOHVSTX'ACKPACKFSBELx9DBELETXBSxF7BSxE9 ;
xCCVTACKFF$FFxD0
<SOx82SO~SI$DLExCDDLExE3DLE]DC1x84DC3`DC4QNAK=SYNBSYNxF7SYNDLECANxEECANSYNNULNULNUL@
SOHLACK\ACKxACKx81ACKw
xCB
xFB
xC8SOxDDSOETBDLEmDLEx87DLExACDLExC2DLE
DC16DC1WDC1x97DC3LCANUCANEMNULxF7ETXxF5ACKEBELETBBSx97BSl ^
i
xE8
:VTxC4
xD9
xE5SO`DC1xACDC3xE1DC3
DC4xBFNAKxFANAKESYNxC5SYNxFDSYNGSETBxA0ETBx8BCANDC3NULx99SOHx9ASTX~ETXWEOTIENQxF3ENQ
xEBBSxC7
(VTSIFFxB1FFxB3SO*DLExACDC1xD7DC13DC2DLEDC3xEEDC3qETB(NULISTXUSETXxAEETXxEBETXSOHEOT
BEOTxB3EOTxB6EOT{ENQxB6ENQxD6ENQxE7ENQENQACK"ACK9ACKeACKxADACKxA4BEL>
xC2
BELVTSYNVTxEDFFSO
@SOxC1SOxDASO@SIx81SIxA0SIxFDSIxCADLExBCDC3x88DC4x9EDC4xE8DC4 NAK+SYN9SYN
CANFSNULHNULxF4NUL
SOHvSOHxD3SOHxDESTXxEFETXBSEOTpEOTx86EOTNULENQ2ENQxB9ENQ<ACKpACKIBELxEFBEL6 ~
GSOx88SOx9FDLEEDC1RDC2|DC3&DC4cNAKxDASYN
NULGSNUL3NULxD1NULxABSOHxDASOHBSETX?ETXXETX`ETXxB5ETXxBEETXx80EOTxAFEOTxCEEOTxC0ENQ
xE0ACK`BELx8A
nVTxC4VTxCFVTxBBSONSI=DLExF1DLExA4DC2xD9DC2x9CDC3x9DSYNxB9SYNxF4ETBxD8CAN"NUL&SOH/
SOHVSOH\SOHeSTXxADSTXxD2STXESCETX,ETX9ETXx8AETXxC4ETXxD5ETX'EOTxC3EOT:ENQxC5ENQxFAENQ
BSACKxA8ACKxE3FFDC1
NAK
ETBSI"SI4SIxE6DLExB0DC2NULDC3VTDC4
ETBx98ETBSYNCANaCANSUBNUL"NULlSOHxA8SOHACKETX7ETXoETXxE5ETXuENQyENQxDAENQ7ACKZACKSUB
BELxC2 g
xB0
DC4VT&VTx91FFxCE
xD7
|SIxC0DLEPDC2xA1DC2SCANDC2NULWNULx87STXESCEOTxD0ACKxE9ACKETB (x92
$VTzVT]FFxD3FFxD5FFUSSOxDDDC1xC9DC2yDC4BELNAKDC4NULYNUL'STXuETXx96EOT/BELxB4BELxB2BS
x9A x94FFBEL
8
|DLExD6DLE1DC1)DC2GDC3xBFDC3cDC4x8FDC43ETB!NULACKNUL&NUL^NULdNULQSOHqSOHx94SOHjSTXH
ETXhETXGSEOT#EOTxA5ENQxAFENQxDEENQxECENQSUBACKSACKxD2ACKxB7 xC5 BFFRS
!SOxC4SOSISI_SIxBCDLEEOTDC3TNAKxCDNAKxABETB]CAN%NULxF1NUL"SOHx88SOHx8DSOH<EOT?EOT
xD2ENQ0ACKx90ACKxC0ACKxE6ACKMBELxB1 xB4 P
Y
xE0
xE5
LVTx8EVTxAEVTxA5FFxE9FFxAESIxF6SIxDEDLExFBDLESUBDC1x93DC1FFDC2xFCDC2VTDC3@DC3SOHDC4`
NAKxB1NAKENQCANFSNULx82NULkEOTxA4ACK8BELUBELx83BEL/
xFD
IFFWFFx83FFxADFFxBBFFxCAFFxF1FFW
k
ETXSIx84SI+DC1x82DC1xC0DC1xD9DC4xEBDC4x88NAKxE8NAKSOSYNxC0CANDLENULxBCSTXdBELrBEL
xDDBELxA0BSs xD9VTxDFVTN
xF0SO{DC4xNAKx90NAKxDDNAKxC5ETBx97CANDC3NULxD2EOTxC7ACKxFDACKxC7BELxD7BEL'BSxD8BS

EMVTx94VT'FFBELSI(SIIDLExFEDLExBCDC1xC1DC4[ETBxF5CANDLENULEOTSOHxE2SOHxC3ACKSO
BELuBSxB6
gVTkVTESCFFPFF5
t
SYNSOODC4DC3NAKxFASYNCANNULxEBACKZBELxBBBSHx85 x8C x84
xB9

xF5
(
-
EOTSOxF9SOx99SIxDBSIx91DC2x9ADC2xC1DC2xABDC4xD2NAKSYNETB9CANx9CCANEOTEM%NUL1NUL
xCDNULrSTX}STXx89STXxABEOT*ENQjENQ+ACKUBSaBSkBSxA6BSEMP x94 xAC xCF xD7 DLE
GS
xA6
0VT]VTxB7VTfFFxB4DLEwDC1xD1DC1xDFDC1xE0DC2xCBSYNxF1SYN~ETBx89ETBx92ETBxB0ETBETBNUL
xA8STXxD2BSxE3BS* xEF
_FFxD7FFxF5FF
SO@DC1x8BDC1x96DC1x9EDC1xECDC1xF8DC1aDC2xB6DC3xC8DC3xF6DC3xF8DC4
NAK-NAKFFCANDC4NULCANETXxC9ETXDC1ENQ.ENQx93ACKx8BVTx91VTNULFFCANFFRSFFMFFSFFxA2FF
xC3FF\SIoSIxB1DC1FSSYNSUBETB,ETBDC1NULxD5NULxCFSOHNULACK BELxCBBELxC1BSxC6BSDC4
aVT|VT#
?
~SOx8BSIx8CDLE8DC2x82NAKVTNULSOEOTx80ENQx94
LSOaSOxA5SOx90SIx91DLEx81DC2\DC4IETBDLENULPNULDC4SOH{SOHDC4STXxC8STXSYNEOTEOTENQQVT
xA6VTDC2SOxCASI[DLExCBDC2x8BDC4TSYNxE2SYN!NUL1NUL[NULiNULBSSOHMSOHdSOHgSOHxD0STX(ACKI
ACKGSBELSOHBSx91<
xEC
xCDVTcFFx9FFFxD3
xF9
=SO SIxFBSIxE1DLExE4DLE^DC1xB9DC1SODC3x82DC3@SYNCSYNBELETBFETBDC3NULSYNETXSIENQQACK
x81BELxDBBELNAK "VT%FFU
i
r

SINDC2ZDC4aDC4RNAKRSYNxF8SYNx95CANETBNULKSOHx92STXxE3STXx9EBELEOTBSxF8BSX RSVT
VTBELFFx99FFxFCFFxD1
%DLEADLEx8CDC2x85DC3DC2DC4x80DC4>SYNx84CANx86CANx93CANEMNULDC2NUL+NULxC6NULxDCNUL)
STXWSTX\ETX+BSxACBSxDF xEA
x83SOxB9SICDLExCEDLExB7DC2xD5DC3xCCDC4!NAKDC3SYNxD1SYNDC1CANxE3CANxE9CANxEFCANFFNUL
xDFSOHMACKzSODC4DLE'DLExC7DLE7DC1XDC1x8EDC2MDC3&SYNEOTETBETBNULxB8NULASOHxE7SOHx82ACK
xCC
@VTxFFxFEFFx86
xE8
xFC
xDESO-SInDLEx88DLExADDLE+DC2IDC3EOTDC4KDC4SYNNAK>CANBCANSONULx87NULzBELxEABELxA7 x
xC9SOCANDLExC3DLEETXDC1
DC1!DC2x98DC3x82DC4xE1NAKVTNULSOHNUL]ACKyACK1BELxBSxBDVTxA8FFeDC4MCANVCANxA0CANUSNUL
xCBEOT~ACKFBELxA0BELxB6BELxBCBELCANBSZ _
xD1
xE9
xA3VT6FF9FFx89FFx96FF:SI3DC1zDC28DC3jDC3mDC3xD2DC3xFADC3BSDC4x91DC4EOTNAKx8DNAK/ETB
NULCANx88CANDC2NULxEDSOHxF9EOTxA9BEL
VT;VTxA1
xE6SO!DC1xCADC1<DC2XDC3xC1DC3xE2DC3DC4DC4!DC4xC0NAKFSYNxA1ETBSYNNULx8BNULxF8ETX
BSx98BSm w xBC <FFx9BFF:
xBE
xDA
CSIxA3SIaDC1iDC1SIDC2_DC3x93DC3JNAKRSETBx8CCANESCNULSYNENQXENQxF6ACKABELACKBS~BS] j
xD4
xC5
xB7SOxC5SI
DLE.DLERDLExD8DLEoDC2PDC3xADDC3xDFDC4SINAKxFBNAKx96SYNxC6SYNxFESYN;ETBPETBDC1NUL
x9ASOHFFACKwBELrBSxFABSx9C S
xC8
FFlFF
SISOSSOxB4SO+DLEx89DC2x90DC3DLENULxC6SOHEOTSTXx9BSTXXEOTx98EOTx82BSsFF/SOxSIxD8DC1
x87DC3xFDDC3?DC4qDC4xA1DC4xA7SYNDC4NULxA8NULxACNULxB0NULxB4NULwETXxA5EOTxF4ENQxECBS
x9F
xB1VTDLEFFxB2FFeSOwDLExADDC14DC2fDC2-DC3xC6NAKrETBDC3NUL5SOHxAFSOHxB8SOHx94STXxE5STX
"ETX ETXxDFETXJENQ=BELxB4BS)VT6VTxE2SI~DLEDC1DC3xEFDC35ETBxDCCAN)NULFSOHSSOHFSTX)ETX^
ETXxAFETXxECETXSTXEOT%EOT~EOTx93EOTxB4EOT|ENQxB7ENQxD7ENQxE8ENQxF1ENQACKACKRSACK#ACK
:ACKWACK\
xC5
x8EFFxEEFFSI
CSOxBFSOxC6SOxDBSOxCBDLE[DC1xDFDC3x86DC4xCFNAK)SYN7SYN:SYNDC3CAN_CANDC3NULiEOT5ACK
xC5BELN xE3
ETBVTOVTx81FFxC2SOx82SIxBEDLE_DC2xC7DC2xBDDC3x89DC4xE9DC4x80NAKxC3ETB
CANSONULDLENULxE5SOH ETXDC4ENQxA7BELDC3
BSVT8SIASIxA1SIxF9SIx9FDC4HNAKPSYN+NULJSTXQSTXxFDSTX'ETXx9CETXCEOTxB7EOT`ENQbENQdENQ
x8FENQxE0ENQxE2ENQFSACK3ACKUACKfACKxAEACKxA5BELxBFBELxE1 ?

```
xC3
x8CFFASOx8CSOxADSOxBBSIxFESIbDLEDLEDC1}DC1ACKDC3cDC3xB4DC4NULSYNUSSYN,SYN5SYNKSYNe
SYNmSYNxF8ETB/NUL(NULINULfNULxE3NULxF5NULSOSOH+SOH=SOHsSOHwSOHx82SOHx85SOHx90SOH
x96SOHdETXx90ETX
EOTSOHENQ3ENQx91ENQxA1ENQSIACK=ACK@ACKCACKJBEL~BELxB9BELxF7BELETXFFFFFF
R
x89SOxFFSOxA0DLExA3DLExB9DLEqDC1tDC1 DC1BSDC3SIDC4'DC4xBEDC4MSYNxEBCAN&NULDNULLNUL
xDENULxD4SOH+STXYSTXxC3STXxDFSTXjETXREOTqEOTxBAENQhACKxD4ACKESCBS-BS7
xC6FFx96
xE4
sSODC1SIFSSIFDC1mDC1xBCDC2xE9DC2SYNDC3eDC3xD7DC3VNAKdNAKnNAKxFAETBeCANxD0CANxE5CANSI
NUL-NULxE6NULxF4SOHqACKxDAACKxF0BEL   xB1
HSOaSI}DC3/DC45NAKxB4NAK.SYNDC3NUL7NULUSSTX1STXxF0ETXx87EOTx8FEOTCENQxB1ENQDC1BEL
xCDBS
DFFYSOEDLESDC2*DC4#NAKSTXSYNxDBSYN"NUL4NULxD2NULxACSOH@ETXYETXaETXxBFETXxB0EOTxCFEOT
xEEENQxBCACKxCCACK,BELaBELxB9 xE3 xE6 ETXVTxD6VTx93
xAASOxBCSO=SIDC1DLE9DLE>DLE_DLEETBDC1TDC1xB9DC2SOHNAKxADETBxF5ETBxD9CANDC1NULDC4NUL
xDBSOHxD8STXyEOTxE1ACKPBELx9E
oVTxC5VTw
xD0DLExF2DLECDC3vDC3XSYNUSCAN1CANSYNNULETXJETXaEOTx81EOTlACKENQBEL5
x82VTEM
`
}
x96SOxD3SINULDLEEDC2xA5DC2x9DDC3RDC4|SYNx9ESYNxD3SYNxDAETB)NULBSNULRSNULxC8NULxB6ETX
xBDEOTENQ%ENQ6ENQxC1ENQxE2BEL6BSxFDBSA
x8B
xD0VTxEAVT3SOx8ESOx9ESOxAFSOxCFSOOSIrSIxB1SIxBDSIVTDLE2DLEDC2DC1:DC1xB4DC1xD2DC2
xDADC2x8CDC3xA7DC3xE6DC3x94DC4xCEDC4sSYNxACSYNxBASYNxACCAN!NULSSTXbSTXxFASTXFSETX9
EOT^EOTxC8EOTxF6EOTx8CENQx94ENQxE4ENQxFBENQ   ACKFACKx9ABEL!FF0FF3FFxF9FFDC2
SYN
VSOx85SO5SIxD0SIxF8DC2SOHDC3;DC3FFDC4kNAKx9ASYNx8FETBbCAN'NUL'SOHVTSTXx9EETXxC5ETXJ
EOTxB9EOT;ENQx97ENQxA7ENQDC2ACKx87ACKx96ACKxB0ACKxAEBS$   xEC STX
b
s
oFFxE4FFCANSI#SIx99DLExA6DLEGSDC1xF4DC2TDC3pDC3DDC4xB6DC4NAKSYNaSYNx91SYNVTETBxE5ETB
xECETB'CANxF1CANSUBNUL<NUL`NULxA2NULxFFNULxB4SOH:STXxFFSTX:ETXOETXfENQxC6ENQxFABEL
xF0BSSTX   SI   xC7
XVTxAAVT#SOxE6SIdDLExE7DLE}DC2"DC3x99ETBETBCAN1NULCANNULxF8NUL0SOHWSOH]SOH2STX6STX>
STXBSTXLSTXfSTXxA4STXxAESTXxD3STXxEBSTXxF0STXxF5STX-ETX3ETXCETXSETXx8BETXx93ETXxCCETX
xD6ETXUSEOT(EOTEEOTxA0EOTxC4EOTxE4EOTESCENQ!ENQOENQpENQxA9ACKxF0ACKxC1BELEMSOTSIWDLE
xB1DC2x9ADC4!SYNgSYNoSYNDC1ETB%ETB+CAN$NUL#NULDLESOH$SOH-SOHmSOHx8ASOHxA9SOHpETX
x88ETXxD2ETXxE6ETXVTEOTnEOTvENQxCDENQxD4ENQxDBENQxF8ENQDC4BShBSxBEBSxB1
xEBFFxC9
xF5SOxCEDC1xA2DC2xFEDC2uNAK}NAKxBDNAKxF7NAKxC3SYNxF5SYNx86ETBBELCANCANNULySOHx92SOH
x85STXxC6STXfETXmETXxE3ETXxA3ENQxC3   xCD N
x82
eVTx92FFBSSO|SOSOHSI&SIx8ESIxB2DLEx89DC1xEADC1xF6DC4^NAKSUBNUL?SOHx98SOHHENQOACK[ACK
ESCBELxF6BSh
v
xE7
'VTxCBVTENQFFSOFFxB0FFxC3
xCF
xD8
x81SO}SISYNDLE)DLExC1DLEKCANTCANxEDCANUSNULGNULxF3NULuSOHxD2SOHxACSTXBELETX8ETXAEOT
xFFEOTzENQxB5ENQ8ACKxA7ACKxDFACK}
NAKVTxC3VTxE2FFxD9SOSYNSI!SI3SIMSIx9FSIxC9DLEQDC2{DC3xBBDC3bNAKxF3ETBxD7CANSYNNULX
NUL&STXENQETXFSEOTxD1ACKxE8ACKxB1BS'   xB6 f
DC3VT%VT\FFxD2FFxD4FFACK
 SO{SIxBBDLExD5DLE0DC1FDC3CANNULxF0NULx87SOHxBBSTX>EOTxA3ACKLBELqBEL      xB3
 xC9FFxE8FFM
xEFSOACKSIxFADLExFDDLEx81DC1x92DC1
DC3NULDC4zDC4xC0DC4xE7NAKxF4CANETBNULxE1SOHx88STX)ENQxC2ACKxEAACK
BELCAN  )   xAB xD6 ^FFxD6FFxF4FF,
x98SIvDC1x95DC1xDEDC1xF7DC1x90DC2xDFDC2NDC4BSNAKSONULONULDC3STXxC8ETXETXENQx92ACK
xC5BSx93
{VTx90VTLFF"
x8BDLExCADC2EMETBETBNUL*NULZNULhNUL(STXxE2STXx80BELW   xDE T
xE0DLExB8DC1MDC2xB6DC2
DC3DC1DC4 DC4xCBDC4DC2SYNxD0SYNACKETBEETBxE2CANxE8CANDC2NULx86NULxDESOH0BELxE9BEL
xBCVTwFFxA7FFxE7
STXDC1 DC2*DC2HDC3LDC3ETXDC4dDC4=CANACANx9FCANETBNULxECSOHWENQxB5BELxBBBELxD0
x88FFx95FF9
xA0
```

USDLExD7DLE
DC12DC1SODC2;DC2yDC2ODC3WDC3^DC3xC0DC3BELDC4x90DC4SONAKDC2NULxA7NULETXSTXvETXxDEETXx97EOTqBSxB3BSx9B R
xB0VTrFFBS
.SOwSI}DLE,DC3pDC44ETBSUBNULRSOH$EOT}EOTxDFENQESCACK2ACKTACK[
xE2
NVTx8BSOxC5SOxF8SIxBDDLEZDC1^DC2ENQDC3xDEDC3GNAKxCENAK(SYN4SYNOSYNdSYNlSYN^CAN#NUL'NULKNULeNULxE2NULxE5NUL*SOHrSOHx81SOHx84SOHx8FSOHx95SOHkSTXiETXxB0ENQ?ACKBACKxD3ACKBS
CFFUS
xE3
DLESIESCSI`SIxA2DLExB8DLEpDC1sDC1xE8DC2)DC4xBDDC44NAKUNAKxB3NAKxCFCANEMNULBELNULIETXxBCEOTBSENQ5ENQxEDENQkACKOBEL5BSxB8 x9D
x81VTx92
2SOxCESOxB0SI
DLE8DLE9DC1BDC3xA6DC3xE5DC3xABSYNxACETB0CANCANNULETBNUL;NUL_NULxA1NULxF7NULxA3STX2ETXx92ETXRSEOT8EOTx93ENQxA6ENQDC1ACKEACKx86ACKxF9BELxC6"SOVDLExA5DLEFSDC1xF3DC2xF7DC2*CAN)NULFNULxF2NUL#SOHx89SOHx84STXxC5STXlETXx87ETX@EOTmEOTxCCENQxD3ENQxA6ACKxDEACKDC3BSxF5BSM
x81
xE6
dVTxC2VTxAFFFxE1FFxEAFFBELSOxD8SONAKSISI2SIx88DC1xCDDC1xE9DC1xFDDC2xBADC3xF5DC4aNAKtNAK|NAKx85ETBACKCANJCANESCNULNNUL=EOTx91ACKxA2ACKxC1ACKxE7ACKxC4BSxB2 xB5 xD5 e
DC2VTx8FVTKFF[FFxC8FFxE7FFxF3FF+
ENQSIx97SIx8ADLExFCDLE/DC1x91DC1x94DC1MDC4GSNULx85NULxA6NULxEBSOHxE1STXV xDD Q
xCF
xAFVTxBBVTvFFx87FFxA6FFx9F
xE6
vSIxDFDLE
DC2USDC2FFDC3KDC3STXDC4ACKDC4oDC4xCADC4DC1SYN<CAN@CANxE7CANESCNULxA0NULxE1NULx80SOHx8ESOH|EOT1ACKjACKx85ACKNBEL4BSBEL
Z
xE1
MVTx91
xE2
xAFSIxF7SI7DLEESCDC1oDC1ADC3xDDDC3xB2NAK3SYNxAASYN/CAN&NULx84NULx9FNULxE0NULxEASOHx86ETXlEOTx84ACKxA1ACKxA5ACKDC2BS3BSxF4BSxDC ACK
xCE
DC1VTxC1VTJFFZFFx86FFxAEFFxE0FFxF2FFxE1
xD7SOEOTSIDC4SIUSSI1SIx96SI.DC1x87DC1xE8DC1RSDC2xDCDC3xC9DC4sNAKDLESYNRNULqNULx83NULx9ENULxE9SOH-STXx99ACKx9AACKx84BELDLEBSDC1BSGSBSRSBSUSBS/BS0BS1BS2BSDBSEBSFBS[BSENQ
xFE
XFFYFFx84FFx85FFxBCFFxCBFFxCCFFX
Y
Z
[
xEA
xFE
DC3SIRSSI/SI0SIhSIiSI,DC1-DC1x83DC1x84DC1x85DC1x86DC1xC1DC1xE6DC1xE7DC1ACKDC2BELDC2xD9DC3xDADC3xDBDC3xC8DC4xDADC4xECDC4XNAKYNAKfNAKpNAKqNAKrNAKxE9NAKxEANAKSISYNxE8SYNxE9SYNxEASYNxA4ETBxA5ETBxA6ETBxB8ETBxB9ETBxCFETBjCANxB6CANxBBCANxC5CANxD2CANGSNULx95NULx9ESTXxB2STX9BELjBEL BSGBS= ETB
0
xCDFF\
l
xF5
xFF
jSISOHDC2BSDC2DC4DC2gNAKx89NAKxEBNAKxF3NAKxB1SYNxA7ETBxBAETBxD0ETBkCANxD3CAN"NULrNULxD8NUL.STXSIETX.EOT2EOTx9BACKVBELx85BEL\BSxBC
xFF
BVTxBDFFB
x9B
xEB
x85SIHDC1LDC1xC2DC1-DC2xEEDC2xDBDC4xEDDC4ZNAKxEFNAKxEBSYNUETBxB7CANxBCCANxC1CANxC6CANxCACANDC3NULCSOH[EOTxAAENQNAKACK:BELxD4BELx9DBSt xA4 O
]
xF6
DC2BDC2xBEDC2hNAKx8ANAKxDENAKxA8ETBSYNNULx96NULeBELxA1BSCAN
x98
xE0SOxEBDC2[DC3|DC4xA4DC4xAEDC4yNAKx91NAKxA8NAKxECNAKxF4NAKBETBxD1ETBDCANgCANlCANxD4CANSYNNULvNULxD6SOHx9FSTXsEOTSOHBELx90BELxDEBEL!BSHBSxC9BS&
1
xDAVTNUL

m
xAD
STXDC24DC33DC4xBBETBxC6ETBxE0ETBDC3NULxB3STXxBDSTXkBELsBEL<BS9 >
VTFVTxE0VTxCEFFNULSOxF1SOkSINAKDC2EMDC2IDC2xB2SYNx98CANDC3NULxD9NUL/STXTEOTxBCENQW
BELxC8BELxD8BEL(BS]BSNULVTUVTxFCSOxBDDC1gDC3xB3DC3xDCDC4xC0ETBxD7ETBxBDCAN
NULsNULxBANULxA6ETX/EOTxDDEOTx9CACKxFEACKxE6BELCVTx95VTxEFVTxF7VTzFFx9C
xA4
xA7
xAA
BSSIxAFDLExFFDLEIDC1[NAKxF0NAK\ETB_ETBxB5ETBxCCETBxA5CANxB8CANxC2CANxC7CANxF6CANEM
NULxD3EOTxD8EOTx87ENQxD6ACKx86BELxD9BSVT SUBVT(FFxBEFFI
uSOpDLExEBDLExC3DC1.DC2x94DC2CANDC3xC2DC4xEEDC4)NAKxECSYNVETBxFCETBxCBCANNAKNUL[STX
DLEETX3EOT>ENQxC8ACKxEF xBD
}FFC
x88
xEC
)SO)SIx86SIJDLEx82DLEMDC1xEFDC2kDC4CANNAKfETBSINULENQSOHxABENQSYNACKx8DACKxC4ACK]BEL
xECBELRBSx87
hVTlVTx9ADC3RSDC4$DC4xFBSYNSYNNULUNULxE4ACKSBELxD5BELx9EBSxD6BSxB7
.VTFSFFQFF&
6
JSO SOxDBDC1
DC2'DC2xBFDC2xC6DC3xCBNAKxA9ETB7CANDC1NUL/NULx89NUL;BELvBSu x8F xA5 4VT>VTxBC
xF7
VTSIxC5DLExD0DC3DC4NAKx8BNAKxDFNAKDC1NULDSOHxE3SOH\EOTSIBEL|BELx98BEL" P
^
u
ETBSOqSOCDC2BDC4PDC4x84DC4iNAKSINULxBCBSBELxA9
VTKSIBSDLEwDC2\DC3}DC4xBBDC4xF5NAKETBETBCETBx9DCANxD5CANSYNNULx9CNULx84ETXx9FACK
xDCACKxD2BELxBA
xDEFF)
x8F
x99
ENQSOxFASOx94SIx92DC2xECDC21DC4xACDC4zNAKxEDNAKx83ETB:CANhCAN
NULx97NULNAKBEL[BELx86 x8D x85
#DC2%DC2xA9NAKxD7NAKxD2ETBECANmCANDC3NULxF0SOHxECACKfBELxA2BSI EM
xF6
.
xE1SOx9ASIxDCSIx9BDC2xC2DC2xA5DC4xAFDC4x92NAKx98NAKxD3NAKxA8CANDC4NULxCENULxD7SOH
xACEOT+ENQSTXBELxDFBELxCABSDC1
2
^VTxDBVT2
z
xAE
5DC3sDC3xD5DC4xAENAKxC3NAKx9DETBCANNULx99NULxA0STXxB8STXIBSLBSbBSxD0 xA7
1VTxB8VTgFFx8C
xB5DLExDC1xE0DC1ETXDC21DC3xABNAK0SYNxF2SYNxD4ETB"CANGCAN{CAN+NULmNULwNULxC2NULsSTX~
STXx8ASTX,ACKxACBELxCEBELxF2BEL"BSlBSxA7BS0 Q x88 x95 xAD RS
'
xD9
e
n
x82
xB7
ENQDC1xD2DC1jDC2xE1DC2xB6NAKxD9NAKxE3NAKACKSYNxCCSYN ETBx8AETBx93ETBxB1ETBxBCETB
xC7ETBxE1ETBuCANxF9CANCANNULx91NULxE8NULxC9SOHtEOTkENQsACKxB3ACKx91BELVBSSUB B
b xD8 xF5 xA1
tVTSOH
xB3
xA2DC34DC4tDC4vETBxE8ETBxEFETBDC2NULETBBELtBELxB0BELxA0VT`FFxF6FFSOHSOPSOlSItDLE
x96DLE DC3xF7DC3xB3SYNSOETB
CANx99CANSOHEMGSNULSOSTXxA9STXxBESTX#BELlBEL=BSOBSxD3BS: ? g z
x9A
SOVTxC8VTxCFFFnSOxCBSOxF2SOcSIxE3DC1SYNDC2JDC2xE5DC2xB7DC3ETBDC4xD2DC4xBANAKxB3CAN
CANNULxF6SOHx87BSx8CBSxE4BS"
o
xF0
xE5VTxFAVTSUBDLEFFDC1x8CDC1x9FDC1xEDDC1SUBDC2YDC2bDC2xACDC2xF9DC4.NAK7NAKlETBoCANx80
CANDC4NULxB4STXxA1ETXxB8ACK@BS+
GVTxE1VTxD8FFVTSOxE9SOiDLEADC1x97DC1xF9DC1sDC2RSDC3(DC3xC9DC3GDC4
NAKDC3NULaSOHEMETXUEOT/ENQxBDENQETXACK^BSj
FFFTFFxE3SOxEFDLExA8DC1x9DDC2xA7DC4x85NAKxD8SYNESCETBOCANDC1NULXBELxC9BELxA4BSESC
-

x8CVTx92VTEMFFNFFxA3FF]SIGDLEx8FDLExBEDC1x98DC2xB4DC3xBECAN
NULxDANULxCESTXxD9BEL)BS|BSjFFxC8DC1hDC3xDDDC4x94SYNSTXETB-ETB9ETBGSNULSONULxF2SOH0
STXxC1STXxCAETXx91EOTx9EEOTDC2ENOx94ACKxEEACK*BELSOHVTVVTSOHFFUSFFxC4FFxEDSOpSISIDLE
{DC1xB2DC1xD0DC2x8ADC3GSSYNISYNxC1ETBxD8ETB%CANxAACANDC3NUL9NULxEENULxD0SOHDC1STX$
STXxDCETXxB3ENOVTBELoBELxE7BEL SOxEDSOx8CSIxB0DLExD3DLENULDC19DC2yDC3\NAKRSNULtNUL
xD6NUL0EOTxFFACKhBELxC2BSxC7BSxD3 NAK
DVTbVTxBFVT{FF@
G
x9D
xA5
xA8
xAB
xF3
fSIJDC1xF1NAK]ETBxB6ETBxCDETBxDEETBxB9CANxC3CANxC8CANDC3NULx9DACKDC3BEL!BELxCCBEL
x85BSx98
rVT}VTxF8VT$
0

SIxC4DC3xB9DC4xD5NAKEOTSYN~CANxA6CANxF7CANDC1NULxBBNULxCCSTXxA7ETXMEOTxDEEOTSOH
ACKzBS~ +
xAA
x96VTxF0VTx8DDLExF5DLEx83NAK[SYN`ETBDC4NULnSTX{ACKxD7ACKxCFBSxE8BSFF K x8F
ESCVTx86VTbSOxA2SOx9CDLExA9DLE,DC4<DC4]DC4*NAKWETBxFDETBFFNULEENO{ x95
J
MSOx91SIqDLExECDLExF7DLEx95DC2xA2CANxCCCANSINULxBDNULx81ENO&BEL3BELx98VT)FF[SOx92DLE
/DC2EMDC3xC3DC4>NAK]SYNxEDSYNXCANETBNULESCSTXSIEOTxD4EOTxD9EOTx88ENOx87BELxDABSx80
VT
xBFFFvSOxA6SOxD3SOxC4DC1x82DC2SUBDC4gDC4xEFDC4GSNAKx94NAKx9ANAKJETBbETBEMNULETXNUL
NAKSOHxDBSTXxA9ETXOEOT_ACKxC9ACKxFEBELxBE
xF8
xA7VT~FFxAAFFxB7FFDC3SO*SIxD7SI\DLEQDC1xC4DC2xD6DC2x8CDC4xB1DC4xFEDC4USYNDC2NUL!STX
xC9STXx9BEOTxFCEOTENOENOx8AACKeBSRVTD
x89
x9CSINDC1?DC2xA9DC2xF0DC2lDC4DNAK4CANSINULxFCSOHBELSTXNAKSTX\STXETBEOTxEBEOTx9AENO
x90BSxED
xDESI$DC1xF3DC1UDC2CANSYNxDDSYNSYNNULQNUL|SOHxC0SOHDC1ETXxF2ETX4EOTx89EOTxE0EOT?ENO
xF0 xAC
xF2VT*SOx87SIxCBSIKDLEx83DLExCCDC2EMNAK%NAKxE3SYNgETBDC3NUL
SOHNSOHhSOHxC1ETXxACENOETBACKJACK^BELxA2BELxEDBELx88
ENOVTx88VTxD4
>SO;DLEONAKBSETBSTXCAN
NUL\NULjNULx80NULSTXSOHpSTX)ACKx8EACKxBEACKxC5ACKxCEACKxFBACKRSBELSBSxE1BSqx92 xED
xF3
iVTxVTxDDVT@FFdFFxA0FF=
xACSIxBADC1xD7DC41ETBGETBYETB[CANESCNULACKSOHeSOHx80ACKxF4ACKSTXBSxEABSxE8 :
xFA
xABDLExE2DLEBSDC15DC1VDC1_DC1xD6DC12DC2SIDC3x83DC3x96DC3USDC4_DC4ASYNDSYNxFCSYN
x9FETBx8ACANCANNUL2NULxD0NULxD9SOHxD1STXWETXxBDETXxAEEOTxB2EOTxCDEOToACKHBEL5 =
mVTxCEVT?SIx80SIxFCSIx9EDLExE5DLExAFDC2x9BDC3
DC4%DC4SYNNULVNULcNULSUBEOTRACK.BELSYN xC1 x91
#VT7
RSSOSOSI{DLExDCDC1(DC2ODC2bDC4xDC4ACKNAKSNAKxCCNAKxAAETBEMNULxD1EOT/ACKxE5ACKTBELc
BELx82BELxD6BELxDCBEL&BSx9FBSxD7BSxB0 xDF
xADVTxD8VT&FFV
j
xDDDLEEMDC1VTDC2x8FNAKxB0NAKxDCNAKx96CANSUBNULxCCNUL|STXxAAEOTiENOxD1BSx8B SI
xA5
xB8
/VT\VTxB6VT'
4
s
?DC1x9DDC1xC0DC2xC7DC3,NAKxF0SYNxF9SYNNAKETB}ETBxAFETB8CANNAKNULxD4NULxCESOHETBETX
DLEENO-ENODC3
`VTxA5VTGSFFRFFKSO`SOxA4SO[SIxB0DC17DC2x80DC2[DC4ESCSYNSSYN+ETBESCNUL0NULxC5NULLSOH
NAKETX[ETXxABBSx90 xEB
GSVTUSVT!VTx98FFx9EFFh
q
xD2
xF8
FFSI@DLEBDLExD4DC3YDC4?SYNx83CANx85CANx92CANx94CANSONULxB7NULwBSxA6 ?VTxFDFFx85
DC3DLE&DLExC6DLEx8DDC2x81DC4NAKNAKxE0NAK%SYNCANNULx8ANUL}ACKx9FBELENOBSY \
v xBB xD3
8FF;FFx9AFFxBD

```
xC4SIhDC17DC31DC3xD1DC3DC3DC4ETXNAKx8CNAKOETBxFFETBx87CANDC4NULxABNULxAFNULxB3NUL
xAESOHxB7SOHxC5SOHx93STXxE4STX<BELxF9BS5VTBSFFdSOxE1SIeDC2x86DC3xFCDC3>DC4xC5NAK
xDBCANNAKNULESOHxE4SOH]ETXx9BETXhEOT_ENQxF0ENQxBEBELDC2     M    xE0
x8BFFxACSOxBESOxBASIaDLESIDC1bDC3x85DC4xF7ETBDC2CANFSNUL,NUL6NULxDDNUL<SOH*STXXSTXc
ETXx8FETXxD9ACKDLEBEL}BELxB8BELSUBBS,BSxCCBSQ
x95
xB0
rSODDLE1DC1xBBDC2NAKDC3xD6DC3.DC4"NAKxE4CANxEACANNAKNULDC3NULxC7NULxD7STXEOTBEL
xE1BELxE5   4
xE9VT_
v
|
<SIxCFDLESYNDC1DDC2xB8DC2uDC3QDC4x93DC4xCDDC4xD2SYNFSNUL
STX=STXBETXRETX]EOTx99BELxADBSxEFBSSOH  SO   #    xEB  a
r
CANSOx84SOxCFSI|DC2:DC3oDC3CDC4x99DC4jNAKDC4SYN`SYNx8EETBxE4ETBxF0CAN
NULGENQxF7ENQNACKxBDBS{SOLSINAKDLE(DLExC8DLExF6NAKxC2SYNxF4SYNxD6CANFFNULxE0SOH
xBASTXBS    xAA  ENQ
L
xF9DLExF6DC1x8FDC2EDC3xE6NAKCANETB
NULxDDSOHSTXSTXxDC2NDC3]DC3~DC4xCFSYNENQETBDETBx9ECANFFNULSYNNULxA2STXx80VT
DLExB7DLE8DC1YDC1xE4DC3xBCDC4'SYNkSYNxCECANNAKNULxA5NULx83STX{EOTxDDACKU   L
xBAVT*
x90
ACKSOx89DLExCCDC1JDC3ENQDC4LDC4{NAK2SYNx84ETB;CAN?CANICANGSNULpNULx9DNULxE8SOHx85ETX
x83ACKxA0ACKZBSxF3BSxBB
xCD
AVTxDFFFx9A
xE0
xE9
xFD
.SIx95SINULDC2ENQDC2DC3DC2GSDC2,DC2xEDDC2xC7DC4xEENAKxB0SYNxA3ETBiCANSINULBSOHxD3BEL
x9CBSxA3   %
FFVTxFFFFxDFSOHDC23DC3ZDC32DC4xADDC4AETBCCANDLENULxB9NULx86ENQyFFx87
xA3
xFBSOoDLExAEDLExEADLEx93DC2ETBNAKxB4ETBxBFETBxCBETBxD6ETBxA4CANDC3NULx88NULxE3ACKR
BEL\BEL{BELx97BELxEBBELx8E   x86
-VT3VT=VTxBB
^SOxC4DLE&DC2x99DC3#DC4x83DC4   NULx9BNULxEFSOHxD1BELxA8    x8E
x93SI"DC2$DC2x82ETBSUBNULx90NULx98NULxC1NULxABBELKBS/  x87 xF4 xA0
d
y
x81
EOTDC1iDC20DC3xAANAKxADNAKxC2NAKxD8NAKxE2NAKuETBx9CETBxD3ETB!CANFCANtCANEMNULxB7ACK
SYNBELxAFBELNBS!
y
xC7VTOSOmSOxCASOxE8SOEMDLEhDLEsDLEx95DLEVTDC1xE2DC1XDC2xE4DC2GSDC3SYNDC4xB9NAKkETBn
CANNULEMDC3NUL
NULxF1SOHxEDACK)BELxA3BSSUB
iFFxE2SOxEEDLEzDC1xA7DC1x97DC2x9CDC2xA6DC4HSYNxD7SYN$CANNCANxA9CANDC4NUL
BELgBELyBS} xD2  *
x97
xA9
qVTxBEVT/
xF2
xD2DLExF4DLExDC3xC3DC3xD4NAKZSYNxDDETB}CAN
NULzACK2BELxF7BSJ   z
x85VT;DC4fDC4=NAKx93NAKx99NAKWCANxA1CANNAKNULSTXNULxDASTXxEAEOTxFBEOT^ACKxFDBELdBS
x8FBSxF7
xA9FFxB6FFx9BSIxD6SIxDDSI#DC1>DC2xA8DC2xC3DC2xB0DC4xFDDC43CANSUBNUL NULxCFNULxD8SOH
xADEOTxCCEOTnACK ACKGBELxA1BEL4 p    xDCVT?FF<
BELDC14DC1xD5DC11DC2x95DC3
DC4xD6DC40ETBx9EETBSOHCANZCANx89CANEMNULxCDSOH{STX,ENQ.ACK%BSx8A    xAF  DC2
xDE
[VT_VTxA4VT3
GSSOzDLExDCDLE>DC1x9CDC1ENQNAKx8ENAKxAFNAKxDBNAKxEFSYN*ETB|ETBDC4NULxC4NULxAABS[
xD2
xEA
7FF:FFx97FFx9DFFg
p
x84
gDC16DC3kDC3xD3DC3xFBDC3xC4NAKNETBx91CANEMNUL^ENQETXBELxB7BELxBDBELxE0BELEMBSxCBBS3
`
```

```
xE8VTx8AFF{
xAF
;SIkDC1{DC29DC3aDC3nDC3tDC3x92DC4x98DC4_SYNx8DETBxE3ETBBSNULxA1STXxB9STXxB6DLExE3DC3
xE5NAKxC1SYNxCESYNxF3SYNEMNULoNULx82STXx9BBST xA2 VTVTxB9VTxA2
xDF
xCBDC1xFFDC1EOTDC2DC2DC2GDC22DC3YDC3xC6DC41SYN@ETBxA2ETBxB3ETBxBEETBxCAETBxD5ETBHCAN
NULx8FNULx9ANULxC0NULxEESOHx96BELxAABELxAEBELxD0BELJBSMBS
2VT<VTc
x80
x8D
xBA
]SOlSOxE7SOgDLEx94DLExE1DC1xE3DC2FSDC3NAKDC4"DC4xACNAKxB8NAKxC1NAKx81ETBsCANSUBNULFF
NULxFAEOT BEL(BELcBSy xD1 )
xA8
x84VThFFxB5FFxD5SI"DC1yDC1=DC2xA7DC2xC2DC3:DC4xFCDC4<NAKGSYNxD6SYNxDCETB#CAN|CANRS
NUL~NULxC3NUL]ENO-ACK$BSxA9BS3  o   x89 xAE xDD
>FFx9CFF;
f
o
x83
ACKDC1fDC1jDC1x9BDC1xD4DC1`DC3x94DC3xDANAK)ETB{ETBx8CETBxE2ETBx90CAN#NULnNULx8ENUL
xBFNULx81STXBSBELx95BELxADBELxCFBELx9ABSS   x   US
(
b

xB9
xDE
kSOxFEDC1DC1DC2xE2DC2ESCDC3xC5DC4;NAKxB7NAKxE4NAKxC0SYNxCDSYNxD5SYN?ETBx80ETBxB2ETB
xBDETBxC9ETBrCAN7NULxNULyNULzNUL{NUL|NUL}NULx8CNULx8DNUL STXx80STXx8BSTXx8CSTXx8DSTX
xF9ETXcEOT\ENOBELBEL#BSx99BSxA8BS1    2   R   n   xDA
xDB
xDC
=FFxB8
xDB
xDC
xDD
DSIESIbDC1cDC1dDC1eDC1xA2DC1xD3DC1xFDDC1DLEDC2TDC4:NAKxA2NAKxA3NAKUSETB
ETBzETBx8BETBxC8ETBvCANx8DCANx8ECANx8FCANGSNULtSTXx8ESTXxFAETXdEOTxF3BELVTBSmBS
x96   xBD xBF
FSIxA4SIxE9SISTXDLExA3DC1kDC2UDC4@NAKKNAKxA4NAKBELSYNxSYN~SYNx8BSYNxA0SYN!ETBx94ETBw
CANxFACANRSNULx8FSTXLETXxFBETXx83EOT5BELBBELxF4BELBELBS BSxB8BSx97    xFF xFDVTESC
xB8SOxF1SI!DLE/DLE'DC1lDC2QDC3xEBDC3xE3DC4DLENAKySYNx97SYN"ETBx95ETBxE9ETBFSCANCAN
NULx92NULxBDSOHCANSTXSTXETXx8DBELnBSxCA xD9 x9DVTxB4
xC0
xC6
&SOSDLExF0DC1pDC2x9FDC3xA3DC31NAKANAKxA5NAKxFFSYNxF0ETBxCAN#NULxE9NULxCASOHxFFSOHuSTX
xSTXeEOTYENOx83ENOxB4ACKx92BELWBSESC xBE xF6 xFC 7
xA2
uVTx9AVTSTX
hSOxEASIETXDLExD9DLExA4DC15DC4VDC4uDC4xE0DC4LNAK SYNx8CSYN<ETBwETBxFBCANGSNULVTETXu
EOTETBENOlENOtACKxF7ACKFFBSC    ^   c   k
xD5
DC3FF+FF8SOx92SOx98SOGSIxA5SIxC6SIxAEDC3x9ENAKxFCNAKBSSYNx82SYNx87SYNxA1SYNxC7SYNQ
ETBCANNULx9BSOH=ETXxFCETXDC1EOTx84EOT8ENOxC3ENOCBELxB1BELx82    T
xC9


xB1SOxB9SOxF2SI"DLE(DC1xECDC3xE4DC4xB4SYNoETBx96ETBSOCANDLENUL
NULCANBEL6BELsBSxB9BSx98    xFEVTxC1FFFS
STXSODLESOmSIDC1NAKxE0SYNx9ACANSTXEMNAKNULx90STXuBELxBELBSBSx80BSx9D
xA1VTaFFQSOxSOxB5SOxB7SI,DLEuDLEmDC2x8ADC2x80DC3x8EDC3x91DC3xF8DC3USNAKEMNULGSSTXM
ETXx8DEOTx8AENO
ACKxF5BELxFBBSRS    NUL

FFmFFxF7FFTSOx9CSOxA8SO0DLEx97DLERDC3zSYNx8FSYNx98SYNSIETB#ETBxEAETBGSCANSUBNUL
xAASTXETXETX'ENOoBSxCB {
x9B
xC9VTxD0FFxC1
xC7
0SOxCCSOxF3SOySIGSDLETDLExB6DC1KDC2\DC2xE6DC2xA4DC3xFEDC32NAKxBBNAKxF1ETBEMNULx93NUL
x9CSTXYEOTxD6EOTxDBEOTx8EBELxE4BEL;   xDA
SIVTtFF'SOxD5SOtSIx8FDC1xE4DC1ETBDC2xB8DC3iDC4xA2DC4xF3DC4xA6NAKxA8SYNdETBxB4CANETB
NULxC7SOH>BSPBSx8ABSxD4BSENO   @   x9EVTxDCFFxB5
```

oSOACKDLExD9DC1qDC2xA0DC3FSDC4@DC4rDC4xD0DC4xD3DC4x96NAKxC9NAKyCANCANNULxECNULxBESOH
ENQSTXSISTXEMSTXxBFSTXxDAETXx99EOT$BELmBELx83BSh x8D
xA0SOxD1SOdSI
DLExC6DC1xF1DC1xD4DC2x88DC3CANDC4BNAKNULETBFSNULxA9NULxADNULxB1NULxB5NULxCANULfEOT
xA8EOTxDFBSxEDBSxFFBSxBF
8
p
xA3
xF1
vVTxB4VT
DC1DC4DC1xAEDC15DC2cDC2xADDC2WDC4vDC4MNAKx81CANDC2NULNULSTXx84ENQxF5ENQxB5ACKXBS
x8DBSxE5BS#
J
ETX
xF0
xA5DC1ESCDC2gDC2.DC3xAESYNsETBxFECAN#NUL
NULxCBSOHvSTXySTXZENQx8BBELx93BELxA0   xFD
x9BVTxE6VTxFBVTDC1FFxB3FFfSOiSOx90SOxDLExDADLE<DC1xA0DC1xEEDC18DC4x96DC4xE1DC4xFADC4
/NAK8NAKx9CNAKx80SYN=ETBLETBxETBSUBCANpCANEMNUL?NULxEANULxF7SOHxETXxA6EOTxF1EOTx89BEL
x88BSFS xF7 H
xB2VTxBFSIxEBSIEOTDLEESCDLEx8DDC1ZDC26DC4xF1DC4xC7NAKx8DSYNmETBxAECANxFCCANESCNULA
NULxFCNUL_STXvEOTxF3EOTCANENQmENQuACKxB9ACK>BELD
7VTHVTFFSO9SOx93SOQSIxC7SIxE3SIjDLEBDC1USDC3xA9DC3xF3DC3HDC4xFDNAKxC8SYNSUBNULxF9SOH
xB5STXFFETXxE0ETXTENQxC9ENQ
BSABS_ d   l
xD6
xE2VTxECVTxEASOHSI4DLE DLEtDC2xDCDC2%DC3)DC3VTNAK6ETBRETBxB0CANDC4NULx98ETXxEEEOT
xF8ACKxB5BSxDCBS, xF9
*VTxD9FF5SOx99SOxC1SIx98DC1xFADC1DC2DC3xE8DC3xF0DC3x9FNAKuSYNx88SYNUSNUL6SOHxB0SOH
xB9SOHx95STXxE6STX#ETXzETXx80ETXxA2ETXxB8ETXxBFEOTVTENQKENQx9DENQ8BSx93BSC
xD2VTDC4FF,FFxA6SIxB3SIxEDSIx84DC2xAFDC3xCADC3
SYNx83SYNxA2SYNxBCSYNxDDCAN'NULESCNULETBSOHGSOHTSOHZSOHx9CSOHGSTXx98STX*ETXxABETX
xB0ETXxEDETXxF5ETXxFDETXETXEOTDC2EOT0ENQ}ENQxBEENQxE9ENQxFDENQUSACK$ACKaACKU
xC0
xFA
VT
DSOxF3SIxA9DC1x9EDC2=DC3xA8DC4xE5DC4;SYNxB5SYNDC4CANPCANDC3NULx94EOTsENQxD8ENQXACK
xB2BEL BSF   x83
JVTGFFUFFx8FFFxB9FFxEFFFxD9SI)DC1xFADC2x86NAKxD0NAKUSNULbSOHUSTX}ETXVEOTxF2ENQwACKD
BELx9CBELx96BSk]
xC6
xCA
9VT#FF;SOxB2SOxC7SOxDCSOxE4SO#DLElDLEx86DLExCCDLE\DC1xABDC3xE0DC3xEDDC3FSETBpETBSI
CAN#NULdSTXxDDSTXSUBETXRSETX>ETX_ETX&EOT EOTx85EOTxB5EOTxC2EOT9ENQxB8ENQxC4ENQxE6ENQ
EOTACKBELACK;ACKxACACKDLE
DC4
x87SOxBASOxC0SOxF0DLEDDC1xD8DC2x87DC4x9DDC4*SYN8SYNx9CSYNxD9SYNx97ETB`CANDC2NULENQ
NUL!NULiSTX6ETXGETX"EOT6ACKEMBELx99 xAF
GS
xC3SO^SIxBFDLExC8DC2ETXDC3xBEDC3x8EDC4DC3NUL;EOTjEOT7BELxC6BEL.
xE4
CANVTx8DVTx93VTx82FFxA4FFxACFFx83SIHDLExBFDC1xEADC4
SYNxC4ETBxBFCANNAKNULxA7STXYBELtBSxA5BSxBABSO   FS
SUBFFOFFETXSONAKSO`DC2x99DC2xB5DC3xF5DC3DC2NAKxCASYNx91ETBVTCANx9BCANETXEMSONUL
xCABELPVTxFFVTETBFFxC2FFDC1SOnSIx8ASIxC9SIZDLEx90DLEx8ADC4x81NAKxE1SYNDC2NULDC1NUL
xDBNULx91STXxCFSTXSOENQHACKxDABELNULBS*BSDC4   bFFxFBFFxB8SIxFASIx8BDC2x81DC3
NAKQSYNACKNULxE6SOHyBELySO,SIJDC4ETXETBSUBNULxCAEOTxF8EOTNAKENQ@BELxA8BEL BS}BS
VTxA2VT5FFxB6SO9SIBSIxA2SI-DLEQDLExC9DC1nDC2iDC3x92DC3xF9DC3xDEDC4INAKx95SYN.ETB:ETB
SINUL4SOH!ETXxA4EOTVTACKvBELx81BSx9E
kFFSOSORSOvDLEx88DC2x8FDC3xA0DC4xA6SYNRSNULSINULESTXPSTXxFCSTX&ETX(ETXx92EOTDC3ENQa
ENQcENQx8EENQxE1ENQGSACK4ACKVACKxA6BELxC4BELxC4
x80FFx8DFFBSO7SI|DC1xC6DC2xB3DC4xFFNAKRSSYN6SYNJSYNxC2ETBFSNULCNULxF3SOHRSSTXxC2STXQ
EOTx8EEOTBENQx90ENQxA0ENQSOACKgACKxF6BELUS
STXFFVTFFxC5FFXSOxFESO~DC1BELDC3dDC3SODC4mNAKSOHSYN-SYNLSYNxF9ETBdCAN$NUL`EOTxEOT$
ENQxBBACKxCBACK+BELxFCBSxE2 @
STXVTxD5VTCAN
x8DSOx95SOx9DSOxA9SOxAESOqSIxBCSIxD2SIxFFSIDLEDLE1DLE^DLEDC1DC1SDC1xB3DC1xD1DC2
x8BDC3NULNAKWSYNrSYN{SYNxD9ETBRSCANxABCAN=NULxFENULxB3SOH1STX5STX9STXASTXKSTXRSTXaSTX
xEASTXxEFSTXxF4STXxF9STXxFESTXNETXx9DETXxCBETXDEOTIEOTx9FEOTxB8EOTxC7EOTxE3EOTxF5EOT
SUBENQ ENQNENQeENQoENQx8BENQx96ENQxE3ENQx95ACKxAFACKxEFACKxC0BELSOH
WVTxA9VT FF/FF2FFnFFxF8FFUSOSSIxE5SIcDLEx98DLE!DC3SDC3xB5DC4
SYNfSYNnSYNx90SYNx99SYNDLEETB$ETBxEBETB&CAN%NULSISOH,SOH>SOHtSOHxSOHx91SOHx97SOH
xD1SOHxABSTXeETXxD1ETXxE2ETX

```
EOTxFEEOTxA2ENQxB4ENQgBSxCC u
|
xCAVTEOTFF
FFxC2
xC8
x80SOxF4SONULSI%SIx8DSIx9ESIxB1DLEzDC3]NAKxBCNAKxF2ETBxECCANSUBNULxEFNULx86SOHDC2STX%STXEOTETXxC7ETXSTXENQ(ENQFFBELKBELpBELxB0BS& xD1FF!
xEESOzSIxBADLExD4DLEuDC1x80DC1xDEDC2DC3xFFDC3xBFDC4xF3CANETBNUL)NULgNULxDDETXVENQ BELxBABELxE8BELpBSqFFS
-SORSDLESOHDC1USDC1xB7DC1:DC2LDC2xB5DC2+DC3VDC3DLEDC4
NAKxE1CAN&NUL:NULJNULxE4NULxF6NUL)SOHx83SOH1ETXx91ETX7EOTxBBEOTBELENQ4ENQx92ENQDLEACK>ACKAACKDACKxF8BELx9C
1SOx8ASOxCDSOSUBSIUDLExA1DLExA4DLErDC1]DC2xE7DC2xF2DC2xF6DC2xA5DC3(DC43NAKFNAKNSYNcSYN)CANFSNULENULMNUL SOHxC4STXxE0STXkETXxCBENQiACKxC3BSxD4 d
x80
cVTuFFxC7FFxE6FFx9E
xE5
uSI6DLEnDC1x90DC1xB9DC3nDC4xF4DC4xA9SYN.CANxE6CAN,NULx94NULxD7NULxDFNUL,STXx9DSTXxB1STXSOETX-EOT1EOTx98ACKiBELSIBSFSBS.BSCBS< xDB EOT
SYN
DLEVTxC0VTA
xF4
xD6SODC2SIGSSIgSIGDC1KDC1xE5DC1xD8DC3WNAKeNAKoNAKxF2NAKxE7SYNTETBxB7ETBxCEETBxB5CANxBACANxC4CANxC9CANxD1CANSYNNULuNULxD5SOHZEOTrEOTxA9ENQDC4ACKNULBELx8FBEL;BSxC8BS8EVTx97
xAC
CANDC2ADC2xBDDC2xEADC2xA3DC4xA7NAKxDFETBfCANFSNULZSTXxA5ETXSEOTxD7EOTxDCEOT=ENQxBBENQxD5ACKxE5BELxEE   TVTxEEVTxF6VT|FFH
xA6
xA9
(SOtSOx81DLEETBDC3fDC3xB2DC3jDC4(NAK^ETBeETBxFBETBDC1NUL.NULTNULx8CACKQBSxD5BS!     %
ISOpSO
SIxDADC1xC5DC3xCFDC3GSDC4ADC4xCANAK6CANSINULx83ETXx9EACKxDBACKDC4BELACK~VTxDDFFJSIBELDLEvDC20DC4xBADC4x97NAKxD6NAKxA7CANSUBNULxE7NULxC8SOHxB7STXrACKxB2ACKxCDBELxF1BELA   a    xD8
sVT1
x8B
xB2
xB6
rDC3xA1DC3sDC4xD4DC4xB5NAKENQSYN/SYNxE7ETBxEEETBzCANxF8CANCANNULxF5SOH
STXxA0ETX"BEL?BSx86BSx8BBSf n
x99
x9FVTxE4VTxF9VTbSIrDC2xABDC2'DC3~DC3FDC4xD1DC46NAK
ETB CANxB2CANDC4NUL`SOHxC0STXxCDSTXx90EOTx9DEOTSTXACK{BSi   ,
EFFSODLEFDLEx8EDLExC7DC1xCFDC2x89DC3x84NAKx93SYNSOHETB8ETBDLENUL8NULxEDNULDLESTX#STXxCBSTXxDBETXLEOTxB2ENQDC2BELnBELx84BSF
xECSOeSIxB8DC4ETXSYNNAKNULxBCNULSUBSTXmSTXDENQ%BELxCEBS

x8E
x97VTZSOxA1SOxD2SOx9BDLExA8DLExF6DLEEMDC4+DC4FSNAK\SYNaETBSYNNULxBFSOHxFBSOHACKSTXSTXxA8ETXxF1ETXNEOTx88EOTx9AEOTxDFEOTx99ENQx89ACKxAB
xF1VTPDC1xF2DC1TDC2xD5DC2$NAKCNAKETBSYNxDCSYNFSNULSOHSOHoSTXVETXxBCETXxC0ETXxB1EOTxBDACKxCDACKxF3ACKxFAACKxE0BSxE9BSxE7   9
xF2
EOTVTwVTx87VT>SIxABSI:DLEx9DDLExAADLEUDC1xAEDC2^DC4NNAKXETBFSNULbNULxCBNULxD3NULEMEOTxA9EOTxD0EOThENQ-BELbBELxD0BSxC0 SO
x90
xA4
xACVTxB5VTxD7VTxA3SOZSICANDC1xAFDC16DC2 DC2wDC4+NAKSUBSYNDC4ETBxAEETBEMNULxAANULxAENULxB2NULxB6NULxADSOHxB6SOHxC4SOHDC4ETXZETX|ACKxBAFSVTcSOxC3SIxE0SIDC2DLE?DLEdDC2=DC4XDC4STXNAK$SYNxFEETBx82CANxDACANUSNUL5NUL;SOHSTX<STXxD6STXAETXQETXbETXx8EETXx9AETXgEOTxEFENQxD8ACKxEEBSNUL
     DC1 L    xE4 q
x94
xABSOxBDSOxCESI`DLESODC1NAKDC1xBADC2DC4DC3-DC4xF6ETBFFNULNAKNULxDCSOHSOHSTXFENQxF6ENQEOT
K
xF8DLExF5DC1DDC3jSYNxCDCANVTNULxA4NULzEOTx85ENQYBSxF2BS$
K
xE9DLEFSDC2xAFSYNxA3CANDC4NULxB6ACKxE2ACKQBEL. xF3 x9F
,VTxC6VTx
NSOx92SIrDLEWDC2hDC2/DC3jETBtETBx9BETBCANxFFCANDC1NULxD9STXxE9EOTxFCBELx8EBSxE6BS|    x96
```

540 pVTxF1
541 xD1DLExEDDLExF3DLExA6DC1x96DC2wDC3YSYN2CANSINULxCCSOHzSTXmACKZVTxE7VTFSSOyDLExDBDLE=
DC10DC2x97DC4^SYNxEESYNMETBYCANSONULVTNUL'BELxA1
x83VTxB4FF\SOxD4SIfDLEx93DLEFDC2xA6DC29DC4xFBDC4xDBETBGSNULxBENULbEOT[ENOACKBEL
x94BELa
542 ~
543 jSOxE8SISOHDLEx9ADC1xA1DC1xFCDC1SUBDC3SDC4xC4DC49NAK?NAKxA1NAKwSYN}SYNx8ASYNx9FSYN
xBFSYNxD4SYN(ETB>ETByETBqCAN&NULxBCSOHxFESOHETBSTXwSTXSOHETX
544 ETXKETXx82EOTx82ENO4BELx8CBELxB7BSxC9 xFB xFE 6
545 x99VTx9CVTxFCVTDC2FF*FFSUB
546 %SO7SOgSOx91SOx97SOxF0SI&DC1xEFDC1x9EDC3xEADC3xE2DC40NAKx9DNAKx81SYNx86SYNESCCANNAK
NULUSNULFSSTX<ETXDLEEOTx8CEOT7ENOx89ENOxC2ENOGS x81
xC0FFwSOx9BSOxA7SOxB0SOxB6SIx8DDC3RSNAKx8ESYNxDFSYNnETBGSNULxEBNULxD9ETXxD5EOTxDAEOT
&ENOxE3BELx89BSEOT x8C
547 xDBFFx9FSOxD0SOxD4SOsSIENODLEFFDLEFSDLEx8EDC1xB5DC1xC5DC1[DC2xD3DC2ESCDC4hDC4xCFDC4
xF2DC4x95NAKxC8NAKcETBESCNUL NUL>NULxC9NULxA7EOTxF0EOTx88BELx8ABELxDEBSxFEBSFF
548 G
549 I
550 xB3VTxEF
551 x8FSOxBESIDC3DC1;DC17DC4x95DC4xF0DC4x9BNAKxADSYNKETBEMCANxADCANxFDCAN$NUL@NULxFBNUL
xF8SOH^STXyETXx97ETXxB7ETXxBEEOTxEDEOTxF2EOT
552 ENOSENOx9CENOxC8ENO7BSx92BSxDBBSxF8B
553 xD1VTxEBVT4SOPSIxB2SIxC0SIxECSI3DLEx83DC2xDBDC2$DC3xA8DC3xE7DC3xF2DC3tSYNxBBSYN
xAFCAN(NULSUBNULSYNSOHYSOHTSTXcSTXx97STXxDCSTXGSETX|ETXxAAETXxF4ETXxC1EOTrENOxE5ENO
xFCENO`ACKvACKxABACKx9BBELx95BSE xBF
554 xF9
555 8VTIVT"FFxB8FFDC3
556 :SOx86SOxD8SIkDLEx85DLECDC1xD7DC2xF9DC2<DC3xAADC3x9CDC4x9BSYNDC4NULEOTNULhSTXxA6STX5
ETXFETX!EOT:EOTxAE
557 SYNFFxABFFDC4SOx89SIxC8SIYDLESTXDC3xF4DC3x8DDC4FFSYNxC9SYNx90ETBDC1NUL3SOHxA3EOT
xC9EOTxF7EOT
558 ENO
559 ACKGACK?BELxFFBEL4FFxFAFF
560 SO+SIPDLEx87DC2IDC4xA5SYN:NULBNULxFDNULxB2SOH4STX8STX@STXDSTXOSTX`STXxE9STXxEESTX
xF3STXxF8STXxFBSTX%ETXHEOTPEOT_EOTwEOTxC6EOTxE2EOTxF4EOTEMENOUSENO#ENOAENOMENOnENO
x8DENOx95ENOx9FENOxBAACKxCAACKxC3BELxA8VTxD4VT.FF1FF FFETB
561 WSOx94SO6SIRSIxD1SIxE4SI]DLERDC1xC5DC2 DC3
562 DC4xB2DC4xFFDC4lNAKxFENAKVSYNqSYNcCAN
NUL(SOH0ETXxC6ETXxD0ETXxE1ETX6EOTxBAEOTxFDEOTACKENOUENOfBSxAFBS%t
563 pFF,SOEMSI$SIx9DSIRSDC1xB4DC2xDDDC2xF1DC2xF5DC2*DC3UDC3FFNAKENAKbSYN(CANxE0CANxF2CAN
GSNUL~SOHxB0STX
564 ETXxA4ETX,EOT<ENOxA8ENOxCAENODC3ACKx97ACKSOBS:BSBBSxED ETX
565 c
566 SVTxEDVTxF5VTxE5FF5DLEx80DLE@DC2xB1DC3mDC4'NAKxE6SYNSETB-CANEMNULSNULFFSTXxB6STX
x82ETXx9FETXx8BACKxB1ACK` e m
567 xD7
568 xE3VTx8A
569 ISTuDC2xAADC2&DC3qDC3xCEDC3EDC4FFETBxE6ETBxEDETB5CANxB1CANDC3NUL_SOHxFASOH"STXxCASTXK
EOTx9CEOTx98ENOx88ACKE
570 xEBSOx9ADLExA7DLEODC1xCEDC2xB7DC4ESCNAKSYNSYNx92SYN7ETBRSNULaNULNULSOH:SOHxB5SOH
xC3SOHBSSTX;STXxD5STXDC3ETXPETXUETXx8DETXx99ETXxBBETXCANEOTgENOxF2ACKxF9ACKDLE
xABVTYSIxAASIxC2SIxCDSIxDFSI~DC2DC3DC3EMSYN#SYNDC3ETB
571 NULxA3NULxE8EOTxFBBELxF1BS- xF2
+VTxE8DLExF4DC1VDC2iSYNiETBx9AETBCANNULxBBSOHxFDSOHSYNSTXNULETXxB6BSxC8 xFA
YVTESCSO$SO6SOxE7SIxEFSIeDLE%DC1x99DC1xFBDC1xE9DC3xA0NAKvSYNx85SYNx89SYNxBESYN'ETB
CANNUL=NULxFANUL]STX;ETXx96ETXxD8ETXx8BEOTxECEOTxEFEOTRENOx9BENOxC7ENOx91BSxDDBSETX
F
572 xDAFFxEF
573 x9ASOxB5SI#DC3xF1DC3xDESYNCANCAN2NULEMNUL2SOHXSOHxB1SOH3STX7STX?STXCSTXNSTXgSTXx96STX
xA5STXxE8STXxEDSTXxF2STXxF7STX$ETX4ETXEETX{ETXxF3ETX
EOTGEOTxA2EOTxC0EOTxC5EOTxE1EOTFFENORSENO"ENO@ENOLENOqENOx9EENOxAAACKxC2BELx94BSxAD
574 xD3VTNAKFF-FFx88SIODLEXDLEx84DLEx86DC2x9BDC4VTSYNpSYNxA4SYNSYNNULRNUL^SOH}SOHxAFSTX/
ETXx81ETXxA3ETXxCFETX+EOT5EOT9BSxF4VT+SOxB3DC2xCDDC2xB0DC3xCDDC3SUBNAK&NAKxE5SYN,CAN
xDFCANRSNULxF9NUL9SOHxBASOHxC2SOHxD4STXDC2ETXTETXx8CETXx95ETXxBAETXxD7ETXx8AEOT
xE7EOTQENOxF1ACKxF1 E
575 SUBSOXSIxA9SIxB4SIxCCSIxEESI"SYNhSYNx84SYNxBDSYNDC2ETB&ETBhETB*NUL1SOH7SOH8SOHxC1SOH
MSTXxE7STXxECSTXxF1STXxF6STX.ETXDETXx94ETXxB9ETXxCDETXxCEETX)EOT*EOTFEOTxA1EOTxE5EOT
xE6EOTFSENOGSENOPENOD
576 xF3VTUSIVSIWSIxA7SIxA8SILDLEMDLENDLEx85DC2xB2DC2xCBDC3xCCDC3
577 SYNxA3SYNxE4SYNxDECANETXNULNUL2xDFSOHNULNULDC3nGACNNNNNNGTC,CCWGGTACCWGG,GCACNNNAACGT
TNNGTGC,CACNNNGTG,AACNAACRYGTGCGTGC,GCACGCACRYGTTNGTT,GCACNAACRYGTTNGTGC,AACGCACRYGTG
CGTT,GACNNNNNGTC,GAANNNNNNNTTGG,CCAANNNNNNNTTC,CACNNNNGTG,AACACNNNNGTGC,GCACNNNNGTGTT,
GCANNNNNNTGC,GAACNNNNNNTCC,GGANNNNNNGTTC,CAGNNNCTG,CCAGGNNCTG,CAGNNCCTGG,CCAGGNCCTGG,C

CWGGGCCC,GGGCCCWGG,CCWGGGCCCWGG,AACNNNNNNGTGCACNNNNNNGTT,CCWGGWCCWGG,ACNNNNGTAYC,GRTAC
NNNNGT,CCWGGYRCCWGG,AACGGCNNNGTGC,GCACNNNGCCGTT,GCACGGCNNNGTT,AACNNNGCCGTGC,CGANNNNNNT
GC,GCANNNNNNTCG,CGATCNNNNTGC,GCANNNNGATCG,TGANNNNNNTCA,TGATCNNNNTCA,TGANNNNGATCA,TGATC
NNGATCA,AACCTGCNNGTGC,GCACNNGCAGGTT,GCACCTGCNNGTT,AACNNGCAGGTGC,GCCNNNNNGGC,AACNNNNNNG
TGCMC,GKGCACNNNNNNGTT,AACTGGGNNGTGC,GCACNNCCCAGTT,GCACTGGGNNGTT,AACNNCCCAGTGC,GACNNNNG
TC,GAGNNNNNCTC,CCWGGTCTC,GAGACCWGG,GATNNNNATC,GATCNNNATC,GATNNNGATC,GATCNNGATC,CCWGGCG
CCWGG,ACNNNNNCTCC,GGAGNNNNNGT,CCNNNNNNNGG,CCWGGNCCWGG,AACNNNNNNGTGCAG,CTGCACNNNNNNGTT,
GATCCGGATC,GATCATGATC,CCWGGNNCCWGG,AACTGGNNNGTGC,GCACNNNCCAGTT,GCACTGGNNNGTT,AACNNNCCA
GTGC,AACNAACCGGTGCGTGC,GCACGCACCGGTTNGTT,GCACNAACCGGTTNGTGC,AACGCACCGGTGCGTT,GCANNNNNT
GC,GCACNNNNNNGTTCGAACNNNNNNGTGC,CCANNNNNNTGG,CCAGGNNCCTGG,CCTGGCCAGG,CAANNNNNGTGG,CCAC
NNNNNTTG,TTTAAACNNNNNNGTGC,GCACNNNNNNGTTTAAA,GCACNNNNNNGTTTAAACNNNNNNGTGC,CCAGGCCTGG,C
CTNNNNNAGG,CCAGGNCCY,RGGNCCTGG,CCWGGGNCCTGG,CCAGGNCCCWGG,GAYNNNNNVTC,GABNNNNNRTC,GATCN
NNNVTC,GABNNNNGATC,GAYNNNNGATC,GATCNNNNRTC,GATCNNNGATC,GTYAACNNNNNNGTGC,GCACNNNNNNGTTR
AC,GCACNNNNNNGTTAACNNNNNNGTGC,GATCNGATC,GCNNNNNNNGC,TAACTATAACGGTCCTAAGGTAGCGAA,TTCGCT
ACCTTAGGACCGTTATAGTTA,TAGGGATAACAGGGTAAT,ATTACCCTGTTATCCCTA,GTTAACNNNNNNGTGC,GCACNNNNN
NGTTAAC,AACNAACGCGTGCGTGC,GCACGCACGCGTTNGTT,GCACNAACGCGTTNGTGC,AACGCACGCGTGCGTT,TCCAAC
NNNNNNGTGC,GCACNNNNNNGTTGGA,CAYNNNNRTG,AACAYNNNNGTGC,GCACNNNNRTGTT,GTTTAAACNNNNNNGTGC,
GCACNNNNNNGTTTAAAC,CCWGGCGCC,GGCGCCWGG,CCWGGNNCC,GGNNCCWGG,GATCGCGATC,AACNAACATGTGCGTG
C,GCACGCACATGTTNGTT,GCACNAACATGTTNGTGC,AACGCACATGTGCGTT,GAANNNNTTC,GACNNNGTC,CCANNNNNT
GG,CCAGGNNNTGG,CCANNNCCTGG,TGGCAAACAGCTATTATGGGTATTATGGGT,ACCCATAATACCCATAATAGCTGTTTGC
CA,ATCTATGTCGGGTGCGGAGAAAGAGGTAATGAAATGGCA,TGCCATTTCATTACCTCTTTCTCCGCACCCGACATAGAT,AAC
NNGCACGTGCNNGTT,GAACNNNNNCTC,GAGNNNNNGTTC,CCAGGWCCY,RGGWCCTGG,CCWGGGWCCTGG,CCAGGWCCCWG
G,CCAGGWCCTGG,TTATAACNNNNNNGTGC,GCACNNNNNNGTTATAA,GCACNNNNNNGTTATAACNNNNNNGTGC,GGCCNNN
NNGGCC,AACNNACTAGTGC,GCACTAGTNNGTT,AACTAGTNNGTGC,GCACNNACTAGTT,AACNAACTAGTGCGTGC,GCACG
CACTAGTTNGTT,GCACNAACTAGTTNGTGC,AACGCACTAGTGCGTT,AACNNNAACGTGCNNGTGC,GCACNNGCACGTTNNNG
TT,TTAACNNNNNNGTGC,GCACNNNNNNGTTAA,CACNNNNNNTCC,GGANNNNNNGTG,CACNNNNGATCC,GGATCNNNNGTG
,GATCTAGATC,CCANNNNNNNNNTGG,ATAACGGTCCTAAGGTAGCGAA,TTCGCTACCTTAGGACCGTTAT,GCACCCANNNGT
T,AACNNNTGGGTGC,TAACTATGACTCTCTTAAGGTAGCCAAAT,ATTTGGCTACCTTAAGAGAGTCATAGTTA,CCWGGTACC,
GGTACCWGG,GAAGNNNNNNTAC,GTANNNNNNCTTC,CGAGTTCATAGCTGGGCCCAAC,GTTGGGCCCAGCTATGAACTCG,AA
GNNNNNCTT,GAACNNNNNNTAC,GTANNNNNNGTTC,GAGNNNNNATC,GAGNNNNNGTC,ATCTATGTCGGGTGCGGAGAAAGA
GGTAATGAAATGG,CCATTTCATTACCTCTTTCTCCGCACCCGACATAGAT,GGCGCGCC,GCGATCGC,CCWGGWCC,GGWCCWG
G,CCWGGGAC,GTCCCWGG,GATCGATC,GATCCGGA,TCCGGATC,GATCATGA,TCATGATC,CCTGGCCR,YGGCCAGG,CCW
GGNCC,GGNCCWGG,CCAGGCCT,AGGCCTGG,GGCCGGCC,RTGCGCAY,GATCNNGA,TCNNGATC,CCTGGCCA,TGGCCAGG
,GTTTAAAC,GCGGCCGC,GATCGCGA,TCGCGATC,TTAATTAA,VCTCGAGB,CCTGCAGG,CRCCGGYG,ATTTAAAT,GATC
TAGA,TCTAGATC,CGCGCGCG,CGCCGGCG,CGTCGACG,GCCCGGGC,CCTCGAGG,CACCTGC,GCAGGTG,CCTCAGC,GCT
GAGG,GCTNAGC,CCTNAGC,GCTNAGG,GATCGAT,ATCGATC,GGTNACC,CCTNAGG,CCCGGGG,CCCCGGG,CGGWCCG,C
CTGGAG,CTCCAGG,RGGNCCY,GGTGATC,GATCACC,GATCNGA,TCNGATC,GCTCTTC,GAAGAGC,GAAGATC,GATCTTC
,CCCWGGG,TCCNGGA,TCCWGGA,RGGWCCY,ACCWGGT,GGGWCCC,GACGTC,GTMKAC,GGTACC,AACGTT,CTGAAG,CT
TCAG,AGCGCT,CTTAAG,ACRYGT,ACCGGT,CACGTC,GACGTG,GWGCWC,GTGCAC,GGGCCC,RAATTY,ATTAAT,CYCG
RG,CCTAGG,GGATCC,GGYRCC,GRGCYC,CACGAG,CTCGTG,GAAGAC,GTCTTC,GTATCC,GGATAC,TGATCA,ACTAGT
,ACTGGG,CCCAGT,CTRYAG,ATGCAT,ACCTGC,GCAGGT,AGATCT,GKGCMC,GCTAGC,CTGGAG,CTCCAG,CTTGAG,C
TCAAG,GGTCTC,GAGACC,YACGTR,GRCGYC,CCNNGG,WCCGGW,GCAATG,CATTGC,GAGGAG,CTCCTC,CCCAGC,GCT
GGG,GTGCAG,CTGCAC,CGRYCG,CGTACG,GAATGC,GCATTC,CGTCTC,GAGACG,TTCGAA,GDGCHC,TGTACA,RGCGC
Y,TCGCGA,ATCGAT,TCCGGA,TCATGA,GGNNCC,CCGCTC,GAGCGG,RCCGGY,GCGCGC,GTATAC,RGATCY,CCRYGG,
GCGATG,CATCGC,GCAGTG,CACTGC,GCNNGC,YGGCCR,CCGCGG,TTTAAA,CGGCCG,CTCTTC,GAAGAG,GGCGGA,TC
CGCC,GAGCTC,GATATC,TACGTA,CCWWGG,AGGCCT,CTGRAG,CTYCAG,CACGTG,CAGCAG,CTGCTG,GAATTC,GGCG
CC,TGCGCA,GTYRAC,AAGCTT,GTTAAC,TCNNGA,GTNNAC,CAATTG,TGGCCA,ACGCGT,TCCRAC,GTYGGA,CMGCKG
,GCCGGC,CCATGG,CATATG,RCATGY,GCATGC,CTCGAG,ACATGT,TTATAA,CTGCAG,CGATCG,CAGCTG,GTCGAC,A
GTACT,CCCGGG,CTYRAG,AATATT,GATCGA,TCGATC,WGTACW,TARCCA,TGGYTA,TCTAGA,GCCGAG,CTCGGC,GAC
CGA,CACCCA,TCGGTC,TGGGTG,GGATC,GATCC,GTCTC,GAGAC,GCWGC,GGWCC,GCAGC,GCTGC,CCATC,GATGG,A
CGGC,GCCGT,CCSGG,CCNGG,CCWGG,GGATG,CATCC,CTCAG,CTGAG,ACTGG,CCAGT,GGGAC,GTCCC,GGNCC,GAC
GC,GCGTC,CTNAG,CCCGC,GCGGG,GCNGC,GANTC,GGTGA,TCACC,TCNGA,CGWCG,ACNGT,GCATC,GATGC,GAAGA
,TCTTC,GAGTC,GACTC,GTSAC,GAWTC,GCSGC,CASTG,CCTTC,GAAGG,ATGAA,TTCAT,ACGGA,TCCGT,GTNAC,C
AGTG,CACTG,CCGC,GCGG,AGCT,CTAG,GATC,CGCG,GGCC,GTAC,CATG,GCGC,CCGG,TGCA,ACGT,CCTC,GAGG,
TTAA,TCGA,AATT,RGCY,ASST,CNNG,YATRNULNULNULETXNULNULNULNULNULNULNULNULNULNULNULSTXNUL
NULNULNULNULNULNULNULNULNULNULNULNULNULNULNULNULNULNULSOHNULNULNULNULNULNULNULNULNUL
NULNULSOHNULNULNULNULNULNULNULNULNULNULNULSOHNULNULNULNULNULNULNULSOHNULNULNULSOHNUL
NULNULNULNULNULNULNULNULNULNULNULNULNULNULNULNULNULNULNULNULNULNULNULNULNULNULNULNUL
NULNULNULNULNULNULNULNULNULNULNULNULNULNULNULNULNULNULNULNULNULNULNULNULNULNULNULNUL
NULNULNULNULNULNULSTXNULNULNULSTXNULNULNULNULNULNULNULNULNULNULNULSTXNULNULNULNULNUL
NULNULNULNULNULNULNULNULNULNULNULNULNULNULNULNULNULNULNULNULNULNULNULNULNULNULEOTNUL
NULNULSOHNULNULNULNULNULNULNULNULNULNULNULNULNULNULNULNULNULNULNULNULNULNULNULSOHNUL
NULNULSOHNULNULNULNULNULNULNULNULNULNULNULSOHNULNULNULNULNULNULNULSOHNULNULNULNULNUL
NULNULNULNULNULNULSTXNULNULNULENONULNULNUL*NULNULNULNULNULNULNULNULNULNULNULNULNUL
NULNULNULNULNULNULNULNULNULNULSOHNULNULNULNULNULNULNULNULNULNULNULNULNULNULNULNULNUL
NULNULNULNULNULNULNULNULNULNULNULNULNULNULNULNULNULNULSOHNULNULNULNULNULNULNULSTXNUL
NULNULNULNULNULNULNULNULNULNULNULNULNULNULNULNULNULNULNULNULNULNULNULNULNULNULNULNUL
NULNULNULNULNULNULEOTNULNULNULNULNULNULNULNULNULNULNULNULNULNULNULNULNULNULNULENONUL
NULNULACKNULNULNULNULNULNULNULNULNULNULNULNULNULNULNULNULNULNULNULNULNULNULNULNULNUL
NULNULNULNULNULNULNULNULNULNULNULNULNULNUL'NULNULNULNULNULNULNULNULNULNULNULNULNUL
NULNULNULNULNULNULNULNULNULNULNULNULNULNULNULNULNULNULNULNULNULNULNULNULNULNULNULNUL
NULNULNULNULNULNULNULNULNULNULACKNULNULNULNULNULNULNULNULNULNULNULNULNULNULNULNULNUL
NULNULNULNULNULNULNULNULNULNULETXNULNULNULSOHNULNULNULNULNULNULNULNULNULNULNULNULNUL

NULNULSOHNULNULNULNULNULNULNULSTXNULNULNULSTXNULNULNULNULNULNULNULNULNULNULNULNULNUL
NULNULSOHNULNULNULNULNULNULNULNULNULNULNULNULNULNULNULSOHNULNULNULSOHNULNULNULETXNUL
NULNULNULNULNULNULNULNULNULNULNULNULNULNULNULNULNULNULSOHNULNULNULNULNULNULNULSTXNUL
NULNULNULNULNULNULSOHNULNULNULSTXNULNULNULNULNULNULNULNULNULNULSOHNULNULNULSOHNUL
NULNULNULNULNULNULNULNULNULNULNULNULNULNULNULNULNULNULNULNULNULNULNULNULNULNULNULNUL
NULNULNULNULNULNULNULNULNULNULNULNULNULNULNULVTNULNULNULNULNULNULNULNULNULNULNULNUL
NULNULSOHNULNULNULSOHNULNULNULSOHNULNULNULSOHNULNULNULSTXNULNULNULSOHNULNULNULSTXNUL
NULNULSTXNULNULNULEOTNULNULNULNULNULNULNULNULNULNULNULNULNULNULNULNULSOHNULNULNULSTXNUL
NULNULSTXNULNULNULSTXNULNULNULETXNULNULNULSOHNULNULNULNULNULNULNULENONULNULNULNULNUL
NULNULNULNULNULNULNULNULNULNULBSNULNULNULSTXNULNULNULACKNULNULNULEOTNULNULNULSOHNUL
NULNULVTNULNULNULNULNULNULNULSOHNULNULNUL
578 NULNULNUL
579 NULNULNULNULNULNULNULSTXNULNULNULSTXNULNULNULSTXNULNULNULNULNULNULNULEOTNULNULNULSOH
NULNULNULNULNULNULNULSOHNULNULNULETXNULNULNULBELNULNULNULNULNULNULNULSOHNULNULNULENO
NULNULNULNULNULNULNUL
NULNULNULSOHNULNULNULSTXNULNULNULSTXNULNULNULSTXNULNULNULETXNULNULNULNULNULNULNULSOH
NULNULNULSTXNULNULNUL NULNULNULETBNULNULNUL
NULNULNULSOHNULNULNULSOHNULNULNULSTXNULNULNULSTXNULNULNULSTXNULNULNULETXNULNULNULSOH
NULNULNULSOHNULNULNULFFNULNULNULNULNULNULNULSOHNULNULNULETXNULNULNULSOHNULNULNULNUL
NULNULNULSOHNULNULNULNAKNULNULNULSOHNULNULNUL
NULNULNULNULNULNULNULETXNULNULNULSTXNULNULNULETXNULNULNUL.NULNULNULSTXNULNULNULENO
NULNULNULVTNULNULNULETXNULNULNULNULNULNULNULVTNULNULNULENONULNULNULNULNULNULNULSOH
NULNULNULENONULNULNULSOHNULNULNUL)NULNULNULBELNULNULNULSOHNULNULNULENONULNULNULENO
NULNULNULEOTNULNULNULSTXNULNULNULSTXNULNULNULBSNULNULNULSOHNULNULNULNULNULNULNULNUL
NULNULNULSTXNULNULNULSOHNULNULNULEOTNULNULNULENONULNULNULSOHNULNULNULACKNULNULNULSTX
NULNULNULNULNULNULNULEOTNULNULNULSTXNULNULNULETXNULNULNULSOHNULNULNULSOHNULNULNULDC3
NULNULNULSINULNULNULEOTNULNULNULNULNULNULNULSTXNULNULNULETXNULNULNULSOHNULNULNULBEL
NULNULNULACKNULNULNULSOHNULNULNULNULNULNULNULETXNULNULNULSOHNULNULNULSOHNULNULNULSOH
NULNULNULEOTNULNULNULETXNULNULNULSTXNULNULNULNULNULNULNULSOHNULNULNULETXNULNULNULEOT
NULNULNULACKNULNULNULEOTNULNULNULNULNULNULNULNULNULNULNULENONULNULNULSOHNULNULNULENO
NULNULNULSOHNULNULNULSOHNULNULNULETXNULNULNULSTXNULNULNULSOHNULNULNULETXNULNULNULSOH
NULNULNULACKNULNULNULBSNULNULNULEOTNULNULNULETXNULNULNULGSNULNULNUL
NULNULNULDC2NULNULNULVTNULNULNUL
580 NULNULNULENONULNULNULEOTNULNULNULACKNULNULNULSYNNULNULNUL#NULNULNUL
581 NULNULNULEOTNULNULNULENONULNULNULEOTNULNULNULACKNULNULNULBELNULNULNUL
NULNULNULBELNULNULNUL NULNULNUL"NULNULNUL NULNULNULSTXNULNULNUL
582 NULNULNULETBNULNULNULSONULNULNUL6NULNULNULDC4NULNULNULENONULNULNULACKNULNULNULDC4NUL
NULNULVTNULNULNULDLENULNULNULEOTNULNULNULENONULNULNUL
NULNULNULSONULNULNULBELNULNULNULEOTNULNULNULBSNULNULNUL
NULNULNULEMNULNULNULDLENULNULNUL
583 NULNULNUL
584 NULNULNULACKNULNULNULENONULNULNULEOTNULNULNULETXNULNULNULDC3NULNULNUL
NULNULNULBELNULNULNUL:NULNULNUL)NULNULNULEMNULNULNULFFNULNULNULETBNULNULNULRSNULNUL
NUL"NULNULNULVTNULNULNULDC1NULNULNUL+NULNULNUL(NULNULNULFSNULNULNUL
585 NULNULNULCANNULNULNULSUBNULNULNULEMNULNULNULDLENULNULNULSYNNULNULNULsNULNULNULPNUL
NULSOHxB3NULNULNUL<NULETXNULNULNULNULNULSTXNULNULNULNULNULNULNULNULSOHNULNULNUL
NULNULSOHNULNULNULNULNULSOHNULNULNULSOHNULSOHNULNULNULNULNULNULNULNULNULNULNULNUL
NULNULNULNULNULNULNULNULNULNULNULNULNULNULNULNULNULNULSTXNULSTXNULNULNULNULSTXNUL
NULNULNULNULNULNULNULNULNULNULNULNULNULEOTNULSOHNULNULNULNULNULNULNULNULNULNULNULNUL
SOHNULSOHNULNULNULNULNULSOHNULNULNULSOHNULNULNULNULNULSTXNULENONUL*NULNULNULNULNUL
NULNULNULNULNULNULSOHNULNULNULNULNULNULNULNULNULNULNULNULNULNULNULNULNULSOHNULNULNUL
STXNULNULNULNULNULNULNULNULNULNULNULNULNULNULNULEOTNULNULNULNULNULNULNUL
ENONULACKNULNULNULNULNULNULNULNULNULNULNULNULNULNULNULNULNULNUL'NULNULNULNULNUL
NULNULNULNULNULNULNULNULNULNULNULNULNULNULNULNULNULNULNULACKNULNULNULNULNULNUL
NULNULNULNULNULNULETXNULSOHNULNULNULNULNULNULNULNULNULNULNULSOHNULNULNULNULNULNUL
NULNULNULNULNULNULNULNULNULNULNULNULSOHNULSTXNULNULNULNULNULNULNULNULNULNULNULNUL
NULNULNULNULSOHNULNULNULNULNULNULNULNULNULNULNULNULNULNULNULNULNULNULNULNULNULNUL
NULNULSOHNULSTXNULNULNULNULNULNULNULSOHNULNULNULNULNULNULNULNULNULNULNULNULNULNUL
NULNULNULNULNULNULNULNULNULNULNULNULNULNULNULNULNULSTXNULSTXNULNULNULNULNULNULNUL
NULNULNULNULNULNULNULNULNULNULNULNULNULNULNULSOHNULNULNULNULNULNULNULNULNULNULNUL
NULNULSOHNULNULNULSTXNULSTXNULNULNULNULNULNULNULSOHNULNULNULNULNULNULSOHNULSOHNUL
ETXNULNULNULNULNULNULNULNULNULSOHNULNULNULSTXNULNULNULSOHNULSTXNULNULNULNULSOHNUL
SOHNULNULNULNULNULNULNULNULNULNULNULNULNULNULNULNULNULNULNULNULNULVTNULNULNULNULNUL
NULNULSOHNULSOHNULSOHNULSOHNULSTXNULSOHNULSTXNULSTXNULEOTNULNULNULNULNULNULSOHNUL
STXNULSTXNULSTXNULETXNULSOHNULNULNULENONULNULNULNULNULNULBSNULSTXNULACKNULEOTNUL
SOHNULVTNULNULNULSOHNUL
586 NUL
587 NULNULNULSTXNULSTXNULSTXNULNULNULEOTNULSOHNULNULNULSOHNULETXNULBELNULNULNULSOHNULENO
NULNULNUL NULSOHNULSTXNULSTXNULSTXNULETXNULNULNULSOHNULSTXNUL NULETBNUL
NULSOHNULSOHNULSTXNULSTXNULSTXNULETXNULSOHNULSOHNULFFNULNULNULSOHNULETXNULSOHNULNUL
NULSOHNULNAKNULSOHNUL
NULNULNULETXNULSTXNULETXNUL.NULSTXNULENONULVTNULETXNULNULNULVTNULENONULNULNULSOHNUL
ENONULSOHNUL)NULBELNULSOHNULENONULENONULEOTNULSTXNULSTXNULBSNULSOHNULNULNULNULNULSTX

NULSOHNULEOTNULENONULSOHNULACKNULSTXNULNULNULEOTNULSTXNULETXNULSOHNULSOHNULDC3NULSI
NULEOTNULNULNULSTXNULETXNULSOHNULBELNULACKNULSOHNULNULNULETXNULSOHNULSOHNULSOHNULEOT
NULETXNULSTXNULNULNULSOHNULETXNULEOTNULACKNULEOTNULNULNULNULNULENONULSOHNULENONULSOH
NULSOHNULETXNULSTXNULSOHNULETXNULSOHNULACKNULBSNULEOTNULETXNULGSNUL NULDC2NULVTNUL
588 NULENONULEOTNULACKNULSYNNUL#NUL
589 NULEOTNULENONULEOTNULACKNULBELNUL NULBELNUL NUL"NUL NULSTXNUL
590 NULETBNULSONUL6NULDC4NULENONULACKNULDC4NULVTNULDLENULEOTNULENONUL
NULSONULBELNULEOTNULBSNUL NULEMNULDLENUL
591 NUL
592 NULACKNULENONULEOTNULETXNULDC3NUL
NULBELNUL:NUL)NULEMNULFFNULETBNULRSNUL"NULVTNULDC1NUL+NUL(NULFSNUL
593 NULCANNULSUBNULEMNULDLENULSYNNULsNULPSOHxB3NUL<NULNULpDC3NULNULqINULNULqgNULNULq
xA5NULNULuxEDNULNULzxE9NULNULx84GSNULFFoSI,ETB#STXSOHETBx81DC2`SOH-NUL2ETBxA4DLE
x82FFx8ADC4xBAENOHSORSSO[SI'SOHUSEOTxF8DC2xECETBCANNAKLSOxD3FFEOTSOEOTSO9NULxEBEOTn
ETXxEBENOxB3BELx90FF2ETBxFANULSYNSOH7SOH?STX@STXMSTXhSTXxD5STXDC2ETX0ETXx81ETXxA3ETX
xA4ETXxAAETXxB9ETXxBAETXxBBETXxE1ETXwEOTxBAEOTxC5EOTxF7EOT@ENOnENOrENOx9BENOxC7ENO
xFCENOGACKx9BBELSOBSxB6BS- F
594 xDAFF6SOEMSI+SIxD1SIxE8DLE"SYNhETBxF2ACK=SOHSNUL_SOHx9BENOxB6BSF
595 xDAFFDC4STXx94 x84
596 x81DC2xCBDC2oETXxECENOx94 g
597 x92
598 x81DC2'SOH=SOHJSTXYSTX
ETX:ETXJETXaETXx93ETXxB6ETXUSEOT~EOTxB0EOTxB4EOTxB1ENOxD7ENOxE8ENOACKACK
ACK:ACK=ACKhACK-BSxE3 0VTETXFFx85SOxC6SO8SItDC1xF8DC2,SYN.SYNXSYNsSYNx91SYNxECETB
599 CANxD0CANTNUL`SOH~DC3GSDC4x84NAK CANxD0ETXxF2ACK$SIxF5ACKxD9DC4xBCDC1BELNAK3ETB&SOH8
NULxC7STXx90FFe g
600 x92
601 oETXxECENOxD2BSDC1
602 xD2BSxEF
603 xDFBS<DC1_CANRSNUL:SOHRSTXxE3ENO
FFxE3ENOCANNULWSOHACKSTXETXFFxEBCANyEOTaNUL-SOH.SOHySOH+ETXx88ETXx89ETXxD3ETXxD4ETX
DC4EOTHENO'VTxF3DC3x8ESOxB4
x93DC1xECDC1-NAKxCFETXxE0ETXxF8ACKxE0ETXxF8ACKySOHDC4EOTHENO'VTSISONULNUL\ACKgDC3
xDCDC4xACVTCANDC1SOHETXxC9 0NAKx8BSOSONULxCCSTXDC2ENOzBS
SIACKNULxC6NULjSTXxAFENOBFFCDLExCCDC4!NAKCDLE!NAK.SOHUSOH[SOH+ETXx89ETXxD4ETX BSl
604 xBAETBxC5ETB1DC3EMNULUSNULXSOHgSTXx96STX4ETXEETXxF3ETXDLEEOT EOTxAD
605 SONULNULISTXxEFETXBEOTx86EOTxB6EOT\ACKxADACKxE9
x82SORDC2xDASYNxEECANACKNULFSNUL.SOHUSOH[SOH+ETXx89ETXxB4ETXxD4ETXGSEOTxBFENOx89
606 xB8SYNxB6SIRSNAKx90ACKx8EVTDLEFFeSOG xF4
607 xDASIxD1NAKxD1BSx86VT+SOxB3DC2xDFCAN;SOHbETXx8EETXDC2DLExD1DLEwDC3$SYNBELSTX=SOH
x96SOHETXFFFFFFxEBCAN.SOHUSOH[SOH+ETXx89ETXxD4ETXxEEETXCDLE!NAKDC4NULxCDACKwVTSENO
xDBDC2FSSOyDLEsSIx8EDC1xF2DC4xC9DC2xCDSTXSOHETBISTXUSETXxC9ETXBEOTxB6EOTx93ACKxADACK
RSFFx9EDC4EMNULSYNSOHXSOH_SOHDSTXx96STX5ETX;ETXTETXxAAETXxBAETXxCFETXxD0ETXxF3ETX
xCAENOGACK`ACKx8BACKxF1ACKxFFBELfBSxAE
608 xA9SIuACKD 7VTHVTxB7FF9SOxD7SIjDLExA9DC3xBCDC2rDC3xC2
x91FFxEDEOTxDBBS/ETX5EOTxBFETXETXVT9DLExE9CAN5NULSUBETXBELACKxC6
609 DLE
610 DC4
611 DC1SOxB2SOZDLExF5DC3x8ADC4VTCAN`CANN
612 xE6NULqACKxB1
613 xA4EOT5BELACKNULFSNULxC6NUL.SOHUSOH[SOHjSTX+ETXx89ETXxB4ETXxD4ETXxEEETXGSEOTxAFENO
xBFENOx89
614 BFFCDLExCCDC4!NAKxB8SYNwBSISTXBEOTxB6EOTxADACK>
615 x8BSOxFDSIdSYNlSYNSOHSOx99CANSOHEMD
HVTxD4BSxD9DC1xC9NAKNULNUL]NULDC2SOH.SOH/SOHUSOH[SOHzSOHISTX+ETX,ETX~ETXx89ETXxB5ETX
xC2ETXxD3ETXxD4ETXxEFETXDC4EOT'EOTBEOTx86EOTxB6EOTIENOxA4ENO\ACKxADACKxC6ACKxF5ACK
xC4 xE9 i
616 xCFVT
SOx82SO'SINSIRDC2xB0DC2xD9DC2xACDC3xFANAKxB9SYNxC5SYNxDASYNxEECAN#ENOqSYNQSOH#EOT
xC4SOxCDNAK]CANSSTXbSTXxDBSTXFSETXxE4ENOx9ABEL!FFDC2
617 x85SOxD6DC2x9ASYN`ENObENOxE0ENOxE2BS) xEE
618 xD6FF SOx8ADC1x95DC1xEBDC1xF7DC1xF7DC4BSNAK_SOHDSTXGACKx8BACKxFFBEL
619 ETB(NULfNUL~BELR
620 SIDC4+DC4DLENULDC4NUL'SOHSSOHsSOH2STX6STX:STX>STXBSTXFSTXbSTXFSETX)ETXOETXxECETX
x8FEOTxB0EOTxB9EOTxC4EOT!ENO`ENObENOxE0ENOxE4ENOhACKxC1BELSI DC2
621 x85SOxC2SOCANSI=SImDC1xF4DC2aSYNoSYNx9ASYNDC3CAN'CANSUBETX9ENOBELACKDLE
622 DC4
623 DC4`CANDSTXnNULxDE
624 xFEDC1DC1DC2xC5DC4SUBETXBELACKDLE
625 DC4
626 `CANx8ABELx8FSOx9BNAKEMCANxE6ACKxB4
xD6ENOxEDFFxFCNUL_STXxF3EOTCANENOmENOQSIxE3SIUSDC3ACKNULxCAENOx8BACK8ETXxCDACKwVT
xACVTCANDC1SOHETXSENOxC9 xDBDC20NAKxCDSTX)SOHx83SOH>ACKAACKxA1DLErDC1xE4

NAKDC1ISTXBEOTxB6EOTxADACKbDC3xF7ETBxA3VTxA1
SISOjACKxA1
xCBSOHySTXxD4BSxDFBSD xCB xF1
HVTvVTDC4FFETX
xF2SIxDLExDADLE<DC1x8FDC1xD9DC1xDCDC2xC9NAKGSBELxBSw @VTxA3VT%FFx9FFFxA1
SISOCDLE+DC2!NAKxFESYN5ETBFETBxD5SOHNULBELxC8BSxAC
SONULDC2ENOxECVTxC7SI DLE/ETBDSTXxBAACKxCAACKxD4VT]DLERDC1xFFDC4bSTXFSETXxE4ENODC2
x85SOx9ASYNx8BACK-NUL SI USNUL SIx9ANULxD0BELx8D
x81ETB;SOHbETXx8EETXxF5DC3VTCANSISO0BELdDC4x9FCANEMNULXSOHx96STXxF3ETXUSNULFSSOsSIy
DLEx8EDC1xF2DC4x96NULxA8NAKxD1ETBDCAN0BELwBSxBCVTdDC4x9FCANx87
xD9SOHmVTUSDC4DSYNx9FETBETX
SOHDC3<ETX7ENOxC2ENODC2SOx8BDC4$NAKxB1SOxECDC3oETBONAKxE2BSxEE
SOx8ADC1xEBDC1xF7DC4* xD7FF
SO@DC1x96DC1xF8DC1xC8DC3 NAKwETXxA5EOTxB1VTxC6NAKx92
zVTxC9DC24NULINULxE3NULxF5NULx82SOH@ETXaETXx90ETX3ENOx91ENOxEEENOSIACK=ACK@ACKCACK
xF7BELxE3 x93
x89SOxAASOxBCSO_DLExA0DLExA3DLEqDC1xB9DC2'DC4MSYNxF5ETBDC3SOHzSOHxC7STX~ETXNAKEOTI
ENO(VTDLEDC3xEEDC3INULxE3NULxF5NULx82SOHx90ETX3ENOx91ENOSIACK=ACK@ACKCACKxF7BELx89SO
xA0DLExA3DLEqDC1'DC4MSYN4NUL@ETXaETXxEEENOxE3 x93
xAASOxBCSO_DLExB9DC2xF5ETBFFSTXx9FETXe m
xE3VTxAADC2&DC3EDC4FFETBxB1CANEOTSOHxC3ACKgVTkVTxFASYNEMETXETXACKxEFDLExD8SYNxC8EOT
xF6EOT
ACKFACK3FFxF9FFEMNULSUBNULSYNSOHXSOHYSOHx96STXx97STX{ETXxAAETXxF3ETXxF4ETXxC0EOTqENO
xFCENO`ACKxAAACKx94BSxBF
xF9
x84DLE<DC3x9BDC4EMNULSUBNULNULSOHSYNSOHXSOHYSOHx96STXx97STXUETX{ETXxAAETXxBBETX
xD0ETXxE1ETXxF3ETXxF4ETXxC0EOTxFDEOTqENOxFCENO`ACKxAAACKxF2ACKxF9ACKfBSx94BSt
xBF
xF9
$SIx9DSIxAASIx84DLE<DC3x9BDC4NULSOHUETXxBBETXxD0ETXxE1ETXxFDEOTxF2ACKxF9ACKfBSt
$SIx9DSIxAASIETXSOHxB5
fVTjVTxA0ETX?BSrDC2'DC3FDC4&ENOFSDLExB5DC1[DC2bNULEMEOT-BELxC0 x90
wDC4nSTXxE8BSx86VTx9CDLExA9DLE]DC4WETBUENOpFF,SORSDC1xB4DC2*DC3UDC3FFNAKxE0CANDC2SOH
FFEOTDC4EOT~ENOxFEENOxBFBSx89VT5SOHxAFSOHxB8SOHx94STXxE5STX"ETX ETXJENOxDCCANDC3SOH%
SOH.SOH/SOHUSOHVSOH[SOH\SOHzSOHeSTXxADSTXxC7STXxD2STX+ETX,ETX~ETXx89ETXx8AETXxC3ETX
xD4ETXxD5ETXNAKEOT'EOTxC3EOTIENOxF9ENOxA8ACK(VTxB0DC2xFFDC2DLEDC3xEEDC3
ETBNAKCANxC9ETXDC1ENOx93ACKNULFFRSFFxC3FFoSIxB1DC1FSSYN
ETXx9FDC4bNULxA4NULEMEOTzEOT&ENO-BELxC0 K
x90
FSDLExB5DC1[DC2wDC4xB1SOH3STX7STX?STXCSTXNSTXxE8STXxEDSTXxF2STXxF7STX$ETXGEOTxC5EOT
xE1EOTRSENO"ENO@ENOLENOx9EENOxC2BELxD3VT-FFpSYNFSOHSSOHFSTX)ETXxAFETXxECETXSTXEOT|ENO
xE8ENORSACK#ACKCSO:SYNDC3CAN4NULINULxE3NULxF5NULx82SOHxFASTX@ETX^ETXaETXx90ETX%EOT^
EOT~EOTxB4EOT3ENOx8CENOx91ENOx94ENOxB7ENOxEEENOACKACKSIACK:ACK=ACK@ACKCACKxF7BEL
xE3 0FFSI
SYN
x93
VSOx89SOxAASOxBCSOxBFSO5SIxD0SI_DLExA0DLExA3DLEqDC1xB9DC2FFDC4'DC4x86DC4kNAK)SYN7SYN
MSYNxF5ETB_CANbCANxBCSTXuETXSOEOTx96EOTjENOrBELUBSxB2BSEMx9A xD7 xDFVTBEL
xA5SOxF0SO|DLEx81DC23ETBIETBx97CANPACK$FF`DC4QNAKxF7SYNxBESOHENOSTXx99EOTxF1DC1
xD4DC2BNAKxCANULETXETXxA8EOT'ENOxB1BELx82 xBF
xA3
xB4VTxD0FFySIxC7SI(DC1xAEDC15DC2xF3DC3xFEDC3vDC4xC8SYNVTACKvBELxFEVTETBFFkFFxC2FFSO
SORSOnSIx88DC2x8FDC3aSOHxDCETXUEOT{ACKxE7BELxFEBELj
ESCVTbSOxE3SO*SINULDC19DC2<DC4ESCETBxFDETBxF4SOHaSI}DC35NAKxE2SOHSOBELt
SYNSOODC4<EOTx90ACKxC0ACKxE6ACKxB1 xB4 x8EVTxFBDLEx93DC1vSTXx8BBELxFD
x9BVTxFBVTDC1FFfSOx90SOxEEDC1xE1DC4/NAKx9CNAKx80SYNSUBCANuETXx96EOTxB2BSx9A BEL
|DLE3ETBSOEOTxA5SOx81DC2IETBEOTSTXSYNETXx98EOTSIENOx82BSx87DC3ZDC4RSYNxBCSTXjENOrBEL
UBSEM xD7
xDFVTxF0SOx97CANxFASTX^ETX%EOT^EOT~EOTxB4EOTx8CENOx94ENOxB7ENOACKACK:ACK0FFSI
SYN
VSOxBFSO5SIxD0SIFFDC4x86DC4kNAK)SYN7SYN_CANbCAN)NULgNULmSTX BELxCEBSx8E
S
xA1SOx9BDLExA8DLExB7DC1LDC2xB5DC2DLEDC4+DC4xE1CANxBBSOHxFDSOHSYNSTXNULETXxB6BSxC8
xFA $SO6SOxEESI%DC1xE9DC3x85SYNxF1NUL"SOHx88SOH?EOTxD2ENOxE5
xE9FFxFCDC2`NAKENOCANxE4ACKSBELxD5BELx9EBSxD6BS
DC2x9DVTxB4
pDC2x9FDC3xCAN^BSFFFTFFx85NAK>BELFFSOHDC4xB8NULEOTSTXSYNETXx98EOTxF9EOTSIENOx82BSxFF
x86
nDLExADDLE!DC1<DC2x87DC3xC1DC3ZDC4SYNNAKFSYNRSYN)NULgNUL BELS
xB7DC1LDC2xB5DC2DLEDC4xE1CANmSTXxCEBSx8E
xA1SOx9BDLExA8DLE+DC4BNULxFDNULxB2SOH4STX8STX@STXDSTXOSTX`STXxE9STXxEESTXxF3STXxF8STX
xFBSTX%ETXHEOTPEOT_EOTwEOTxC6EOTxE2EOTxF4EOTEMENOUSENO#ENOAENOMENOnENOx8DENOx95ENO
x9FENOxBAACKxCAACKxC3BELxA8VTxD4VT.FF1FF FFETB

```
WSOx94SO6SIRSIxD1SIxE4SI]DLERDC1xC5DC2 DC3
DC4xB2DC4xFFDC4lNAKxFENAKVSYNqSYNcCANFSOHSSOHFSTX)ETX^ETXxAFETXxECETXSTXEOT%EOT~EOTx93EOTxB4EOT|ENOxB7ENOxD7ENOxE8ENOxF1ENOACKACKRSACK#ACK:ACKWACK\
xC5
x8EFFxEEFFSI
CSOxBFSOxC6SOxDBSOxCBDLE[DC1xDFDC3x86DC4xCFNAK)SYN7SYN:SYNDC3CAN_CANBELNULIETXxBCEOTBSENO5ENOxEDENOkACKOBEL5BSxB8 x9D
x81VTx92
2SOxCESOxB0SI
DLE8DLE9DC1BDC3xA6DC3xE5DC3xABSYNxACETB0CANNAKNULxDCSOHSOHSTXFENOxF6ENOEOT
K
xF8DLExF5DC1DDC3jSYNxCDCANxA8STXxD2BSxE3BS*xEF
_FFxD7FFxF5FF
SO@DC1x8BDC1x96DC1x9EDC1xECDC1xF8DC1aDC2xB6DC3xC8DC3xF6DC3xF8DC4NAK-NAKFFCANSINULESTXPSTXxFCSTX&ETX(ETXx92EOTDC3ENOaENOcENOx8EENOxE1ENOGSACK4ACKVACKxA6BELxC4BELxC4
x80FFx8DFFBSO7SI|DC1xC6DC2xB3DC4xFFNAKRSSYN6SYNJSYNxC2ETB&SOH/SOHVSOH\SOHeSTXxADSTXxD2STXESCETX,ETX9ETXx8AETXxC4ETXxD5ETX'EOTxC3EOT:ENOxC5ENOxFAENOBSACKxA8ACKxE3FFDC1
NAK
ETBSI"SI4SIxE6DLExB0DC2NULDC3VTDC4
ETBx98ETBSYNCANaCANSOHNUL]ACKyACK1BELxBSxBDVTxA8FFeDC4MCANVCANxA0CAN.NULTNULx8CACKQBSxD5BS!    %
ISOpSO
SIxDADC1xC5DC3xCFDC3GSDC4ADC4xCANAK6CANJSTXQSTXxFDSTX'ETXx9CETXCEOTxB7EOT`ENObENOdENOx8FENOxE0ENOxE2ENOFSACK3ACKUACKfACKxAEACKxA5BELxBFBELxE1 ?
xC3
x8CFFASOx8CSOxADSOxBBSIxFESIbDLEDLEDC1}DC1ACKDC3cDC3xB4DC4NULSYNUSSYN,SYN5SYNKSYNeSYNmSYNxF8ETBSUBNULSYNSOHYSOHTSTXcSTXx97STXxDCSTXGSETX|ETXxAAETXxF4ETXxC1EOTrENOxE5ENOxFCENO`ACKvACKxABACKx9BBELx95BSExBF
xF9
8VTIVT"FFxB8FFDC3
:SOx86SOxD8SIkDLEx85DLECDC1xD7DC2xF9DC2<DC3xAADC3x9CDC4x9BSYN,NUL6NULxDDNUL<SOH*STXXSTXcETXx8FETXxD9ACKDLEBEL}BELxB8BELSUBBS,BSxCCBSQ
x95
xB0
rSODDLElDC1xBBDC2NAKDC3xD6DC3.DC4"NAKxE4CANxEACANxDANULxCESTXxD9BEL)BS|BSjFFxC8DC1hDC3xDDDC4x94SYNSTXETB-ETB9ETB=NULxFANUL]STX;ETXx96ETXxD8ETXx8BEOTxECEOTxEFEOTRENOx9BENOxC7ENOx91BSxDDBSETX    F
xDAFFxEE
x9ASOxB5SI#DC3xF1DC3xDESYNCANCAN"NULlSOHxA8SOHACKETX7ETXoETXxE5ETXuENOyENOxDAENO7ACKZACKSUBBELxC2 g
xB0
DC4VT&VTx91FFxCE
xD7
|SIxC0DLEPDC2xA1DC2SCANoNULx82STXx9BBST xA2 VTVTxB9VTxA2
xDF
xCBDC1xFFDC1EOTDC2DC2DC2GDC22DC3YDC3xC6DC41SYN@ETBxA2ETBxB3ETBxBEETBxCAETBxD5ETBHCANNULCANBEL6BELsBSxB9BSx98 xFEVTxC1FFFS
STXSODLESOmSIDC1NAKxE0SYNx9ACANSTXEMvNULxD6SOHx9FSTXsEOTSOHBELx90BELxDEBEL!BSHBSxC9BS&
1
xDAVTNUL
m
xAD
STXDC24DC33DC4xBBETBxC6ETBxE0ETBBELNULETBNULGSNUL3NUL;NUL_NULxA1NULxD1NULxF7NUL&SOH/SOHVSOH\SOHxABSOHxDASOHeSTXxA3STXxADSTXxD2STXBSETXESCETX,ETX2ETX9ETX?ETXIETXXETX`ETXx8AETXx92ETXxB5ETXxBEETXxC4ETXxD5ETXRSEOT'EOT8EOTx80EOTxAFEOTxBCEOTxC3EOTxCEEOTBSENO5ENO:ENOx93ENOxA6ENOxC0ENOxC5ENOxEDENOxFAENOBSACKDC1ACKEACKkACKx86ACKxA8ACKxE0ACKOBEL`BELxF9BEL5BSxB8 xC6 x8A
x9D
nVTx81VTxC4VTxCFVTxE3FFDC1
NAK
x92
"SO2SOxBBSOxCESOETBSI"SI4SINSIxB0SI
DLE8DLE=DLEVDLExA5DLExE6DLExF1DLEFSDC19DC1xA4DC2xB0DC2xD9DC2xF3DC2xF7DC2NULDC3BDC3x9CDC3xA6DC3xE5DC3VTDC4x9DSYNxABSYNxB9SYN
ETBx98ETBxACETBxF4ETBSYNCAN*CAN0CANaCANxD8CANBELNULxDANUL?SOHx98SOHxFCSOHBELSTXNAKSTX\STXxCESTXIETXETBEOTxBCEOTxEBEOTBSENO5ENOHENOx9AENOxEDENOOACK[ACKkACKESCBELOBELxD9BEL)BS5BS|BSx90BSxF6BSxB8 h
v
x9D
xE7
'VTx81VTxCBVTENOFFSOFFjFFxB0FFx92
```

xC3
xCF
xD8
xED
2SOx81SOxCESO}SIxB0SIxDESI
DLESYNDLE)DLE8DLExC1DLE$DC19DC1xC8DC1xF3DC1UDC2BDC3hDC3xA6DC3xE5DC3xDDDC4CANSYNx94SYNxABSYNxDDSYNSTXETB-ETB9ETBxACETB0CANKCANTCANxEDCANBELEMENONULSINULDC1NULNAKNULEMNULSUBNULESCNULUSNUL!NUL.NULENULMNULTNULaNULxDBNULNULSOHSOHSOHSISOHSYNSOHETBSOH(SOH,SOH2SOH:SOH>SOHGSOHTSOHXSOHYSOHZSOH`SOHtSOHxSOH SOHx91SOHx97SOHx9CSOHxB1SOHxB5SOHxC3SOHxCCSOHxD1SOHxDCSOHSOHSTXBSSTXFSSTX3STX7STX;STX?STXCSTXESTXGSTXNSTXPSTXTSTXcSTXgSTXiSTXoSTXzSTXx91STXx96STXx97STXx98STXxA5STXxABSTXxC0STXxC4STXxCDSTXxCFSTXxD5STXxDCSTXxE0STXxE8STXxEDSTXxF2STXxF7STXxFCSTXDC3ETXGSETX$ETX&ETX(ETX*ETX0ETX4ETX6ETX<ETXEETXGETXPETXUETXVETXeETXkETX{ETX|ETXx8DETXx99ETXxAAETXxABETXxB0ETXxBBETXxBCETXxC0ETXxC6ETXxD0ETXxD1ETXxE1ETXxE2ETXxEDETXxF3ETXxF4ETXxF5ETXxFDETXETXEOT
EOTDLEEOTDC2EOTCANEOT
EOT"EOT6EOTGEOTx8CEOTx90EOTx92EOTx9DEOTxA2EOTxB1EOTxBAEOTxC0EOTxC1EOTxC5EOTxE1EOTxFDEOTxFEEOTACKENOFFENOSOENODC3ENORSENO"ENO0ENO7ENO@ENOFENOLENOUENOaENOcENOgENOqENOrENO}ENOx89ENOx8EENOx9EENOxA2ENOxB4ENOxBEENOxC2ENOxCBENOxE1ENOxE5ENOxE9ENOxF6ENOxFCENOxFDENOSTXACKGSACKUSACK$ACK4ACK6ACKHACKVACK`ACKaACKiACKmACKvACKx8CACKxAAACKxABACKxBDACKxCDACKxF2ACKxF3ACKxF9ACKxFAACKEMBELx9BBELxA6BELxC2BELxC4BELxDABELNULBS*BSQBSfBSgBS{BSx94BSx95BSxAFBSxC3BSxD5BSxE0BSxE9BSDLE DC4 GS ! % E i x81 x99 xCC xD4 xE7 ,
9
U
d
t
u
|
x80
xAD
xAF
xBF
xC0
xC4
xF2
xF9
xFA
EOTVT8VTIVTZVTcVTwVTx87VTxABVTxCAVTxD3VTxE7VTEOTFF
FFNAKFF"FF-FFEFFbFFpFFuFFx80FFx8DFFxB8FFxC0FFxC7FFxE6FFxFBFFEOT
VT
DC3
GS
%
K
x9E
xC2
xC8
xE5
FSSO,SO:SOBSODSOISOpSOwSOx80SOx86SOx9BSOxA7SOxB0SOxC3SOxF4SONULSI
SIEMSI$SI%SI7SI>SIYSI^SIuSIx88SIx8DSIx9DSIx9ESIxAASIxABSIxB6SIxB8SIxC2SIxCDSIxD8SIxDFSIxF3SIxFASISODLE6DLE:DLEFDLEODLEXDLEkDLEyDLEx84DLEx85DLEx8EDLEx9DDLExAADLExB1DLExBFDLExDBDLExF8DLERSDC1=DC1CDC1UDC1nDC1|DC1x90DC1xA9DC1xC7DC1xDADC1xF5DC10DC2~DC2x86DC2x8BDC2x9EDC2xAEDC2xB4DC2xC6DC2xC8DC2xCFDC2xD7DC2xDDDC2xF1DC2xF5DC2xF9DC2ETXDC3DC3DC3*DC3<DC3=DC3DDC3UDC3zDC3x81DC3x89DC3x8DDC3xAADC3xB9DC3xBEDC3xC5DC3xCFDC3GSDC4ADC4^DC4nDC4x8EDC4x97DC4x9BDC4x9CDC4xA8DC4xB3DC4xE5DC4xF4DC4FFNAKRSNAKNAKENAKNNAK]NAKx84NAKxBCNAKxCANAKxFFNAKVTSYNEMSYNRSSYN#SYN6SYN;SYNJSYNQSYN^SYNbSYNjSYNpSYNx8ESYNx93SYNx9BSYNxA4SYNxA9SYNxB5SYNxDFSYNxEESYNSOHETBDC3ETB8ETBMETBXETBnETBxC2ETBxF2ETBDC4CAN(CAN.CAN6CANPCANYCANxCDCANxE0CANxE6CANxECCANxF2CAN.NULTNULx88NULx9BNULxEFSOHx83ETXx8CACKx9EACKxDBACKxE3ACKDC4BELRBEL\BEL{BELx97BELxD1BELxEBBELQBSxD5BSACK ! x8E xA8 x86
-VT3VT=VT~VTxDDFF%
x8E
xBB
ISO^SOpSO
SIJSIx93SIBELDLExC4DLExDADC1"DC2$DC2&DC2vDC2x99DC3xC5DC3xCFDC3GSDC4#DC40DC4ADC4x83DC4xBADC4x97NAKxCANAKxD6NAKx82ETB6CANxA7CANBSNULNULSOH'<AdditionalSequenceProperties>
<UpstreamStickiness>0</UpstreamStickiness>
<DownstreamStickiness>0</DownstreamStickiness>
<UpstreamModification>FivePrimePhosphorylated</UpstreamModification>
<DownstreamModification>FivePrimePhosphorylated</DownstreamModification>
</AdditionalSequenceProperties>

768


```
NULNULDC4+<?xml version="1.0"?><Features nextValidID="9"><Feature recentID="8"
name="AURKB MUT1 promoter" type="misc_feature" allowSegmentOverlaps="0"
consecutiveTranslationNumbering="1">&lt;Segment range="32-1648" color="#a6acb3"
type="standard"/></Feature><Feature recentID="7" name="luciferase"
directionality="1" translationMW="60645.64" type="CDS" allowSegmentOverlaps="0"
consecutiveTranslationNumbering="1" hitsStopCodon="1">&lt;Segment range="1678-3330"
color="#ffcc00" type="standard" translated="1"/><Q name="codon_start"><V
int="1"/></Q><Q name="EC_number"><V
text="&lt;html&gt;&lt;body&gt;&lt;br&gt;&lt;/body&gt;&lt;/html&gt;"/></Q><Q
name="product"><V text="&lt;html&gt;&lt;body&gt;firefly
luciferase&lt;/body&gt;&lt;/html&gt;"/></Q><Q name="note"><V
text="&lt;html&gt;&lt;body&gt;enhanced&amp;nbsp;&lt;i&gt;luc+&lt;/i&gt; version of
the luciferase gene&lt;/body&gt;&lt;/html&gt;"/></Q><Q name="gene"><V
text="&lt;html&gt;&lt;body&gt;&lt;i&gt;luc+&lt;/i&gt;&lt;/body&gt;&lt;/html&gt;"/></Q>
<Q name="transl_table"><V int="1"/></Q><Q name="protein_id"><V
text="&lt;html&gt;&lt;body&gt;&lt;br&gt;&lt;/body&gt;&lt;/html&gt;"/></Q><Q
name="translation"><V
text="MEDAKNIKKGPAPFYPLEDGTAGEQLHKAMKRYALVPGTIAFTDAHIEVDITYAEYFEMSVRLAEAMKRYGLNTNHRIVV
CSENSLQFFMPVLGALFIGVAVAPANDIYNERELLNSMGISQPTVVFVSKKGLQKILNVQKKLPIIQKIIIMDSKTDYQGFQSMYT
FVTSHLPPGFNEYDFVPESFDRDKTIALIMNSSGSTGLPKGVALPHRTACVRFSHARDPIFGNQIIPDTAILSVVPFHHGFGMFTT
LGYLICGFRVVLMYRFEEELFLRSLQDYKIQSALLVPTLFSFFAKSTLIDKYDLSNLHEIASGGAPLSKEVGEAVAKRFHLPGIRQ
GYGLTETTSAILITPEGDDKPGAVGKVVPFFEAKVVDLDTGKTLGVNQRGELCVRGPMIMSGYVNNPEATNALIDKDGWLHSGDIA
YWDEDEHFFIVDRLKSLIKYKGYQVAPAELESILLQHPNIFDAGVAGLPDDDAGELPAAVVVLEHGKTMTEKEIVDYVASQVTTAK
KLRGGVVFVDEVPKGLTGKLDARKIREILIKAKKGGKIAV*"/></Q></Feature><Feature recentID="6"
name="AmpR" directionality="2" translationMW="31558.16" type="CDS"
swappedSegmentNumbering="1" allowSegmentOverlaps="0" cleavageArrows="5461"
readingFrame="-1" consecutiveTranslationNumbering="1" hitsStopCodon="1"
detectionMode="exactProteinMatch">&lt;Segment range="4670-5461" color="#ccffcc"
type="standard" translated="1"/>&lt;Segment name="signal sequence" range="5462-5530"
color="#ccffcc" type="standard" translated="1"/><Q name="codon_start"><V
int="1"/></Q><Q name="product"><V
text="&lt;html&gt;&lt;body&gt;尾-lactamase&lt;/body&gt;&lt;/html&gt;"/></Q><Q
name="note"><V text="&lt;html&gt;&lt;body&gt;confers resistance to ampicillin,
carbenicillin, and related antibiotics&lt;/body&gt;&lt;/html&gt;"/></Q><Q
name="gene"><V
text="&lt;html&gt;&lt;body&gt;&lt;i&gt;bla&lt;/i&gt;&lt;/body&gt;&lt;/html&gt;"/></Q><
Q name="transl_table"><V int="1"/></Q><Q name="translation"><V
text="MSIQHFRVALIPFFAAFCLPVFA,HPETLVKVKDAEDQLGARVGYIELDLNSGKILESFRPEERFPMMSTFKVLLCGAVL
SRIDAGQEQLGRRIHYSQNDLVEYSPVTEKHLTDGMTVRELCSAAITMSDNTAANLLLTTIGGPKELTAFLHNMGDHVTRLDRWEP
ELNEAIPNDERDTTMPVAMATTLRKLLTGELLTLASRQQLIDWMEADKVAGPLLRSALPAGWFIADKSGAGERGSRGIIAALGPDG
KPSRIVVIYTTGSQATMDERNRQIAEIGASLIKHW*"/></Q></Feature><Feature recentID="5"
name="ori" directionality="2" type="rep_origin" swappedSegmentNumbering="1"
allowSegmentOverlaps="0" readingFrame="-1"
consecutiveTranslationNumbering="1">&lt;Segment range="3911-4499" color="#ffff00"
type="standard"/><Q name="direction"><V text="LEFT"/></Q><Q name="note"><V
text="&lt;html&gt;&lt;body&gt;high-copy-number ColE1/pMB1/pBR322/pUC origin of
replication&lt;/body&gt;&lt;/html&gt;"/></Q></Feature><Feature recentID="4" name="f1
ori" directionality="1" type="rep_origin" allowSegmentOverlaps="0"
consecutiveTranslationNumbering="1">&lt;Segment range="5662-6117" color="#ffff00"
type="standard"/><Q name="direction"><V text="RIGHT"/></Q><Q name="note"><V
text="&lt;html&gt;&lt;body&gt;f1 bacteriophage origin of replication; arrow
indicates direction of (+) strand
synthesis&lt;/body&gt;&lt;/html&gt;"/></Q></Feature><Feature recentID="3" name="SV40
poly(A) signal" type="polyA_signal" swappedSegmentNumbering="1"
allowSegmentOverlaps="0" readingFrame="-1"
consecutiveTranslationNumbering="1">&lt;Segment range="3371-3492" color="#a6acb3"
type="standard"/><Q name="note"><V text="&lt;html&gt;&lt;body&gt;SV40
polyadenylation signal&lt;/body&gt;&lt;/html&gt;"/></Q></Feature><Feature
recentID="2" name="AmpR promoter" directionality="2" type="promoter"
swappedSegmentNumbering="1" allowSegmentOverlaps="0" readingFrame="-1"
consecutiveTranslationNumbering="1">&lt;Segment range="5531-5635" color="#ffffff"
type="standard"/><Q name="gene"><V
text="&lt;html&gt;&lt;body&gt;&lt;i&gt;bla&lt;/i&gt;&lt;/body&gt;&lt;/html&gt;"/></Q><
/Feature><Feature recentID="1" name="pause site" type="misc_feature"
allowSegmentOverlaps="0" consecutiveTranslationNumbering="1">&lt;Segment
range="6310-6401" color="#a6acb3" type="standard"/><Q name="note"><V
text="&lt;html&gt;&lt;body&gt;RNA polymerase II transcriptional pause signal from
the human&amp;nbsp;伪2 globin
gene&lt;/body&gt;&lt;/html&gt;"/></Q></Feature><Feature recentID="0" name="poly(A)
signal" type="polyA_signal" allowSegmentOverlaps="0"
consecutiveTranslationNumbering="1">&lt;Segment range="6248-6296" color="#a6acb3"
type="standard"/><Q name="note"><V text="&lt;html&gt;&lt;body&gt;synthetic
polyadenylation signal&lt;/body&gt;&lt;/html&gt;"/></Q></Feature></Features>
```

ENQNULNULNULx97<?xml version="1.0"?><Primers nextValidID="0"><HybridizationParams minContinuousMatchLen="10" allowMismatch="1" minMeltingTemperature="40"/></Primers>
ACKNULNULNULxF1<Notes>
<Type>Synthetic</Type>
<ConfirmedExperimentally>0</ConfirmedExperimentally>
<Created>2025.1.21</Created>
<LastModified>2025.1.21</LastModified>
<CreatedBy>111111111</CreatedBy>
<TransformedInto>Unspecified</TransformedInto>
</Notes>

NULNULSOHYNULNULSOHNULSOHNULNULKNULNULNULNULNULNULNULNULNULETXUnique 6+ CuttersNULONULNULdNULNULNULNULNULNULNULNULNULNULNULNULNULSOHSOHSOHSOHNUL
