# Supplementary material for: Dynamic changes in chromatin accessibility reveal the role of NF-Y targeting AURKB in mediating cell cycle during asynchronous oogenesis in the Chinese Alligator (Alligator sinensis)
Source: Front Zool. 2026 Apr 29;23:24. doi: 10.1186/s12983-026-00611-8 (PMC13274144; doi:10.1186/s12983-026-00611-8)
Supplement: Supplementary file 11 — Additional file11 (PDF 137 KB): AURKB MUT-all promoter Sequencing Report. [file 12983_2026_611_MOESM11_ESM.pdf]

```
                                                                  10        20        30        40
                                                               TAGGCTGTCCCCAGTGCAAGTGCAGGTGCCAGAACATTTCTCTATCGAT
G12-B60900-g0347484-6_pgl3-basic-kpni-seqf.ab1(1>442)      →   TAGGCTGTCCCCAGTGCAAGTGCAGGTGCCAGAACATTTCTCTATCGAT

                                                               50        60        70        80        90
                                                               AGGTACCGAGCTCTTACGCGTGCTAGCCCGGGCTCGAGGGAGCAGTGCA
G0347484-6.seq(1>1617)                                     →                                   CTCGAGGGAGCAGTGCA
G12-B60900-g0347484-6_pgl3-basic-kpni-seqf.ab1(1>442)      →   AGGTACCGAGCTCTTACGCGTGCTAGCCCGGGCTCGAGGGAGCAGTGCA

                                                                100       110       120       130       140
                                                               TGTGGCTGCACAGCCTCTCCGCAAGGCAGCAAGACCCATGAGAGTGGAG
G0347484-6.seq(1>1617)                                     →   TGTGGCTGCACAGCCTCTCCGCAAGGCAGCAAGACCCATGAGAGTGGAG
G12-B60900-g0347484-6_pgl3-basic-kpni-seqf.ab1(1>442)      →   TGTGGCTGCACAGCCTCTCCGCAAGGCAGCAAGACCCATGAGAGTGGAG

                                                                 150       160       170       180       190
                                                               CCTGAGCAGTGGATTTAAAACAATTTTTTTTTGGAAAAGTATGTTTTTA
G0347484-6.seq(1>1617)                                     →   CCTGAGCAGTGGATTTAAAACAATTTTTTTTTGGAAAAGTATGTTTTTA
G12-B60900-g0347484-6_pgl3-basic-kpni-seqf.ab1(1>442)      →   CCTGAGCAGTGGATTTAAAACAATTTTTTTTTGGAAAAGTATGTTTTTA

                                                                  200       210       220       230       240
                                                               TTCAAATATTATAAAAGCCTAAGTCTGTCTGTCTGTCTGTAACACTTTA
G0347484-6.seq(1>1617)                                     →   TTCAAATATTATAAAAGCCTAAGTCTGTCTGTCTGTCTGTAACACTTTA
G12-B60900-g0347484-6_pgl3-basic-kpni-seqf.ab1(1>442)      →   TTCAAATATTATAAAAGCCTAAGTCTGTCTGTCTGTCTGTAACACTTTA

                                                                   250       260       270       280       290
                                                               TTTGTGCTCTGATTGGCTGACAAACGTGCAAAGCAGCATTCTCACAGAA
G0347484-6.seq(1>1617)                                     →   TTTGTGCTCTGATTGGCTGACAAACGTGCAAAGCAGCATTCTCACAGAA
G12-B60900-g0347484-6_pgl3-basic-kpni-seqf.ab1(1>442)      →   TTTGTGCTCTGATTGGCTGACAAACGTGCAAAGCAGCATTCTCACAGAA

                                                                    300       310       320       330       340
                                                               GGCAGCCCTCCGCCTGGATGGTGGGGGCAGGGGACCGGGGGGGGGGGAA
G0347484-6.seq(1>1617)                                     →   GGCAGCCCTCCGCCTGGATGGTGGGGGCAGGGGACCGGGGGGGGGGGAA
G12-B60900-g0347484-6_pgl3-basic-kpni-seqf.ab1(1>442)      →   GGCAGCCCTCCGCCTGGATGGTGGGGGCAGGGGACCGGGGGGGGGGGAA
B09-B60900-g0347484-6_g0347484-1-seq1r.ab1(1>366)          ←                                               GGGAA

                                                                     350       360       370       380       390
                                                               GGGCCAGCAGGGCCCCGTCCCCCTGCAGGTAATGCGGGGTGTGGGAGCG
G0347484-6.seq(1>1617)                                     →   GGGCCAGCAGGGCCCCGTCCCCCTGCAGGTAATGCGGGGTGTGGGAGCG
G12-B60900-g0347484-6_pgl3-basic-kpni-seqf.ab1(1>442)      →   GGGCCAGCAGGGCCCCGTCCCCCTGCAGGTAATGCGGGGTGTGGGAGCG
B09-B60900-g0347484-6_g0347484-1-seq1r.ab1(1>366)          ←   GGGCCAGCAGGGCCCCGTCCCCCTGCAGGTAATGCGGGGTGTGGGAGCG

                                                                      400       410       420       430       440
                                                               GGCCCGGGCCCACGGTGGTGGGGAGGGGAGCAGGCAGGACCCAAGCAGC
G0347484-6.seq(1>1617)                                     →   GGCCCGGGCCCACGGTGGTGGGGAGGGGAGCAGGCAGGACCCAAGCAGC
G12-B60900-g0347484-6_pgl3-basic-kpni-seqf.ab1(1>442)      →   GGCCCGGGCCCACGGTGGTGGGGAGGGGAGCAGGCAGGACCCAAGCAGC
B09-B60900-g0347484-6_g0347484-1-seq1r.ab1(1>366)          ←   GGCCCGGGCCCACGGTGGTGGGGAGGGGAGCAGGCAGGACCCAAGCAGC

                                                                       450       460       470       480       490
                                                               AGAAGGGAAGCAGGAGCAGGTCGGGGGGGGGGGAGGGCTGTCCCGCCT
G0347484-6.seq(1>1617)                                     →   AGAAGGGAAGCAGGAGCAGGTCGGGGGGGGGGGAGGGCTGTCCCGCCT
G12-B60900-g0347484-6_pgl3-basic-kpni-seqf.ab1(1>442)      →   A
B09-B60900-g0347484-6_g0347484-1-seq1r.ab1(1>366)          ←   AGAAGGGAAGCAGGAGCAGGTCGGGGGGGGGGGAGGGCTGTCCCGCCT

                                                                        500       510       520       530
                                                               GTCCCTTCACCCCTGTCATTCTTGACAGGCAATTGGCTAGTAGATGCGT
G0347484-6.seq(1>1617)                                     →   GTCCCTTCACCCCTGTCATTCTTGACAGGCAATTGGCTAGTAGATGCGT
B09-B60900-g0347484-6_g0347484-1-seq1r.ab1(1>366)          ←   GTCCCTTCACCCCTGTCATTCTTGACAGGCAATTGGCTAGTAGATGCGT
```

```
                                                           540       550       560       570       580
                                                           AAAAGTTATACGCATCTCCACCTTCTAGTCACCTGCCATCACAGACCTT
G0347484-6.seq(1>1617)                               →     AAAAGTTATACGCATCTCCACCTTCTAGTCACCTGCCATCACAGACCTT
B09-B60900-g0347484-6_g0347484-1-seq1r.ab1(1>366)    ←     AAAAGTTATACGCATCTCCACCTTCTAGTCACCTGCCATCACAGACCTT

                                                             590       600       610       620       630
                                                           CACTCGCACCACAGAGTGCAAAACGCCCGCCCGCCTGCCCGCCCGCGGG
G0347484-6.seq(1>1617)                               →     CACTCGCACCACAGAGTGCAAAACGCCCGCCCGCCTGCCCGCCCGCGGG
B09-B60900-g0347484-6_g0347484-1-seq1r.ab1(1>366)    ←     CACTCGCACCACAGAGTGCAAAACGCCCGCCCGCCTGCCCGCCCGCGGG

                                                               640       650       660       670       680
                                                           CGCCCCGCGCCGGTGCAACCTCCGCCGGCCCGAGCACTGGATTTCTTCT
G0347484-6.seq(1>1617)                               →     CGCCCCGCGCCGGTGCAACCTCCGCCGGCCCGAGCACTGGATTTCTTCT
B09-B60900-g0347484-6_g0347484-1-seq1r.ab1(1>366)    ←     CGCCCCGCGCCGGTGCAACCTCCGCCGGCCCGAGCACTGGATTTCTTCT
B10-B60900-g0347484-6_g0347484-1-seq2.ab1(1>804)     →                 GTGCAaCCTCCGCCGGCCCGAGCACTGGATTTCTTCT

                                                                 690       700       710       720       730
                                                           TGATTTTTAAGGAGATTTTTTCGTGTCCCGGGTCAAATTAGCCCGATCA
G0347484-6.seq(1>1617)                               →     TGATTTTTAAGGAGATTTTTTCGTGTCCCGGGTCAAATTAGCCCGATCA
B09-B60900-g0347484-6_g0347484-1-seq1r.ab1(1>366)    ←     TGATTTtTAAGGAGATTT
B10-B60900-g0347484-6_g0347484-1-seq2.ab1(1>804)     →     TGATTTTTAAGGAGATTTTTTCGTGTCCCGGGTCAAATTAGCCCGATCA

                                                                   740       750       760       770       780
                                                           GGCCCAAATCCATTAGAATCACGCAAGGACCACACGTGGCCCTGCTACC
G0347484-6.seq(1>1617)                               →     GGCCCAAATCCATTAGAATCACGCAAGGACCACACGTGGCCCTGCTACC
B10-B60900-g0347484-6_g0347484-1-seq2.ab1(1>804)     →     GGCCCAAATCCATTAGAATCACGCAAGGACCACACGTGGCCCTGCTACC

                                                                     790       800       810       820       830
                                                           GGCAAGTGTCCCCGCCCCGCCCCGCCCCGCCGCGCCTTCAGAGGCTTCC
G0347484-6.seq(1>1617)                               →     GGCAAGTGTCCCCGCCCCGCCCCGCCCCGCCGCGCCTTCAGAGGCTTCC
B10-B60900-g0347484-6_g0347484-1-seq2.ab1(1>804)     →     GGCAAGTGTCCCCGCCCCGCCCCGCCCCGCCGCGCCTTCAGAGGCTTCC

                                                                       840       850       860       870       880
                                                           AAACCCTGTGACGGCCGGCGTCCCGCGCGGGCCCCAGCCCGAGGCCTCG
G0347484-6.seq(1>1617)                               →     AAACCCTGTGACGGCCGGCGTCCCGCGCGGGCCCCAGCCCGAGGCCTCG
B10-B60900-g0347484-6_g0347484-1-seq2.ab1(1>804)     →     AAACCCTGTGACGGCCGGCGTCCCGCGCGGGCCCCAGCCCGAGGCCTCG

                                                                         890       900       910       920       930
                                                           GCTGCCCCGAGCTTCGCCTGCCCTGGCTGTGCGGCTGCAGGAGCAAGGA
G0347484-6.seq(1>1617)                               →     GCTGCCCCGAGCTTCGCCTGCCCTGGCTGTGCGGCTGCAGGAGCAAGGA
B10-B60900-g0347484-6_g0347484-1-seq2.ab1(1>804)     →     GCTGCCCCGAGCTTCGCCTGCCCTGGCTGTGCGGCTGCAGGAGCAAGGA

                                                                           940       950       960       970       980
                                                           GGGGGAGTCTCCCGGTCCCATAAAGGGCCCTGCAGCCCCTCCTGCGCCA
G0347484-6.seq(1>1617)                               →     GGGGGAGTCTCCCGGTCCCATAAAGGGCCCTGCAGCCCCTCCTGCGCCA
B10-B60900-g0347484-6_g0347484-1-seq2.ab1(1>804)     →     GGGGGAGTCTCCCGGTCCCATAAAGGGCCCTGCAGCCCCTCCTGCGCCA

                                                                             990       1000      1010      1020
                                                           TCCCAACACCGGGGCGGGGGGCTCCCCTGGCTGGGGCCAGACGCCCCCA
G0347484-6.seq(1>1617)                               →     TCCCAACACCGGGGCGGGGGGCTCCCCTGGCTGGGGCCAGACGCCCCCA
B10-B60900-g0347484-6_g0347484-1-seq2.ab1(1>804)     →     TCCCAACACCGGGGCGGGGGGCTCCCCTGGCTGGGGCCAGACGCCCCCA

                                                           1030      1040      1050      1060      1070
                                                           GGGGCCCTCACAGTCCAGGAGGGGGGCGGGCACCCGGGTTTTCGGGGGC
G0347484-6.seq(1>1617)                               →     GGGGCCCTCACAGTCCAGGAGGGGGGCGGGCACCCGGGTTTTCGGGGGC
B10-B60900-g0347484-6_g0347484-1-seq2.ab1(1>804)     →     GGGGCCCTCACAGTCCAGGAGGGGGGCGGGCACCCGGGTTTTCGGGGGC
```

```
                                                           1080      1090      1100      1110      1120
                                                            GGGGGGCAGGGACTCGGGGACCTGAGAGCCCGAGCGGCCCCCAAACAAA
G0347484-6.seq(1>1617)                               →     GGGGGGCAGGGACTCGGGGACCTGAGAGCCCGAGCGGCCCCCAAACAAA
B10-B60900-g0347484-6_g0347484-1-seq2.ab1(1>804)     →     GGGGGGCAGGGACTCGGGGACCTGAGAGCCCGAGCGGCCCCCAAACAAA
B12-B60900-g0347484-6_RVp3-R.ab1(1>663)              ←      GGGGGCAGGGACTCGGGGACCTGAGAGCCCGAGCGGCCCCCAAACAAA

                                                            1130      1140      1150      1160      1170
                                                            CCCAGCCGAAGAAGGCGCCCCGCCACACCGCAACGGTCAATGCCGCTTT
G0347484-6.seq(1>1617)                               →     CCCAGCCGAAGAAGGCGCCCCGCCACACCGCAACGGTCAATGCCGCTTT
B10-B60900-g0347484-6_g0347484-1-seq2.ab1(1>804)     →     CCCAGCCGAAGAAGGCGCCCCGCCACACCGCAACGGTCAATGCCGCTTT
B12-B60900-g0347484-6_RVp3-R.ab1(1>663)              ←     CCCAGCCGAAGAAGGCGCCCCGCCACACCGCAACGGTCAATGCCGCTTT

                                                             1180      1190      1200      1210      1220
                                                            TCTGCGAAAGGGCAATTCCGCTAAGCGGCTTCGGCACCCTCGCACGCGG
G0347484-6.seq(1>1617)                               →     TCTGCGAAAGGGCAATTCCGCTAAGCGGCTTCGGCACCCTCGCACGCGG
B10-B60900-g0347484-6_g0347484-1-seq2.ab1(1>804)     →     TCTGCGAAAGGGCAATTCCGCTAAGCGGCTTCGGCACCCTCGCACGCGG
B12-B60900-g0347484-6_RVp3-R.ab1(1>663)              ←     TCTGCGAAAGGGCAATTCCGCTAAGCGGCTTCGGCACCCTCGCACGCGG

                                                              1230      1240      1250      1260      1270
                                                            AGTCACCACGCCCCGTCTCTGATTGGCTGGCGGCGCCAGCTCCCGGCCC
G0347484-6.seq(1>1617)                               →     AGTCACCACGCCCCGTCTCTGATTGGCTGGCGGCGCCAGCTCCCGGCCC
B10-B60900-g0347484-6_g0347484-1-seq2.ab1(1>804)     →     AGTCACCACGCCCCGTCTCTGATTGGCTGGCGGCGCCAGCTCCCGGCCC
B12-B60900-g0347484-6_RVp3-R.ab1(1>663)              ←     AGTCACCACGCCCCGTCTCTGATTGGCTGGCGGCGCCAGCTCCCGGCCC

                                                               1280      1290      1300      1310      1320
                                                            GCCGTTGGCTGAACTCAACTCAACACCCGCCCCCTACCTCCTCTCCGCC
G0347484-6.seq(1>1617)                               →     GCCGTTGGCTGAACTCAACTCAACACCCGCCCCCTACCTCCTCTCCGCC
B10-B60900-g0347484-6_g0347484-1-seq2.ab1(1>804)     →     GCCGTTGGCTGAACTCAACTCAACACCCGCCCCCTACCTCCTCTCCGCC
B12-B60900-g0347484-6_RVp3-R.ab1(1>663)              ←     GCCGTTGGCTGAACTCAACTCAACACCCGCCCCCTACCTCCTCTCCGCC

                                                                1330      1340      1350      1360      1370
                                                            GTTACCAGGCAGACCAGCTCCCGTGACGCGTTCCGCCCCCGCCCGCTCA
G0347484-6.seq(1>1617)                               →     GTTACCAGGCAGACCAGCTCCCGTGACGCGTTCCGCCCCCGCCCGCTCA
B10-B60900-g0347484-6_g0347484-1-seq2.ab1(1>804)     →     GTTACCAGGCAGACCAGCTCCCGTGACGCGTTCCGCCCCCGCCCGCTCA
B12-B60900-g0347484-6_RVp3-R.ab1(1>663)              ←     GTTACCAGGCAGACCAGCTCCCGTGACGCGTTCCGCCCCCGCCCGCTCA

                                                                 1380      1390      1400      1410      1420
                                                            GATTGACGGGCAGCTCGGCCAACCCGCACTAGGTCCCGCCCCTCCAGTT
G0347484-6.seq(1>1617)                               →     GATTGACGGGCAGCTCGGCCAACCCGCACTAGGTCCCGCCCCTCCAGTT
B10-B60900-g0347484-6_g0347484-1-seq2.ab1(1>804)     →     GATTGACGGGCAGCTCGGCCAACCCGCACTAGGTCCCGCCCCTCCAGTT
B12-B60900-g0347484-6_RVp3-R.ab1(1>663)              ←     GATTGACGGGCAGCTCGGCCAACCCGCACTAGGTCCCGCCCCTCCAGTT

                                                                  1430      1440      1450      1460      1470
                                                            CTTTTGCGCGCGCCTGATTCCGCCCGGAGGGAGGCGGGACTTCTAACTC
G0347484-6.seq(1>1617)                               →     CTTTTGCGCGCGCCTGATTCCGCCCGGAGGGAGGCGGGACTTCTAACTC
B10-B60900-g0347484-6_g0347484-1-seq2.ab1(1>804)     →     CTTTTGCGCGCGCCTGATTCCGCCCGGAGGGA
B12-B60900-g0347484-6_RVp3-R.ab1(1>663)              ←     CTTTTGCGCGCGCCTGATTCCGCCCGGAGGGAGGCGGGACTTCTAACTC

                                                                   1480      1490      1500      1510
                                                            GCCGCGCAGCCGCCACCTCCCGCAGGAGCCAATGGGAGCACAGGCGGCA
G0347484-6.seq(1>1617)                               →     GCCGCGCAGCCGCCACCTCCCGCAGGAGCCAATGGGAGCACAGGCGGCA
B12-B60900-g0347484-6_RVp3-R.ab1(1>663)              ←     GCCGCGCAGCCGCCACCTCCCGCAGGAGCCAATGGGAGCACAGGCGGCA

                                                          1520      1530      1540      1550      1560
                                                            ACGGGCTCGGCCTCCAAGGGGGAAGGGCGGAGGGAGCGCGCCGGCGGGA
G0347484-6.seq(1>1617)                               →     ACGGGCTCGGCCTCCAAGGGGGAAGGGCGGAGGGAGCGCGCCGGCGGGA
B12-B60900-g0347484-6_RVp3-R.ab1(1>663)              ←     ACGGGCTCGGCCTCCAAGGGGGAAGGGCGGAGGGAGCGCGCCGGCGGGA
```

```
                                                      1570      1580      1590      1600      1610
                                                      GCTGCGGTCTAGGGCCGGGACACGGCGGCCGTCGCAGCCAATGGGAGCG
G0347484-6.seq(1>1617)                          →     GCTGCGGTCTAGGGCCGGGACACGGCGGCCGTCGCAGCCAATGGGAGCG
B12-B60900-g0347484-6_RVp3-R.ab1(1>663)         ←     GCTGCGGTCTAGGGCCGGGACACGGCGGCCGTCGCAGCCAATGGGAGCG

                                                      1620      1630      1640      1650      1660
                                                      CGGGGCGGGGTGGATTTGAAGCGCGAGGCGGCAGCAGCAGCCGTGGGTA
G0347484-6.seq(1>1617)                          →     CGGGGCGGGGTGGATTTGAAGCGCGAGGCGGCAGCAGCAGCCGTGGGTA
B12-B60900-g0347484-6_RVp3-R.ab1(1>663)         ←     CGGGGCGGGGTGGATTTGAAGCGCGAGGCGGCAGCAGCAGCCGTGGGTA

                                                      1670      1680      1690      1700      1710
                                                      GGTGAGCGCGGAGGTACCGGGGGCGCAAGCTTGGCATTCCGGTACTGTT
G0347484-6.seq(1>1617)                          →     GGTGAGCGCGGAGGTACCGGGGGCGCAAGCTT
B12-B60900-g0347484-6_RVp3-R.ab1(1>663)         ←     GGTGAGCGCGGAGGTACCGGGGGCGCAAGCTTGGCATTCCGGTACTGTT

                                                      1720      1730      1740
                                                      GGTAAAGCCACCATGGAAGACGCCAAA
B12-B60900-g0347484-6_RVp3-R.ab1(1>663)         ←     GGTAAAGCCACCATGGAAGACGCCAAA
```
