# Supplementary material for: Dynamic changes in chromatin accessibility reveal the role of NF-Y targeting AURKB in mediating cell cycle during asynchronous oogenesis in the Chinese Alligator (Alligator sinensis)
Source: Front Zool. 2026 Apr 29;23:24. doi: 10.1186/s12983-026-00611-8 (PMC13274144; doi:10.1186/s12983-026-00611-8)
Supplement: Supplementary file 12 — Additional file12 (PDF 182 KB): AURKB MUT-all promoter Target Gene Sequence. [file 12983_2026_611_MOESM12_ESM.pdf]

AURKB MUT-all promoter Target Gene Sequence.seq

5' CTCGAGGGAGCAGTGCATGTGGCTGCACAGCCTCTCCGCAAGGCAGCAAGACCCATGAGAGTGGAGCCTG 70

5' AGCAGTGGATTTAAAACAATTTTTTTTTGGAAAAGTATGTTTTTATTCAAATATTATAAAAGCCTAAGTC 140

5' TGTCTGTCTGTCTGTAACACTTTATTTGTGCTCTGATTGGCTGACAAACGTGCAAAGCAGCATTCTCACA 210

5' GAAGGCAGCCCTCCGCCTGGATGGTGGGGGCAGGGGACCGGGGGGGGGGGAAGGGCCAGCAGGGCCCCGT 280

5' CCCCCTGCAGGTAATGCGGGGTGTGGGAGCGGGCCCGGGCCCACGGTGGTGGGGAGGGGAGCAGGCAGGA 350

5' CCCAAGCAGCAGAAGGGAAGCAGGAGCAGGTCGGGGGGGGGGGGAGGGCTGTCCCGCCTGTCCCTTCACC 420

5' CCTGTCATTCTTGACAGGCAATTGGCTAGTAGATGCGTAAAAGTTATACGCATCTCCACCTTCTAGTCAC 490

5' CTGCCATCACAGACCTTCACTCGCACCACAGAGTGCAAAACGCCCGCCCGCCTGCCCGCCCGCGGGCGCC 560

5' CCGCGCCGGTGCAACCTCCGCCGGCCCGAGCACTGGATTTCTTCTTGATTTTTAAGGAGATTTTTTCGTG 630

5' TCCCGGGTCAAATTAGCCCGATCAGGCCCAAATCCATTAGAATCACGCAAGGACCACACGTGGCCCTGCT 700

5' ACCGGCAAGTGTCCCCGCCCCGCCCCGCCCCGCCGCGCCTTCAGAGGCTTCCAAACCCTGTGACGGCCGG 770

5' CGTCCCGCGCGGGCCCCAGCCCGAGGCCTCGGCTGCCCCGAGCTTCGCCTGCCCTGGCTGTGCGGCTGCA 840

5' GGAGCAAGGAGGGGGAGTCTCCCGGTCCCATAAAGGGCCCTGCAGCCCCTCCTGCGCCATCCCAACACCG 910

5' GGGCGGGGGGCTCCCCTGGCTGGGGCCAGACGCCCCCAGGGGCCCTCACAGTCCAGGAGGGGGGCGGGCA 980

5' CCCGGGTTTTCGGGGGCGGGGGGCAGGGACTCGGGGACCTGAGAGCCCGAGCGGCCCCCAAACAAACCCA 1050

5' GCCGAAGAAGGCGCCCCGCCACACCGCAACGGTCAATGCCGCTTTTCTGCGAAAGGGCAATTCCGCTAAG 1120

5' CGGCTTCGGCACCCTCGCACGCGGAGTCACCACGCCCCGTCTCTGATTGGCTGGCGGCGCCAGCTCCCGG 1190

5' CCCGCCGTTGGCTGAACTCAACTCAACACCCGCCCCCTACCTCCTCTCCGCCGTTACCAGGCAGACCAGC 1260

5' TCCCGTGACGCGTTCCGCCCCCGCCCGCTCAGATTGACGGGCAGCTCGGCCAACCCGCACTAGGTCCCGC 1330

5' CCCTCCAGTTCTTTTGCGCGCGCCTGATTCCGCCCGGAGGGAGGCGGGACTTCTAACTCGCCGCGCAGCC 1400

5' GCCACCTCCCGCAGGAGCCAATGGGAGCACAGGCGGCAACGGGCTCGGCCTCCAAGGGGGAAGGGCGGAG 1470

5' GGAGCGCGCCGGCGGGAGCTGCGGTCTAGGGCCGGGACACGGCGGCCGTCGCAGCCAATGGGAGCGCGGG 1540

5' GCGGGGTGGATTTGAAGCGCGAGGCGGCAGCAGCAGCCGTGGGTAGGTGAGCGCGGAGGTACCGGGGGCG 1610

5' CAAGCTT 1617
