# Supplementary material for: Dynamic changes in chromatin accessibility reveal the role of NF-Y targeting AURKB in mediating cell cycle during asynchronous oogenesis in the Chinese Alligator (Alligator sinensis)
Source: Front Zool. 2026 Apr 29;23:24. doi: 10.1186/s12983-026-00611-8 (PMC13274144; doi:10.1186/s12983-026-00611-8)
Supplement: Supplementary file 13 — Additional file13 (PDF 94 KB): AURKB WT promoter in pGL3-Basic. [file 12983_2026_611_MOESM13_ESM.pdf]

```
LOCUS       Exported                6441 bp ds-DNA    circular SYN 11-1月-2025
DEFINITION  .
ACCESSION   .
VERSION     .
KEYWORDS    Untitled 41
SOURCE      synthetic DNA construct
  ORGANISM  synthetic DNA construct
REFERENCE   1  (bases 1 to 6441)
  AUTHORS   111111111
  TITLE     Direct Submission
  JOURNAL   Exported 2025年1月11日 from SnapGene 2.3.2
            http://www.snapgene.com
FEATURES             Location/Qualifiers
     source          1..6441
                     /organism="synthetic DNA construct"
                     /mol_type="other DNA"
     misc_feature    32..1681
                     /note="AURKB WT promoter"
     CDS             1711..3363
                     /codon_start=1
                     /gene="luc+"
                     /product="firefly luciferase"
                     /EC_number="
                     "
                     /note="luciferase"
                     /note="enhanced luc+ version of the luciferase gene"
                     /protein_id="
                     "
                     /translation="MEDAKNIKKGPAPFYPLEDGTAGEQLHKAMKRYALVPGTIAFTDA
                     HIEVDITYAEYFEMSVRLAEAMKRYGLNTNHRIVVCSENSLQFFMPVLGALFIGVAVAP
                     ANDIYNERELLNSMGISQPTVVFVSKKGLQKILNVQKKLPIIQKIIIMDSKTDYQGFQS
                     MYTFVTSHLPPGFNEYDFVPESFDRDKTIALIMNSSGSTGLPKGVALPHRTACVRFSHA
                     RDPIFGNQIIPDTAILSVVPFHHGFGMFTTLGYLICGFRVVLMYRFEEELFLRSLQDYK
                     IQSALLVPTLFSFFAKSTLIDKYDLSNLHEIASGGAPLSKEVGEAVAKRFHLPGIRQGY
                     GLTETTSAILITPEGDDKPGAVGKVVPFFEAKVVDLDTGKTLGVNQRGELCVRGPMIMS
                     GYVNNPEATNALIDKDGWLHSGDIAYWDEDEHFFIVDRLKSLIKYKGYQVAPAELESIL
                     LQHPNIFDAGVAGLPDDDAGELPAAVVVLEHGKTMTEKEIVDYVASQVTTAKKLRGGVV
                     FVDEVPKGLTGKLDARKIREILIKAKKGGKIAV"
     polyA_signal    3404..3525
                     /note="SV40 poly(A) signal"
                     /note="SV40 polyadenylation signal"
     rep_origin      complement(3944..4532)
                     /direction=LEFT
                     /note="ori"
                     /note="high-copy-number ColE1/pMB1/pBR322/pUC origin of
                     replication"
     CDS             complement(4703..5563)
                     /codon_start=1
                     /gene="bla"
                     /product="beta-lactamase"
                     /note="AmpR"
                     /note="confers resistance to ampicillin, carbenicillin, and
                     related antibiotics"
                     /translation="MSIQHFRVALIPFFAAFCLPVFAHPETLVKVKDAEDQLGARVGYI
                     ELDLNSGKILESFRPEERFPMMSTFKVLLCGAVLSRIDAGQEQLGRRIHYSQNDLVEYS
                     PVTEKHLTDGMTVRELCSAAITMSDNTAANLLLTTIGGPKELTAFLHNMGDHVTRLDRW
                     EPELNEAIPNDERDTTMPVAMATTLRKLLTGELLTLASRQQLIDWMEADKVAGPLLRSA
                     LPAGWFIADKSGAGERGSRGIIAALGPDGKPSRIVVIYTTGSQATMDERNRQIAEIGAS
                     LIKHW"
     promoter        complement(5564..5668)
                     /gene="bla"
                     /note="AmpR promoter"
     rep_origin      5695..6150
                     /direction=RIGHT
                     /note="f1 ori"
                     /note="f1 bacteriophage origin of replication; arrow
                     indicates direction of (+) strand synthesis"
     polyA_signal    6281..6329
                     /note="synthetic polyadenylation signal"
     misc_feature    6343..6434
                     /note="pause site"
                     /note="RNA polymerase II transcriptional pause signal from
                     the human alpha-2 globin gene"
```

```
ORIGIN
        1 ggtaccgagc tcttacgcgt gctagcccgg gctcgaggga gcagtgcatg tggctgcaca
       61 gcctctccgc aaggcagcaa gacccatgag agtggagcct gagcagtgga tttaaaacaa
      121 tttttttttg gaaaagtatg tttttattca aatattataa aagcctaagt ctgtctgtct
      181 gtctgtaaca ctttatttgt gctctgattg gctgacaaac agccaatcag agtgcaaagc
      241 agcattctca cagaaggcag ccctccgcct ggatggtggg ggcaggggac cggggggggg
      301 ggaagggcca gcagggcccc gtccccctgc aggtaatgcg ggtgtgggga gcgggcccgg
      361 gcccacggtg gtggggaggg gagcaggcag gacccaagca gcagaaggga agcaggagca
      421 ggtcgggggg ggggggaggg ctgtcccgcc tgtcccttca cccctgtcat tcttgacagg
      481 caattggcta gtagatgcgt aaaagttata cgcatctcca ccttctagtc acctgccatc
      541 acagaccttc actcgcacca cagagtgcaa aacgcccgcc cgcctgcccg cccgcgggcg
      601 ccccgcgccg gtgcaacctc cgccggcccg agcactggat ttcttcttga tttttaagga
      661 gatttttttcg tgtcccgggt caaattagcc cgatcaggcc caaatccatt agaatcacgc
      721 aaggaccaca cgtggccctg ctaccggcaa gtgtccccgc cccgccccgc cccgccgcgc
      781 cttcagaggc ttccaaaccc tgtgacggcc ggcgtcccgc gcgggcccca gcccgaggcc
      841 tcggctgccc cgagcttcgc ctgccctggc tgtgcggctg caggagcaag gaggggggagt
      901 ctcccggtcc cataaagggc cctgcagccc ctcctgcgcc atcccaacac cggggcgggg
      961 ggctcccctg gctggggcca gacgccccca ggggccctca cagtccagga gggggggcggg
     1021 cacccgggtt ttcgggggcg gggggcaggg actcggggac ctgagagccc gagcggcccc
     1081 caaacaaacc cagccgaaga aggcgccccg ccacaccgca acggtcaatg ccgcttttct
     1141 gcgaaagggc aattccgcta agcggcttcg gcaccctcgc acgcggagtc accacgcccc
     1201 gtctctgatt ggctggcggc gccagctccc ggcccgccgt tggctgaact caactcaaca
     1261 cccgccccct acctcctctc cgccgttacc aggcagacca gctcccgtga cgcgttccgc
     1321 ccccgcccgc tcagattgac gggcagctcg gccaacccgc actaggtccc gcccctccag
     1381 ttcttttgcg cgcgcctgat tccgcccgga gggaggcggg acttctaact cgccgcgccg
     1441 ccaatccgaa gccgccacct cccgcaggag ccaatgggag cacaggcggc aacgggctcg
     1501 gcctccaagg gggaagggcg gagggagcgc gcggccaatg aaacggcggg agctgcggtc
     1561 tagggccggg acacggcggc cgtcgcagcc aatgggagcg cggggcgggg tggatttgaa
     1621 gcgcgaggcg gcagcagcag ccgtgggtag gtgagcgcgg aggtaccggg ggcgcaagct
     1681 tggcattccg gtactgttgg taaagccacc atggaagacg ccaaaaacat aaagaaaggc
     1741 ccggcgccat tctatccgct ggaagatgga accgctggag agcaactgca taaggctatg
     1801 aagagatacg ccctggttcc tggaacaatt gcttttacag atgcacatat cgaggtggac
     1861 atcacttacg ctgagtactt cgaaatgtcc gttcggttgg cagaagctat gaaacgatat
     1921 gggctgaata caaatcacag aatcgtcgta tgcagtgaaa actctcttca attctttatg
     1981 ccggtgttgg gcgcgttatt tatcggagtt gcagttgcgc ccgcgaacga catttataat
     2041 gaacgtgaat tgctcaacag tatgggcatt tcgcagccta ccgtggtgtt cgtttccaaa
     2101 aaggggttgc aaaaaatttt gaacgtgcaa aaaaagctcc caatcatcca aaaaattatt
     2161 atcatggatt ctaaaacgga ttaccaggga tttcagtcga tgtacacgtt cgtcacatct
     2221 catctacctc ccggttttaa tgaatacgat tttgtgccag agtccttcga tagggacaag
     2281 acaattgcac tgatcatgaa ctcctctgga tctactggtc tgcctaaagg tgtcgctctg
     2341 cctcatagaa ctgcctgcgt gagattctcg catgccagag atcctatttt tggcaatcaa
     2401 atcattccgg atactgcgat tttaagtgtt gttccattcc atcacggttt tggaatgttt
     2461 actacactcg gatatttgat atgtggattt cgagtcgtct taatgtatag atttgaagaa
     2521 gagctgtttc tgaggagcct tcaggattac aagattcaaa gtgcgctgct ggtgccaacc
     2581 ctattctcct tcttcgccaa aagcactctg attgacaaat acgatttatc taatttacac
     2641 gaaattgctt ctggtggcgc tcccctctct aaggaagtcg gggaagcggt tgccaagagg
     2701 ttccatctgc caggtatcag gcaaggatat gggctcactg agactacatc agctattctg
     2761 attacacccg agggggatga taaaccgggc gcggtcggta aagttgttcc attttttgaa
     2821 gcgaaggttg tggatctgga taccgggaaa acgctgggcg ttaatcaaag aggcgaactg
     2881 tgtgtgagag gtcctatgat tatgtccggt tatgtaaaca atccggaagc gaccaacgcc
     2941 ttgattgaca aggatggatg gctacattct ggagacatag cttactggga cgaagacgaa
     3001 cacttcttca tcgttgaccg cctgaagtct ctgattaagt acaaaggcta tcaggtggct
     3061 cccgctgaat tggaatccat cttgctccaa caccccaaca tcttcgacgc aggtgtcgca
     3121 ggtcttcccg acgatgacgc cggtgaactt cccgccgccg ttgttgtttt ggagcacgga
     3181 aagacgatga cggaaaaaga gatcgtggat tacgtcgcca gtcaagtaac aaccgcgaaa
     3241 aagttgcgcg gaggagttgt gtttgtggac gaagtaccga aaggtcttac cggaaaactc
     3301 gacgcaagaa aaatcagaga gatcctcata aaggccaaga agggcggaaa gatcgccgtg
     3361 taattctaga gtcggggcgg ccggccgctt cgagcagaca tgataagata cattgatgag
     3421 tttggacaaa ccacaactag aatgcagtga aaaaaatgct ttatttgtga aatttgtgat
     3481 gctattgctt tatttgtaac cattataagc tgcaataaac aagttaacaa caacaattgc
     3541 attcatttta tgtttcaggt tcagggggag gtgtgggagg ttttttaaag caagtaaaac
     3601 ctctacaaat gtggtaaaat cgataaggat ccgtcgaccg atgcccttga gagccttcaa
     3661 cccagtcagc tccttccggt gggcgcgggg catgactatc gtcgccgcac ttatgactgt
     3721 cttctttatc atgcaactcg taggacaggt gccggcagcg ctcttccgct tcctcgctca
     3781 ctgactcgct gcgctcggtc gttcggctgc ggcgagcggt atcagctcac tcaaaggcgg
     3841 taatacggtt atccacagaa tcaggggata acgcaggaaa gaacatgtga gcaaaaggcc
     3901 agcaaaaggc caggaaccgt aaaaaggccg cgttgctggc gtttttccat aggctccgcc
     3961 cccctgacga gcatcacaaa aatcgacgct caagtcagag gtggcgaaac ccgacaggac
     4021 tataaagata ccaggcgttt ccccctggaa gctccctcgt gcgctctcct gttccgaccc
     4081 tgccgcttac cggatacctg tccgcctttc tcccttcggg aagcgtggcg ctttctcata
     4141 gctcacgctg taggtatctc agttcggtgt aggtcgttcg ctccaagctg ggctgtgtgc
     4201 acgaaccccc cgttcagccc gaccgctgcg ccttatccgg taactatcgt cttgagtcca
     4261 acccggtaag acacgactta tcgccactgg cagcagccac tggtaacagg attagcagag
```

```
     4321 cgaggtatgt aggcggtgct acagagttct tgaagtggtg gcctaactac ggctacacta
     4381 gaagaacagt atttggtatc tgcgctctgc tgaagccagt taccttcgga aaaagagttg
     4441 gtagctcttg atccggcaaa caaaccaccg ctggtagcgg tggttttttt gtttgcaagc
     4501 agcagattac gcgcagaaaa aaaggatctc aagaagatcc tttgatcttt tctacggggt
     4561 ctgacgctca gtggaacgaa aactcacgtt aagggatttt ggtcatgaga ttatcaaaaa
     4621 ggatcttcac ctagatcctt ttaaattaaa aatgaagttt taaatcaatc taaagtatat
     4681 atgagtaaac ttggtctgac agttaccaat gcttaatcag tgaggcacct atctcagcga
     4741 tctgtctatt tcgttcatcc atagttgcct gactccccgt cgtgtagata actacgatac
     4801 gggagggctt accatctggc cccagtgctg caatgatacc gcgagaccca cgctcaccgg
     4861 ctccagattt atcagcaata aaccagccag ccggaagggc cgagcgcaga agtggtcctg
     4921 caactttatc cgcctccatc cagtctatta attgttgccg ggaagctaga gtaagtagtt
     4981 cgccagttaa tagtttgcgc aacgttgttg ccattgctac aggcatcgtg gtgtcacgct
     5041 cgtcgtttgg tatggcttca ttcagctccg gttcccaacg atcaaggcga gttacatgat
     5101 cccccatgtt gtgcaaaaaa gcggttagct ccttcggtcc tccgatcgtt gtcagaagta
     5161 agttggccgc agtgttatca ctcatggtta tggcagcact gcataattct cttactgtca
     5221 tgccatccgt aagatgcttt tctgtgactg gtgagtactc aaccaagtca ttctgagaat
     5281 agtgtatgcg gcgaccgagt tgctcttgcc cggcgtcaat acgggataat accgcgccac
     5341 atagcagaac tttaaaagtg ctcatcattg gaaaacgttc ttcggggcga aaactctcaa
     5401 ggatcttacc gctgttgaga tccagttcga tgtaacccac tcgtgcaccc aactgatctt
     5461 cagcatcttt tactttcacc agcgtttctg ggtgagcaaa aacaggaagg caaaatgccg
     5521 caaaaaaggg aataagggcg acacggaaat gttgaatact catactcttc ctttttcaat
     5581 attattgaag catttatcag ggttattgtc tcatgagcgg atacatattt gaatgtattt
     5641 agaaaaataa acaaataggg gttccgcgca catttccccg aaaagtgcca cctgacgcgc
     5701 cctgtagcgg cgcattaagc gcggcgggtg tggtggttac gcgcagcgtg accgctacac
     5761 ttgccagcgc cctagcgccc gctcctttcg ctttcttccc ttcctttctc gccacgttcg
     5821 ccggctttcc ccgtcaagct ctaaatcggg ggctcccttt agggttccga tttagtgctt
     5881 tacggcacct cgaccccaaa aaacttgatt agggtgatgg ttcacgtagt gggccatcgc
     5941 cctgatagac ggtttttcgc cctttgacgt tggagtccac gttctttaat agtggactct
     6001 tgttccaaac tggaacaaca ctcaacccta tctcggtcta ttcttttgat ttataaggga
     6061 ttttgccgat ttcggcctat tggttaaaaa atgagctgat ttaacaaaaa tttaacgcga
     6121 attttaacaa aatattaacg cttacaattt gccattcgcc attcaggctg cgcaactgtt
     6181 gggaagggcg atcggtgcgg gcctcttcgc tattacgcca gcccaagcta ccatgataag
     6241 taagtaatat taaggtacgg gaggtacttg gagcggccgc aataaaatat ctttattttc
     6301 attacatctg tgtgttggtt ttttgtgtga atcgatagta ctaacatacg ctctccatca
     6361 aaacaaaacg aaacaaaaca aactagcaaa ataggctgtc cccagtgcaa gtgcaggtgc
     6421 cagaacattt ctctatcgat a
//
```
