# Supplementary material for: Dynamic changes in chromatin accessibility reveal the role of NF-Y targeting AURKB in mediating cell cycle during asynchronous oogenesis in the Chinese Alligator (Alligator sinensis)
Source: Front Zool. 2026 Apr 29;23:24. doi: 10.1186/s12983-026-00611-8 (PMC13274144; doi:10.1186/s12983-026-00611-8)
Supplement: Supplementary file 14 — Additional file14 (PDF 145 KB): AURKB WT promoter Sequencing Report. [file 12983_2026_611_MOESM14_ESM.pdf]

```
                                                                          10        20        30        40
                                                                 GTTGGTTTTTTGTGTGAATCGATAGTACTAACATACGCTCTCCATCAAA
A03-B57450-G0347484-1_G0347484-1-SEQ1R.ab1(1>792)            ←   GTTGGTTTTTTGTGTGAATCGATAGTACTAACATACGCTCTCCATCAAA

                                                                 50        60        70        80        90
                                                                 ACAAAACGAAACAAAACAAACTAGCAAAATAGGCTGTCCCCAGTGCAAG
A03-B57450-G0347484-1_G0347484-1-SEQ1R.ab1(1>792)            ←   ACAAAACGAAACAAAACAAACTAGCAAAATAGGCTGTCCCCAGTGCAAG
H08-B57450-G0347484-1_PGL3-BASIC-KPNI-SEQF.ab1(1>339)        →                               AATAGGCTGTCCCCAGTGCAAG

                                                                  100       110       120       130       140
                                                                 TGCAGGTGCCAGAACATTTCTCTATCGATAGGTACCGAGCTCTTACGCG
A03-B57450-G0347484-1_G0347484-1-SEQ1R.ab1(1>792)            ←   TGCAGGTGCCAGAACATTTCTCTATCGATAGGTACCGAGCTCTTACGCG
H08-B57450-G0347484-1_PGL3-BASIC-KPNI-SEQF.ab1(1>339)        →   TGCAGGTGCCAGAACATTTCTCTATCGATAGGTACCGAGCTCTTACGCG

                                                                   150       160       170       180       190
                                                                 TGCTAGCCCGGGCTCGAGGGAGCAGTGCATGTGGCTGCACAGCCTCTCC
G0347484-1.seq(1>1650)                                       →               CTCGAGGGAGCAGTGCATGTGGCTGCACAGCCTCTCC
A03-B57450-G0347484-1_G0347484-1-SEQ1R.ab1(1>792)            ←   TGCTAGCCCGGGCTCGAGGGAGCAGTGCATGTGGCTGCACAGCCTCTCC
H08-B57450-G0347484-1_PGL3-BASIC-KPNI-SEQF.ab1(1>339)        →   TGCTAGCCCGGGCTCGAGGGAGCAGTGCATGTGGCTGCACAGCCTCTCC

                                                                    200       210       220       230       240
                                                                 GCAAGGCAGCAAGACCCATGAGAGTGGAGCCTGAGCAGTGGATTTAAAA
G0347484-1.seq(1>1650)                                       →   GCAAGGCAGCAAGACCCATGAGAGTGGAGCCTGAGCAGTGGATTTAAAA
A03-B57450-G0347484-1_G0347484-1-SEQ1R.ab1(1>792)            ←   GCAAGGCAGCAAGACCCATGAGAGTGGAGCCTGAGCAGTGGATTTAAAA
H08-B57450-G0347484-1_PGL3-BASIC-KPNI-SEQF.ab1(1>339)        →   GCAAGGCAGCAAGACCCATGAGAGTGGAGCCTGAGCAGTGGATTTAAAA

                                                                     250       260       270       280       290
                                                                 CAATTTTTTTTTGGAAAAGTATGTTTTTATTCAAATATTATAAAAGCCT
G0347484-1.seq(1>1650)                                       →   CAATTTTTTTTTGGAAAAGTATGTTTTTATTCAAATATTATAAAAGCCT
A03-B57450-G0347484-1_G0347484-1-SEQ1R.ab1(1>792)            ←   CAATTTTTTTTTGGAAAAGTATGTTTTTATTCAAATATTATAAAAGCCT
H08-B57450-G0347484-1_PGL3-BASIC-KPNI-SEQF.ab1(1>339)        →   CAATTTTTTTTTGGAAAAGTATGTTTTTATTCAAATATTATAAAAGCCT

                                                                      300       310       320       330       340
                                                                 AAGTCTGTCTGTCTGTCTGTAACACTTTATTTGTGCTCTGATTGGCTGA
G0347484-1.seq(1>1650)                                       →   AAGTCTGTCTGTCTGTCTGTAACACTTTATTTGTGCTCTGATTGGCTGA
A03-B57450-G0347484-1_G0347484-1-SEQ1R.ab1(1>792)            ←   AAGTCTGTCTGTCTGTCTGTAACACTTTATTTGTGCTCTGATTGGCTGA
H08-B57450-G0347484-1_PGL3-BASIC-KPNI-SEQF.ab1(1>339)        →   AAGTCTGTCTGTCTGTCTGTAACACTTTATTTGTGCTCTGATTGGCTGA

                                                                       350       360       370       380       390
                                                                 CAAACAGCCAATCAGAGTGCAAAGCAGCATTCTCACAGAAGGCAGCCCT
G0347484-1.seq(1>1650)                                       →   CAAACAGCCAATCAGAGTGCAAAGCAGCATTCTCACAGAAGGCAGCCCT
A03-B57450-G0347484-1_G0347484-1-SEQ1R.ab1(1>792)            ←   CAAACAGCCAATCAGAGTGCAAAGCAGCATTCTCACAGAAGGCAGCCCT
H08-B57450-G0347484-1_PGL3-BASIC-KPNI-SEQF.ab1(1>339)        →   CAAACAGCCAATCAGAGTGCAAAGCAGCATTCTCACAGAAGGCAGCCCT

                                                                        400       410       420       430       440
                                                                 CCGCCTGGATGGTGGGGGCAGGGGACCGGGGGGGGGGAAGGGCCAGCA
G0347484-1.seq(1>1650)                                       →   CCGCCTGGATGGTGGGGGCAGGGGACCGGGGGGGGGGAAGGGCCAGCA
A03-B57450-G0347484-1_G0347484-1-SEQ1R.ab1(1>792)            ←   CCGCCTGGATGGTGGGGGCAGGGGACCGGGGGGGGGGAAGGGCCAGCA
H08-B57450-G0347484-1_PGL3-BASIC-KPNI-SEQF.ab1(1>339)        →   CCGCCTGGATGGTGGGGGCAGGG

                                                                         450       460       470       480       490
                                                                 GGGCCCCGTCCCCCTGCAGGTAATGCGGGGTGTGGGAGCGGGCCCGGGC
G0347484-1.seq(1>1650)                                       →   GGGCCCCGTCCCCCTGCAGGTAATGCGGGGTGTGGGAGCGGGCCCGGGC
A03-B57450-G0347484-1_G0347484-1-SEQ1R.ab1(1>792)            ←   GGGCCCCGTCCCCCTGCAGGTAATGCGGGGTGTGGGAGCGGGCCCGGGC

                                                                          500       510       520       530
                                                                 CCACGGTGGTGGGGAGGGGAGCAGGCAGGACCCAAGCAGCAGAAGGGAA
G0347484-1.seq(1>1650)                                       →   CCACGGTGGTGGGGAGGGGAGCAGGCAGGACCCAAGCAGCAGAAGGGAA
A03-B57450-G0347484-1_G0347484-1-SEQ1R.ab1(1>792)            ←   CCACGGTGGTGGGGAGGGGAGCAGGCAGGACCCAAGCAGCAGAAGGGAA
```

```
                                                        540       550       560       570       580
                                                        GCAGGAGCAGGTCGGGGGGGGGGGGAGGGCTGTCCCGCCTGTCCCTTCA
G0347484-1.seq(1>1650)                               →  GCAGGAGCAGGTCGGGGGGGGGGGGAGGGCTGTCCCGCCTGTCCCTTCA
A03-B57450-G0347484-1_G0347484-1-SEQ1R.ab1(1>792)    ←  GCAGGAGCAGGTCGGGGGGGGGGGGAGGGCTGTCCCGCCTGTCCCTTCA

                                                         590       600       610       620       630
                                                        CCCCTGTCATTCTTGACAGGCAATTGGCTAGTAGATGCGTAAAAGTTAT
G0347484-1.seq(1>1650)                               →  CCCCTGTCATTCTTGACAGGCAATTGGCTAGTAGATGCGTAAAAGTTAT
A03-B57450-G0347484-1_G0347484-1-SEQ1R.ab1(1>792)    ←  CCCCTGTCATTCTTGACAGGCAATTGGCTAGTAGATGCGTAAAAGTTAT

                                                          640       650       660       670       680
                                                        ACGCATCTCCACCTTCTAGTCACCTGCCATCACAGACCTTCACTCGCAC
G0347484-1.seq(1>1650)                               →  ACGCATCTCCACCTTCTAGTCACCTGCCATCACAGACCTTCACTCGCAC
A03-B57450-G0347484-1_G0347484-1-SEQ1R.ab1(1>792)    ←  ACGCATCTCCACCTTCTAGTCACCTGCCATCACAGACCTTCACTCGCAC

                                                           690       700       710       720       730
                                                        CACAGAGTGCAAAACGCCCGCCCGCCTGCCCGCCCGCGGGCGCCCCGCG
G0347484-1.seq(1>1650)                               →  CACAGAGTGCAAAACGCCCGCCCGCCTGCCCGCCCGCGGGCGCCCCGCG
A03-B57450-G0347484-1_G0347484-1-SEQ1R.ab1(1>792)    ←  CACAGAGTGCAAAACGCCCGCCCGCCTGCCCGCCCGCGGGCGCCCCGCG

                                                            740       750       760       770       780
                                                        CCGGTGCAACCTCCGCCGGCCCGAGCACTGGATTTCTTCTTGATTTTTA
G0347484-1.seq(1>1650)                               →  CCGGTGCAACCTCCGCCGGCCCGAGCACTGGATTTCTTCTTGATTTTTA
A03-B57450-G0347484-1_G0347484-1-SEQ1R.ab1(1>792)    ←  CCGGTGCAACCTCCGCCGGCCCGAGCACTGGATTTCTTCTTGATTTTTA
A04-B57450-G0347484-1_G0347484-1-SEQ2.ab1(1>791)     →          ACCTCCGCCGGCCCGAGCACTGGATTTCTTCTTGATTTTTA

                                                             790       800       810       820       830
                                                        AGGAGATTTTTTCGTGTCCCGGGTCAAATTAGCCCGATCAGGCCCAAAT
G0347484-1.seq(1>1650)                               →  AGGAGATTTTTTCGTGTCCCGGGTCAAATTAGCCCGATCAGGCCCAAAT
A03-B57450-G0347484-1_G0347484-1-SEQ1R.ab1(1>792)    ←  AGGAGATT
A04-B57450-G0347484-1_G0347484-1-SEQ2.ab1(1>791)     →  AGGAGATTTTTTCGTGTCCCGGGTCAAATTAGCCCGATCAGGCCCAAAT

                                                              840       850       860       870       880
                                                        CCATTAGAATCACGCAAGGACCACACGTGGCCCTGCTACCGGCAAGTGT
G0347484-1.seq(1>1650)                               →  CCATTAGAATCACGCAAGGACCACACGTGGCCCTGCTACCGGCAAGTGT
A04-B57450-G0347484-1_G0347484-1-SEQ2.ab1(1>791)     →  CCATTAGAATCACGCAAGGACCACACGTGGCCCTGCTACCGGCAAGTGT

                                                               890       900       910       920       930
                                                        CCCCGCCCCGCCCCGCCCCGCCGCGCCTTCAGAGGCTTCCAAACCCTGT
G0347484-1.seq(1>1650)                               →  CCCCGCCCCGCCCCGCCCCGCCGCGCCTTCAGAGGCTTCCAAACCCTGT
A04-B57450-G0347484-1_G0347484-1-SEQ2.ab1(1>791)     →  CCCCGCCCCGCCCCGCCCCGCCGCGCCTTCAGAGGCTTCCAAACCCTGT

                                                                940       950       960       970       980
                                                        GACGGCCGGCGTCCCGCGCGGGCCCCAGCCCGAGGCCTCGGCTGCCCCG
G0347484-1.seq(1>1650)                               →  GACGGCCGGCGTCCCGCGCGGGCCCCAGCCCGAGGCCTCGGCTGCCCCG
A04-B57450-G0347484-1_G0347484-1-SEQ2.ab1(1>791)     →  GACGGCCGGCGTCCCGCGCGGGCCCCAGCCCGAGGCCTCGGCTGCCCCG

                                                                 990      1000      1010      1020
                                                        AGCTTCGCCTGCCCTGGCTGTGCGGCTGCAGGAGCAAGGAGGGGGAGTC
G0347484-1.seq(1>1650)                               →  AGCTTCGCCTGCCCTGGCTGTGCGGCTGCAGGAGCAAGGAGGGGGAGTC
A04-B57450-G0347484-1_G0347484-1-SEQ2.ab1(1>791)     →  AGCTTCGCCTGCCCTGGCTGTGCGGCTGCAGGAGCAAGGAGGGGGAGTC

                                                        1030      1040      1050      1060      1070
                                                        TCCCGGTCCCATAAAGGGCCCTGCAGCCCCTCCTGCGCCATCCCAACAC
G0347484-1.seq(1>1650)                               →  TCCCGGTCCCATAAAGGGCCCTGCAGCCCCTCCTGCGCCATCCCAACAC
A04-B57450-G0347484-1_G0347484-1-SEQ2.ab1(1>791)     →  TCCCGGTCCCATAAAGGGCCCTGCAGCCCCTCCTGCGCCATCCCAACAC
E03-B57450-G0347484-1_RVP3-R.ab1(1>795)              ←                          GCCCCTCCTGCGCCATCCCAACAC
```

```
                                                     1080      1090      1100      1110      1120
                                                     CGGGGCGGGGGGCTCCCCTGGCTGGGGCCAGACGCCCCCAGGGGCCCTC
G0347484-1.seq(1>1650)                           →   CGGGGCGGGGGGCTCCCCTGGCTGGGGCCAGACGCCCCCAGGGGCCCTC
A04-B57450-G0347484-1_G0347484-1-SEQ2.ab1(1>791) →   CGGGGCGGGGGGCTCCCCTGGCTGGGGCCAGACGCCCCCAGGGGCCCTC
E03-B57450-G0347484-1_RVP3-R.ab1(1>795)          ←   CGGGGCGGGGGGCTCCCCTGGCTGGGGCCAGACGCCCCCAGGGGCCCTC

                                                     1130      1140      1150      1160      1170
                                                     ACAGTCCAGGAGGGGGGCGGGCACCCGGGTTTTCGGGGGCGGGGGGCAG
G0347484-1.seq(1>1650)                           →   ACAGTCCAGGAGGGGGGCGGGCACCCGGGTTTTCGGGGGCGGGGGGCAG
A04-B57450-G0347484-1_G0347484-1-SEQ2.ab1(1>791) →   ACAGTCCAGGAGGGGGGCGGGCACCCGGGTTTTCGGGGGCGGGGGGCAG
E03-B57450-G0347484-1_RVP3-R.ab1(1>795)          ←   ACAGTCCAGGAGGGGGGCGGGCACCCGGGTTTTCGGGGGCGGGGGGCAG

                                                     1180      1190      1200      1210      1220
                                                     GGACTCGGGGACCTGAGAGCCCGAGCGGCCCCCAAACAAACCCAGCCGA
G0347484-1.seq(1>1650)                           →   GGACTCGGGGACCTGAGAGCCCGAGCGGCCCCCAAACAAACCCAGCCGA
A04-B57450-G0347484-1_G0347484-1-SEQ2.ab1(1>791) →   GGACTCGGGGACCTGAGAGCCCGAGCGGCCCCCAAACAAACCCAGCCGA
E03-B57450-G0347484-1_RVP3-R.ab1(1>795)          ←   GGACTCGGGGACCTGAGAGCCCGAGCGGCCCCCAAACAAACCCAGCCGA

                                                     1230      1240      1250      1260      1270
                                                     AGAAGGCGCCCCGCCACACCGCAACGGTCAATGCCGCTTTTCTGCGAAA
G0347484-1.seq(1>1650)                           →   AGAAGGCGCCCCGCCACACCGCAACGGTCAATGCCGCTTTTCTGCGAAA
A04-B57450-G0347484-1_G0347484-1-SEQ2.ab1(1>791) →   AGAAGGCGCCCCGCCACACCGCAACGGTCAATGCCGCTTTTCTGCGAAA
E03-B57450-G0347484-1_RVP3-R.ab1(1>795)          ←   AGAAGGCGCCCCGCCACACCGCAACGGTCAATGCCGCTTTTCTGCGAAA

                                                     1280      1290      1300      1310      1320
                                                     GGGCAATTCCGCTAAGCGGCTTCGGCACCCTCGCACGCGGAGTCACCAC
G0347484-1.seq(1>1650)                           →   GGGCAATTCCGCTAAGCGGCTTCGGCACCCTCGCACGCGGAGTCACCAC
A04-B57450-G0347484-1_G0347484-1-SEQ2.ab1(1>791) →   GGGCAATTCCGCTAAGCGGCTTCGGCACCCTCGCACGCGGAGTCACCAC
E03-B57450-G0347484-1_RVP3-R.ab1(1>795)          ←   GGGCAATTCCGCTAAGCGGCTTCGGCACCCTCGCACGCGGAGTCACCAC

                                                     1330      1340      1350      1360      1370
                                                     GCCCCGTCTCTGATTGGCTGGCGGCGCCAGCTCCCGGCCCGCCGTTGGC
G0347484-1.seq(1>1650)                           →   GCCCCGTCTCTGATTGGCTGGCGGCGCCAGCTCCCGGCCCGCCGTTGGC
A04-B57450-G0347484-1_G0347484-1-SEQ2.ab1(1>791) →   GCCCCGTCTCTGATTGGCTGGCGGCGCCAGCTCCCGGCCCGCCGTTGGC
E03-B57450-G0347484-1_RVP3-R.ab1(1>795)          ←   GCCCCGTCTCTGATTGGCTGGCGGCGCCAGCTCCCGGCCCGCCGTTGGC

                                                     1380      1390      1400      1410      1420
                                                     TGAACTCAACTCAACACCCGCCCCCTACCTCCTCTCCGCCGTTACCAGG
G0347484-1.seq(1>1650)                           →   TGAACTCAACTCAACACCCGCCCCCTACCTCCTCTCCGCCGTTACCAGG
A04-B57450-G0347484-1_G0347484-1-SEQ2.ab1(1>791) →   TGAACTCAACTCAACACCCGCCCCCTACCTCCTCTCCGCCGTTACCAGG
E03-B57450-G0347484-1_RVP3-R.ab1(1>795)          ←   TGAACTCAACTCAACACCCGCCCCCTACCTCCTCTCCGCCGTTACCAGG

                                                     1430      1440      1450      1460      1470
                                                     CAGACCAGCTCCCGTGACGCGTTCCGCCCCCGCCCGCTCAGATTGACGG
G0347484-1.seq(1>1650)                           →   CAGACCAGCTCCCGTGACGCGTTCCGCCCCCGCCCGCTCAGATTGACGG
A04-B57450-G0347484-1_G0347484-1-SEQ2.ab1(1>791) →   CAGACCAGCTCCCGTGACGCGTTCCGCCCCCGCCCGCTCAGATTGACGG
E03-B57450-G0347484-1_RVP3-R.ab1(1>795)          ←   CAGACCAGCTCCCGTGACGCGTTCCGCCCCCGCCCGCTCAGATTGACGG

                                                     1480      1490      1500      1510
                                                     GCAGCTCGGCCAACCCGCACTAGGTCCCGCCCCTCCAGTTCTTTTGCGC
G0347484-1.seq(1>1650)                           →   GCAGCTCGGCCAACCCGCACTAGGTCCCGCCCCTCCAGTTCTTTTGCGC
A04-B57450-G0347484-1_G0347484-1-SEQ2.ab1(1>791) →   GCAGCTCGGCCAACCCGCACTAGGTCCCGCCCCTCCAGTTCTTTTGCGC
E03-B57450-G0347484-1_RVP3-R.ab1(1>795)          ←   GCAGCTCGGCCAACCCGCACTAGGTCCCGCCCCTCCAGTTCTTTTGCGC

                                                     1520      1530      1540      1550      1560
                                                     GCGCCTGATTCCGCCCGGAGGGAGGCGGGACTTCTAACTCGCCGCGCCG
G0347484-1.seq(1>1650)                           →   GCGCCTGATTCCGCCCGGAGGGAGGCGGGACTTCTAACTCGCCGCGCCG
A04-B57450-G0347484-1_G0347484-1-SEQ2.ab1(1>791) →   GCGCCTGATTCCGCC
E03-B57450-G0347484-1_RVP3-R.ab1(1>795)          ←   GCGCCTGATTCCGCCCGGAGGGAGGCGGGACTTCTAACTCGCCGCGCCG
```

```
                                                  1570      1580      1590      1600      1610
                                                  CCAATCCGAAGCCGCCACCTCCCGCAGGAGCCAATGGAGCACAGGCGG
G0347484-1.seq(1>1650)                        →   CCAATCCGAAGCCGCCACCTCCCGCAGGAGCCAATGGAGCACAGGCGG
E03-B57450-G0347484-1_RVP3-R.ab1(1>795)       ←   CCAATCCGAAGCCGCCACCTCCCGCAGGAGCCAATGGAGCACAGGCGG

                                                  1620      1630      1640      1650      1660
                                                  CAACGGGCTCGGCCTCCAAGGGGGAAGGGCGGAGGGAGCGCGCGGCCAA
G0347484-1.seq(1>1650)                        →   CAACGGGCTCGGCCTCCAAGGGGGAAGGGCGGAGGGAGCGCGCGGCCAA
E03-B57450-G0347484-1_RVP3-R.ab1(1>795)       ←   CAACGGGCTCGGCCTCCAAGGGGGAAGGGCGGAGGGAGCGCGCGGCCAA

                                                  1670      1680      1690      1700      1710
                                                  TGAAACGGCGGGAGCTGCGGTCTAGGGCCGGGACACGGCGGCCGTCGCA
G0347484-1.seq(1>1650)                        →   TGAAACGGCGGGAGCTGCGGTCTAGGGCCGGGACACGGCGGCCGTCGCA
E03-B57450-G0347484-1_RVP3-R.ab1(1>795)       ←   TGAAACGGCGGGAGCTGCGGTCTAGGGCCGGGACACGGCGGCCGTCGCA

                                                  1720      1730      1740      1750      1760
                                                  GCCAATGGGAGCGCGGGGCGGGGTGGATTTGAAGCGCGAGGCGGCAGCA
G0347484-1.seq(1>1650)                        →   GCCAATGGGAGCGCGGGGCGGGGTGGATTTGAAGCGCGAGGCGGCAGCA
E03-B57450-G0347484-1_RVP3-R.ab1(1>795)       ←   GCCAATGGGAGCGCGGGGCGGGGTGGATTTGAAGCGCGAGGCGGCAGCA

                                                  1770      1780      1790      1800      1810
                                                  GCAGCCGTGGGTAGGTGAGCGCGGAGGTACCGGGGGCGCAAGCTTGGCA
G0347484-1.seq(1>1650)                        →   GCAGCCGTGGGTAGGTGAGCGCGGAGGTACCGGGGGCGCAAGCTT
E03-B57450-G0347484-1_RVP3-R.ab1(1>795)       ←   GCAGCCGTGGGTAGGTGAGCGCGGAGGTACCGGGGGCGCAAGCTTGGCA

                                                  1820      1830      1840
                                                  TTCCGGTACTGTTGGTAAAGCCACCATGGAAGACGC
E03-B57450-G0347484-1_RVP3-R.ab1(1>795)       ←   TTCCGGTACTGTTGGTAAAGCCACCATGGAAGACGC
```
