# Supplementary material for: Dynamic changes in chromatin accessibility reveal the role of NF-Y targeting AURKB in mediating cell cycle during asynchronous oogenesis in the Chinese Alligator (Alligator sinensis)
Source: Front Zool. 2026 Apr 29;23:24. doi: 10.1186/s12983-026-00611-8 (PMC13274144; doi:10.1186/s12983-026-00611-8)
Supplement: Supplementary file 15 — Additional file15 (PDF 182 KB): AURKB WT promoter Target Gene Sequence. [file 12983_2026_611_MOESM15_ESM.pdf]

5' CTCGAGGGAGCAGTGCATGTGGCTGCACAGCCTCTCCGCAAGGCAGCAAGACCCATGAGAGTGGAGCCTG 70

5' AGCAGTGGATTTAAAACAATTTTTTTTTGGAAAAGTATGTTTTTATTCAAATATTATAAAAGCCTAAGTC 140

5' TGTCTGTCTGTCTGTAACACTTTATTTGTGCTCTGATTGGCTGACAAACAGCCAATCAGAGTGCAAAGCA 210

5' GCATTCTCACAGAAGGCAGCCCTCCGCCTGGATGGTGGGGGCAGGGGACCGGGGGGGGGGAAGGGCCAG 280

5' CAGGGCCCCGTCCCCCTGCAGGTAATGCGGGGTGTGGGAGCGGGCCCGGGCCCACGGTGGTGGGGAGGGG 350

5' AGCAGGCAGGACCCAAGCAGCAGAAGGGAAGCAGGAGCAGGTCGGGGGGGGGGGGAGGGCTGTCCCGCCT 420

5' GTCCCTTCACCCCTGTCATTCTTGACAGGCAATTGGCTAGTAGATGCGTAAAAGTTATACGCATCTCCAC 490

5' CTTCTAGTCACCTGCCATCACAGACCTTCACTCGCACCACAGAGTGCAAAACGCCCGCCCGCCTGCCCGC 560

5' CCGCGGGCGCCCCGCGCCGGTGCAACCTCCGCCGGCCCGAGCACTGGATTTCTTCTTGATTTTTAAGGAG 630

5' ATTTTTTCGTGTCCCGGGTCAAATTAGCCCGATCAGGCCCAAATCCATTAGAATCACGCAAGGACCACAC 700

5' GTGGCCCTGCTACCGGCAAGTGTCCCCGCCCCGCCCCGCCCCGCCGCGCCTTCAGAGGCTTCCAAACCCT 770

5' GTGACGGCCGGCGTCCCGCGCGGGCCCCAGCCCGAGGCCTCGGCTGCCCCGAGCTTCGCCTGCCCTGGCT 840

5' GTGCGGCTGCAGGAGCAAGGAGGGGGAGTCTCCCGGTCCCATAAAGGGCCCTGCAGCCCCTCCTGCGCCA 910

5' TCCCAACACCGGGGCGGGGGGCTCCCCTGGCTGGGGCCAGACGCCCCCAGGGGCCCTCACAGTCCAGGAG 980

5' GGGGGCGGGCACCCGGGTTTTCGGGGGCGGGGGGCAGGGACTCGGGGACCTGAGAGCCCGAGCGGCCCCC 1050

5' AAACAAACCCAGCCGAAGAAGGCGCCCCGCCACACCGCAACGGTCAATGCCGCTTTTCTGCGAAAGGGCA 1120

5' ATTCCGCTAAGCGGCTTCGGCACCCTCGCACGCGGAGTCACCACGCCCCGTCTCTGATTGGCTGGCGGCG 1190

5' CCAGCTCCCGGCCCGCCGTTGGCTGAACTCAACTCAACACCCGCCCCCTACCTCCTCTCCGCCGTTACCA 1260

5' GGCAGACCAGCTCCCGTGACGCGTTCCGCCCCCGCCCGCTCAGATTGACGGGCAGCTCGGCCAACCCGCA 1330

5' CTAGGTCCCGCCCCTCCAGTTCTTTTGCGCGCGCCTGATTCCGCCCGGAGGGAGGCGGGACTTCTAACTC 1400

5' GCCGCGCCGCCAATCCGAAGCCGCCACCTCCCGCAGGAGCCAATGGGAGCACAGGCGGCAACGGGCTCGG 1470

5' CCTCCAAGGGGGAAGGGCGGAGGGAGCGCGCGGCCAATGAAACGGCGGGAGCTGCGGTCTAGGGCCGGGA 1540

5' CACGGCGGCCGTCGCAGCCAATGGGAGCGCGGGGCGGGGTGGATTTGAAGCGCGAGGCGGCAGCAGCAGC 1610

5' CGTGGGTAGGTGAGCGCGGAGGTACCGGGGGCGCAAGCTT 1650
