# Supplementary material for: Dynamic changes in chromatin accessibility reveal the role of NF-Y targeting AURKB in mediating cell cycle during asynchronous oogenesis in the Chinese Alligator (Alligator sinensis)
Source: Front Zool. 2026 Apr 29;23:24. doi: 10.1186/s12983-026-00611-8 (PMC13274144; doi:10.1186/s12983-026-00611-8)
Supplement: Supplementary file 16 — Additional file16 (PDF 97 KB): NFYA in pcDNA3.1(+). [file 12983_2026_611_MOESM16_ESM.pdf]

```
LOCUS       Exported                6468 bp ds-DNA    circular SYN 27-12月-2024
DEFINITION  .
ACCESSION   .
VERSION     .
KEYWORDS    Untitled 21
SOURCE      synthetic DNA construct
  ORGANISM  synthetic DNA construct
REFERENCE   1  (bases 1 to 6468)
  AUTHORS   111111111
  TITLE     Direct Submission
  JOURNAL   Exported 2024年12月27日 from SnapGene 2.3.2
            http://www.snapgene.com
FEATURES             Location/Qualifiers
     source          1..6468
                     /organism="synthetic DNA construct"
                     /mol_type="other DNA"
     enhancer        235..614
                     /note="CMV enhancer"
                     /note="human cytomegalovirus immediate early enhancer"
     promoter        615..818
                     /note="CMV promoter"
                     /note="human cytomegalovirus (CMV) immediate early
                     promoter"
     promoter        863..881
                     /note="T7 promoter"
                     /note="promoter for bacteriophage T7 RNA polymerase"
     misc_feature    895..1953
                     /note="NFYA"
     polyA_signal    2065..2289
                     /note="bGH poly(A) signal"
                     /note="bovine growth hormone polyadenylation signal"
     rep_origin      2335..2763
                     /direction=RIGHT
                     /note="f1 ori"
                     /note="f1 bacteriophage origin of replication; arrow
                     indicates direction of (+) strand synthesis"
     promoter        2777..3106
                     /note="SV40 promoter"
                     /note="SV40 enhancer and early promoter"
     rep_origin      2957..3092
                     /note="SV40 ori"
                     /note="SV40 origin of replication"
     CDS             3173..3967
                     /codon_start=1
                     /gene="aph(3')-II (or nptII)"
                     /product="aminoglycoside phosphotransferase from Tn5"
                     /note="NeoR/KanR"
                     /note="confers resistance to neomycin, kanamycin, and G418
                     (Geneticin(R))"
                     /translation="MIEQDGLHAGSPAAWVERLFGYDWAQQTIGCSDAAVFRLSAQGRP
                     VLFVKTDLSGALNELQDEAARLSWLATTGVPCAAVLDVVTEAGRDWLLLGEVPGQDLLS
                     SHLAPAEKVSIMADAMRRLHTLDPATCPFDHQAKHRIERARTRMEAGLVDQDDLDEEHQ
                     GLAPAELFARLKARMPDGEDLVVTHGDACLPNIMVENGRFSGFIDCGRLGVADRYQDIA
                     LATRDIAEELGGEWADRFLVLYGIAAPDSQRIAFYRLLDEFF"
     polyA_signal    4141..4262
                     /note="SV40 poly(A) signal"
                     /note="SV40 polyadenylation signal"
     primer_bind     complement(4311..4327)
                     /note="M13 rev"
                     /note="common sequencing primer, one of multiple similar
                     variants"
     protein_bind    4335..4351
                     /bound_moiety="lac repressor encoded by lacI"
                     /note="lac operator"
                     /note="The lac repressor binds to the lac operator to
                     inhibit transcription in E. coli. This inhibition can be
                     relieved by adding lactose or
                     isopropyl-beta-D-thiogalactopyranoside (IPTG)."
     promoter        complement(4359..4389)
                     /note="lac promoter"
                     /note="promoter for the E. coli lac operon"
     protein_bind    4404..4425
                     /bound_moiety="E. coli catabolite activator protein"
```

```
                     /note="CAP binding site"
                     /note="CAP binding activates transcription in the presence
                     of cAMP."
     rep_origin      complement(4713..5301)
                     /direction=LEFT
                     /note="ori"
                     /note="high-copy-number ColE1/pMB1/pBR322/pUC origin of
                     replication"
     CDS             complement(5472..6332)
                     /codon_start=1
                     /gene="bla"
                     /product="beta-lactamase"
                     /note="AmpR"
                     /note="confers resistance to ampicillin, carbenicillin, and
                     related antibiotics"
                     /translation="MSIQHFRVALIPFFAAFCLPVFAHPETLVKVKDAEDQLGARVGYI
                     ELDLNSGKILESFRPEERFPMMSTFKVLLCGAVLSRIDAGQEQLGRRIHYSQNDLVEYS
                     PVTEKHLTDGMTVRELCSAAITMSDNTAANLLLTTIGGPKELTAFLHNMGDHVTRLDRW
                     EPELNEAIPNDERDTTMPVAMATTLRKLLTGELLTLASRQQLIDWMEADKVAGPLLRSA
                     LPAGWFIADKSGAGERGSRGIIAALGPDGKPSRIVVIYTTGSQATMDERNRQIAEIGAS
                     LIKHW"
     promoter        complement(6333..6437)
                     /gene="bla"
                     /note="AmpR promoter"
ORIGIN
        1 gacggatcgg gagatctccc gatcccctat ggtgcactct cagtacaatc tgctctgatg
       61 ccgcatagtt aagccagtat ctgctccctg cttgtgtgtt ggaggtcgct gagtagtgcg
      121 cgagcaaaat ttaagctaca acaaggcaag gcttgaccga caattgcatg aagaatctgc
      181 ttagggttag gcgttttgcg ctgcttcgcg atgtacgggc cagatatacg cgttgacatt
      241 gattattgac tagttattaa tagtaatcaa ttacggggtc attagttcat agcccatata
      301 tggagttccg cgttacataa cttacggtaa atggcccgcc tggctgaccg cccaacgacc
      361 cccgcccatt gacgtcaata atgacgtatg ttcccatagt aacgccaata gggactttcc
      421 attgacgtca atgggtggag tatttacggt aaactgccca cttggcagta catcaagtgt
      481 atcatatgcc aagtacgccc cctattgacg tcaatgacgg taaatggccc gcctggcatt
      541 atgcccagta catgacctta tgggactttc ctacttggca gtacatctac gtattagtca
      601 tcgctattac catggtgatg cggttttggc agtacatcaa tgggcgtgga tagcggtttg
      661 actcacgggg atttccaagt ctccacccca ttgacgtcaa tgggagtttg ttttggcacc
      721 aaaatcaacg ggactttcca aaatgtcgta acaactccgc cccattgacg caaatgggcg
      781 gtaggcgtgt acggtgggag gtctatataa gcagagctct ctggctaact agagaaccca
      841 ctgcttactg gcttatcgaa attaatacga ctcactatag ggagacccaa gctggctagc
      901 gccaccatgg aacagtacac agcaaacagc aacagttcta cagaacaaat tgttgtgcaa
      961 gctggacaga ttcagcagca gcagcagggt ggtgttactg ctgtccagtt gcagacagag
     1021 gcccaggtgg catccgcctc aggccagcaa gtccagaccc tccaggtcca aggtcagccg
     1081 ttaatggtac aagtaagcgg aggtcagctg atcacatcaa ccggccagcc aatcatggtg
     1141 caagctgtgc ctggaggtca aggccagaca atcatgcaag tcccggtttc tggaacgcaa
     1201 ggattgcagc agattcagtt ggtccagcca ggtcagattc agattcaagg tgggcaggcg
     1261 gtgcaggtcc agggtcaaca gggccagacc cagcaaatca ttattcagca gccacagact
     1321 gcagttactg ctggccagac gcagacccag cagcaaatag cagttcaagg gcagcaggtg
     1381 gcacagacag cagaaggcca gaccatcgtc tatcagcctg ttaatgctga tggcaccatt
     1441 cttcaacaag ttacagtccc tgttacaggc atgatcacca tccccgcagc cagtttggct
     1501 ggagcacaga ttgtccagac gggagccaac accaacacaa ccagcagtgg gcaaggaact
     1561 gtaactgtga cactaccagt tgctggaaat gtggtcaatt cgggtggaat ggttatgatg
     1621 gtaccaggag ctggatcagt gccagctatc cagaggattc ctttgcctgg agcagaaatg
     1681 cttgaagagg agcccctgta tgtaaatgcc aagcagtacc accggattct gaagagaaga
     1741 caggcacgag ctaagctgga agctgaaggg aaaatcccca aagaaagaag gaaatacttg
     1801 catgaatccc gacatcgtca tgctatggct aggaagcggg gagaaggtgg ccgtttcttc
     1861 tccccaaagg aaaaggatag tccccatatg tcggatccaa ctcaagcaaa tgaagaagca
     1921 atgacacaga tgatcagagt atcctaaaag cttggtaccg agctcggatc cactagtcca
     1981 gtgtggtgga attctgcaga tatccagcac agtggcggcc gctcgagtct agagggcccg
     2041 tttaaacccg ctgatcagcc tcgactgtgc cttctagttg ccagccatct gttgtttgcc
     2101 cctcccccgt gccttccttg accctggaag gtgccactcc cactgtcctt tcctaataaa
     2161 atgaggaaat tgcatcgcat tgtctgagta ggtgtcattc tattctgggg ggtggggtgg
     2221 ggcaggacag caagggggag gattgggaag acaatagcag gcatgctggg gatgcggtgg
     2281 gctctatggc ttctgaggcg gaaagaacca gctggggctc tagggggtat ccccacgcgc
     2341 cctgtagcgg cgcattaagc gcggcgggtg tggtggttac gcgcagcgtg accgctacac
     2401 ttgccagcgc cctagcgccc gctcctttcg ctttcttccc ttcctttctc gccacgttcg
     2461 ccggctttcc ccgtcaagct ctaaatcggg ggctcccttt agggttccga tttagtgctt
     2521 tacggcacct cgaccccaaa aaacttgatt agggtgatgg ttcacgtagt gggccatcgc
     2581 cctgatagac ggtttttcgc cctttgacgt tggagtccac gttctttaat agtggactct
     2641 tgttccaaac tggaacaaca ctcaacccta tctcggtcta ttcttttgat ttataaggga
     2701 ttttgccgat ttcggcctat tggttaaaaa atgagctgat ttaacaaaaa tttaacgcga
     2761 attaattctg tggaatgtgt gtcagttagg gtgtggaaag tccccaggct ccccagcagg
     2821 cagaagtatg caaagcatgc atctcaatta gtcagcaacc aggtgtggaa agtccccagg
```

```
     2881 ctccccagca ggcagaagta tgcaaagcat gcatctcaat tagtcagcaa ccatagtccc
     2941 gcccctaact ccgcccatcc cgcccctaac tccgcccagt tccgcccatt ctccgcccca
     3001 tggctgacta attttttta tttatgcaga ggccgaggcc gcctctgcct ctgagctatt
     3061 ccagaagtag tgaggaggct tttttggagg cctaggcttt tgcaaaaagc tcccgggagc
     3121 ttgtatatcc attttcggat ctgatcaaga gacaggatga ggatcgtttc gcatgattga
     3181 acaagatgga ttgcacgcag gttctccggc cgcttgggtg gagaggctat tcggctatga
     3241 ctgggcacaa cagacaatcg gctgctctga tgccgccgtg ttccggctgt cagcgcaggg
     3301 gcgcccggtt ctttttgtca agaccgacct gtccggtgcc ctgaatgaac tgcaggacga
     3361 ggcagcgcgg ctatcgtggc tggccacgac gggcgttcct tgcgcagctg tgctcgacgt
     3421 tgtcactgaa gcgggaaggg actggctgct attgggcgaa gtgccggggc aggatctcct
     3481 gtcatctcac cttgctcctg ccgagaaagt atccatcatg gctgatgcaa tgcggcggct
     3541 gcatacgctt gatccggcta cctgcccatt cgaccaccaa gcgaaacatc gcatcgagcg
     3601 agcacgtact cggatggaag ccggtcttgt cgatcaggat gatctggacg aagagcatca
     3661 ggggctcgcg ccagccgaac tgttcgccag gctcaaggcg cgcatgcccg acggcgagga
     3721 tctcgtcgtg acccatggcg atgcctgctt gccgaatatc atggtggaaa atggccgctt
     3781 ttctggattc atcgactgtg gccggctggg tgtggcggac cgctatcagg acatagcgtt
     3841 ggctacccgt gatattgctg aagagcttgg cggcgaatgg gctgaccgct tcctcgtgct
     3901 ttacggtatc gccgctcccg attcgcagcg catcgccttc tatcgccttc ttgacgagtt
     3961 cttctgagcg ggactctggg gttcgaaatg accgaccaag cgacgcccaa cctgccatca
     4021 cgagatttcg attccaccgc cgccttctat gaaaggttgg gcttcggaat cgttttccgg
     4081 gacgccggct ggatgatcct ccagcgcggg gatctcatgc tggagttctt cgcccacccc
     4141 aacttgttta ttgcagctta taatggttac aaataaagca atagcatcac aaatttcaca
     4201 aataaagcat ttttttcact gcattctagt tgtggtttgt ccaaactcat caatgtatct
     4261 tatcatgtct gtataccgtc gacctctagc tagagcttgg cgtaatcatg gtcatagctg
     4321 tttcctgtgt gaaattgtta tccgctcaca attccacaca acatacgagc cggaagcata
     4381 aagtgtaaag cctggggtgc ctaatgagtg agctaactca cattaattgc gttgcgctca
     4441 ctgcccgctt tccagtcggg aaacctgtcg tgccagctgc attaatgaat cggccaacgc
     4501 gcggggagag gcggtttgcg tattgggcgc tcttccgctt cctcgctcac tgactcgctg
     4561 cgctcggtcg ttcggctgcg gcgagcggta tcagctcact caaaggcggt aatacggtta
     4621 tccacagaat caggggataa cgcaggaaag aacatgtgag caaaaggcca gcaaaaggcc
     4681 aggaaccgta aaaaggccgc gttgctggcg tttttccata ggctccgccc ccctgacgag
     4741 catcacaaaa atcgacgctc aagtcagagg tggcgaaacc cgacaggact ataaagatac
     4801 caggcgtttc cccctggaag ctccctcgtg cgctctcctg ttccgaccct gccgcttacc
     4861 ggatacctgt ccgcctttct cccttcggga agcgtggcgc tttctcatag ctcacgctgt
     4921 aggtatctca gttcggtgta ggtcgttcgc tccaagctgg gctgtgtgca cgaacccccc
     4981 gttcagcccg accgctgcgc cttatccggt aactatcgtc ttgagtccaa cccggtaaga
     5041 cacgacttat cgccactggc agcagccact ggtaacagga ttagcagagc gaggtatgta
     5101 ggcggtgcta cagagttctt gaagtggtgg cctaactacg gctacactag aagaacagta
     5161 tttggtatct gcgctctgct gaagccagtt accttcggaa aaagagttgg tagctcttga
     5221 tccggcaaac aaaccaccgc tggtagcggt ggtttttttg tttgcaagca gcagattacg
     5281 cgcagaaaaa aaggatctca agaagatcct ttgatctttt ctacggggtc tgacgctcag
     5341 tggaacgaaa actcacgtta agggattttg gtcatgagat tatcaaaaag gatcttcacc
     5401 tagatccttt taaattaaaa atgaagtttt aaatcaatct aaagtatata tgagtaaact
     5461 tggtctgaca gttaccaatg cttaatcagt gaggcaccta tctcagcgat ctgtctattt
     5521 cgttcatcca tagttgcctg actccccgtc gtgtagataa ctacgatacg ggagggctta
     5581 ccatctggcc ccagtgctgc aatgataccg cgagacccac gctcaccggc tccagattta
     5641 tcagcaataa accagccagc cggaagggcc gagcgcagaa gtggtcctgc aactttatcc
     5701 gcctccatcc agtctattaa ttgttgccgg gaagctagag taagtagttc gccagttaat
     5761 agtttgcgca acgttgttgc cattgctaca ggcatcgtgg tgtcacgctc gtcgtttggt
     5821 atggcttcat tcagctccgg ttcccaacga tcaaggcgag ttacatgatc ccccatgttg
     5881 tgcaaaaaag cggttagctc cttcggtcct ccgatcgttg tcagaagtaa gttggccgca
     5941 gtgttatcac tcatggttat ggcagcactg cataattctc ttactgtcat gccatccgta
     6001 agatgctttt ctgtgactgg tgagtactca accaagtcat tctgagaata gtgtatgcgg
     6061 cgaccgagtt gctcttgccc ggcgtcaata cgggataata ccgcgccaca tagcagaact
     6121 ttaaaagtgc tcatcattgg aaaacgttct tcggggcgaa aactctcaag gatcttaccg
     6181 ctgttgagat ccagttcgat gtaacccact cgtgcaccca actgatcttc agcatctttt
     6241 actttcacca gcgtttctgg gtgagcaaaa acaggaaggc aaaatgccgc aaaaaaggga
     6301 ataagggcga cacggaaatg ttgaatactc atactcttcc tttttcaata ttattgaagc
     6361 atttatcagg gttattgtct catgagcgga tacatatttg aatgtattta gaaaaataaa
     6421 caaatagggg ttccgcgcac atttccccga aaagtgccac ctgacgtc
//
```
