# Supplementary material for: Dynamic changes in chromatin accessibility reveal the role of NF-Y targeting AURKB in mediating cell cycle during asynchronous oogenesis in the Chinese Alligator (Alligator sinensis)
Source: Front Zool. 2026 Apr 29;23:24. doi: 10.1186/s12983-026-00611-8 (PMC13274144; doi:10.1186/s12983-026-00611-8)
Supplement: Supplementary file 39 — Additional file39 (DOCX 187 KB): Figure S1. The base curves are similar to each other, with GC content ranging from 46.12% to 53.34%. The differences are not significant, and there is no obvious GC separation phenomenon. [file 12983_2026_611_MOESM39_ESM.docx]

| 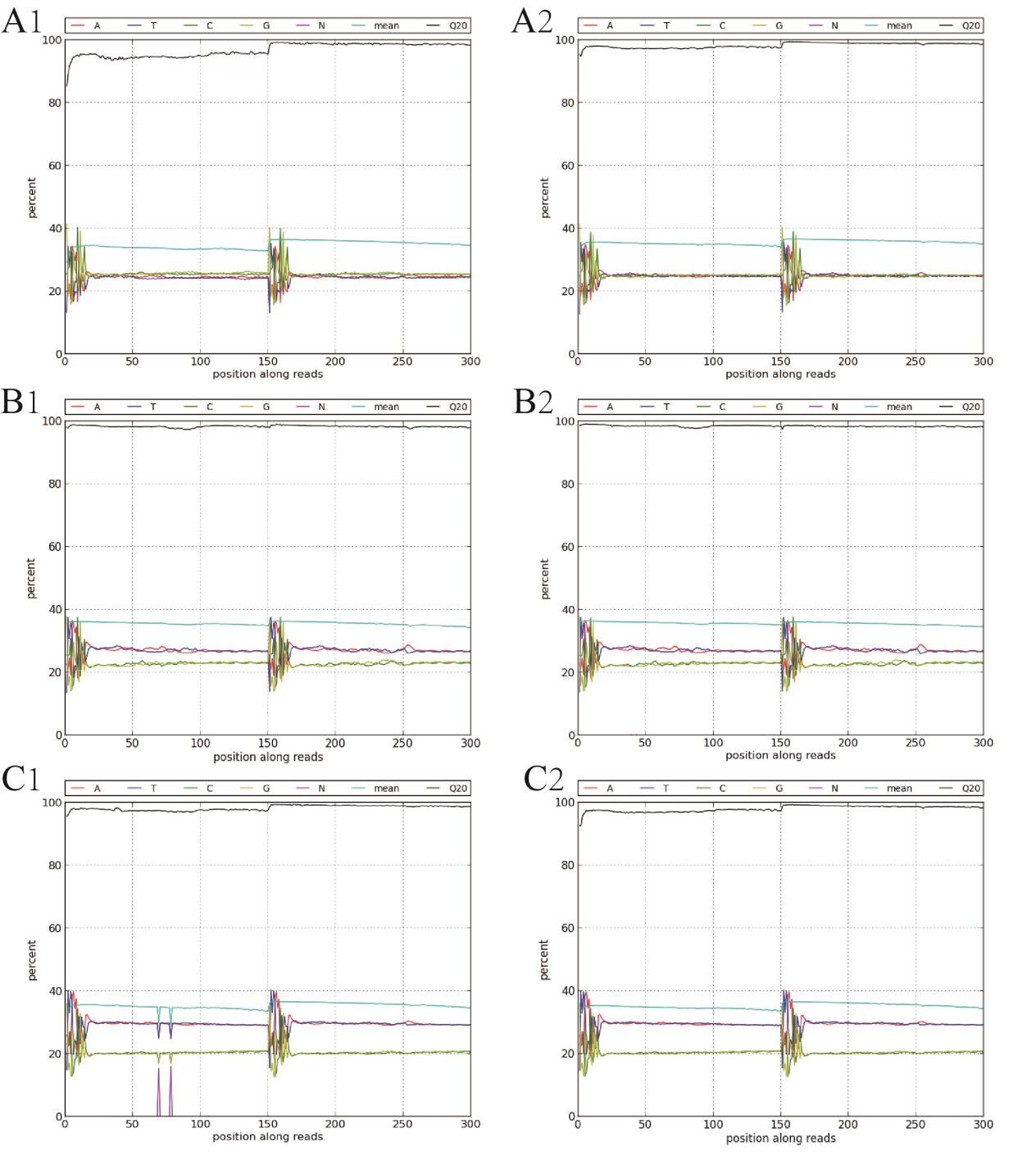 |
| --- |
| **Supplementary Figures 1: Distribution of base composition of each sample after filtering.** The base curves are similar to each other, with GC content ranging from 46.12% to 53.34%. The differences are not significant, and there is no obvious GC separation phenomenon. |
