# Supplementary material for: Dynamic changes in chromatin accessibility reveal the role of NF-Y targeting AURKB in mediating cell cycle during asynchronous oogenesis in the Chinese Alligator (Alligator sinensis)
Source: Front Zool. 2026 Apr 29;23:24. doi: 10.1186/s12983-026-00611-8 (PMC13274144; doi:10.1186/s12983-026-00611-8)
Supplement: Supplementary file 40 — Additional file40 (DOCX 765 KB): Figure S2. The signal is strongest for short fragments (less than 100bp in length), which correspond to DNA fragments bound by histones in open chromatin regions. As the fragment length increases, the signal gradually weakens, and these longer fragments originate from DNA regions with histone binding. [file 12983_2026_611_MOESM40_ESM.docx]

| **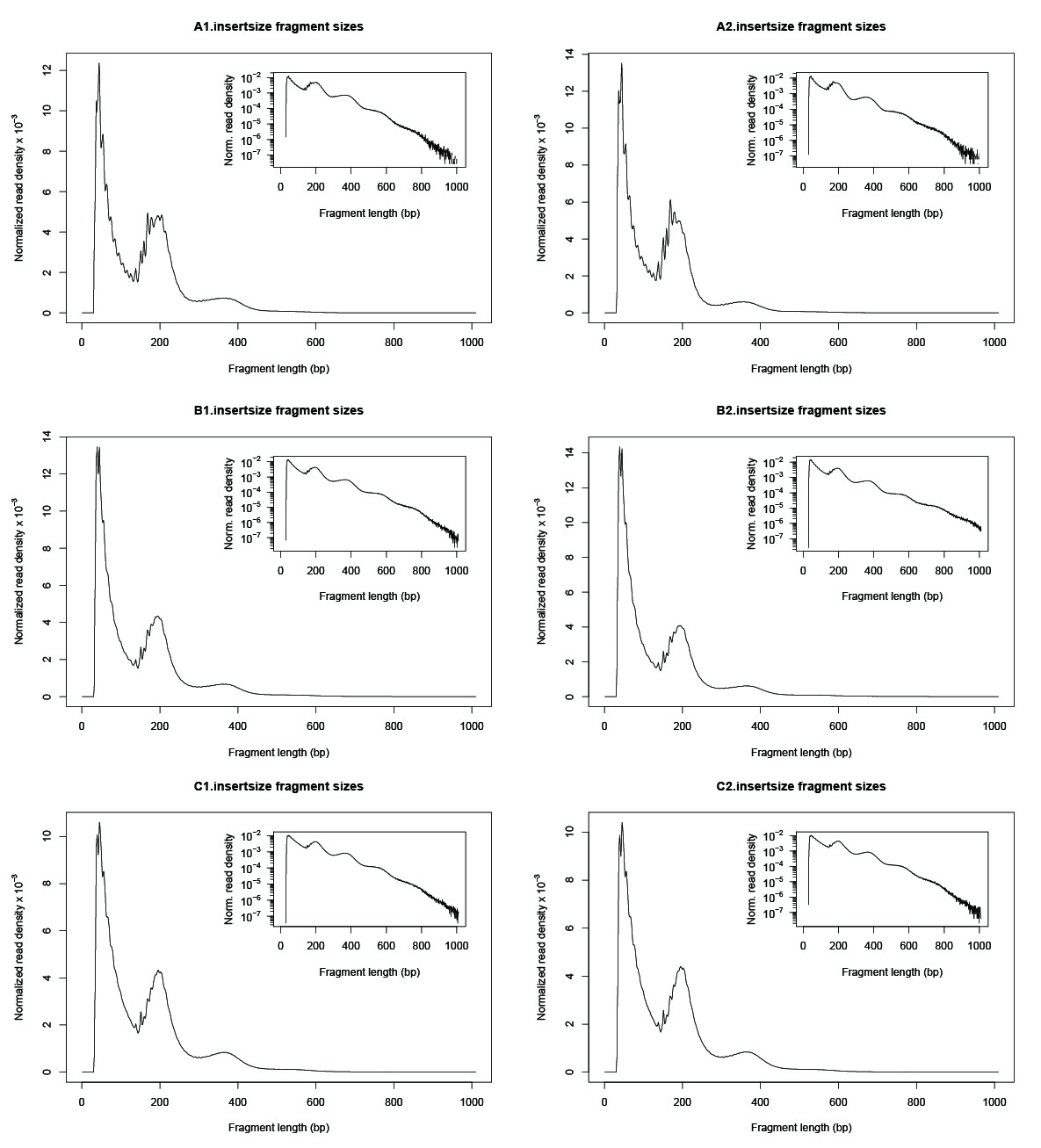** |
| --- |
| **Supplementary Figures 2: Distribution of Inserted Fragment for Each Sample.**  The signal is strongest for short fragments (less than 100bp in length), which correspond to DNA fragments bound by histones in open chromatin regions. As the fragment length increases, the signal gradually weakens, and these longer fragments originate from DNA regions with histone binding. |
