# Supplementary material for: Dynamic changes in chromatin accessibility reveal the role of NF-Y targeting AURKB in mediating cell cycle during asynchronous oogenesis in the Chinese Alligator (Alligator sinensis)
Source: Front Zool. 2026 Apr 29;23:24. doi: 10.1186/s12983-026-00611-8 (PMC13274144; doi:10.1186/s12983-026-00611-8)
Supplement: Supplementary file 41 — Additional file41 (DOCX 339 KB): Figure S3. On the left are the top 20 GO term circle plots of the DARs enriched in the comparisons of AH vs. BH (A), AH vs. CH (C), and BH vs. CH (E). The outermost circle shows the top 20 GO terms, with a gene count scale outside the circle. Different colors indicate distinct Ontologies. The second circle displays the background gene count and Q-value for each GO term. The third circle shows the number of genes enriched in each GO term. The fourth circle presents the RichFactor values of the GO terms. On the right are the top 20 KEGG pathway enrichment plots of the DARs in AH vs. BH (B), AH vs. CH (D), and BH vs. CH (F). [file 12983_2026_611_MOESM41_ESM.docx]

| **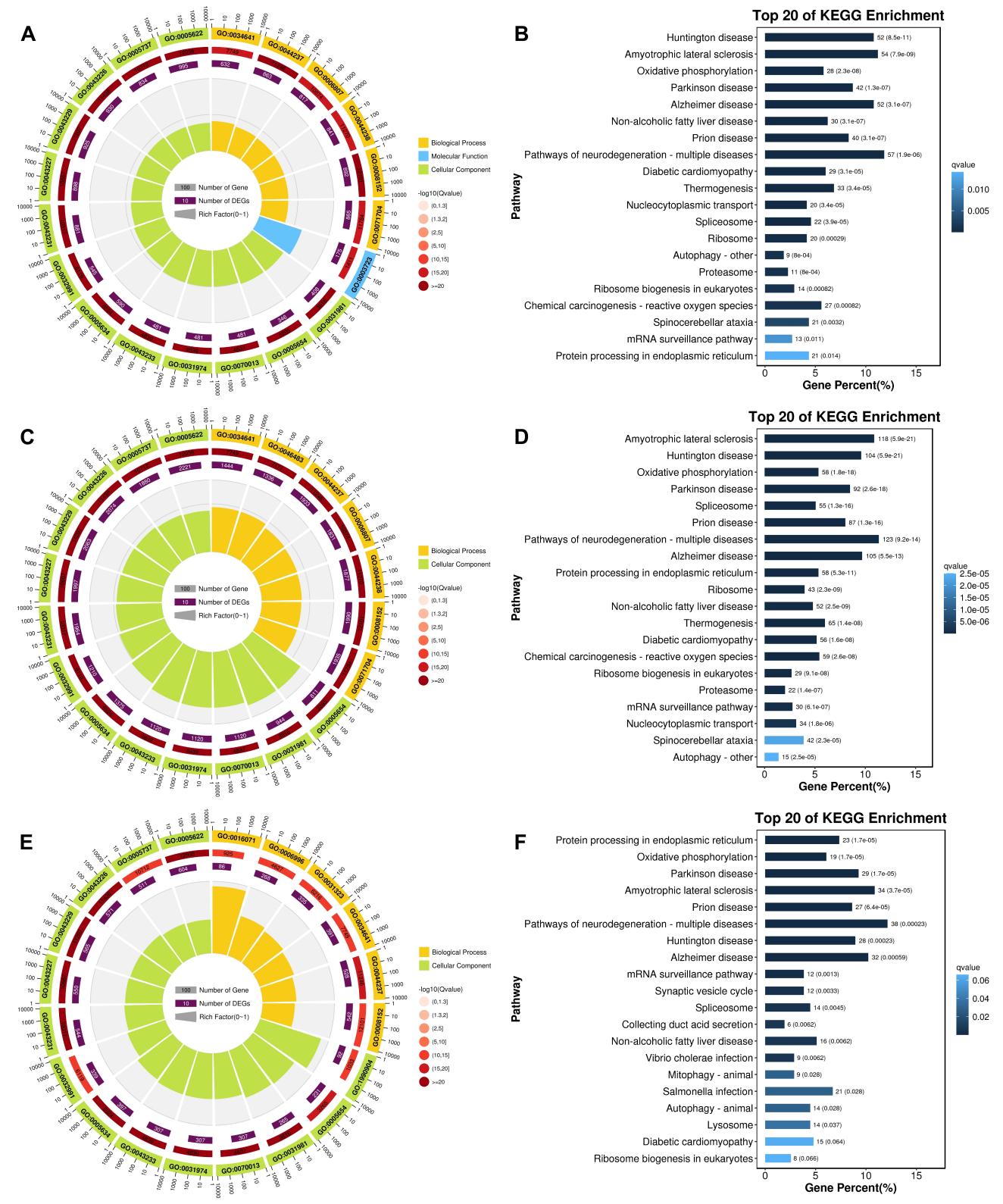** |
| --- |
|  |

**Supplementary Figures 3 GO and KEGG pathway enrichment analysis of the DARs.**

On the left are the top 20 GO term circle plots of the DARs enriched in the comparisons of AH vs. BH (A), AH vs. CH (C), and BH vs. CH (E). The outermost circle shows the top 20 GO terms, with a gene count scale outside the circle. Different colors indicate distinct Ontologies. The second circle displays the background gene count and Q value for each GO term. The third circle shows the number of genes enriched in each GO term. The fourth circle presents the RichFactor values of the GO terms. On the right are the top 20 KEGG pathway enrichment plots of the DARs in AH vs. BH (B), AH vs. CH (D), and BH vs. CH (F).
