# Supplementary material for: Dynamic changes in chromatin accessibility reveal the role of NF-Y targeting AURKB in mediating cell cycle during asynchronous oogenesis in the Chinese Alligator (Alligator sinensis)
Source: Front Zool. 2026 Apr 29;23:24. doi: 10.1186/s12983-026-00611-8 (PMC13274144; doi:10.1186/s12983-026-00611-8)
Supplement: Supplementary file 42 — Additional file42 (DOCX 403 KB): Figure S4. (A) Expression levels of all 724 NF-Y target genes identified by transcription factor footprinting analysis. (B) Expression levels of differentially expressed NF-Y target genes. [file 12983_2026_611_MOESM42_ESM.docx]

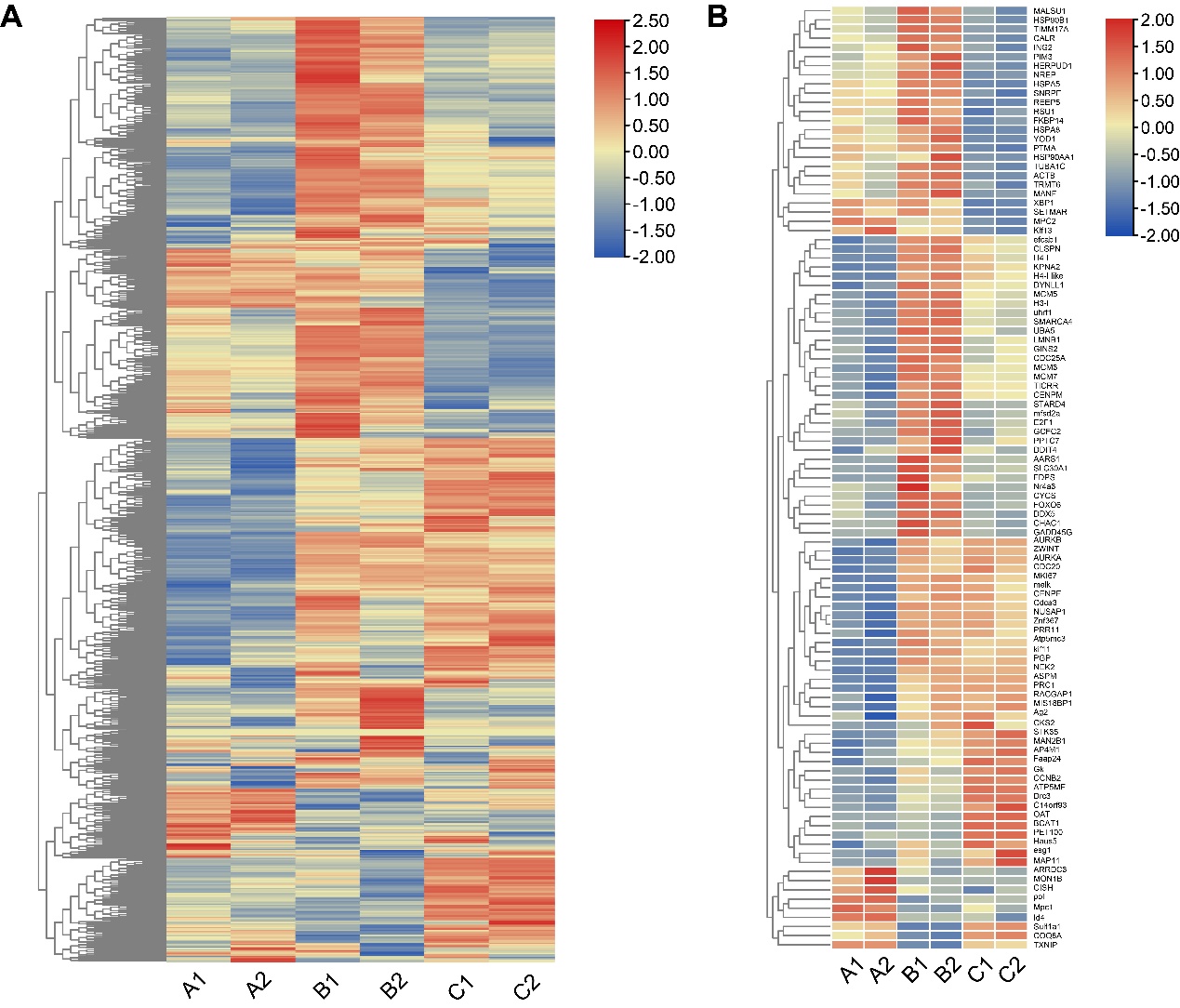


**Supplementary Figures 4: Expression profiles of NF‑Y target genes across developmental stages (AH, BH, CH).**

(A) Expression levels of all 724 NF-Y target genes identified by transcription factor footprinting analysis. (B) Expression levels of differentially expressed NF-Y target genes.
